# Pan-cancer evaluation of regulated cell death to predict overall survival and immune checkpoint inhibitor response

**DOI:** 10.1038/s41698-024-00570-5

**Published:** 2024-03-27

**Authors:** Wei Zhang, Yongwei Zhu, Hongyi Liu, Yihao Zhang, Hongwei Liu, Abraham Ayodeji Adegboro, Ruiyue Dang, Luohuan Dai, Siyi Wanggou, Xuejun Li

**Affiliations:** 1 Department of Neurosurgery, Xiangya Hospital, Central South University, Changsha, China; 2 Hunan International Scientific and Technological Cooperation Base of Brain Tumor Research, Xiangya Hospital, Central South University, Changsha, China; 3 Department of Oncology, Xiangya Hospital, Central South University, Changsha, China

**Keywords:** High-throughput screening, Tumour biomarkers, Cell death and immune response, Immunotherapy

## Abstract

Regulated cell death (RCD) plays a pivotal role in various biological processes, including development, tissue homeostasis, and immune response. However, a comprehensive assessment of RCD status and its associated features at the pan-cancer level remains unexplored. Furthermore, despite significant advancements in immune checkpoint inhibitors (ICI), only a fraction of cancer patients currently benefit from treatments. Given the emerging evidence linking RCD and ICI efficacy, we hypothesize that the RCD status could serve as a promising biomarker for predicting the ICI response and overall survival (OS) in patients with malignant tumors. We defined the RCD levels as the RCD score, allowing us to delineate the RCD landscape across 30 cancer types, 29 normal tissues in bulk, and 2,573,921 cells from 82 scRNA-Seq datasets. By leveraging large-scale datasets, we aimed to establish the positive association of RCD with immunity and identify the RCD signature. Utilizing 7 machine-learning algorithms and 18 ICI cohorts, we developed an RCD signature (RCD.Sig) for predicting ICI response. Additionally, we employed 101 combinations of 10 machine-learning algorithms to construct a novel RCD survival-related signature (RCD.Sur.Sig) for predicting OS. Furthermore, we obtained CRISPR data to identify potential therapeutic targets. Our study presents an integrative framework for assessing RCD status and reveals a strong connection between RCD status and ICI effectiveness. Moreover, we establish two clinically applicable signatures and identify promising potential therapeutic targets for patients with tumors.

## Introduction

Cell death is a fundamental biological process that accompanies various life phenomena, including growth, development, aging, and disease. It can be categorized into related cell death (RCD) and accidental cell death (ACD) depending on the triggering mechanism. Unlike ACD, RCD is a controlled and sequential form of cell death that operates through specific molecular mechanisms, under genetic regulation, and can be modulated through pharmacological or genetic interventions. In 2018, the Nomenclature Committee on Cell Death established guidelines encompassing morphological and biological aspects of cell death, identifying 12 modes of cell death, such as necroptosis and immunogenic cell death^1^. Recent advancements have unveiled additional types of cell death that play crucial roles. These include autosis, cuproptosis, anoikis, disulfidptosis, alkaliptosis, oxeiptosis, and mitotic cell death. Throughout cancer progression, cell death has been identified to function at various stages, and resistance to cell death is considered a key feature of cancer^2^. Importantly, cell death is intricately linked to the response and tolerance of cancer treatments, such as radiotherapy and immunotherapy. High-energy ionizing radiation, for instance, exerts its antitumor effects by inducing ferroptosis, thereby enhancing the sensitivity of tumors to IR therapy^3,4^. Intriguingly, ferroptosis can induce a suppressive immune microenvironment by influencing the migration and polarization of tumor macrophages, consequently reducing the efficacy of immune checkpoint blockade^5^.

Immunotherapy has emerged as a groundbreaking approach in cancer treatment, establishing itself as the “fifth pillar” alongside radiation therapy, chemotherapy, surgery, and targeted therapy. It serves as a new treatment option for patients with advanced cancer^6^. Various immunotherapy strategies are currently employed, including lytic virus therapy, cancer vaccines, cytokine therapy, pericyte transfer, and immune checkpoint inhibitors (ICI)^7^. Throughout tumor development and progression, cancer cells have evolved multiple mechanisms to evade immune surveillance, including the co-inhibition of immune receptors, i.e., immune checkpoints^8^. ICIs reactivate immune cells by blocking the co-inhibitory signaling pathways, enabling them to effectively target tumor cells. This therapy has demonstrated positive efficacy and promising potential in clinical practice^9^. The anti-CTLA-4 and anti-PD-L1 antibodies have received approval for the treatment of various progressive cancer types, including melanoma^10^, non-small cell lung cancer^11^, and renal clear cell carcinoma^12^.

While ICIs have significantly advanced the field of immunotherapy in oncology, several challenges remain. Many patients do not benefit from ICIs, with response rates ranging from 15% to 30% in most solid tumors and 45-60% in melanoma and MSI-H tumors^9^. The incidence of immune-related adverse events (irAE) is notably high and represents a limitation of ICIs. Given the above, the active development of predictive biomarkers to identify patients who could benefit from ICIs treatment or to predict the occurrence of irAE is of utmost value to improve the current landscape of ICIs treatment.

This study represents the first extensive evaluation of RCD levels at a cancer-wide scale, integrating 18 types of RCD and multiple RCD-associated genes. It introduces a tumor RCD measure known as the RCD score and establishes a strong association between RCD, ICI outcomes, and prognosis of tumor patients through an in-depth analysis of large-scale data. Significantly, we have constructed two gene expression signatures, RCD.Sig and RCD.Sur.Sig. The former successfully predicts ICI response across multiple cancer types, while the latter accurately predicts prognosis in patients with various cancer types.

## Results

### Integrative delimitation of RCD levels in cancers and normal tissues

**Fig. 1:**
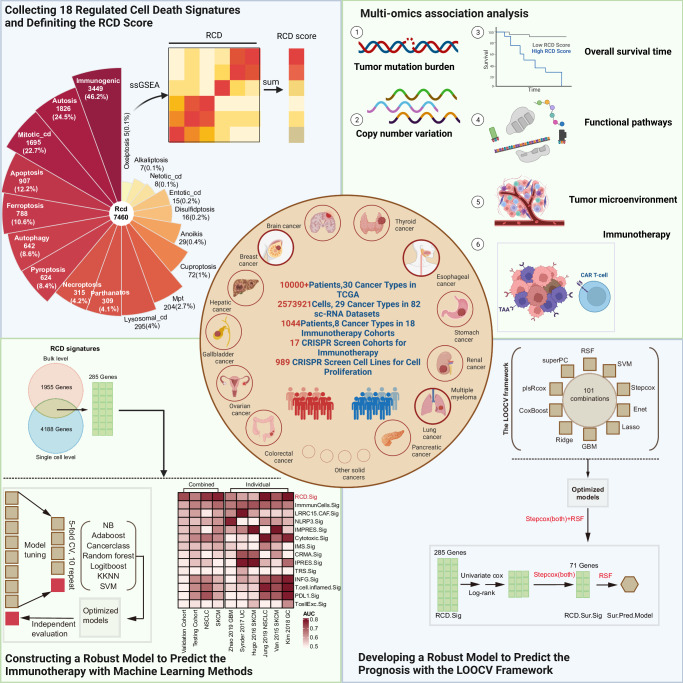
Graphic abstract of this study. Comprehensive evaluation of regulated cell death and integrated development of pan-cancer signatures to predict overall survival and immune checkpoint inhibitor response.

**Fig. 2:**
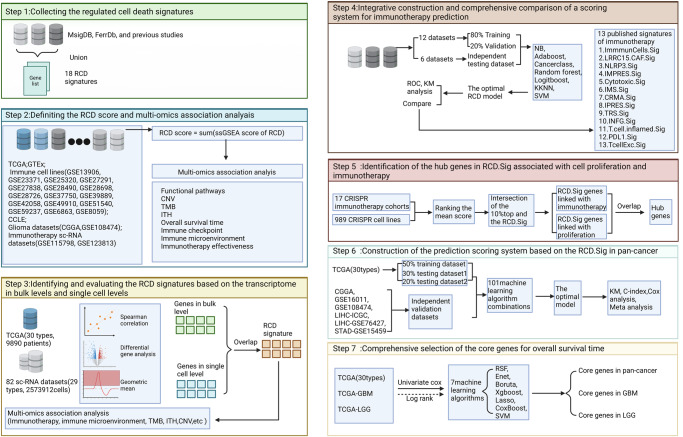
Overall methodology. The workflow of a comprehensive analysis of the RCD landscape across cancers utilizing multi-omics data.

**Fig. 3:**
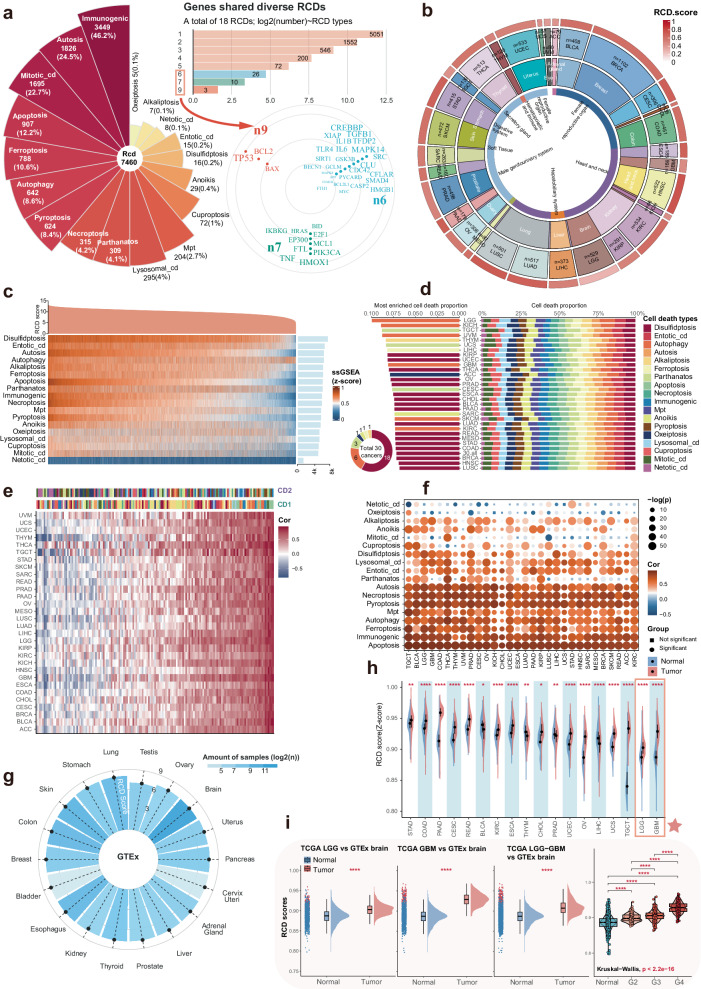
Integrative quantification of regulated cell death levels in cancers and normal tissues. **a** The 18 regulated cell death signatures in this study. Three genes, including TP53, BCL2, and BAX were screened out in 9 RCD types. **b** The circle heat map shows the average of the RCD score in individual cancer types. The average RCD score, cancer types, and tissue types were shown from the outer to the inner side. The RCD score was scaled by zero-mean normalization (Z score). **c** The heat map shows the RCD score and the ssGSEA scores of individual samples in TCGA. The RCD score was defined as the sum of the ssGSEA score of the 18 RCD. The right panel was the bar plot showing the sum of the ssGSEA score of all samples in TCGA. **d** The right panel shows the proportion of ssGSEA score of each RCD across the cancers in TCGA. The middle panel shows the RCD type, which had the highest proportion across the cancers. The left panel shows the RCD types with the highest proportion of RCD types among all cancers. For example, of a total 30 cancers, disulfidptosis had the highest proportion of 18 RCD types in 18 cancer types. **e** The heat map shows the Spearman correlation between any two of the 18 RCD types for the 30 cancers. CD 1 contained 18 types of RCD with the exception of pyroptosis, and CD 2 contained 18 types of RCD with the exception of alkaliptosis. **f** The bubble plot shows the Spearman correlation of the RCD score and the ssGSEA score of the single RCD type. The *p* values < 0.05 were considered significant, while the opposite was considered non-significant. **g** Average RCD score across the normal tissues in the GTEx databank. The darker the blue, the larger the sample size. **h** In most cancers, compared with adjacent normal solid tissue (blue), the RCD score in primary tumors (red) was higher, with utilization of the Wilcoxon rank sum test. **** represented *p* < 0.0001. *** represented *p* < 0.001, ** represented *p* < 0.01. * represented *p* < 0.05. **i** Compared with the normal brain cortex, the RCD score of the LGG, GBM, and glioma (LGG-GBM) was significantly higher in the GTEx and TCGA databases. And the RCD score increased with grade. For Wilcoxon rank sum test, “****”, “***”, “**”, “*” represented *p* < 0.0001, *p* < 0.001, *p* < 0.01, <0.05, respectively.

A graphic abstract and overall framework flow of this study were presented in Figs. 1 and 2, respectively. Among the 18 RCD signatures collected from previous studies, the number of genes ranged from 5 to 3449, with corresponding proportions ranging from 0.1% to 46.2% (Fig. 3a). The intersection of the 9 signatures revealed 3 genes, including TP53, BCL2, and BAX, suggesting the presence of common nodes and hubs between RCD modalities. This finding may partially explain the mutual regulation and influence between cell death modalities and their ability to interconvert in highly heterogeneous tumor microenvironments (TME). The RCD scores were artificially defined as the sum of the ssGSEA scores of all 18 RCD signatures. These scores were used to assess the overall level of RCD signaling in individual samples as a whole, providing a comprehensive dimension of analysis. Several approaches were utilized to demonstrate the robustness and practical value of the RCD score. RCD score was calculated in tumor samples from TCGA and normal tissue samples from GTEx to investigate RCD activity across 30 cancers and their corresponding normal tissues. The overall level of RCD in low-grade glioma (LGG), glioblastoma (GBM), and testicular cancer (TGCT) was comparatively lower than in other cancers (Fig. 3b).

At the pan-cancer level, the disulfidptosis signal, with the highest sum of the scores, appeared to be the core RCD type in the tumors (Fig. 3c). Disulfidptosis and autophagy were the most enriched cell death signature in 18 cancer types and in 6 cancer types, respectively (Fig. 3d).

In all tumors, there was a positive correlation between the majority of RCD modes; however, netotic cell death and oxeiptosis showed a negative correlation with other modes of RCD in some cancer types (Fig. 3e). Regarding the association with the RCD score, netotic cell death and oxeiptosis were negative or not significant in most cancer types, while other RCD modes showed a significant positive correlation or were not significant (Fig. 3f).

We also collected transcriptomic data from cancer cell lines of different tumor types and estimated the RCD level across these tumors based on the cell line data (Supplementary Fig. 1A). The levels of RCD varied considerably between cell lines of different cancer origins. Moreover, the heterogeneity of RCD was not only observed in tumors but also in normal tissues (Fig. 3g). The RCD level was highly elevated in most cancers, including LGG and GBM (Fig. 3h, i). Furthermore, the RCD score increased with increasing glioma grades (Fig. 3i).

The association between RCD levels and functional pathway signals and genomic variations was also investigated at the pan-cancer level. RCD scores were negatively associated with DNA repair, MYC-related genes, and cell cycle signaling, while positively associated with metastasis, stemness, and inflammation (Supplementary fig. 1B–E). These results were consistent with the notion that failures in DNA repair, exceptional expression of MYC transcription factor family members, or abnormal assembly of oncogenic signaling hubs can trigger cell death^13–16^.

Genomic instability, a common characteristic of most cancer cells, was observed in multiple RCD types, such as autophagy^17^, apoptosis^18^, ferroptosis^19^, pyroptosis^20^, and necroptosis^21^. However, the specific relationship between genomic instability and RCD levels requires further investigation. Here, we explored the correlation between the RCD score and copy number variation (CNV) score, tumor mutational burden (TMB), and intra-tumor heterogeneity (ITH). The RCD level was negatively associated with the CNV score in most cancer types (Supplementary Fig. 1F). Furthermore, the RCD level was significantly positively associated with TMB in colon cancer (COAD), thymoma (THTM) and breast cancer (BRCA), while it was significantly negatively associated with TMB in prostate cancer (PRAD), head and neck cancer (HNSC) and lung squamous cell carcinoma (LUSC) (Supplementary Fig. 1G). The median values analysis revealed a positive association between the RCD level and ITH (*R* = 0.51, *p* value = 0.00434) and TMB (*R* = 0.67, *p* value = 0.0000495), while showing no association with the CNV score (*R* = −0.14, *p* value = 0.471) (Supplementary Fig. 1H–J).

In summary, RCD levels exhibited heterogeneity at the pan-cancer level in both tumor and normal tissues. Notably, RCD levels were higher in tumors compared to normal tissues, particularly in gliomas. The robustness of the RCD score, along with the highly positive correlation between the RCD score and ITH/TMB, and the negative correlation between the RCD score and CNV score in most cancer types, suggests that CNV is more likely to be increased in samples with low RCD levels. Therefore, the RCD level, as an effective complement to TMB and ITH, may serve as another important biomarker for tumor ICI therapy.

### Cancer type-specific association of RCD levels with tumor immunity

Tumor immunity plays a critical role in tumor prognosis and development. We investigated the impact of RCD-level heterogeneity on cancer prognosis using datasets from TCGA and validated our findings with independent datasets. Our results demonstrate that the heterogeneity of RCD level may significantly impact clinical outcomes in various tumors, particularly in brain tumors. Therefore, RCD-level heterogeneity could serve as a feasible and robust predictor for prognosis in several cancers (Supplementary Fig. 2A–F).

**Fig. 4:**
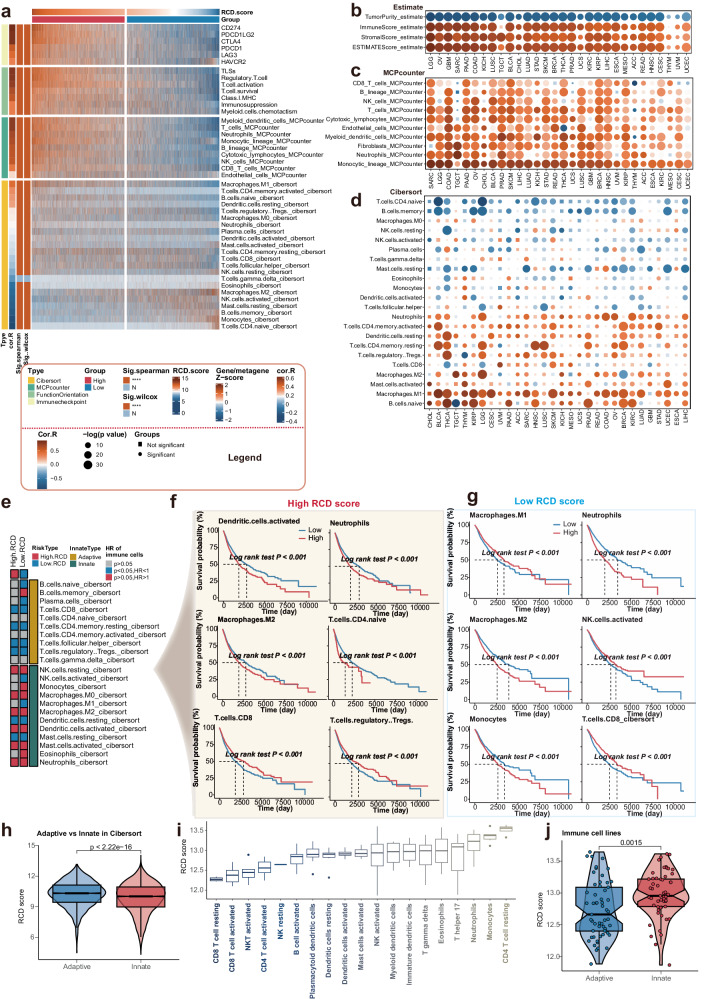
Cancer type-specific association of RCD levels with tumor immunity. The RCD score exhibited strong relevance with tumor immunity. **a** The heat map showing the Spearman correlation of RCD score and tumor immunity including the TME defined by the MCP-counter *Z* scores, the infiltrating immune cells defined by the CIBERSORT algorithm, expression of gene signatures associated with the functional orientation of the immune TME, expression of genes linked with the immune checkpoints. Based on the median RCD score, the pan-cancer cohort of TCGA was divided into two subgroups, named the high RCD subgroup and the low RCD subgroup. The difference of the tumor immunity, as mentioned previously between the high RCD subgroup and low RCD subgroup was estimated with the Wilcoxon rank sum test. “****” indicated the *p* value < 0.001, and “*N*” indicated the *p* > 0.05, which was considered as nonsense. **b** Spearman correlations (color) between RCD scores and the ESTIMATE score, immune score, and stromal score investigated by ESTIMATE algorithm for individual TCGA cancer types. **c** Spearman correlations (color) between RCD scores and the infiltrating immune cells estimated by MCP-counter algorithm for individual TCGA cancer types. **d** Spearman correlations (color) between RCD scores and the absolute abundance of 22 immune cell types estimated by CIBERSORT for individual TCGA cancer types. **e** HRs represent the association between the 22 known immune cell types and OS in the high RCD subgroup and low RCD subgroup. Red indicated higher risk, blue indicated lower risk, and gray indicated nonsense. **f** Kaplan–Meier curves of OS between the subgroups stratified by the infiltration of several representative immune cells in the high RCD subgroup. **g** Kaplan–Meier curves of OS between the subgroups stratified by the infiltration of several representative immune cells in the low RCD subgroup. **h** The difference in the RCD score between the adaptive immune cells and innate immune cells estimated by CIBERSORT algorithm was investigated by the Wilcoxon rank sum test. **i** The box plot showing the RCD scores of the multiple purified immune cells. **j** The graph shows the difference in the RCD score between the adaptive immune cells and innate immune cells extracted from the purified immune cell lines. The Wilcoxon rank sum test was performed to estimate the difference.

To explore the influence of RCD level on tumor immunity, we employed several approaches. Immune markers such as PD-L1 expression have been recognized as predictive of ICI responsiveness and improved clinical outcomes^22^. Therefore, we examined the association between RCD scores and the expression of immune checkpoints, including CD274, CTLA4, and so on. Our results suggest a positive correlation between RCD scores and the expression of these genes. Additionally, we assessed the relationship between RCD levels and the activity of TLSs, regulatory T cell, T-cell activation, T-cell survival, class I MHC, immunosuppression, and myeloid cell chemotaxis. Significantly positive associations were observed (Fig. 4a).

Furthermore, we examined the infiltration immune cells at the pan-cancer level using the MCP-counter algorithm and the CIBERSORT cell deconvolution method. The results revealed that immune cell populations defined by the MCP counter exhibited an increasing trend from immune depletion to immune-enrichment as RCD levels increased. However, the association between RCD levels and infiltrating immune cells, as estimated by CIBERSORT, showed a more complex relationship. Antitumor-related immune cells such as M1 macrophages, activated memory CD4 T cells, and resting dendritic cells demonstrated a strong positive correlation with RCD scores, whereas classical tumor-promoting immune cells like M2 macrophages were negatively associated with RCD levels (Fig. 4a).

Additionally, we assessed the correlation between RCD scores and immune characteristics in individual cancer types. We found a highly positive relationship between RCD scores and ESTIMATE scores, stromal scores, and immune scores across various cancers. Conversely, an inverse association was observed for the tumor purity (Fig. 4b). The infiltrating immune cells calculated by the MCP counter showed a highly positive correlation with RCD scores in nearly all cancer types (Fig. 4c).

To evaluate the potential crosstalk between RCD levels and immune cells, as estimated by CIBERSORT, we examined their impact on prognosis. In the high and low RCD subgroups, 12 and 19 immune cell types, respectively, were significantly associated with OS (Fig. 4e–g, supplementary table 1). Interestingly, most immune cell types in adaptive immunity exhibited a protective effect on OS, whereas the opposite trend was observed in innate immunity (Fig. 4e).

Understanding the two cooperative and complementary immune systems, namely innate immunity and adaptive immunity, is crucial for developing effective immunotherapies that can overcome tumor-induced immune suppression and target tumors for elimination. We compared the RCD levels in adaptive immunity and innate immunity, as estimated by the CIBERSORT algorithm. Our findings indicate that the RCD level in adaptive immunity is significantly higher than in innate immunity. Additionally, using purified immune cell lines, we calculated RCD scores across 19 immune cell lines, categorizing them into innate immune cells and adaptive cells based on their functions (Fig. 4i, supplementary Fig. 3A, B). Interestingly, the RCD level in adaptive immunity was lower than in innate immunity, contradicting the results obtained from CIBERSORT (Fig. 4j). One possible explanation for this apparent contradiction is that the alteration of RCD levels is influenced by the immune microenvironment, which shapes the immune response and impacts tumor development and progression.

### Direct positive association of RCD levels with the effectiveness of tumor ICI

**Fig. 5:**
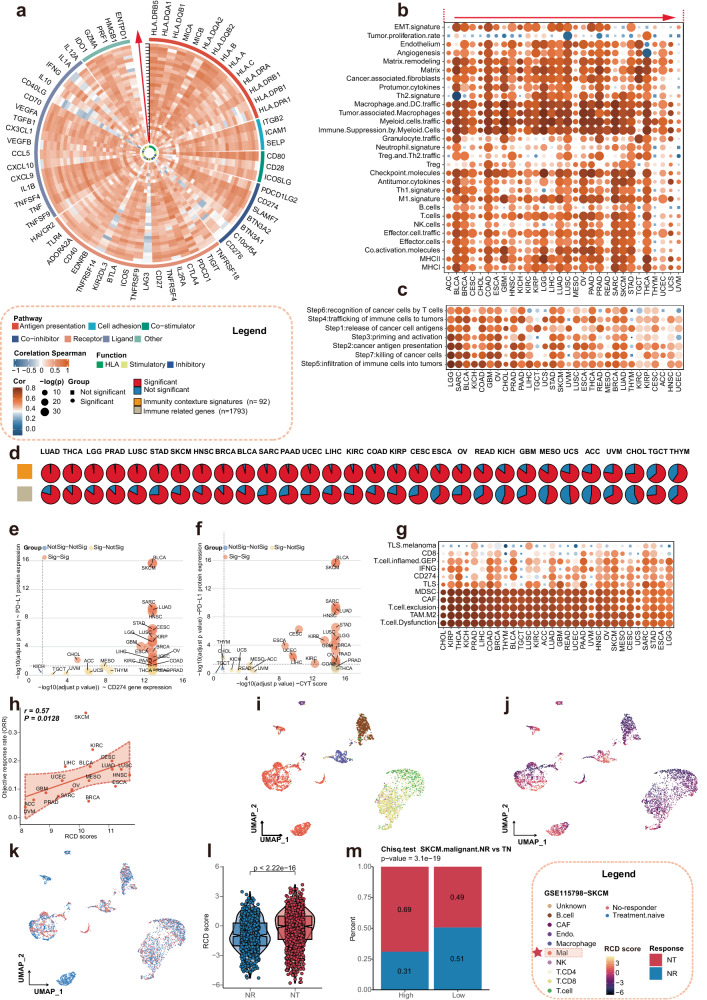
Identification of a positive association between the RCD score and immunotherapy response. **a** The circle heat map showing the association between the RCD score and the gene expression of the immune checkpoints in individual cancer type, with Spearman correlation. From inside to outside of the circle heat map, the vertical axis with a black arrow indicated the different cancer types, which were annotated in by the *x* axis of plot B. **b** The heat map depicting the Spearman correlation between the RCD score and the ssGSEA score of 29 microenvironment signatures across multiple cancers. **c** The heat map indicating the correlation between the RCD score and the activity of the 7-step antitumor immune cycle signals across multiple cancer types. **d** The upper panel showing the proportions of signatures significantly correlated with the RCD score in 92 immune contexture signatures. The bottom panel showing the proportions of genes significantly correlated with the RCD score in 1793 immune-related genes. The cancer types were ordered by increasing proportions of RCD score-related signatures in 92 immune microenvironment signatures. **e** Significant of the Spearman correlations of RCD score with gene expression of CD274 (*x* axis) and PD-L1 protein expression (*y* axis) at pan-caner level. The orange indicated that both two *p* values were <0.05. The yellow indicated that the CD274-related *p* values were <0.05, while the PD-L1-related *p* values were not <0.05. And the blue indicated that both the two kinds of *p* values were not <0.05. **f** Significant of the Spearman correlations of RCD score with CYT score (*x* axis) and PD-L1 protein expression (*y* axis) at pan-caner level. **g** The heat map depicting the Spearman correlation between the RCD score and ssGSEA scores of the immunotherapy response signatures. **h** Spearman correlation of median RCD score and the objective response rate of each cancer types. **i** UMAP plot of the identified cell types in GSE115798 (SKCM). Different colors represented the different cell types. **j** UMAP plot of the identified cells colored by the RCD score. The RCD score was calculated as described in methods. **k** UMAP plot of the identified cells colored by the immunotherapy response. **l** The difference in RCD score between the NR and NT in SKCM cohort. The center of the box pot was the median values, the bounds of the box were the 25% and 75% quantiles. Wilcoxon rank sum test was used for estimating the difference. NR non-responder, NT naive treatment. **m** The difference in the proportion of the NT and NR between in high RCD subgroup and the low RCD subgroup. The chi-square statistic test was used and the cells were divided into a high RCD subgroup and a low RCD subgroup based on the median of the RCD score.

The expression of immune checkpoint molecules on tumor cells and immune cells in the TME can influence the response to ICI. Targeting these checkpoints with ICI can release the brakes on the immune system and enhance antitumor immune responses^8^. In this study, we aimed to investigate the association between RCD levels and the expression of immune checkpoint in each cancer type. Our analysis revealed a significantly positive correlation between almost all checkpoint genes and RCD levels across different cancers (Fig. 5a).

Furthermore, we examined the activity of 29 signatures, categorized into four groups: anti-TME, pro-TME, angiogenesis fibrosis, and malignant cell properties. The results demonstrated a significant positive relationship between these signatures and RCD levels across most cancers (Fig. 5b). Notably, the approaches to enhance the efficacy of tumor ICI therapy primarily focus on increasing tumor immunogenicity, enhancing antigen presentation, promoting immune activation, and so on^23^. Here, we investigated the relationship between the seven steps in the antitumor immune cycle and the RCD level in individual cancer types (Fig. 5c). The analysis suggested a significant positive association between RCD levels and all steps of the antitumor cycle across most cancers. Moreover, when considering 1793 immune-related genes, we observed that the majority of the genes were significantly associated with the RCD levels in almost all solid cancers. A similar trend was observed for the 92 immunity contexture signatures (Fig. 5d).

To foster a comprehensive understanding, we integrated the CD274 gene expression and PD-L1 protein expression, as well as the CYT score and PD-L1 protein expression. The analysis revealed a significant positive relationship between RCD levels and these indicators in several cancer types, such as LGG and GBM (Fig. 5e, f). Additionally, we collected some signatures associated with immune response and investigated their association with RCD levels in individual cancer types (Fig. 5g). Across most cancer types, a significant positive correlation was observed between these signatures and RCD levels.

Objective response rate (ORR), a metric used in clinical trials and cancer research to measure the effectiveness of a particular therapy in inducing tumor shrinkage, was analyzed in relation to RCD levels. Our findings indicated a significant positive association between RCD levels and ORR (Fig. 5h). Furthermore, consistent with previous observation, RCD levels showed a positive correlation with TMB, higher TMB is often associated with increased neo-antigen formation, rendering the tumor more recognizable to the immune system^24^. However, a seemingly controversial result emerged, indicating a positive correlation between RCD levels and ITH. ITH can influence the response to ICI, and a higher level of ITH can complicate the ICI response and hinder consistent and durable outcomes^25^.

To further elucidate the relationship between antitumor-infiltrating immune cells, RCD levels, and ITH, we divided patients into four distinct subgroups based on median RCD score and median ITH score. The subgroups include high RCD-high ITH (HRHI), high RCD-low ITH (HRLI), low RCD-high ITH (LRHI), and low RCD-low ITH (LRLI). The analysis of immune cell infiltration estimated by the MCP counter revealed higher levels of infiltration in the high RCD subgroup compared to the low RCD subgroup (Supplementary fig. 3C). Similarly, in the comparison between high ITH and low ITH subgroups, seven out of ten immune cell types exhibited higher infiltration in the high ITH subgroup (Supplementary fig. 3D). Furthermore, the comparison of immune cell types among the four subgroups showed that tumors with high RCD levels displayed significantly better antitumor immunity, irrespective of ITH levels (Supplementary fig. 3E).

Additionally, we evaluated the relationship between RCD levels and ICI outcomes, with the scRNA-Seq data from the GSE115798 (Melanoma, SKCM). Considering the absence of responders in this dataset and the inclusion of treatment-naïve patients who may include potential responders, we analyzed 24 patients, including 11 non-responders (NR) and 13 patients with naïve treatment (NT), after excluding patients with no malignant cells. The cells with high RCD scores were mainly malignant cells and were enriched in the NR subgroup (Fig. 5i–k). The RCD score in the NR subgroup was significantly lower than that in the NT subgroup (Wilcoxon rank sum test, *p* < 0.001, Fig. 5l). In the high RCD subgroup, the proportion of cells from the NR subgroup was higher compared to the low RCD subgroup (Chi-square test, *p* < 0.001, Fig. 5m). These results were further validated using scRNA-Seq data from the GSE123813 dataset, which consisted of a BCC cohort including 4 NRs and 6 responders (Wilcoxon rank sum test, *p* = 0.067, supplementary Fig. 4A–D).

Collectively, our results provide compelling evidence for a highly positive correlation between RCD levels and the effectiveness of tumor ICI therapy. Specifically, higher RCD levels are associated with an increased likelihood of ICI efficacy.

### Development and comprehensive description of the RCD.Sig

**Fig. 6:**
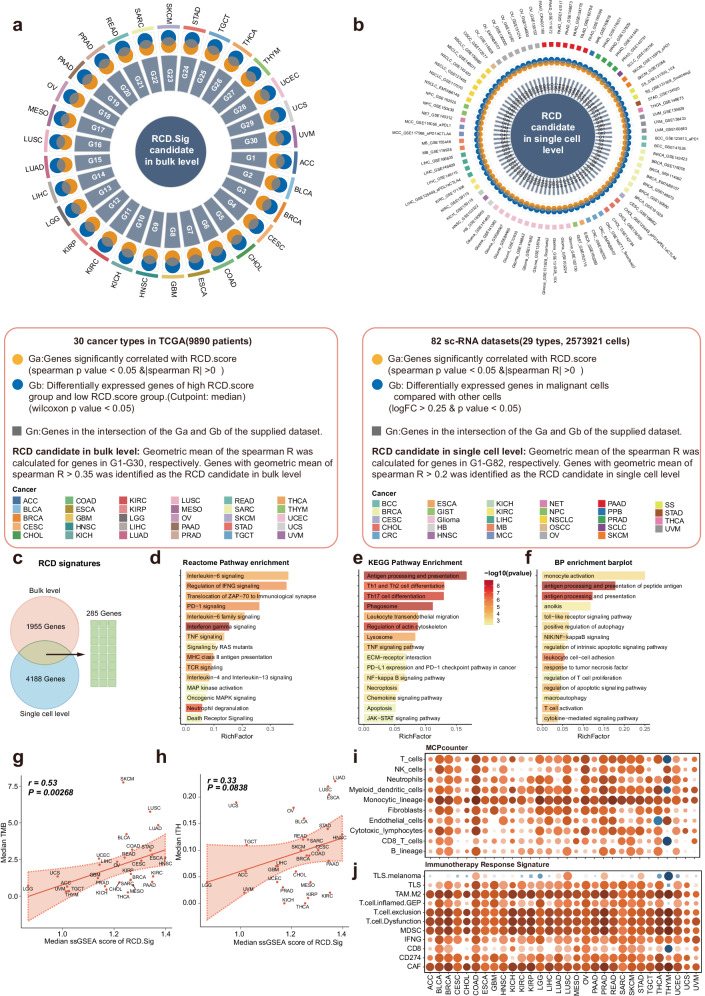
Identification and description of the RCD signature. **a** Circos plot showing the development of RCD.Sig in bulk level. **b** Circos plot showing the development of RCD.Sig in single-cell level. **c** 285 genes were identified as the RCD.Sig by intersecting the 1955 candidate genes in bulk level and 4186 candidate genes at single-cell level. **d** The bar plot depicting the enriched Reactome pathways of the RCD.Sig. **e** The bar plot depicting the enriched KEGG pathways of RCD.Sig. **f** The bar plot depicting the enriched BP pathways of RCD.Sig. BP: biological process. **g** Spearman correlation of median RCD.Sig score and median TMB of each cancer type. The ssGSEA scores were calculated to estimate the activity level of the RCD.Sig for each sample in TCGA. **h** Spearman correlation of median RCD.Sig score and median ITH of each cancer type. **i** The heat map showing the Spearman correlation of the RCD.Sig score and the immune cell infiltration estimated by the MCP-counter algorithm across the cancer types. **j** The heat map depicting the Spearman correlation of the RCD.Sig score and the activity of the immunotherapy response signatures across the cancer types.

We hypothesized that a simplified RCD.Sig, used to estimate the level of RCD in tumors, could enhance the effectiveness of ICI prediction due to its significant correlation with immunity and ICI response. Our four-step framework, described in detail in the methods section, resulted in 1955 and 4188 candidate genes, respectively (Fig. 6a, b). The intersection of these gene sets yielded the RCD.Sig, comprising 285 genes (Fig. 6c).

To investigate the functional significance of the 285 genes in RCD.Sig, we conducted functional pathway enrichment analysis using REACTOME, KEGG, and BP data from GO terms. These genes exhibited enrichment in pathways related to RCD, such as death receptor signaling, apoptosis, and anoikis. Additionally, they were enriched in multiple pathways associated with immunity, including interleukin-6 signaling, PD-1 signaling, and T-cell activation (Fig. 6d–f).

We found that the ssGSEA score of RCD.Sig positively correlated with both TMB and ITH, while showing no association with CNV (Fig. 6g, h, supplementary fig. 5A). Stemness-related properties, such as enhanced DNA repair mechanisms and resistance to apoptosis, have been implicated in resistance to ICI. To explore the association of RCD.Sig with stemness, we investigated their correlation using 26 stemness-related signatures (Supplementary fig. 6A–C). The analysis provided partial evidence of a positive association between RCD level and stemness, with 19 out of 26 signatures demonstrating a positive correlation (Supplementary fig. 6B).

Furthermore, we evaluated the association of RCD.Sig with immunity (Fig. 6i, j, supplementary fig. 5B–E). These results exhibited a remarkable resemblance to the analysis of the RCD score, suggesting that RCD.Sig can be used to assess the RCD level.

In summary, we successfully developed a simplified RCD.Sig to evaluate the RCD level, which can facilitate the construction of further models for improving the prediction of ICI efficacy.

### Construction and comparable assessment of the predictive model for ICI response based on the RCD.Sig

**Fig. 7:**
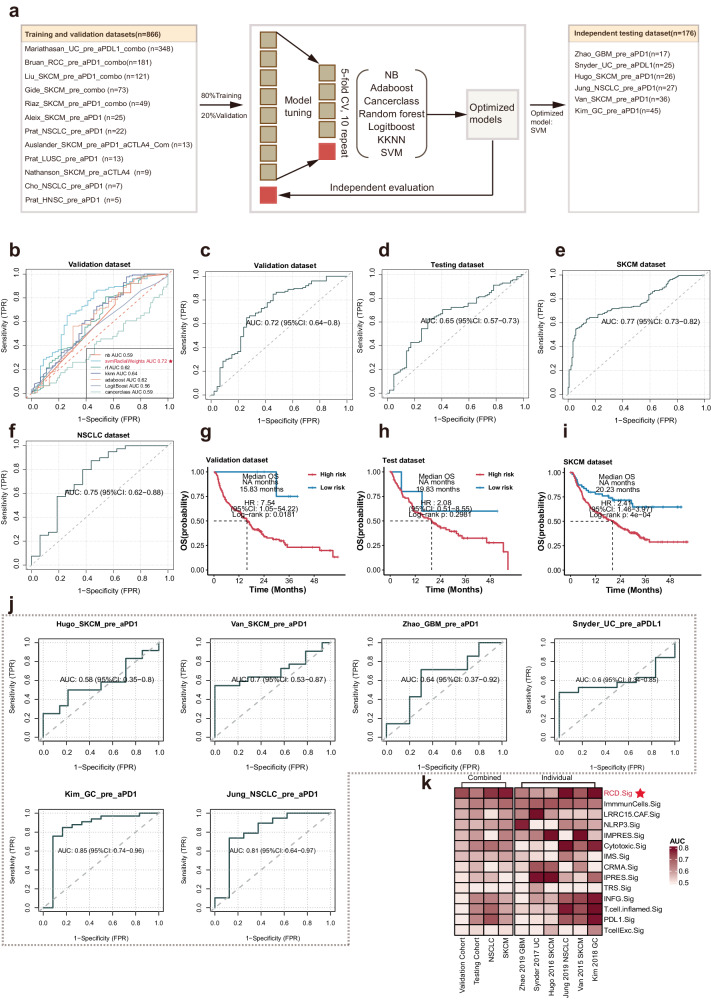
Development and evaluation of the immunotherapy response predictive model. **a** The workflow of development of the model based on the RCD.Sig with 7 machine-learning algorithms. The basic steps including the training, validation and testing of the model. In the training set, 80% of 866 samples from 12 cohorts were used as the training dataset to perform the model tuning. In the validation set, the SVM algorithm with the highest algorithm was considered the optimal RCD.Sig model for immunotherapy response. And in the test dataset including 176 samples from 6 independent cohorts, we tested the performance of the final model. **b** Comparison of the AUC of the multiple model developed by 7 used machine-learning algorithms in validation dataset. The SVM algorithm, the AUC of which was 0.72, was considered the optimal model. **c**–**f** ROC plot showing the performance of the optimal RCD.Sig model in validation dataset, testing dataset, SKCM dataset, and NSCLC dataset, in which the AUC of the final RCD.Sig model was 0.72, 0.65, 0.77, and 0.75, respectively. **g**–**i** K–M curves show the difference in the overall survival between the patients with high risk and patients with low risk. The patients with high risk and the patients with low risk were defined as the patients with “NR” and “R” predicted by the optimal RCD.Sig model. **j** ROC plot showing the performance of the optimal RCD.Sig model in individual testing dataset, the AUC in which was range from 0.58 to 0.85. **k** The heat map comparing the performance of the optimal RCD.Sig with other 13 immunotherapy response models in multiple cohorts. All the models were ordered by the AUC in validation dataset.

Given the dramatic correlation between the RCD level and the ICI response, we aimed to explore the potentially predictive value of the RCD.Sig for ICI. We collected 18 bulk-level transcriptomic cohorts treated with ICI, excluding samples without pre-treatment. As mentioned previously in the methods section, we trained models using seven machine-learning algorithms, obtaining seven trained models. We subsequently calculated and compared the area under the curve (AUC) of these models in the validation dataset (Fig. 7a). The AUC ranged from 0.56 (logitBoost) to 0.72 (SVM) (Fig. 7b). The model trained with the support vector machine (SVM) algorithm, with the highest AUC, was identified as the predictive model for ICI response (Fig. 7c).

To evaluate the performance of the optimal model, we tested it in the testing, SKCM, and non-small cell lung cancer (NSCLC) datasets, with AUC values of 0.65, 0.77, and 0.75, respectively (Fig. 7d–f). To explore the performance of the predictive model for OS in cohorts treated with ICI, we performed a log-rank test after dividing the patients into high-risk and low-risk groups based on the prediction results of the optimal model. The patients in the high-risk group had worse OS than those in the low-risk group in the validation dataset (HR: 7.54, p: 0.0181), testing dataset (HR: 2.08, *p*: 0.2981), and SKCM dataset (HR: 2.41, *p*: 0.00041) (Fig. 7g–i).

Furthermore, we evaluated the robustness of the predictive model using individual testing cohorts, with the AUC ranging from 0.58 to 0.85 (Fig. 7j). We compared the performance of the optimally predictive model with 13 published signatures, indicating that the RCD.Sig-based model exhibited extraordinary superiority and consistently high predictive effectiveness across most cohorts (Fig. 7k, supplementary table 2).

Overall, the predictive model for ICI response based on RCD.Sig demonstrated remarkable robustness and superiority compared to previously published signatures across various cancer types.

### Using CRISPR screening data to determine candidate therapeutically relevant targets from RCD.Sig

To identify potentially important targets, we analyzed 17 datasets derived from the collected 7 CRISPR cohorts. These datasets evaluated the effects of knocking out 22,505 genes on antitumor immunity. Additionally, we gathered 989 CRISPR cell lines with CRISPR essentiality revealed by large-scale knockout screens (CERES) scores for 17,645 genes for analysis (Supplementary Fig. 7A–F). Among these datasets, the top 10% of genes in the CRISPR cell line data and the immune response CRISPR datasets included 29 genes and 41 RCD.Sig genes, respectively (Supplementary Fig. 7C, D). Notably, five genes (WDR83, TLN1, MCL1, ACTR3, and ACTR2) were present in both the 29 genes and the 41 genes (Supplementary fig. 7E).

Then, we compared the expression of these genes, except MCL1, between the NR and the NT group in the GSE 115978 dataset. The percentage of cells expressing these genes was significantly lower in the NT group (Chi-square test *p* < 0.001, Supplementary fig. 7F). Additionally, we assessed the potential value of these five individual genes in OS and the ICI response using a combined cohort (Supplementary Fig. 8A, B).

In summary, we identified five hub genes from RCD.Sig using CRISPR screening data. Knocking out these genes impaired tumor cell fitness and enhanced antitumor immunity.

### Development and validation of the predictive model for OS based on the RCD.Sig

**Fig. 8:**
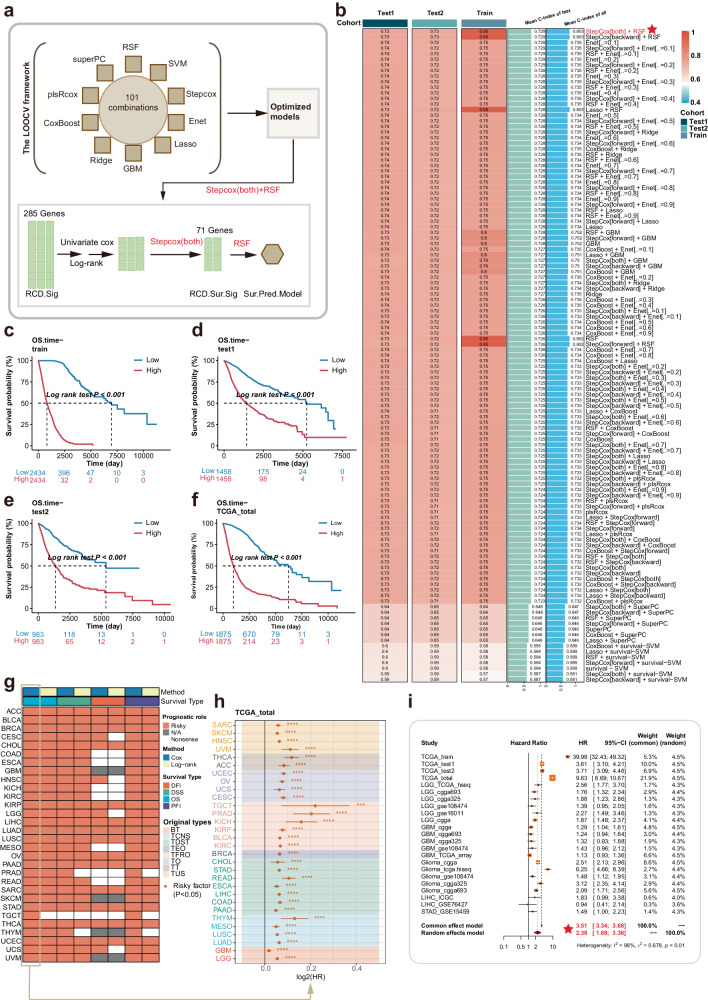
Development and evaluation of the overall survival predictive model. **a** The workflow of development of the overall survival predictive model based on the RCD.Sig with 101 combinations of 10 machine-learning algorithms. The details were summarized in methods. **b** The left panel was a heat map, showing the C-index of 101 combinations in training dataset, testing dataset 1 and testing dataset 2. The right panel was two bar plots, showing the mean C-index of the testing datasets and the mean C-index of the three datasets. The stepcox [“both”]+RSF with the highest mean C-index was considered as the final RCD.Sig survival predictive model. **c**–**f** K–M curves comparing the overall survival between the patients with high risk and the patients with low risk in training. dataset, testing dataset 1, testing dataset 2, and TCGA, which was an amalgam of training dataset, testing dataset 1, testing dataset 2. The risk score was calculated by the optimal survival predictive model. The patients in the cohort were divided into high-risk group and low-risk group based on the median of the risk score. **g** The correlation of the risk score computed by the optimal survival predictive model and the clinical outcomes, including OS, DFI, DSS, and PFI, in TCGA, using the univariate Cox regression analysis and the log-rank test. The *p* value < 0.05 and HR > 1 was considered as risky. The *p* value ≥ 0.05 was considered as nonsense. “N/A” indicated that the corresponding data was missing. **h** The forest plot showing the univariate Cox regression result of the risk score in all TCGA dataset. TCNS tumors of central nervous system, TT thoracic tumors, TDST tumors of digestive system, BT breast tumor, TUS tumors of urinary system and male genital organs, TFRO tumors of female reproductive organs, TEO tumors of endocrine organs, TO tumors of others. **I** Meta-analysis of the comprehensive prognostic performance of the risk score in these 23 cohorts.

Given the significant association of the RCD level with clinical outcomes, particularly OS, we developed a simplified signature named RCD.Sur.Sig. This signature was based on the RCD.Sig genes and aimed to accurately predict the prognosis at the pan-cancer level. The construction of RCD.Sur.Sig involved the leave-one-out cross-validation (LOOCV) framework, which comprised 101 combinations of ten machine-learning algorithms, as previously described in the methods section (Fig. 8a). Within the LOOCV framework, we optimized the combination of stepwise Cox regression (both) and RSF based on the highest mean C-index to construct the model (Fig. 8b, supplementary table 3).

Following stepwise Cox regression analysis, we selected 71 genes as the RCD.Sur.Sig, which were then utilized to develop the predictive model using the RSF algorithm. In all datasets, patients with a high-risk score exhibited significantly worse OS compared to those with a low-risk score (Log-rank test *p* value < 0.001, Fig. 8c–f). Additionally, we assessed the predictive performance of the model for clinical outcomes in individual cancer types (Fig. 8g, h, supplementary Fig. 9A–C, supplementary Table 4). In the TCGA total dataset, the model demonstrated powerful and robust prediction effectiveness across cancer types (Supplementary Fig. 10, 11A). Notably, the risk score calculated by the model was a significant risk factor for OS, in 29 out of 30 cancer types (Supplementary fig. 11B). Furthermore, we tested the robustness of the model using external independent datasets such as CGGA, GSE15459, and GSE76427. These tests confirmed that the model exhibited stable performance, indicating its ability to handle different conditions without significant degradation in accuracy or performance metrics (Supplementary Fig. 11C–E, 12A, B). By conducting a meta-analysis of 23 cohorts, we calculated the comprehensive combined HR, which demonstrated that the risk score was a significantly risky factor for OS (comprehensive combined HR: 3.51, *p* value < 0.001, Fig. 8i, supplementary table 5). To identify the most valuable targets for OS prognosis and facilitate targeted drug development or clinical application, we selected core genes from RCD.Sig using a seven machine-learning framework at the pan-cancer level, specifically in TCGA-LGG and TCGA-GBM (Supplementary Fig. 13A–D). Some genes, such as SLC43A3 and FOSL1, were identified as potential targets (Supplementary Fig. 13E, F).

Collectively, we obtained a 71-gene RCD.Sur.Sig, and developed a powerful and robust model for predicting OS. This model consistently performed well across different scenarios and datasets. Moreover, we identified some core prognostic genes, such as SLC43A3 and FOSL1.

## Discussion

RCD refers to a complex series of processes by which cells in an organism undergo self-destruction in a controlled manner^1^. It plays a crucial role in various biological processes, such as development, tissue homeostasis, and immune response. There are several types of RCD, including apoptosis, necrosis, autophagy, and pyroptosis. However, a comprehensive assessment of the status and associated features of RCD at the pan-cancer level remains unexplored^1^. In this study, we explored the potential association between RCD and immunotherapeutic response and observed a significant correlation between RCD and ICI outcomes. Notably, RCD was found to occur in almost all solid tumors, and a positive correlation between RCD and enhanced antitumor immunity was observed in several cancer types. Motivated by these findings, we hypothesized that a positive correlation between RCD and ICI effectiveness is prevalent across a wide range of cancers. To test this hypothesis, we performed a comprehensive integrative analysis to identify genes significantly associated with RCD in malignant cells. This analysis yielded a pan-cancer RCD signature, referred to as RCD.Sig, which comprised genes that demonstrated a strong correlation with RCD. We conducted meticulous investigations to validate the predictive power of RCD.Sig, and remarkably, it outperformed previous predictive signatures in multiple cohorts, predicting response to ICI based on RNA-Seq data from independent ICI treatments. We also investigated the RCD score with the spatial transcriptomic data of glioblastoma, indicating that RCD were heterogeneous in terms of spatial location (Supplementary Fig. 14).

Additionally, we examined the connection between RCD and the clinical prognosis of oncology patients. In order to better guide precise treatment decisions and predict prognosis across different cancer types, we identified genes highly correlated with patient prognosis at the pan-cancer level using RCD.Sig. This analysis resulted in the development of a pan-cancer RCD survival signature, named RCD.Sur.Sig, which showed significant predictive value. Of note is that RCD.Sur.Sig performed exceptionally well in predicting the prognosis of oncology patients across multiple independent datasets. To the best of our knowledge, this study represents the first extensive evaluation of RCD levels at a cancer-wide level. We achieved this by integrating 18 RCD types and multiple RCD-associated genes.

This multi-parameter index takes advantage of the shared characteristics among different cancer cell types, surmounting the limitation of lacking RCD-specific biomarkers. It provides a standardized measure for comparing RCD levels across different cancer types. Interestingly, we observed that RCD scores were lower in adjacent normal tissue and increased with clinical parameters associated with aggressive phenotypes. These positive correlations with tumor malignancy and aggressiveness suggest a malignant nature of RCD resistance in tumors, which aligns with the well-documented phenomenon of RCD resistance observed in a wide range of malignancies^26^. Furthermore, we identified heterogeneity in RCD scores across different tissue types, immune cell types, and tumor types, among others. By employing mathematical metrics to quantify RCD levels, our findings provide a valuable framework for gaining further insights into the regulation of RCD within the TME. This framework can guide further experimentation and identification of potential biomarkers.

Regarding the enrichment analysis of RCD.Sig genes, we found significant associations with various biological functions such as TNF signaling, RAS mutation signaling, etc. The TNF signaling pathway plays a dual role in regulating cell death, promoting apoptosis under certain conditions while inducing necrosis when the apoptotic pathway is blocked. RAS mutations are known to have a significant impact on cellular signaling pathways, including those involved in regulating cell death processes like apoptosis, autophagy, and senescence^27^. These molecular changes contribute to the development and progression of cancer by influencing cell fate decisions, and promoting cell survival and proliferation. RCD as well as other regulated cell fates (e.g., senescence), are closely linked to stemness^28^. These mechanisms play a role in stem cell maintenance, differentiation, and fate determination, and ultimately influence tissue development, homeostasis, and disease progression^28^. The interplay between RCD and stemness is complex and stringent, ensuring a balance between proper tissue function and self-renewal and differentiation of stem cell populations. RCD.Sig exhibited a positive correlation with most of the stemness features in different cancer types. These results suggest that RCD.Sig encompasses genes strongly and specifically associated with cancer stemness. RCD.Sig serves as a concise representation of RCD levels across multiple dimensions and demonstrates a positive correlation with increased antitumor immunity and ICI response, similar to the RCD score. As a novel biomarker, RCD.Sig was compared to previously published ICI predictive signatures, and our results demonstrated the significant superiority of RCD.Sig in predicting ICI response and differentiating patients with varying survival outcomes across different cohorts.

Similar to Miranda et al.’s findings, we discovered a positive correlation between RCD. Sig and stemness, TMB and ITH. It is noteworthy that high TMB is linked with high RCD^29^. Despite TMB being a well-recognized biomarker for ICI, many patients with high TMB are unresponsive to ICI^30^. Our findings suggest that RCD could serve as a valid rationale for the immune resistance of high TMB tumors. Our analysis revealed a strong positive correlation between the RCD score and antitumor immunity in both low and high ITH tumors. This provides a possible explanation for the immune resistance observed in high ITH tumors. However, we found no significant correlation between the CNV and RCD scores in pan-cancer studies. This highlights the importance of investigating the impact of CNV in the context of specific tumor tissues. This point is consistent with previous research that demonstrates a significant relationship between CNV and ICI in many types of cancer, but it should be noted that the direction and intensity of this association may not be universal across all cancer types with regard to tumor immunity^31–33^.

Furthermore, this study revealed a negative correlation between RCD and MYC-related genes, DNA repair, and the cell cycle pathway, and a positive association with stemness, inflammation, and metastasis. It is important to note that cells experiencing irreparable damage during the cell cycle may pause at checkpoints for DNA repair. However, if the damage remains unaddressed, RCD can be triggered to eliminate these compromised cells^34^. The oncogene MYC has been shown to regulate cell proliferation and apoptosis. Overexpression of MYC suppresses RCD, such as apoptosis, and promotes cell proliferation, which contributes to cancer development^35^. Conversely, decreased MYC levels can trigger RCD. In terms of the positive correlation between RCD and inflammation, inflammation can induce RCD in cells as a protective mechanism. On the other hand, sustained inflammation may disrupt RCD, promoting cell survival and potentially creating a pro-tumorigenic microenvironment^36^. Emerging evidence indicates a bidirectional association between RCD and stemness^37^. RCD regulates stem cell populations by removing damaged cells or maintaining homeostasis. However, stem cells can withstand RCD, promoting their survival and contributing to tumor initiation and progression. The interplay of apoptosis and metastasis is complex^38^. Reduced apoptosis may assist the survival of migrating cancer cells during metastasis^38^. However, in some cases, increased apoptosis may promote the engraftment and spread of metastatic cells to distant organs^38^. These correlations emphasize the intricate involvement of RCD in influencing stemness, inflammation, and metastasis within the complex context of cancer biology.

Our study analyzed the intricate correlation between RCD and immunity, encompassing immune cell infiltration, immune checkpoint genes, and immune features. We provide potential evidence that RCD may serve as a biomarker for predicting ICI response. We found a significant, positive relationship between RCD score and M1 macrophage in nearly all solid tumors, while naive CD4 T cells exhibited the opposite effect. M1 macrophages are significant in their contribution to antitumor immunity and the inhibition of cancer progression, through activities such as tumor suppression, initiation of immune responses, tissue remodeling, antigen presentation, and promotion of inflammation^39^. There are conflicting views on the topic. Furthermore, naive CD4 T cells may also play a crucial role in triggering and regulating the immune response against cancer, by orchestrating various immune mechanisms to identify, respond to, and potentially eradicate cancer cells^40^. In reality, the immune microenvironment of tumors is a delicate interplay between antitumor and pro-tumor factors. The intricate relationship between RCD and the infiltration of two distinct antitumor immune cells demonstrates the complexity of the TME. We discovered that almost all immune checkpoint genes were favorably associated with RCD in almost all solid tumors. This indicates the TME employs immune checkpoint molecules to evade immune surveillance and mitigate immune responses against the tumor, leading to a poor prognosis^41^. The K-M curves with RCD scores across tumors reinforced this.

The significance of biomarker research lies in its potential to improve our understanding of diseases, enable early detection and facilitate personalized medicine, in particular by enabling the development of combinatorial strategies to overcome immune resistance. Given the strong correlation between RCD.Sig and ICI outcomes, as well as the link between RCD and tumor cell death, we utilized CRISPR datasets of cell lines and immune response cohorts to explore potential drug targets for RCD.Sig. We identified the potential therapeutic biomarkers including MCL1, TLN1, ACTR2, ACTR3, and WDR83. MCL1, one of the highest-ranking RCD.Sig genes in both datasets, are essential for cell growth, survival, and proliferation, and play a role in the tumor immune microenvironment^42–44^. MCL1’s regulatory role in cell survival extends to immune cell populations, influencing their viability, activation, and functional outcomes^44^. Overexpression of MCL1 has been associated with resistance to T cell-mediated cytotoxicity in various cancer cell types and mouse xenografts, through regulation of the mitochondrial apoptotic pathway and activation of the NF-κB pathway^45^. Further research on these five genes will contribute to the development of ICI therapy.

We acknowledge certain limitations in our study. First, we defined the RCD score based only on the summation of the ssGSEA score at the bulk level and GSVA score at the single-cell level of the 18 signatures. Whether there is a more optimal algorithm to describe the overall level of RCD in the real world warrants further discussion and debate. Although GSVA demonstrates superiority in UMI-based single-cell data compared to other enrichment algorithms, the presence of dropout events may affect the accuracy of RCD score calculations. Second, additional external independent datasets are required to validate the two prediction models to enhance the reliability and generalizability of the study. Third, an investigation into the biological mechanisms underlying the relationship between RCD levels and tumor ICI therapy is required. Incorporating experimental validation and functional analysis of key genes involved in immune evasion and treatment resistance should be performed to provide deeper insights into the processes at play. Fourth, while this study identified several potential targets with practical clinical applications based on RCD levels using various strategies, specific experiments to validate them were not conducted. Validation experiments will be our next step in future research, and more wet-lab work needs to be done to identify the 12 signaled core genes, especially experiments related to GBM. Fifth, we used the AUC as the metric for analyzing the data from 18 immunotherapy cohorts that are imbalanced (296 (28%) responder, 763 (72%) non-responder), as the previous study had done^46^. When using the AUC as an evaluation metric, it is essential to be aware that it can be overly optimistic in the context of an imbalanced dataset. Therefore, it might be more appropriate to consider using the Matthews Correlation Coefficient (MCC) as the evaluation metric for handling an imbalanced dataset. Finally, in this study, we provided evidence for a positive correlation between RCD scores and the effectiveness of ICI, but the relationship between the RCD scores and immune response was not comprehensively explored, which should be further investigated.

## Methods

### Gathering and preprocessing the RCD signatures

We compiled a comprehensive collection of 18 signatures of RCD by utilizing various databases and published literature^1,47–54^. The specific strategies employed for collecting these signatures are summarized in supplementary table 6. To ensure a more comprehensive and accurate representation of RCD signals, we processed signatures from different sources by merging sets for each type of RCD. The total number of signatures for each RCD type was as follows: alkaliptosis (*n* = 7), anoikis (*n* = 29), apoptosis (*n* = 642), autosis (*n* = 1826), cuproptosis (*n* = 72), disulfidptosis (*n* = 16), entotic cell death (*n* = 15), ferroptosis (*n* = 788), immunogenic cell death (*n* = 3449), lysosomal cell death (*n* = 295), mitotic cell death (*n* = 1695), mitochondrial permeability transition (MPT)-driven necrosis (*n* = 204), necroptosis (*n* = 315), netotic cell death (*n* = 8), oxeiptosis (*n* = 5), parthanatos (*n* = 309) and pyroptosis (*n* = 624). The detailed results were provided on the Github (https://github.com/zwxiangya/RCDscore).

### Pan-cancer bulk-sequencing transcriptomic datasets and preparation

The multi-omics, including transcriptomic data, CNV data, and matched clinical medical information, from The Cancer Genome Atlas (TCGA) and Genotype-Tissue Expression(GTEx)^55^ were obtained from UCSC XENA databank (https://xenabrowser.net/datapages/)^56^. The RNA-seq data transformed by log(*x* + 1), somatic mutation data, and copy number variation data calculated via the GISTIC2 method were generated from the illumine platform, illumine platform, and Hiseq illumine platform, respectively. Samples from patients without survival time or survival status, as well as samples from normal patients, were excluded. Only solid cancer types were included, while patients with acute myeloid leukemia (LAML), pheochromocytoma and paraganglioma (PCPG), and lymphoid neoplasm diffuse large B-cell lymphoma (DLBC) were deleted. Eventually, 9890 tumor samples from 30 solid cancers with multi-omics data were obtained for further analysis.

The tumor mutation burden (TMB) data and the intra-tumor heterogeneity (ITH) data of the TCGA samples were downloaded from the cBioPortal website (https://www.cbioportal.org), and the corresponding literature^57^. Additionally, the transcriptome and the clinical information of CGGA-325 (*n* = 325) and CGGA-693 (*n* = 693), including patients with low-grade glioma and glioblastoma, were downloaded from the Chinese Glioma Genome Altas (http://www.cgga.org.cn/)^58^. Patients without overall survival (OS) data were excluded. RNA-seq data and clinical information (*n* = 232) of LIHC samples were obtained from the ICGC-LIRI-JP cohort (https://www.icgc.gov/). Transcriptomic data and survival information of GSE16011 (Glioma, *n* = 117)^59^, GSE108474 (Glioma, *n* = 247)^60^, GSE76427 (Liver hepatocellular carcinoma, *n* = 115)^61^ and GSE15459 (Stomach adenocarcinoma, *n* = 192)^62^ were gathered from Gene Expression Omnibus (GEO), excluding patients without OS. The objective response rate (ORR) data for anti-PD-1/PD-L1 therapy of 21 cancer types in TCGA were gathered from Joo Sang Lee et al. (Supplementary Table 7)^63^. Data for tumors with an ORR value of 0 were removed. Specifically, data for both COAD_MSS and PAAD tumors were excluded from the analysis process with ORR data.

### Pan-cancer single-cell RNA sequencing datasets and preparation

To identify the RCD signature (RCD.Sig), we obtained 82 scRNA-sequencing datasets, including malignant cells and other cell types from the TISCH website (http://tisch.comp-genomics.org/)^64^. The 82 single-cell sequencing dataset comprised 840 patients with a total of 2,573,921 cells. It encompassed 29 cancer types including basal cell carcinoma (BCC), breast invasive carcinoma (BRCA), cholangiocarcinoma (CHOL), colorectal cancer (CRC), glioblastoma multiforme (Glioma), head and neck squamous cell carcinoma (HNSC), liver hepatocellular carcinoma (LIHC), medulloblastoma (MB), merkel cell carcinoma (MCC), neuroendocrine tumor (NET), non-small cell lung cancer (NSCLC), ovarian serous cystadenocarcinoma (OV), pancreatic adenocarcinoma (PAAD), skin cutaneous melanoma (SKCM), stomach adenocarcinoma (STAD), uveal melanoma (UVM), cervical squamous cell carcinoma and endocervical adenocarcinoma (CESC), esophageal carcinoma (ESCA), hepatoblastoma (HB), nasopharyngeal carcinoma (NPC), oral squamous cell carcinoma (OSCC), kidney chromophobe (KICH), kidney renal clear cell carcinoma (KIRC), prostate adenocarcinoma (PRAD), synovial sarcoma (SS), gastrointestinal stromal tumor (GIST), pleuropulmonary blastoma (PPB), small cell lung cancer (SCLC) and thyroid cancer (THCA) (Supplementary table 8).

### Collection and procession of cohorts with ICI

To develop and rigorously evaluate a robust RCD.Sig-based classifier for predicting the response to ICI, we conducted a comprehensive and systematic collection of 18 cohort datasets comprising pre-treatment samples treated with immune checkpoint inhibitors. These datasets encompassed transcriptomic data along with corresponding clinical information, sourced from published studies and publicly available resources^10,22,65–77^. The 18 cohorts included a total of 1,059 patients (296 responders, 763 non-responders) with 8 cancer types, including glioblastoma (GBM, *n* = 1), head and neck squamous cell carcinoma (HNSC, *n* = 1), lung squamous cell carcinoma (LUSC, *n* = 1), melanoma (*n* = 8), non-small cell lung cancer (NSCLC, *n* = 3), renal cell carcinoma (RCC, *n* = 1), gastric adenocarcinoma (GC, *n* = 1), urothelial carcinoma (UC, *n* = 2). For cohorts where the number of samples was greater than the number of patients, we randomly selected one counterpart of each patient to be included in the analysis as the final sample. Concerning the treatment of immune checkpoint inhibitors, there were four approaches; including anti-PD-1 (*n* = 12), anti-PD-L1 (*n* = 2), anti-CTLA-4 (*n* = 2), and combination of ICI (*n* = 2). The batch effects were removed by the Combat algorithm with the R package “sva”. 6 of 18 cohorts, named Zhao_GBM_pre_aPD1 (*n* = 17), Snyder_UC_pre_aPD1 (*n* = 25), Hugo_SKCM_pre_aPD1 (*n* = 26), Jung_NSCLC_pre_aPD1 (*n* = 27), Van_SKCM_pre_aPD1 (*n* = 36) and Kim_GC_pre_aPD1 (*n* = 45), were utilized as the independent testing dataset (*n* = 176). The others (*n* = 866) were randomly split into two datasets, used as the training dataset (80%, *n* = 693) and validation dataset (20%, *n* = 173). Detailed information on these ICI cohorts has been summarized in supplementary table 9.

### Immunotherapy response signatures

To enhance the description of our model developed using RCD.Sig for predicting immunotherapy response, we collected 13 prediction signatures from the existing literature, and compared our model with these existing signatures across multiple dimensions to assess the superior performance of our model^66,78–88^. The 13 signatures included IFNG (*n* = 6), T cell-inflamed GEP (*n* = 18), PD-L1 (PD-1 expression by IHC), LRRC15 + CAF (*n* = 14), NLRP3 inflammasome (*n* = 30), cytotoxic (*n* = 4), immuneCells (*n* = 108), T-cell exclusion (n = 203), CRMA (*n* = 8), IMPRES (*n* = 15, Gene pairs), IPRES (*n* = 16), TRS (*n* = 6) and IMS (*n* = 24). The details of which have been summarized in the supplementary table 10.

### Stemness-related signatures

To further investigate the correlation between RCD.Sig and stemness, a characteristic that has been shown recently to be highly relevant in cancer treatment with ICI, we collected 26 stemness-related gene lists from various sources, using the StemChecker tool (http://stemchecker.sysbiolab.eu/)^89^. The number of genes in these lists varied from 17 to 982. Further details regarding these gene lists can be found in supplementary table 11.

### Purified immune cell line data

Raw transcriptomic data and corresponding annotation information for 115 purified immune cell lines, comprising 16 datasets and encompassing 19 different immune cell types, were obtained from the GEO databank (GSE8059, GSE59237, GSE49910, GSE39889, GSE28726, GSE28490, GSE27291, GSE23371, GSE13906, GSE25320, GSE27838, GSE28698, GSE37750, GSE42058, GSE51540, GSE6863)^90–105^, and preprocessed as described in the original literature^106^. Related information has been summarized in the supplementary table 12.

### Obtaining the tumor cell line data

Transcriptome data and corresponding annotation data of human cancer cell lines were obtained from the Broad Institute-Cancer Cell Line Encyclopedia (https://portals.broadinstitute.org/ccle/)^107^. Additionally, datasets containing the CERES score for 17,645 genes across 989 cell lines were downloaded from the DepMap portal(https://depmap.org/portal/)^108^. The CERES (CRISPR essentiality revealed by large-scale knockout screens) score is a metric used to evaluate the likelihood of a gene being essential for cell survival. It is derived from CRISPR-Cas9 genome-wide screening experiments, where individual genes are systematically knocked out to observe their impact on cell viability and proliferation. Genes with high negative CERES scores are considered more likely to be essential for cell survival, while genes with positive scores are regarded as less critical or even potentially dispensable. By ranking the 17,645 genes based on the mean CERES scores across 989 cell lines, we identified the top 10 genes with low CERES scores as important genes for cell survival. Further details regarding these data can be found in supplementary table 13.

### Immune CRISPR-screening datasets

To identify hub genes within RCD.Sig for further analysis and provide evidence supporting the application of therapeutic targets, we collected a total of 7 CRISPR/Cas9 screening datasets from public literature^109–115^. These datasets were further divided into 17 datasets based on cell type and experimental conditions. The scores obtained from these cohorts were used to gauge the independent effects of genes on tumor immunity. Genes in top rank of the cell lines, the absence of the targeted, which lead to decreased cell viability or growth, were likely essential for cellular survival under the tested conditions, while genes in bottom rank of the cell lines had little impact on cell fitness. Similarly, the genes in top rank of the immunotherapy cohorts, the absence of which would improve antitumor immunity, were genes associated with resistance to the immunotherapy, while the genes in the bottom rank, related with the sensitivity to the immunotherapy, lead to immunosuppression after knockout. Specifically, a lower score for a gene indicates a stronger immune response when that gene is knocked out, while a higher score suggests a weaker immune response upon gene knockout. Similar to the CERES scores, we ranked the 20,000+ genes based on their mean scores across all cohorts. The top 10% of genes with the lowest scores were considered as the core genes for immunotherapy. The survival information and the source of these cohorts were summarized in supplementary tables 14 and 15.

### Collecting the signatures of immunity and functional pathways

To provide a comprehensive and multi-layered description of the association between RCD and immunity, we assembled an integrated collection of immune-related signatures. These signatures include immune-related genes, immune checkpoint genes, etc. We obtained 1,793 immune-related genes from ImmPort (https://www.immport.org/), which were categorized into 17 pathways^116^ (Supplementary Table 16). Additionally, immune checkpoint genes (*n* = 75, supplementary table 17)^117^, immunity contexture signatures (*n* = 92, supplementary table 18)^57^, signatures of cancer single-cell state (*n* = 14, supplementary table 18)^118^, anti-cancer immune cycle signatures (*n* = 7, supplementary table 18)^119^, T cell-inflamed GEP signature^78^, CAF signature^120^, TAM M2 signature^120^, IFNG signature^120^, CD8 signature^120^, CD274 signature^120^, TLS signature^121^, TLS-melanoma signature^121^, T cell dysfunction signature^120^, T cell exclusion signature^120^ and MDSC signature^120^ were collected from the previous and summarized in supplementary table 18.

Moreover, we downloaded HALLMARK signatures and C6 oncogenic signatures from the MsigDB (https://www.gsea-msigdb.org/gsea/msigdb) to investigate the correlation between RCD and tumor-related functional pathways. The average of transcriptomic expression of GZMA and PRF1 was considered as the CYT score. The ITH data of the pan-cancer was summarized in supplementary table 19.

### RCD score calculation

To comprehensively evaluate the level of regulated cell death across tumor and normal tissues or cell lines at a bulk level, we employed single-sample gene set enrichment analysis (ssGSEA). The ssGSEA is a computational method used in genomics to assess the enrichment of predefined gene sets in individual samples. This technique is an extension of Gene Set Enrichment Analysis (GSEA), calculating separate enrichment scores for each pairing of a sample and gene set. It helps understand the biological relevance of gene sets in the context of individual samples, providing insights into pathway activities and molecular characteristics at a single-sample level. This analysis allowed us to assess the activity of the 18 RCD gene sets in individual samples. The RCD score for each sample was calculated as the sum of the ssGSEA score of the 18 RCD gene sets.

$${RCD}\,{score}\,{in}\,{bulk}\,{level}=\mathop{\sum }\limits_{i=1}^{i=18}{ssGSEA}\,{score}(i)$$

For scRNA-Seq datasets, the RCD score was determined by summing the Gene Set Variation Analysis (GSVA) scores of the 18 RCD gene sets with the R package *“GSVA”* for each single cell. The GSVA is a computational method used in genomics to assess the variation of predefined gene sets across samples. It transforms a gene-by-sample gene expression matrix into a gene set-by-sample pathway enrichment matrix, providing a more detailed understanding of biological pathways’ activities within individual samples. It has been extensively applied to perform functional enrichment analyses for scRNA-seq data^122,123^. This approach accounted for the comparatively poor gene capture rate in single cells and the high rate of dropout data, ensuring accuracy in RCD activity evaluation.

$${RCD}\,{score}\,{in}\,{single}\,{cell}\,{level}=\mathop{\sum }\limits_{i=1}^{i=18}{GSVA}\,{score}(i)$$

In order to facilitate further analysis, we divided the samples into two subgroups low-RCD and high RCD. This division was based on the median RCD score of the samples.

### Evaluation and description of the robustness and practicality of the RCD score

Several approaches were utilized to demonstrate the robustness and practical value of the RCD score. Firstly, we calculated the RCD score in tumor samples from TCGA and normal tissue samples from GTEx to investigate RCD activity across 30 cancers and their corresponding normal tissues. Spearman’ s correlation test was used to assess the correlation between the RCD score and single RCD signal activity, represented by the ssGSEA score, across different cancer types. Additionally, the interconnection among individual RCDs was investigated in the 30 cancers. The difference in RCD scores between tumor and normal tissue samples was evaluated using the Wilcoxon rank sum test, considering *p* values < 0.05 as significant.

Secondly, we obtained the ssGSEA scores of hallmark signatures and c6 oncogenic gene sets from MsigDB for all TCGA samples. We then investigated the Spearman correlations between the RCD score and these ssGSEA scores across the different cancers. A similar approach was applied to correlate the RCD score with cancer single-cell states. For the hallmark signatures, an alternative and complementary approach was used to assess the correlation with the RCD score. Samples were sorted based on the RCD score within each tumor type, and the top 35% and bottom 35% of samples were analyzed for gene expression differences. Differential genes identified were then subjected to GSEA analysis of hallmark signatures.

Thirdly, we calculated the Spearman correlations between the RCD score and the TMB and CNV score. To indicate the level of the gene copy number variation, we defined the CNV score as the sum of squared values of the copy number variation.

Lastly, the predictive capability of the RCD score across cancers was assessed using univariate cox regression and survival analysis. The cut point for cohorts was determined based on the median of the RCD score, and the analysis was validated using several independent datasets, including CGGA, GSE16011, and GSE108474.

### Computational calculation of RCD-related immune microenvironment

Immune signatures of TLSs, regulatory T cells, T cell survival, classIMHC, immunosuppression and myeloid cells chemotaxis were obtained from the literature, and their corresponding scores were calculated based on a previous study^124^.

To investigate the levels of immune cells, we utilized the ESTIMATE algorithm, MCP-counter and CIBERSORT. The R package “IOBR” and the gene expression matrix were used as inputs^125^. Specifically, the ESTIMATE algorithm was employed to assess the levels of immune cells, stromal cells and tumor purity. These were represented by the immune score, stromal score and ESTIMATE score, respectively. CIBERSORT, with 22 signatures, was used to determine the absolute abundance of 22 immune cell types, including both adaptive and innate immune cells. MCP-counter was used to estimate the level of tumor-infiltrating leukocytes.

The Spearman correlation between the RCD score and the immune-related scores was computed, and the difference in immune-related scores between the high RCD subgroup and the low RCD subgroup was investigated using the Wilcoxon rank sum test.

We collected 29 tumor microenvironment signatures and 7 antitumor immune cycle signatures from previously published studies. These signatures were applied to the ssGSEA algorithm to calculate the Spearman correlation with the RCD score.

### Evaluating the correlation of the RCD score and the ICI response

We gathered ICI response signatures and estimated their corresponding signal activity using the ssGSEA algorithm. Additionally, we calculated the Spearman correlation between the RCD score and ssGSEA score of the ICI response signatures across different cancer types.

The objective response rate (ORR) is a metric used in clinical trials and cancer treatment to evaluate the effectiveness of a therapy in shrinking or eliminating tumors. The Spearman correlation between the RCD score and the ORR was computed using the median RCD score for each individual cancer type along with the available ORR data.

To explore the relationship between the RCD score and ICI response, we obtained two scRNA-sequencing datasets with ICI response information from the GEO database. The datasets were GSE 115978 (melanoma)^81^ and GSE123813 (BCC)^126^. Only malignant cells from the scRNA-Seq data with ICI response were included in the analysis. We used the R package “*Seurat*” (version 4.0.2) to investigate the relationship.

In the melanoma scRNA-Seq dataset, we included 24 patients, consisting of 11 non-responders and 13 treatment-naïve patients, excluding 7 patients without malignant cells. In the BCC scRNA-Seq dataset, we included 10 patients, with 6 responders and 4 non-responders, excluding one patient with abnormal mutation and transcriptomic profile. After calculating the RCD score for each single-cell, we compared the differences in RCD score among different types of ICI response.

### Identification of the RCD Signature (RCD.Sig)

We employed a four-step framework to identify RCD score-related genes at both the bulk and single-cell levels. First, we performed Spearman correlation tests to identify genes significantly correlated with the RCD score in each individual dataset. Genes with a *p* value < 0.05 and an absolute Spearman correlation >0 were considered as significantly correlated genes.

Second, we conducted a differential analysis. At the bulk level, we identified differentially expressed genes between the high RCD subgroup and the low RCD group using the Wilcoxon rank sum test (*p* < 0.05). For the single-cell level, we identified differentially expressed genes specifically in malignant cells compared to other cells within each dataset (logFC >0.25, *p* < 0.05).

Third, the genes satisfying both the first and second steps in each dataset were extracted and named Gn.

Fourth, the geometric mean of the Spearman correlation was calculated for each gene across the datasets. Genes with a geometric mean of Spearman correlation >0.35 or >0.2 were identified as RCD candidate genes in the bulk or single-cell level, respectively.

Finally, the RCD.Sig was defined as the intersection of the RCD candidate genes identified in both the bulk and single-cell levels.

### Development and evaluation of the machine-learning model for predicting the ICI response

To assess the predictive performance of the RCD.Sig for ICI response, we employed seven machine-learning methods: Naive Bayes (NB), AdaBoost Classification Tree (AdaBoost), cancerclass, random forest (RF), boost logistic regression (“LogiBoost”), k-nearest neighbors (“KNN”) and support vector machine (“SVM”). Nested cross-validation (CV) was utilized for benchmarking these methods.

The performance of the trained models was evaluated on the validation dataset, and the model with the highest AUC was selected as the optimal RCD.Sig model for the prediction of ICI response. Independent test datasets were then used to assess the performance of the optimal model.

Furthermore, we compared the predictive performance of the RCD.Sig model with 13 previously published ICI response models. This comparison was conducted on the validation set, the combined test set, the NSCLC set, the SKCM set, and six individual test sets.

### Development and evaluation of the machine-learning model for predicting the OS

To construct the RCD.Sur.Sig model (RCD.Sig-based model for the OS), using transcriptomic data from 9890 samples and the RCD.Sig, we employed a novel computational framework that involved multiple machine-learning algorithms. Here are the details of the methodology:

- Data preprocessing: Samples in TCGA without OS information or with a survival time of 0 were excluded, resulting in the inclusion of a cohort of 9750 samples. These samples were randomly split into three cohorts, training cohort (50%, *n* = 4868), testing set 1 (30%, *n* = 2916) and testing set 2 (20%, *n* = 1966).
- Feature Selection: Univariate Cox regression analysis and log-rank survival analysis were performed on the entire TCGA cohort of 9750 samples to identify genes within the RCD.Sig that exhibited potential prognostic prediction. These genes were selected for further analysis.
- Model construction: The LOOCV framework was applied, involving 101 combinations of 10 machine-learning algorithms, including Ridge, CoxBoost, survival forest (RSF), partial least squares regression for Cox (plsRcox), generalized boosted regression model (GBM), Lasso, stepwise Cox, elastic network (Enet), supervised principal components (SuperPC) and survival support vector machine (survival-SVM). The aim was to identify the optimal model with the best mean C-index in the testing datasets.
- RCD.Sur.Sig development: The RCD.Sur.Sig was created by combining the stepwise Cox regression analysis, which identified the most valuable and prognostic genes within the RCD.Sig, and the RSF, which aided the development of the survival prediction model using the RCD.Sur.Sig. The log-rank score splite-rule, based on the log-rank scores, was strictly implemented as described in a published study^127^. Specifically, assuming the ranks of the ordered variables (T, δ) as X1 < X2 < …<Xn, the log-rank scores are as follow:

$${a}_{l}={{\rm{\delta }}}_{l}-\mathop{\sum }\limits_{k=1}^{{\rm{\delta }}({\rm{T}})}\frac{{{\rm{\delta }}}_{k}}{N-{{\rm{\gamma }}}_{k}\left(T\right)+1}$$

where

$${{\rm{\gamma }}}_{k}\left(T\right)=\mathop{\sum }\limits_{l=1}^{N}x\left\{{{\rm{T}}}_{l}\le {{\rm{T}}}_{k}\right\}$$

is the number of dead patients before or at time ${{\rm{T}}}_{k}$.

$${RCD}.{Sur}.{Sig}\,{risk}\,{score}={\rm{M}}\left(x,b\right)=\frac{{\sum }_{{x}_{j}\le b}\left({a}_{j}-{R}_{1}\bar{a}\right)}{\sqrt{{R}_{1}\left(1-\frac{{R}_{1}}{N}\right){S}_{a}^{2}}}$$

where $\bar{a}$ and ${S}_{a}^{2}$ are the mean and the variance of ${a}_{l}$ [*l* = 1,2,3…*n*], and the optimal split is determined by maximizing |${\rm{M}}\left(x,b\right)$| across x and b.

We conducted survival predictive performance testing of the RCD.Sur.Sig model using external and independent datasets as mentioned previously. Additionally, a comprehensive hazard ratio analysis was done to elucidate the combined prognostic predictive performance of the RCD.Sur.Sig model across 23 cohorts. This analysis was carried out through a prognostic meta-analysis using the R package “meta”.

For feature selection, we implemented a novel computational framework based on seven machine-learning algorithms to identify the hub genes within RCD.Sig across pan-cancer, low-grade glioma and glioblastoma. Initially, we employed univariate Cox regression analysis and log-rank survival analysis to filter out the prognostic genes within RCD.Sig. Subsequently, we utilized seven machine-learning algorithms, namely SVM, Boruta, XgBoost, Lasso, RF, Enet, and CoxBoost, to further refine the selection and identify the hub and core genes. These genes were determined by intersecting the genes identified by the aforementioned algorithms in pan-cancer, LGG and GBM, respectively.

### Investigation of the RCD score with spatial scRNA-Seq data of glioblastoma

Processed spatial transcriptomic data were downloaded from R package SPATAData (https://github.com/theMILOlab/SPATAData) and analyzed by R package SPATA2. For the spatial scRNA-Seq datasets, the RCD score was determined by summing the Gene Set Variation Analysis (GSVA) scores of the 18 RCD gene sets with the R package *“GSVA”* for each single cell. This approach accounted for the comparatively poor gene capture rate in single cells and the high rate of dropout data, ensuring accuracy in RCD activity evaluation.

$${RCD}\,{score}\,{in}\,{single}\,{cell}\,{level}=\mathop{\sum }\limits_{i=1}^{i=18}{GSVA}\,{score}(i)$$

### Statistical analysis

All statistical analyses in this study were conducted using R software (version 4.1.3). The Wilcoxon rank sum test and Kruskal-Wallis test were employed to investigate differences between two subgroups or among more than two subgroups for continuous variables. The Chi-Square test was utilized to assess differences in categorical data.

The Benjamini-Hochberg correction method was applied to obtain adjusted p values. All p values were two-sided. All relevant data used in this study was summarized in supplementary table 20.

### Reporting summary

Further information on research design is available in the Nature Research Reporting Summary linked to this article.

### Supplementary information

Supplementary information files

Reporting Summary

## Data Availability

No new data were generated as part of this study. All data used in this study were sourced from the public domain online. All relevant data used in this study was summarized in supplementary Table 20.

## Code availability

Additionally, all key codes utilized in this study and the raw data for every figure are available on Github (https://github.com/zwxiangya/RCDscore). Other codes used in the current study could be available by contacting the corresponding author.
