# Supplementary material for: Pan-cancer evaluation of regulated cell death to predict overall survival and immune checkpoint inhibitor response
Source: NPJ Precis Oncol. 2024 Mar 27;8:77. doi: 10.1038/s41698-024-00570-5 (PMC10973470; doi:10.1038/s41698-024-00570-5)
Supplement: Supplementary file 1 — Supplementary information files [file 41698_2024_570_MOESM1_ESM.pdf]

Supplementary Figures

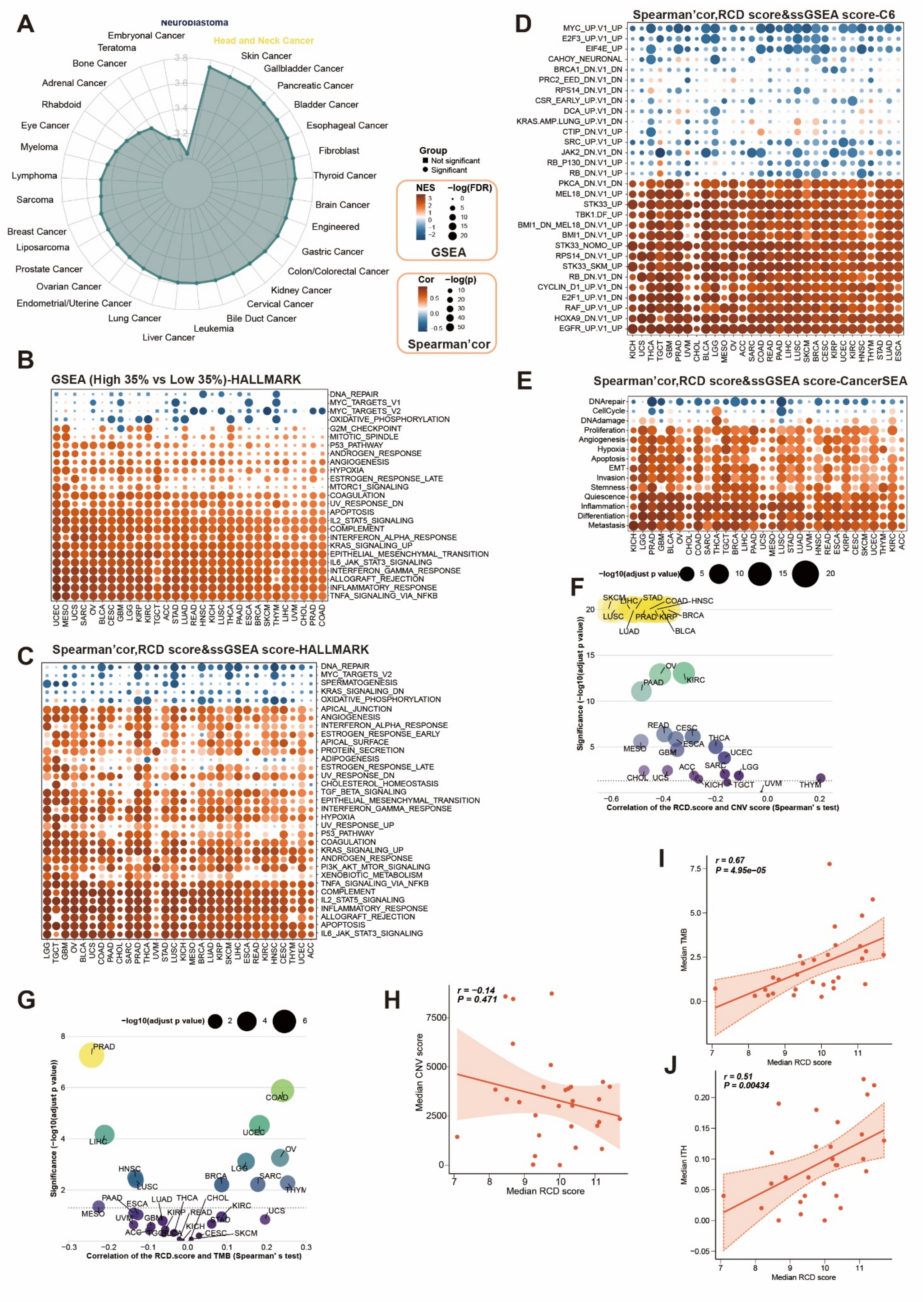

Supplementary figure 1. The association of the RCD score and the functional pathway signals and genomic variations at the pan-cancer level.

A. The average of the RCD scores in the cancer cell lines from the different cancers.
B. The enrichment results of the HALLMARK gene set with the GSEA method. The differential expression genes between the samples with 35% top RCD score and samples with 35% tail RCD score were used for GSEA in individual cancer type, and 25 pathways with good concordance across the 30 cancers were shown. The redder the color of the dots in the graph, the more active the pathway was, while the bluer the color of the dots, the less active the pathway was.
C. The association between the RCD score and the ssGSEA score of the hallmark signatures. The ssGSEA algorithm was performed for hallmark signatures and the Spearman correlation between the RCD score and the NES was calculated in individual cancer type. 33 pathways with good concordance in 30 cancer types were displayed.
D. The Spearman association between the RCD score and the ssGSEA score of the C6 oncogenic signatures. The calculation method was similar with that in previous graph.
E. The Spearman association between the RCD score and the ssGSEA score of the cancer single cell state signatures. The computation method was as described previously.
F. The association of the RCD score and the CNV score in individual cancer type. The Spearman correlation between the RCD score and the CNV score was computed in each cancer and the results were shown. The horizontal coordinate indicated the correlation coefficient, and the vertical coordinate indicated the significance, which was presented by –log10 (adjust p value).
G. The association of the RCD score and the TMB score in individual cancer.
H. Spearman correlation between the median RCD score and the median CNV score of each cancer type in TCGA.
I. The association of the RCD score and the TMB across the cancers. The Spearman correlation was calculated with the median RCD scores and median TMB in each cancer type. The position of each dot was determined by the horizontal coordinate (meidan RCD score) and vertical coordinate (median TMB).
J. The association of the RCD score and the ITH across the cancers. The calculation method was described previously.

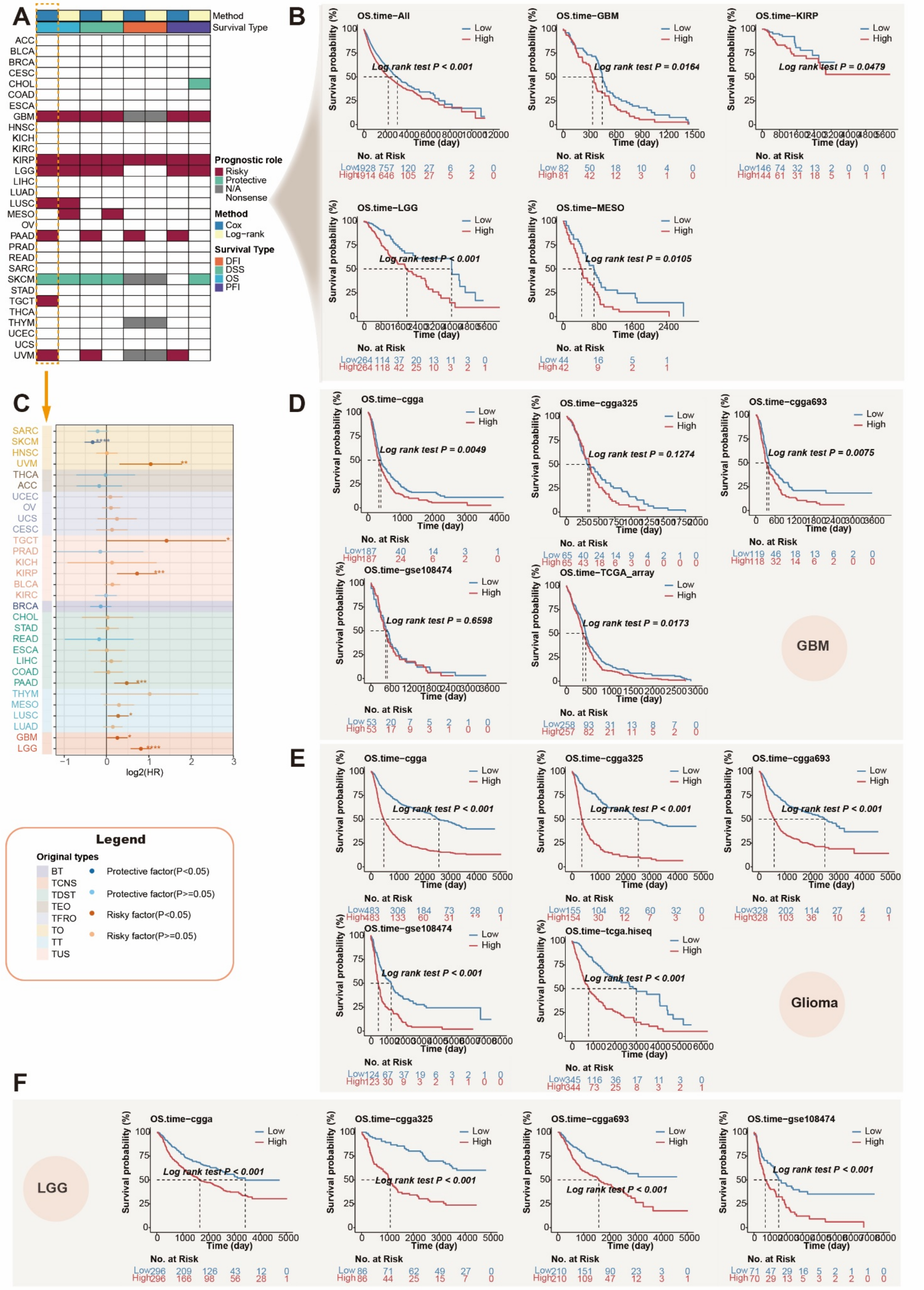

Supplementary figure 2. Impact of RCD score heterogeneity on clinical outcomes.

A. The association of RCD score and the clinical outcomes including overall survival (OS), disease specific survival (DSS), disease free interval (DFI) and progression free interval (PFI), in pan-cancer, with utilization of two methods including univariate Cox regression and log-rank test in TCGA. The p value < 0.05 and HR > 1 was considered as risky. The p value < 0.05 and HR < 1 was considered as protective. The p value ≥0.05 was considered as nonsense. "N/A" indicated that the corresponding data was missing.
B. The Kaplan-Meier survival plot in several representative tumor types of TCGA. The cutoff point of the cohorts was the median RCD score in corresponding cohorts.
C. The forest plot showing the univariate Cox regression result of the RCD score in pan-cancer. TCNS, Tumors of Central Nervous System; TT, Thoracic Tumors; TDST, Tumors of Digestive System; BT, Breast Tumor; TUS, Tumors of Urinary System and Male Genital Organs; TFRO, Tumors of Female Reproductive Organs; TEO, Tumors of Endocrine Organs; TO, Tumors of others.
D. The Kaplan-Meier survival plot in glioblastoma with external validation datasets including CGGA, CGGA325, CGGA693, GSE108474 and TCGA-array. The cutoff point of the cohorts was the median RCD score in corresponding cohorts.
E. The Kaplan-Meier survival plot in glioma with external validation datasets including CGGA, CGGA325, CGGA693, GSE108474 and TCGA-Hiseq (TCGA.LGG-GBM). The cutoff point of the cohorts was the median RCD score in corresponding cohorts.
F. The Kaplan-Meier survival plot in glioblastoma with external validation datasets including CGGA, CGGA325, CGGA693 and GSE108474. The cutoff point of the cohorts was the median RCD score in corresponding cohorts.

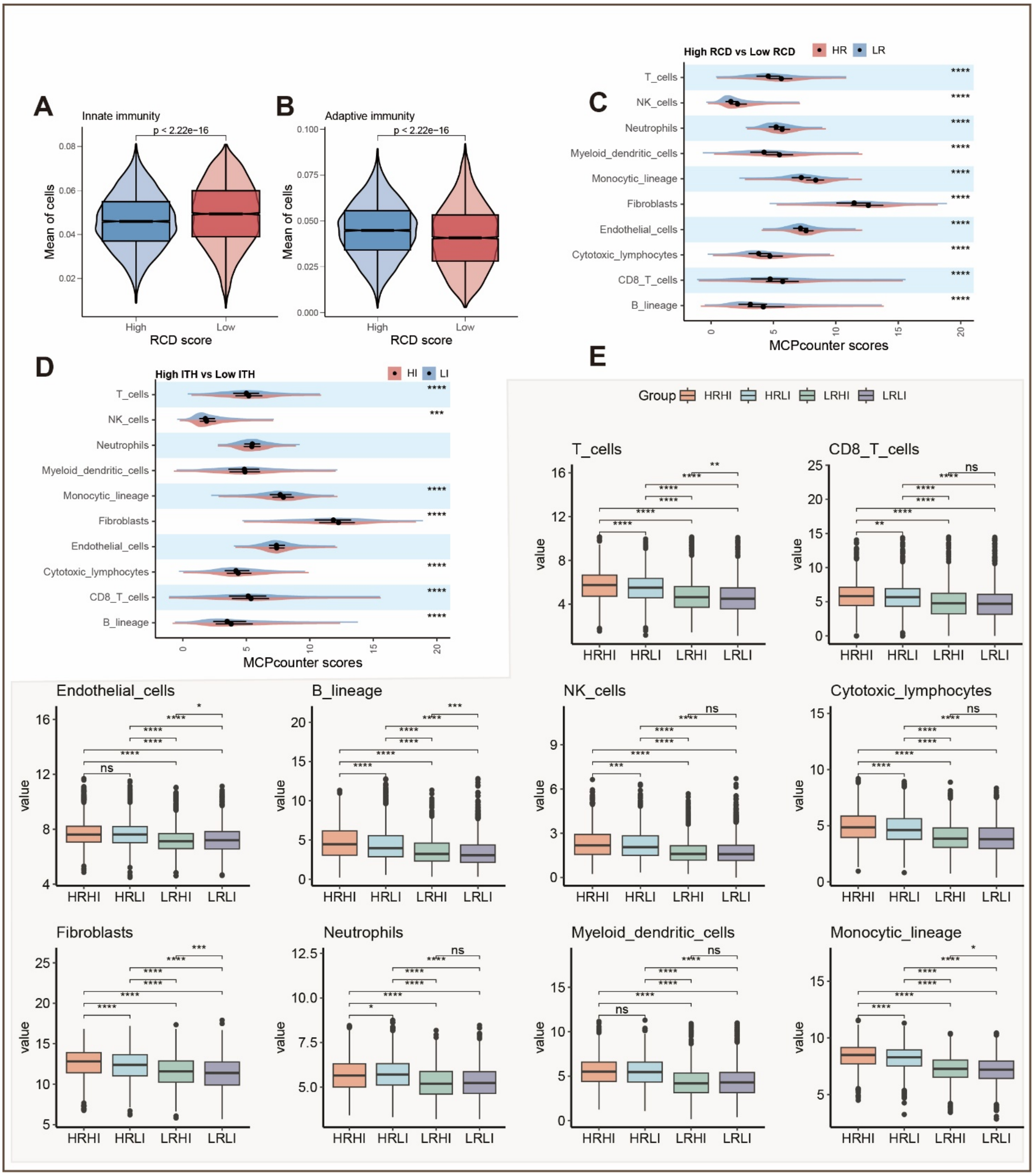

Supplementary figure 3.

A. The difference of the mean of cell numbers between the high RCD subgroup and low RCD subgroup for innate immune cells. The cells were divided into high and low RCD subgroups based on the median RCD score. Wilcoxon rank sum test was used here.

B. The difference of the mean of cell numbers between the high RCD subgroup and low RCD subgroup for adaptive immune cells. The cells were divided into high and low RCD subgroups based on the median RCD score. Wilcoxon rank sum test was used here.

C. The difference of immune cells calculated by the MCP-counter algorithm between the high RCD subgroup and low RCD subgroup, which were obtained based on the median of the RCD score in each cancer type.

D. The difference of immune cells calculated by the MCP-counter algorithm between the high ITH subgroup and low ITH subgroup, which were obtained based on the median of the ITH score in each cancer type. ITH: Intra-tumor heterogeneity.

E. For ten immune cell types, the difference among the four subgroups, including HRHI, HRLI, LRHI and LRLI. HRHI: High RCD High ITH. HRLI: High RCD low ITH. LRHI: Low RCD high ITH. LRLI: Low RCD low ITH. (Mann-Whitney U test; ns, not significant; *P < 0.05, **P < 0.01, ***P < 0.001, ****P < 0.0001).

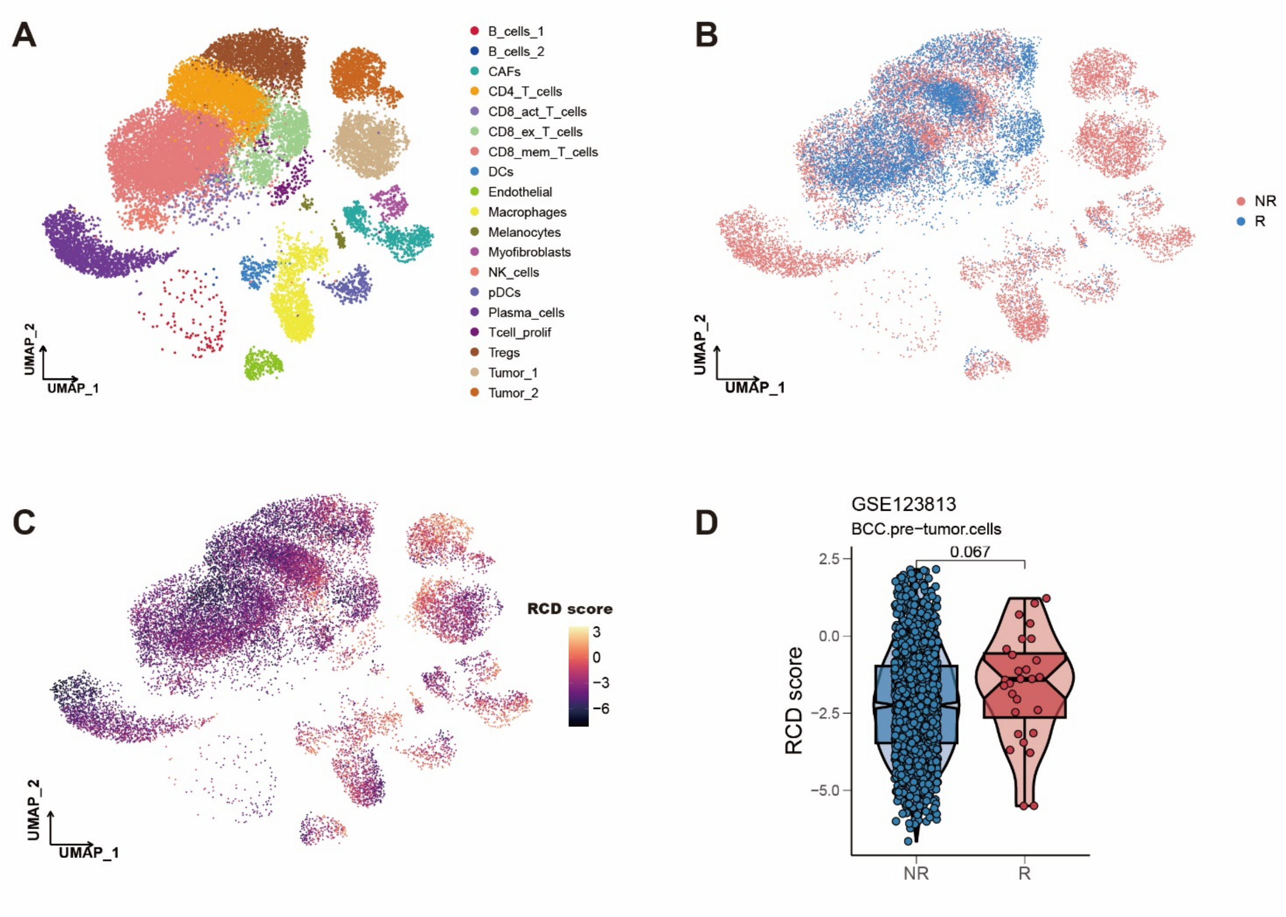

Supplementary figure 4. Validation of the association between the RCD score and the immunotherapy response in GSE123813.

A. UMAP plot of the identified cell types in GSE123813 (BCC). Different colors represented the different cell types.

B. UMAP plot of the identified cells colored by the immunotherapy response.

C. UMAP plot of the identified cells colored by the RCD score. The RCD score was calculated as described in methods.

D. The difference of RCD score between the NR and R in BCC cohort. The center of the box pot was the median values, the bounds of the box were the 25% and 75% quantiles. Wilcoxon rank sum test was used for estimating the difference. NR: non-responder. R: responder.

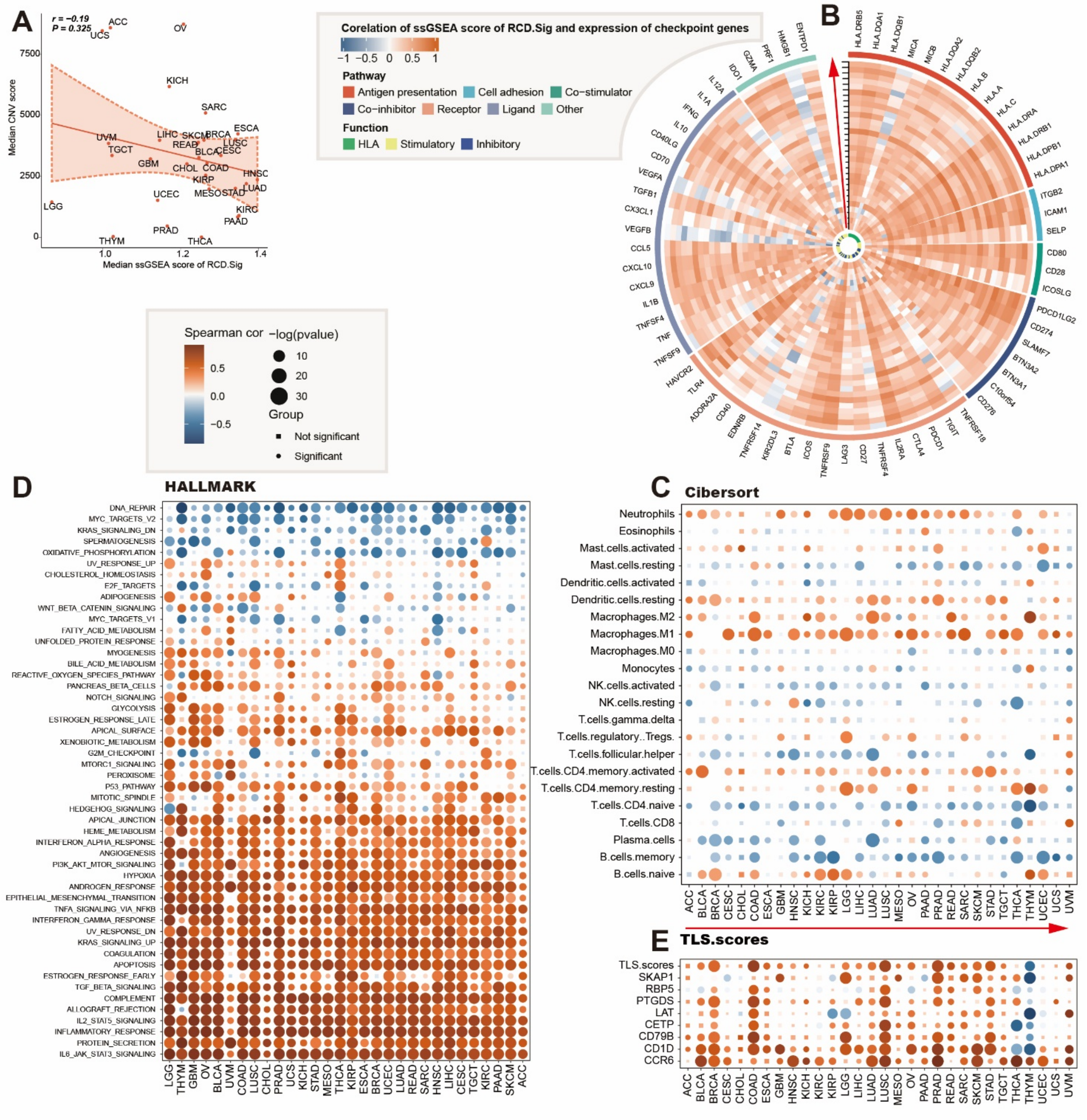

Supplementary figure 5. Association analysis between RCD.Sig score and several features in multi-omics.

A. Spearman correlation between the median RCD.Sig score and the median CNV score of each cancer type in TCGA. RCD.Sig score was calculated by the ssGSEA of the RCD.Sig.
B. The circos plot showing the association between the RCD.Sig score and the gene expression of the immune check points in individual cancer type, with Spearman correlation analysis. From inside to outside of the circle heat map, the vertical axis with a black arrow indicated the different cancer types, which were annotated in by the x axis of plot C.
C. Spearman correlations (color) between RCD.Sig scores and the absolute abundance of 22 immune cell types estimated by CIBERSORT for individual TCGA cancer types.
D. The association between the RCD.Sig score and the ssGSEA score of the hallmark signatures. The ssGSEA algorithm was performed for hallmark signatures and the Spearman correlation between the RCD score and the

NES was calculated in individual cancer type.

E. Spearman correlations (color) between RCD.Sig scores and the TLS scores and between RCD.Sig scores and the expression of the genes in TLS signature for individual TCGA cancer types. TLS scores was calculated by ssGSEA of the TLS signature.

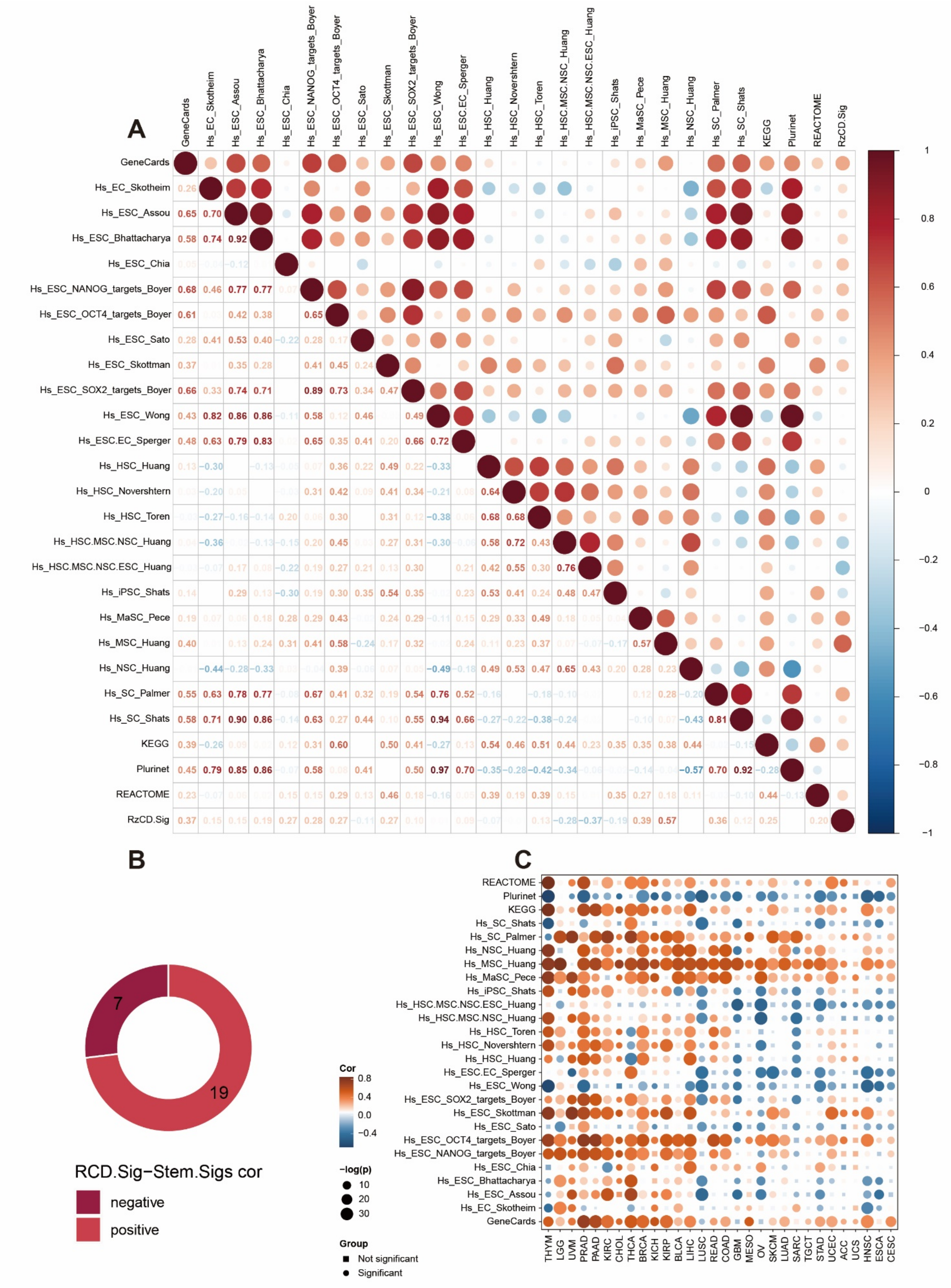

Supplementary figure 6. Association analysis between RCD.Sig and stemness related signatures.

A. The heat map showing the Spearman correlation between the RCD.Sig and 26 stemness related signatures, using the ssGSEA algorithm in TCGA to calculate scores for each stemness-related feature.
B. The pie plot showing the proportion and the number of the stemness related signatures which were positively and negatively associated with the RCD.Sig score.
C. Spearman correlation between the RCD.Sig scores and the 26 stemness related signatures for each cancer type in TCGA, using the ssGSEA algorithm in TCGA to calculate scores for each stemness-related feature.

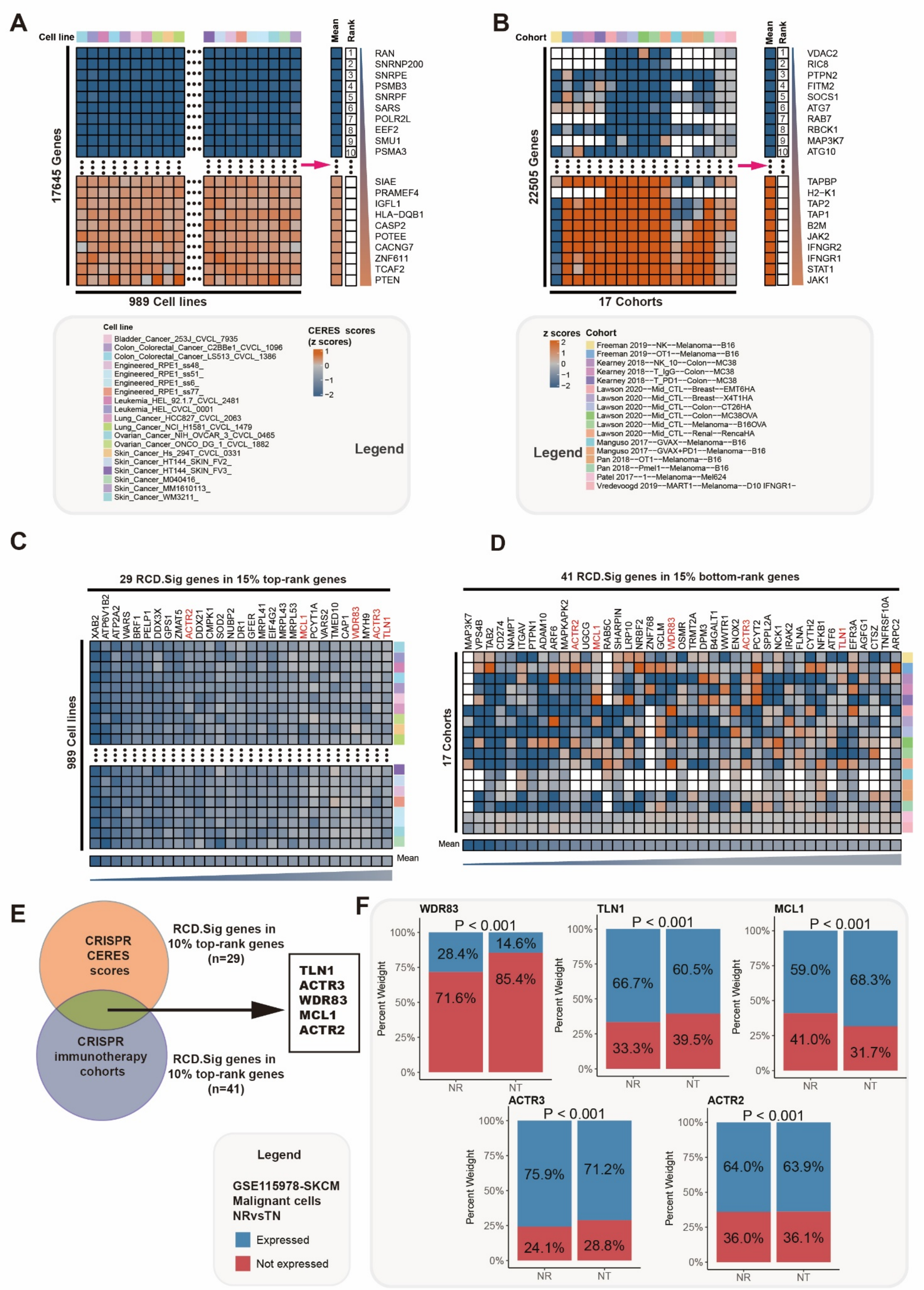

Supplementary figure 7. Exploration of potential therapeutic targets from RCD.Sig utilizing the CRISPR screen data.

A. Ranking of genes based on their knockout effects on cell growth and proliferation across 989 CRISPR cell lines. Negative (positive) z scores indicated worse (better) growth and proliferation after knockout of a specific gene. Genes were ranked according to their average of z scores. Top-ranking genes were more importance for the cell growth and proliferation.
B. Ranking of genes based on their knockout effects on anti-tumor immunity across 17 CRISPR cohorts. Negative (positive) z scores indicated better (worse) anti-tumor immunity after knockout of a specific gene. Genes were ranked according to their average of z scores. Top-ranking genes were more likely associated with the resistance of the anti-tumor immunity.
C. The heat map showing the z scores of 29 RCD.Sig genes in the 10% top-rank genes across 989 CRISPR cell lines.
D. The heat map showing the z scores of 41 RCD.Sig genes in the 10% top-rank genes across 17 CRISPR cohorts.
E. The potential therapeutic targets were identified as the intersection of the 29 RCD.Sig genes in 10% top-rank genes across cell lines and 41 RCD.Sig genes in 10% top-rank genes across immunotherapy cohorts. The five potential therapeutic targets were TLN1, ACTR3, WDR83, MCL1 and ACTR2.
F. The difference of the number of the cells with expression of the specific gene and cells without expression of the gene between the patients with NR and patients with NT in malignant cells of GSE115978 (SKCM) for the five genes, including TLN1, ACTR3, WDR83, MCL1 and ACTR2. The chi-square test was used here. NT: naïve treatment. NR: no response.

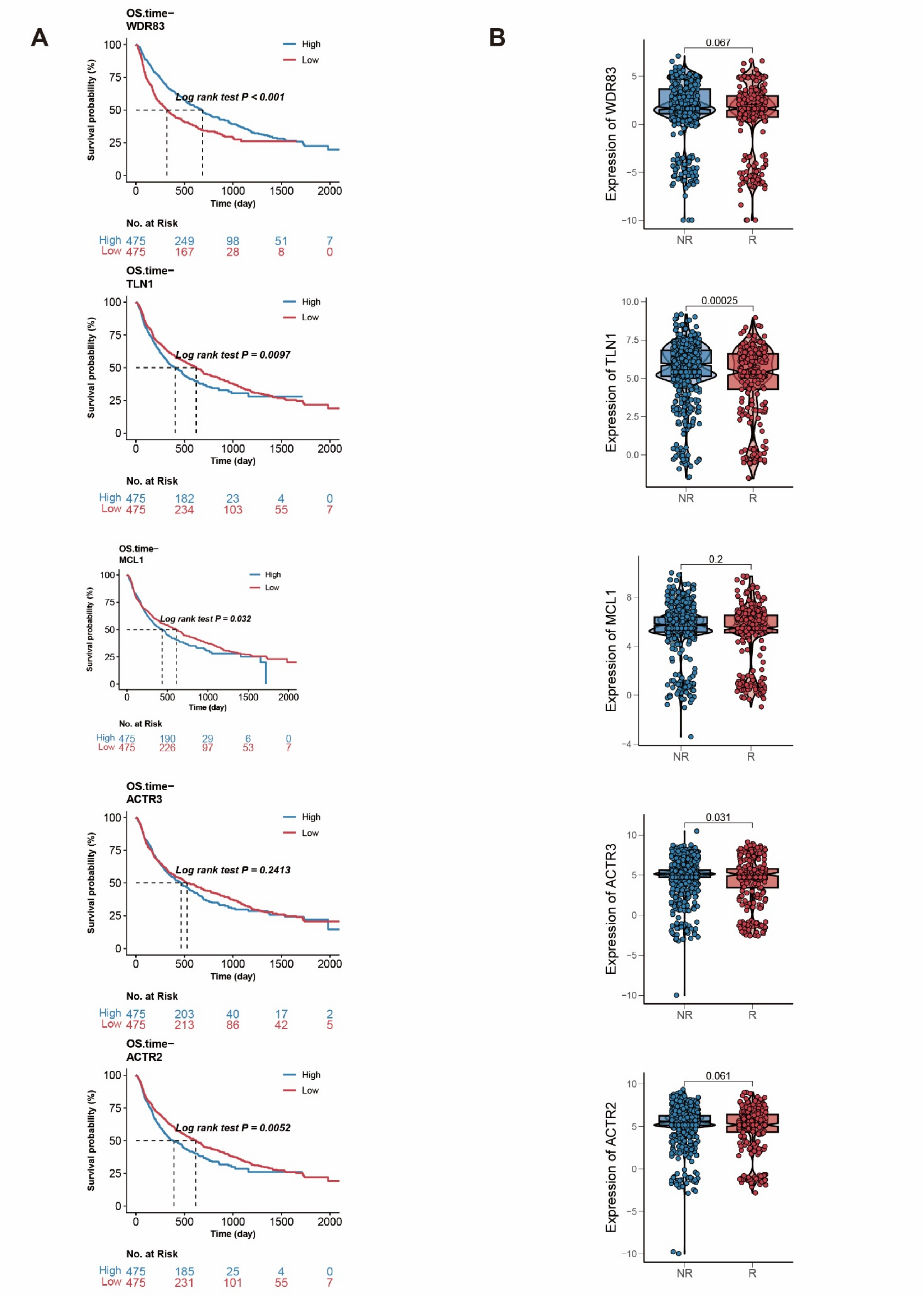

Supplementary figure 8. The survival analysis and the differential expression analysis of the hub genes in the combined immunotherapy cohort.

A. The K-M curves showing the difference of the overall survival between the high group and low group, which were divided based on the median of the expression, for the five genes including WDR83, TLN1, MCL1, ACTR3 and ACTR2.
B. The difference of the expression of the five genes between the NR group and the R group. NR: non-responder. R: responder.

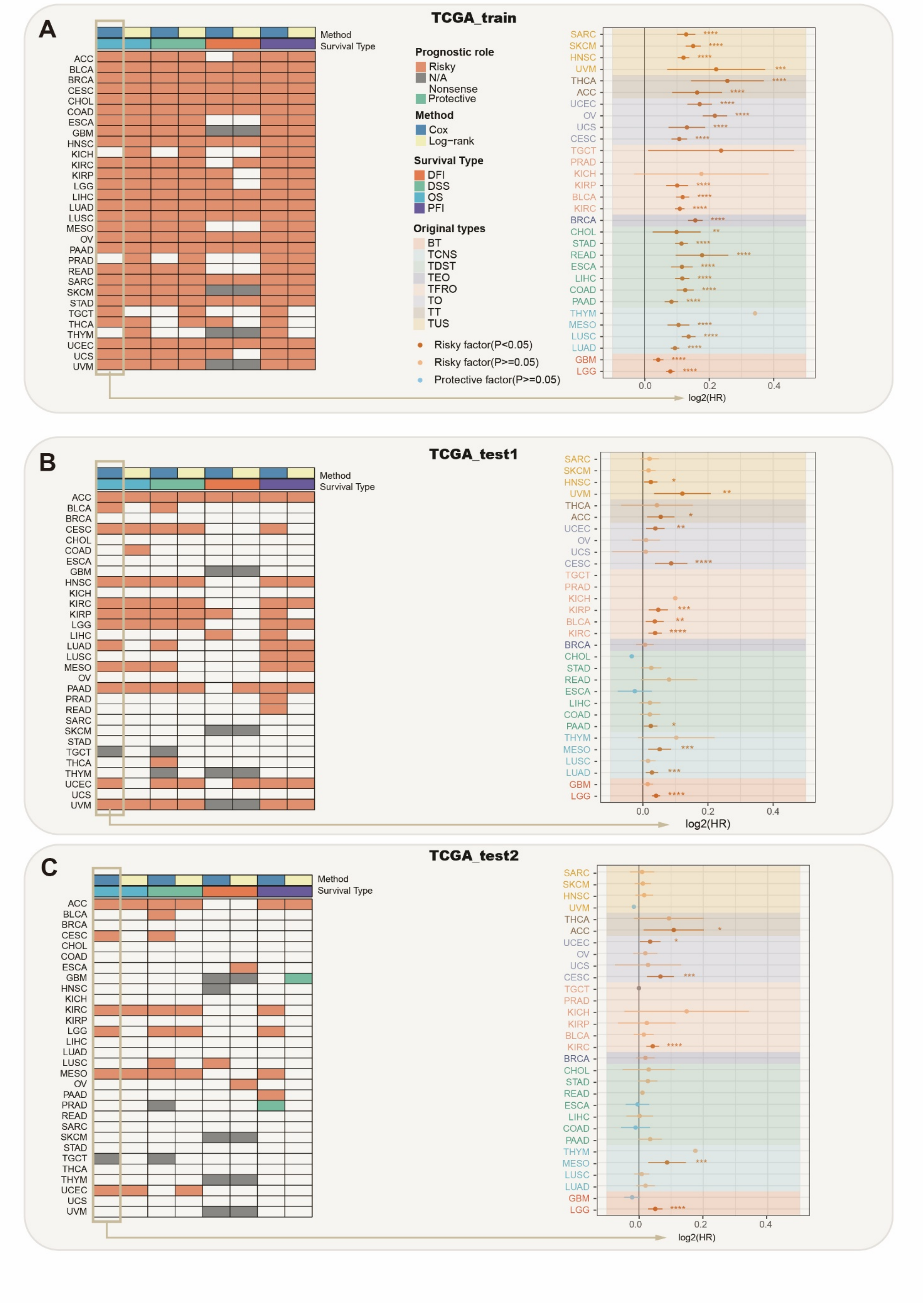

Supplementary figure 9. Association analysis between risk score calculated by the optimal overall survival predictive model and clinical outcomes.

A-C. The correlation of the risk score computed by the optimal survival predictive model and the clinical outcomes, including OS, DFI, DSS and PFI, in TCGA training dataset (A), TCGA testing dataset 1 (B) and TCGA testing dataset 2 (C), using the univariate Cox regression analysis and the log-rank test. The p value < 0.05 and HR > 1 was considered as risky. The p value ≥ 0.05 was considered as nonsense. “N/A” indicated that the corresponding data was missing. The forest plot showing the univariate Cox regression result of the risk score in TCGA training dataset (A), TCGA testing dataset 1 (B) and TCGA testing dataset 2 (C). TCNS, Tumors of Central Nervous System; TT, Thoracic Tumors; TDST, Tumors of Digestive System; BT, Breast Tumor; TUS, Tumors of Urinary System and Male Genital Organs; TFRO, Tumors of Female Reproductive Organs; TEO, Tumors of Endocrine Organs; TO, Tumors of others

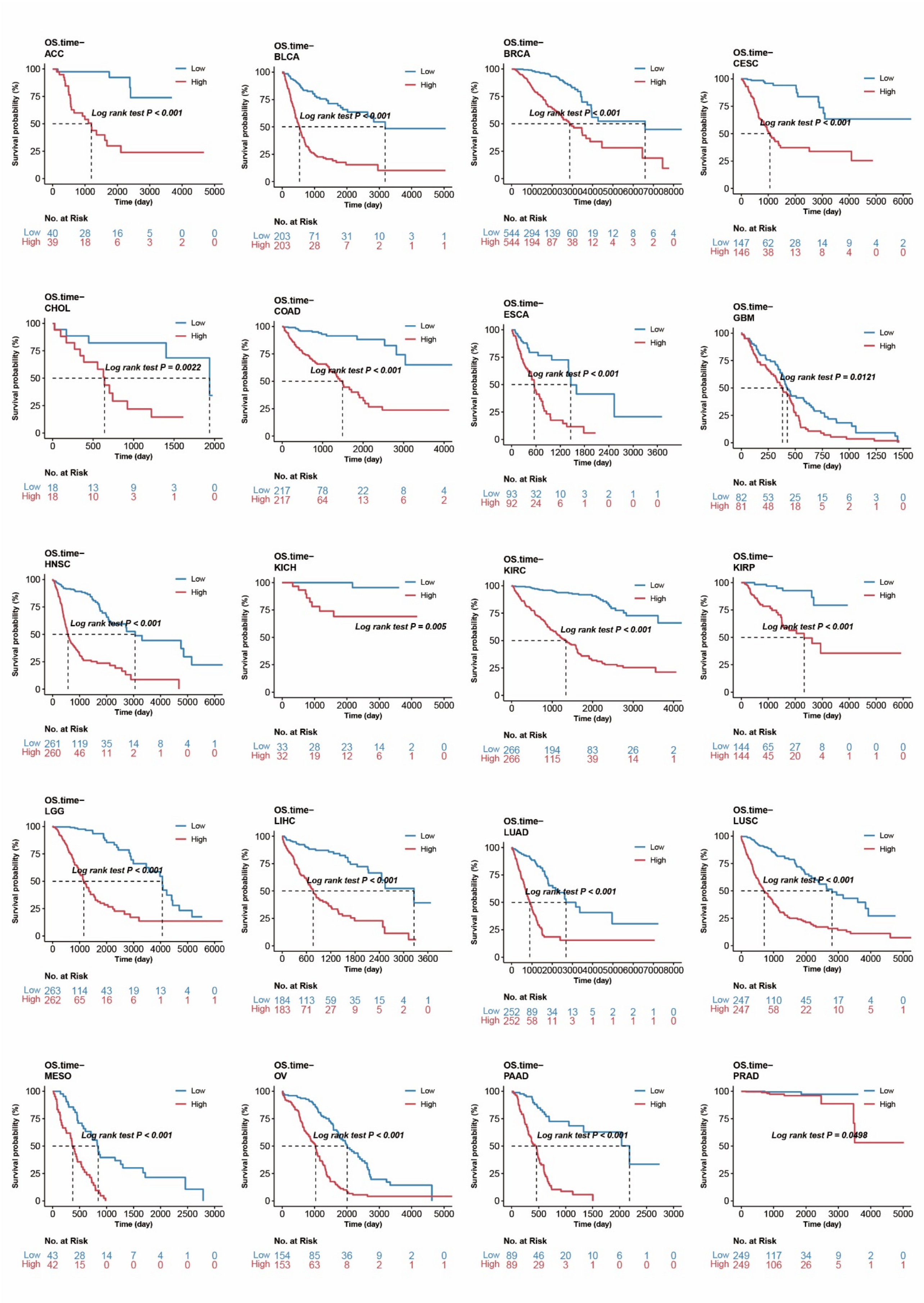

Supplementary figure 10. The Kaplan-Meier survival plot in 20 tumor types of TCGA to compare the overall survival between the high risk group and low risk group. The cutoff point of the cohorts

was the median risk score calculated by the optimal survival predictive model in corresponding cohorts.

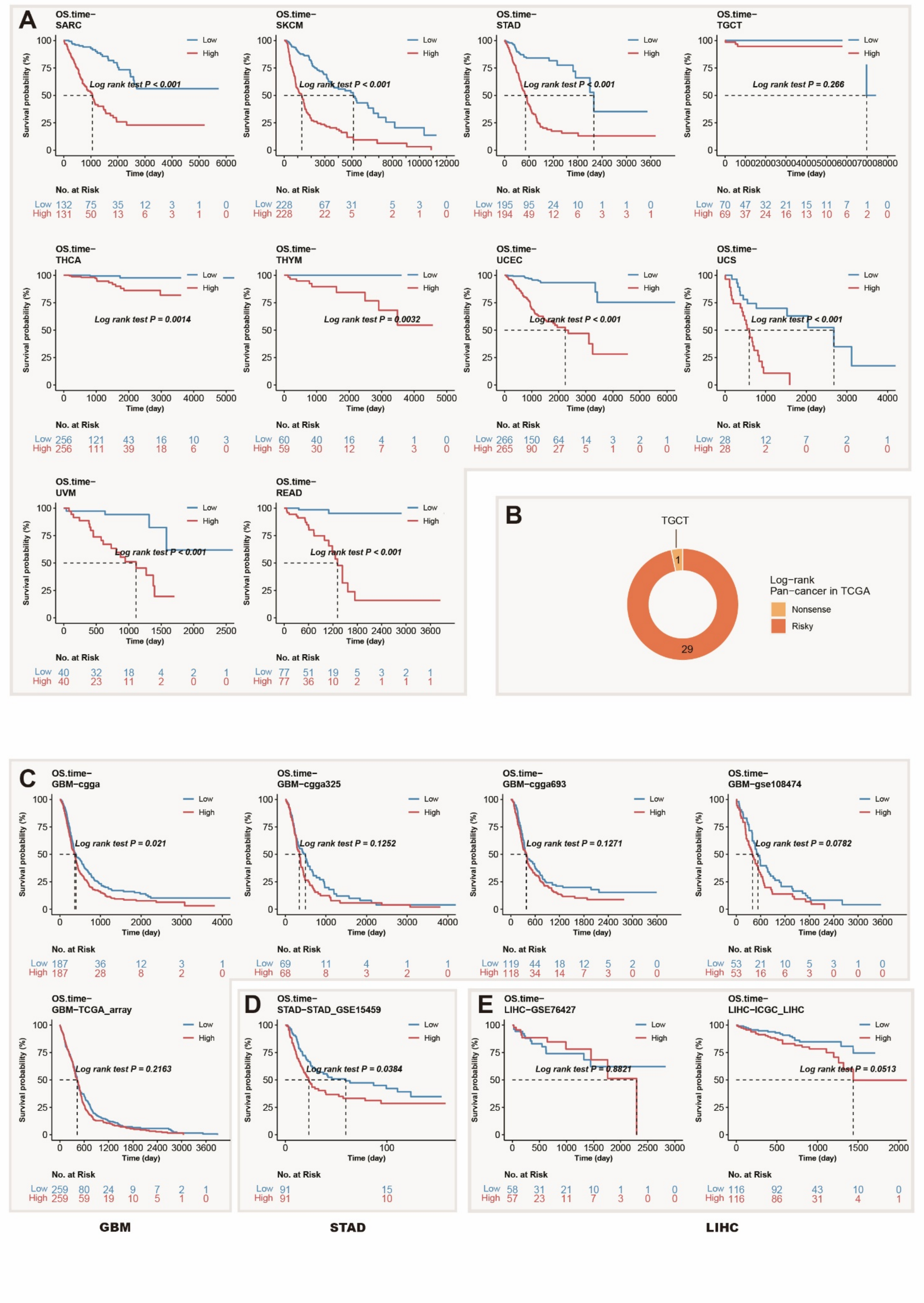

Supplementary figure 11.

A. The Kaplan-Meier survival plot in other 10 tumor types of TCGA to compare the overall survival between the

high risk group and low risk group. The cutoff point of the cohorts was the median risk score calculated by the optimal survival predictive model in corresponding cohorts.

B. The donut plot showing the proportion and number of cancer types in which the survival predictive model had the powerful and robust performance for all 30 cancer types in TCGA.

C. The Kaplan-Meier survival plot in glioblastoma of external independent cohorts to validate the robustness and powerful performance of the model. The cutoff point of the cohorts was the median risk score calculated by the optimal survival predictive model in corresponding cohorts.

D. The Kaplan-Meier survival plot in stomach adenocarcinoma of external independent cohort to validate the robustness and powerful performance of the model. The cutoff point of the cohorts was the median risk score calculated by the optimal survival predictive model in corresponding cohorts.

E. The Kaplan-Meier survival plot in liver hepatocellular carcinoma of external independent cohort to validate the robustness and powerful performance of the model. The cutoff point of the cohorts was the median risk score calculated by the optimal survival predictive model in corresponding cohorts.

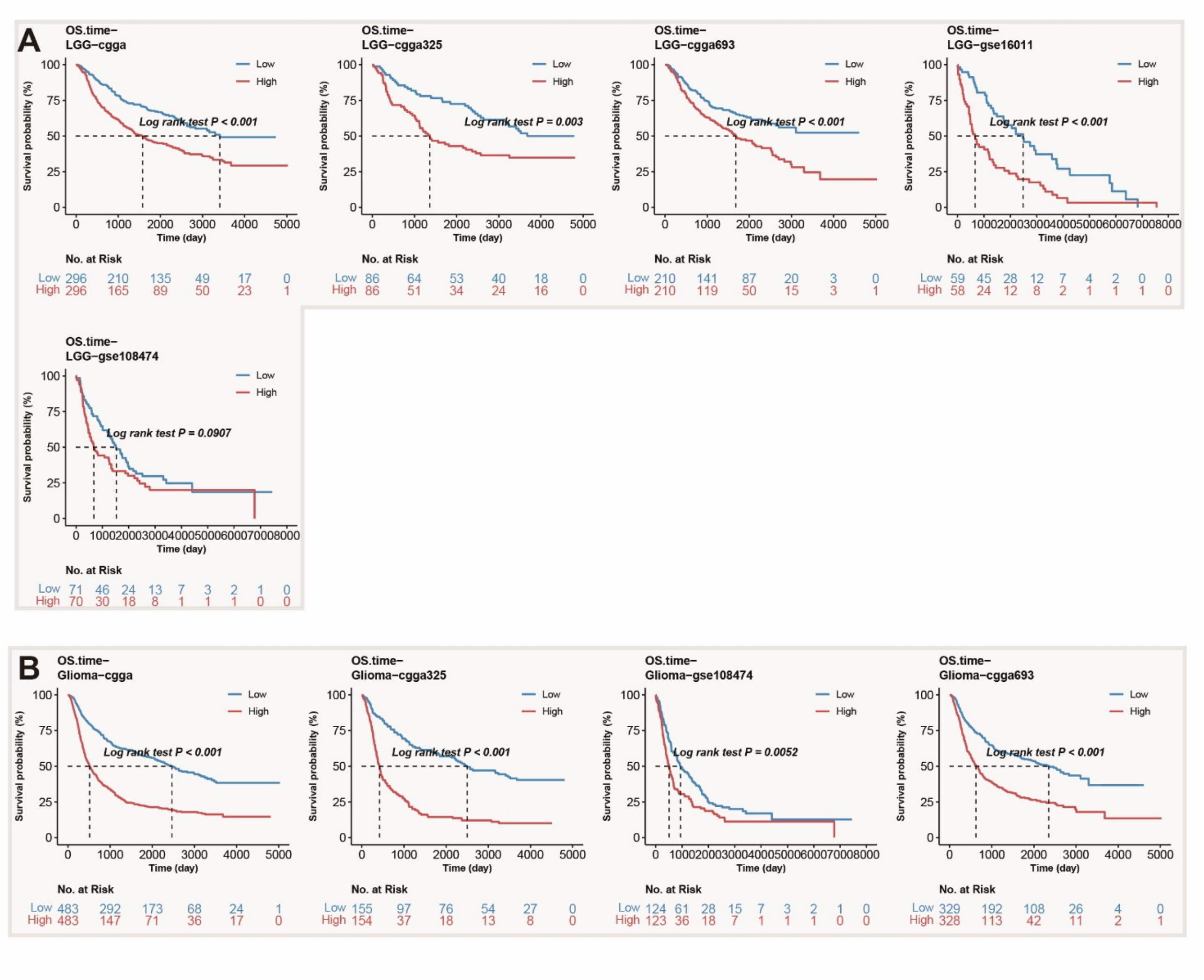

Supplementary figure 12.

A. The Kaplan-Meier survival plot in low grade glioma of external independent cohorts to validate the robustness and powerful performance of the model. The cutoff point of the cohorts was the median risk score calculated by the optimal survival predictive model in corresponding cohorts.

B. The Kaplan-Meier survival plot in glioma of external independent cohorts to validate the robustness and powerful performance of the model. The cutoff point of the cohorts was the median risk score calculated by the

optimal survival predictive model in corresponding cohorts.

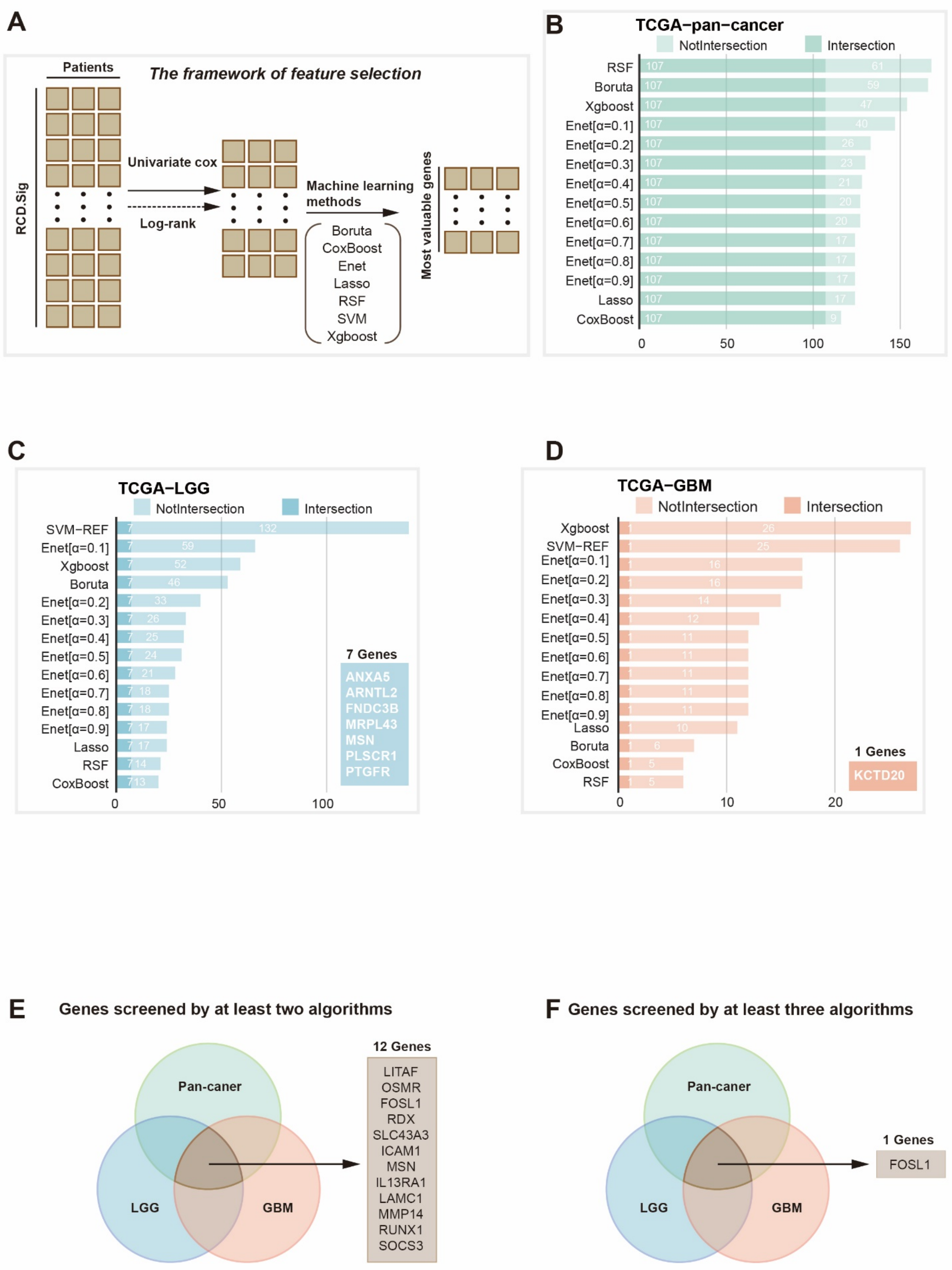

Supplementary figure 13. The core prognostic feature selection of the RCD.Sig.

A. The work flow of core prognostic feature selection based on the RCD.Sig with 7 machine learning algorithms in TCGA training dataset, TCGA-LGG dataset and TCGA-GBM dataset. The details were summarized in methods.
B. The bar plot showing the number of core features screen out by all machine learning algorithms in TCGA training dataset. There were 107 genes screened out by all machine learning algorithms.
C. The bar plot showing the number of core features screen out by all machine learning algorithms in TCGA-LGG dataset. There were 7 genes screened out by all machine learning algorithms. They were ANXA5, ARNTL2, FNDC3B, MRPL43, MSN, PLSCR1 and PTGFR.
D. The bar plot showing the number of core features screen out by all machine learning algorithms in TCGA-GBM dataset. There was 1 genes screened out by all machine learning algorithms. It was KCTD20.
E. The venn plot showing the common genes of the genes screened by at least two algorithms in TCGA training dataset, TCGA-LGG dataset and TCGA-GBM dataset. 12 genes including LITAF, OSMR, FOSL1, RDX, SLC43A3, LCAM1, MSN, IL13RA1, LAMC1, MMP14, RUNX1 and SOCS3 were identified as the core genes for the overall survival time in glioma.
F. The venn plot showing the common genes of the genes screened by at least three algorithms in TCGA training dataset, TCGA-LGG dataset and TCGA-GBM dataset. FOSL1 was obtained and identified as the hub gene for the overall survival time in glioma.

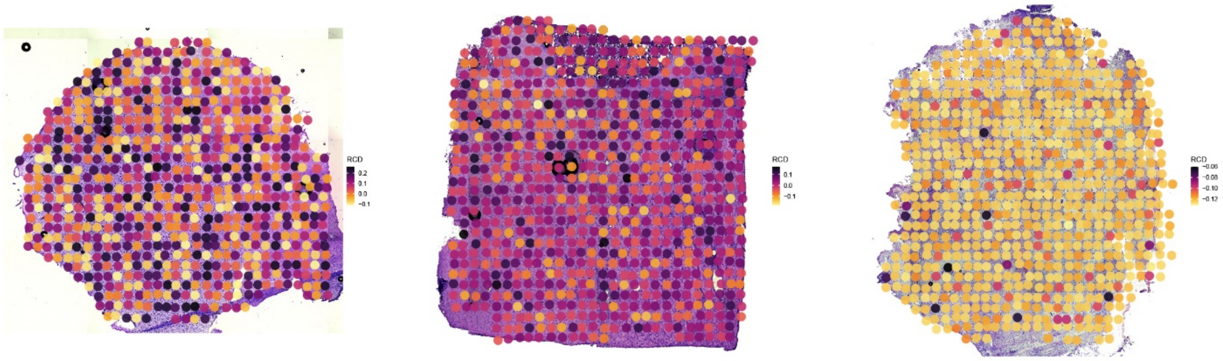

Supplementary figure 14. The exploration of the RCD score in spatial transcriptomic data of glioblastoma.

Supplementary tables

Supplementary table 1. The potential crosstalk between RCD levels and immune cells, as estimated by CIBERSORT.

Features	Event	Beta	Hr	Lower	Upper	P	Rcdtype	Immunity.type
B.cells.naive_cibersort	OS	-0.73723	0.478436	0.191352	1.196232	0.114853	High.RCD	Adaptive
B.cells.memory_cibersort	OS	-0.29062	0.747803	0.164594	3.397501	0.70669	High.RCD	Adaptive
Plasma.cells_cibersort	OS	-0.38588	0.679853	0.284923	1.622192	0.384484	High.RCD	Adaptive
T.cells.CD8_cibersort	OS	-1.63939	0.194099	0.106192	0.354779	9.96E-08	High.RCD	Adaptive
T.cells.CD4.naive_cibersort	OS	3.476245	32.33807	0.663663	1575.727	0.079568	High.RCD	Adaptive
T.cells.CD4.memory.resting_cibersort	OS	-0.64237	0.526042	0.281087	0.984467	0.044545	High.RCD	Adaptive
T.cells.CD4.memory.activated_cibersort	OS	-0.39781	0.67179	0.061248	7.36849	0.744767	High.RCD	Adaptive
T.cells.follicular.helper_cibersort	OS	-2.85224	0.057715	0.018168	0.183341	1.32E-06	High.RCD	Adaptive
T.cells.regulatory..Tregs._cibersort	OS	-3.89033	0.020439	0.003406	0.122648	2.09E-05	High.RCD	Adaptive
T.cells.gamma.delta_cibersort	OS	-7.96078	0.000349	6.01E-08	2.024412	0.071789	High.RCD	Adaptive
NK.cells.resting_cibersort	OS	4.507766	90.71892	12.71304	647.3605	6.93E-06	High.RCD	Innate
NK.cells.activated_cibersort	OS	-0.33724	0.713738	0.14808	3.440183	0.674293	High.RCD	Innate
Monocytes_cibersort	OS	0.999606	2.717211	0.741465	9.957635	0.131416	High.RCD	Innate
Macrophages.M0_cibersort	OS	0.700911	2.015589	1.299814	3.125524	0.001739	High.RCD	Innate
Macrophages.M1_cibersort	OS	-0.347	0.706804	0.249375	2.003295	0.513868	High.RCD	Innate
Macrophages.M2_cibersort	OS	0.661201	1.937117	1.302765	2.880353	0.001088	High.RCD	Innate
Dendritic.cells.resting_cibersort	OS	-1.91005	0.148073	0.024893	0.880793	0.035774	High.RCD	Innate
Dendritic.cells.activated_cibersort	OS	3.481338	32.50318	9.41615	112.1963	3.64E-08	High.RCD	Innate
Mast.cells.resting_cibersort	OS	-3.72288	0.024164	0.006496	0.089889	2.79E-08	High.RCD	Innate

Mast.cells.activated_cibersort	OS	3.544037	34.60635	14.4315	82.98507	1.99E-15	High.RCD	Innate
Eosinophils_cibersort	OS	8.879517	7183.318	0.44548	1.16E+08	0.072434	High.RCD	Innate
Neutrophils_cibersort	OS	5.815894	335.5912	41.50626	2713.361	4.93E-08	High.RCD	Innate
B.cells.naive_cibersort	OS	-3.22922	0.039588	0.01024	0.153049	2.86E-06	Low.RCD	Adaptive
B.cells.memory_cibersort	OS	1.350837	3.860657	1.143162	13.03811	0.029597	Low.RCD	Adaptive
Plasma.cells_cibersort	OS	-1.98185	0.137814	0.04566	0.415961	0.000438	Low.RCD	Adaptive
T.cells.CD8_cibersort	OS	-2.04862	0.128913	0.06442	0.25797	7.12E-09	Low.RCD	Adaptive
T.cells.CD4.naive_cibersort	OS	-1.15704	0.314415	0.061998	1.59452	0.162493	Low.RCD	Adaptive
T.cells.CD4.memory.resting_cibersort	OS	-0.90413	0.404895	0.201535	0.813454	0.011085	Low.RCD	Adaptive
T.cells.CD4.memory.activated_cibersort	OS	-2.9135	0.054286	0.000152	19.35777	0.331194	Low.RCD	Adaptive
T.cells.follicular.helper_cibersort	OS	-3.99274	0.018449	0.006441	0.052845	1.03E-13	Low.RCD	Adaptive
T.cells.regulatory..Tregs._cibersort	OS	-2.35733	0.094673	0.020433	0.438643	0.002584	Low.RCD	Adaptive
T.cells.gamma.delta_cibersort	OS	-0.73472	0.479641	0.000563	408.5032	0.830996	Low.RCD	Adaptive
NK.cells.resting_cibersort	OS	3.796794	44.5581	8.872237	223.7795	4.01E-06	Low.RCD	Innate
NK.cells.activated_cibersort	OS	-2.2962	0.10064	0.021923	0.461999	0.003147	Low.RCD	Innate
Monocytes_cibersort	OS	1.116199	3.053226	1.271663	7.330708	0.012499	Low.RCD	Innate
Macrophages.M0_cibersort	OS	0.950616	2.587303	1.67947	3.985862	1.62E-05	Low.RCD	Innate
Macrophages.M1_cibersort	OS	-3.6587	0.025766	0.005282	0.12569	6.04E-06	Low.RCD	Innate
Macrophages.M2_cibersort	OS	1.325463	3.763926	2.69918	5.248684	5.59E-15	Low.RCD	Innate
Dendritic.cells.resting_cibersort	OS	-8.91715	0.000134	4.45E-06	0.004036	2.85E-07	Low.RCD	Innate
Dendritic.cells.activated_cibersort	OS	1.98759	7.297923	1.517703	35.09229	0.013114	Low.RCD	Innate
Mast.cells.resting_cibersort	OS	-1.73675	0.176091	0.077432	0.400454	3.43E-05	Low.RCD	Innate

Mast.cells.activated_cibersort	OS	1.999838	7.387862	2.80627	19.44948	5.14E-05	Low.RCD	Innate
Eosinophils_cibersort	OS	11.66076	115932.5	314.513	42733794	0.00011	Low.RCD	Innate
Neutrophils_cibersort	OS	7.818406	2485.94	445.3984	13874.98	5.01E-19	Low.RCD	Innate

Supplementary table 2. The optimally predictive model with 13 published signatures across cohorts.

	**Validation Cohort**	**Testing Cohort**	**NSCLC**	**SKCM**	**Zhao 2019 GBM**	**Synder 2017 UC**	**Hugo 2016 SKCM**	**Jung 2019 NSCLC**	**Van 2015 SKCM**	**Kim 2018 GC**
RCD.Sig	0.719807	0.650949	0.748438	0.773273	0.642857	0.596491	0.577381	0.809211	0.704545	0.85101
ImmmunCells.Sig	0.589027	0.643694	0.623438	0.678571	0.657143	0.692982	0.708333	0.690789	0.675325	0.651515
LRRC15.CAF.Sig	0.56677	0.603237	0.509375	0.59326	0.628571	0.807018	0.60119	0.539474	0.532468	0.54798
NLRP3.Sig	0.56608	0.561384	0.5875	0.617706	0.771429	0.464912	0.39881	0.631579	0.61039	0.575758
IMPRES.Sig	0.555987	0.596401	0.491406	0.636922	0.592857	0.640351	0.821429	0.490132	0.837662	0.54798
Cytotoxic.Sig	0.553313	0.63644	0.66875	0.630533	0.457143	0.526316	0.47619	0.802632	0.685065	0.767677
IMS.Sig	0.552105	0.562221	0.559375	0.58558	0.542857	0.517544	0.488095	0.644737	0.568182	0.482323
CRMA.Sig	0.515442	0.563965	0.467188	0.534323	0.528571	0.710526	0.696429	0.476974	0.62013	0.494949
IPRES.Sig	0.506901	0.559431	0.557813	0.45892	0.314286	0.789474	0.833333	0.578947	0.525974	0.676768
TRS.Sig	0.506556	0.442732	0.45	0.43667	0.371429	0.587719	0.488095	0.473684	0.418831	0.434343
INFG.Sig	0.502243	0.631278	0.635938	0.573944	0.5	0.596491	0.565476	0.697368	0.727273	0.777778
T.cell.inflamed.Sig	0.486888	0.631278	0.685938	0.582663	0.528571	0.54386	0.541667	0.776316	0.717532	0.838384
PDL1.Sig	0.486111	0.629325	0.710938	0.595875	0.457143	0.508772	0.52381	0.703947	0.694805	0.785354
TcellExc.Sig	0.47205	0.519531	0.428125	0.511603	0.485714	0.421053	0.565476	0.473684	0.503247	0.626263

Supplementary table 3. The C-index of the LOOCV framework.

**Model**	**Train**	**Test1**	**Test2**	**Val**	**All**
RSF	0.953627	0.728889	0.722357	0.725623	0.801624
RSF + CoxBoost	0.746953	0.734429	0.714843	0.724636	0.732075

RSF + Enet[α=0.1]	0.747828	0.737831	0.719305	0.728568	0.734988
RSF + Enet[α=0.2]	0.747639	0.737753	0.719221	0.728487	0.734871
RSF + Enet[α=0.3]	0.74734	0.73762	0.719119	0.728369	0.734693
RSF + Enet[α=0.4]	0.747624	0.737611	0.718846	0.728228	0.734694
RSF + Enet[α=0.5]	0.746903	0.737259	0.718843	0.728051	0.734335
RSF + Enet[α=0.6]	0.747013	0.737153	0.718615	0.727884	0.73426
RSF + Enet[α=0.7]	0.747098	0.737145	0.718484	0.727814	0.734242
RSF + Enet[α=0.8]	0.747127	0.737113	0.718444	0.727778	0.734228
RSF + Enet[α=0.9]	0.747169	0.737062	0.718388	0.727725	0.734206
RSF + GBM	0.80167	0.734307	0.721018	0.727662	0.752332
RSF + Lasso	0.74718	0.737067	0.718391	0.727729	0.734213
RSF + plsRcox	0.751779	0.734154	0.71437	0.724262	0.733435
RSF + Ridge	0.748619	0.736886	0.718897	0.727891	0.734801
RSF + StepCox[both]	0.748596	0.730469	0.716833	0.723651	0.731966
RSF + StepCox[backward]	0.748596	0.730469	0.716833	0.723651	0.731966
RSF + StepCox[forward]	0.753228	0.732153	0.716066	0.724109	0.733816
RSF + SuperPC	0.645524	0.641411	0.650866	0.646139	0.645934
RSF + survival-SVM	0.578051	0.596743	0.59096	0.593852	0.588585
Enet[α=0.1]	0.747769	0.737913	0.719272	0.728592	0.734985
Enet[α=0.2]	0.747621	0.737758	0.719298	0.728528	0.734892
Enet[α=0.3]	0.747329	0.737642	0.719141	0.728392	0.734704
Enet[α=0.4]	0.74759	0.737553	0.718929	0.728241	0.73469
Enet[α=0.5]	0.746862	0.737248	0.718902	0.728075	0.734337
Enet[α=0.6]	0.746997	0.737191	0.718692	0.727942	0.734294
Enet[α=0.7]	0.747074	0.737162	0.718578	0.72787	0.734271
Enet[α=0.8]	0.747099	0.737142	0.718441	0.727791	0.734227
Enet[α=0.9]	0.747156	0.737106	0.718421	0.727763	0.734228
StepCox[both]	0.748596	0.730469	0.716833	0.723651	0.731966
StepCox[backward]	0.748596	0.730469	0.716833	0.723651	0.731966
StepCox[forward]	0.753228	0.732153	0.716066	0.724109	0.733816
StepCox[both] + CoxBoost	0.748535	0.731145	0.71697	0.724058	0.732217
StepCox[both] + Enet[α=0.1]	0.748605	0.732784	0.718835	0.72581	0.733408
StepCox[both] + Enet[α=0.2]	0.748607	0.732306	0.718396	0.725351	0.733103
StepCox[both] + Enet[α=0.3]	0.748613	0.732094	0.718174	0.725134	0.73296
StepCox[both] + Enet[α=0.4]	0.748595	0.731876	0.717904	0.72489	0.732792
StepCox[both] + Enet[α=0.5]	0.748602	0.731774	0.71785	0.724812	0.732742

StepCox[both] + Enet[α=0.6]	0.748599	0.73161	0.717719	0.724665	0.732643
StepCox[both] + Enet[α=0.7]	0.7486	0.731555	0.717678	0.724616	0.732611
StepCox[both] + Enet[α=0.8]	0.748593	0.731536	0.717641	0.724588	0.73259
StepCox[both] + Enet[α=0.9]	0.748585	0.731322	0.717479	0.7244	0.732462
StepCox[both] + GBM	0.794687	0.731065	0.723401	0.727233	0.749718
StepCox[both] + Lasso	0.748594	0.731536	0.717655	0.724596	0.732595
StepCox[both] + plsRcox	0.747368	0.731458	0.717584	0.724521	0.732137
StepCox[both] + Ridge	0.748633	0.733477	0.719945	0.726711	0.734019
StepCox[both] + RSF	0.951387	0.732576	0.725469	0.729022	0.803144
StepCox[both] + SuperPC	0.644517	0.640903	0.654787	0.647845	0.646736
StepCox[both] + survival-SVM	0.568792	0.588994	0.585316	0.587155	0.581034
StepCox[backward] + CoxBoost	0.748535	0.731145	0.71697	0.724058	0.732217
StepCox[backward] + Enet[α=0.1]	0.748605	0.732784	0.718835	0.72581	0.733408
StepCox[backward] + Enet[α=0.2]	0.748607	0.732306	0.718396	0.725351	0.733103
StepCox[backward] + Enet[α=0.3]	0.748613	0.732094	0.718174	0.725134	0.73296
StepCox[backward] + Enet[α=0.4]	0.748595	0.731876	0.717904	0.72489	0.732792
StepCox[backward] + Enet[α=0.5]	0.748602	0.731774	0.71785	0.724812	0.732742
StepCox[backward] + Enet[α=0.6]	0.748599	0.73161	0.717719	0.724665	0.732643
StepCox[backward] + Enet[α=0.7]	0.7486	0.731555	0.717678	0.724616	0.732611
StepCox[backward] + Enet[α=0.8]	0.748593	0.731536	0.717641	0.724588	0.73259
StepCox[backward] + Enet[α=0.9]	0.748585	0.731322	0.717479	0.7244	0.732462
StepCox[backward] + GBM	0.794687	0.731065	0.723401	0.727233	0.749718
StepCox[backward] + Lasso	0.748594	0.731536	0.717655	0.724596	0.732595
StepCox[backward] + plsRcox	0.747368	0.731458	0.717584	0.724521	0.732137

StepCox[backward] + Ridge	0.748633	0.733477	0.719945	0.726711	0.734019
StepCox[backward] + RSF	0.951387	0.732576	0.725469	0.729022	0.803144
StepCox[backward] + SuperPC	0.644517	0.640903	0.654787	0.647845	0.646736
StepCox[backward] + survival-SVM	0.568792	0.588994	0.585316	0.587155	0.581034
StepCox[forward] + CoxBoost	0.746953	0.734429	0.714843	0.724636	0.732075
StepCox[forward] + Enet[α=0.1]	0.747769	0.737913	0.719272	0.728592	0.734985
StepCox[forward] + Enet[α=0.2]	0.747621	0.737758	0.719298	0.728528	0.734892
StepCox[forward] + Enet[α=0.3]	0.747329	0.737642	0.719141	0.728392	0.734704
StepCox[forward] + Enet[α=0.4]	0.74759	0.737553	0.718929	0.728241	0.73469
StepCox[forward] + Enet[α=0.5]	0.746862	0.737248	0.718902	0.728075	0.734337
StepCox[forward] + Enet[α=0.6]	0.746997	0.737191	0.718692	0.727942	0.734294
StepCox[forward] + Enet[α=0.7]	0.747074	0.737162	0.718578	0.72787	0.734271
StepCox[forward] + Enet[α=0.8]	0.747099	0.737142	0.718441	0.727791	0.734227
StepCox[forward] + Enet[α=0.9]	0.747156	0.737106	0.718421	0.727763	0.734228
StepCox[forward] + GBM	0.80167	0.734307	0.721018	0.727662	0.752332
StepCox[forward] + Lasso	0.747199	0.737071	0.718371	0.727721	0.734214
StepCox[forward] + plsRcox	0.751779	0.734154	0.71437	0.724262	0.733435
StepCox[forward] + Ridge	0.748588	0.737066	0.718962	0.728014	0.734872
StepCox[forward] + RSF	0.953627	0.728889	0.722357	0.725623	0.801624
StepCox[forward] + SuperPC	0.645524	0.641411	0.650866	0.646139	0.645934
StepCox[forward] + survival-SVM	0.57805	0.596741	0.590958	0.59385	0.588583
CoxBoost	0.746953	0.734429	0.714843	0.724636	0.732075
CoxBoost + Enet[α=0.1]	0.751039	0.736962	0.717733	0.727348	0.735245

CoxBoost + Enet[α=0.2]	0.751411	0.736381	0.717222	0.726801	0.735004
CoxBoost + Enet[α=0.3]	0.751571	0.735826	0.716681	0.726253	0.734693
CoxBoost + Enet[α=0.4]	0.751673	0.735584	0.716394	0.725989	0.73455
CoxBoost + Enet[α=0.5]	0.751791	0.735323	0.71616	0.725742	0.734425
CoxBoost + Enet[α=0.6]	0.751816	0.735294	0.716153	0.725724	0.734421
CoxBoost + Enet[α=0.7]	0.75188	0.735059	0.715975	0.725517	0.734305
CoxBoost + Enet[α=0.8]	0.751895	0.735055	0.715972	0.725513	0.734307
CoxBoost + Enet[α=0.9]	0.751836	0.735188	0.716109	0.725648	0.734377
CoxBoost + GBM	0.799701	0.734009	0.720179	0.727094	0.751296
CoxBoost + Lasso	0.751888	0.735027	0.71595	0.725488	0.734288
CoxBoost + plsRcox	0.749403	0.733618	0.713117	0.723367	0.732046
CoxBoost + Ridge	0.749863	0.737502	0.718304	0.727903	0.735223
CoxBoost + StepCox[both]	0.748596	0.730469	0.716833	0.723651	0.731966
CoxBoost + StepCox[backward]	0.748596	0.730469	0.716833	0.723651	0.731966
CoxBoost + StepCox[forward]	0.751988	0.733511	0.714572	0.724041	0.733357
CoxBoost + SuperPC	0.645302	0.641328	0.649932	0.64563	0.645521
CoxBoost + survival-SVM	0.578342	0.598845	0.590649	0.594747	0.589278
plsRcox	0.751779	0.734154	0.71437	0.724262	0.733435
SuperPC	0.645524	0.641411	0.650866	0.646139	0.645934
GBM	0.80167	0.734307	0.721018	0.727662	0.752332
survival - SVM	0.57805	0.596741	0.590958	0.59385	0.588583
Ridge	0.753233	0.735494	0.717556	0.726525	0.735428
Lasso	0.747199	0.737071	0.718371	0.727721	0.734214
Lasso + CoxBoost	0.750735	0.735529	0.713974	0.724751	0.733413
Lasso + GBM	0.799714	0.733558	0.721002	0.72728	0.751425
Lasso + plsRcox	0.748888	0.734159	0.714002	0.72408	0.732349
Lasso + RSF	0.952687	0.732174	0.724007	0.72809	0.802956
Lasso + StepCox[both]	0.748596	0.730469	0.716833	0.723651	0.731966
Lasso + StepCox[backward]	0.748596	0.730469	0.716833	0.723651	0.731966
Lasso + StepCox[forward]	0.751109	0.734617	0.713863	0.72424	0.733196

Lasso + SuperPC	0.645302	0.641328	0.649932	0.64563	0.645521
Lasso + survival-SVM	0.578864	0.598267	0.590251	0.594259	0.589127

Supplementary table 4. The results of the univariate cox regression and the K-M log rank test (cut point: median).

**Univariate cox regression**								**K-M log rank test (cutpoint:median)**					
tumor	event	beta	hr	lower	upper	p		tumor	event	hr	lower	upper	p
ACC	OS	0.057977	1.059691	1.042321	1.07735	6.17E-12		ACC	OS	9.337648	4.371337	19.94622	3.03E-07
ACC	DSS	0.059912	1.061743	1.043925	1.079865	3.97E-12		ACC	DSS	11.51555	4.260279	20.46619	3.24E-07
ACC	DFI	0.048063	1.049237	1.005712	1.094646	0.026186		ACC	DFI	3.19105	2.479349	35.16717	0.042031
ACC	PFI	0.036085	1.036744	1.048872	1.048872	1.19E-09		ACC	PFI	5.185245	4.960481	17.57726	2.22E-07
BLCA	OS	0.0456	1.046655	1.040196	1.053155	3.04E-47		BLCA	OS	4.663919	3.453363	6.29883	0
BLCA	DSS	0.047611	1.048763	1.040843	1.056743	7.95E-35		BLCA	DSS	5.170541	3.237295	6.719235	0
BLCA	DFI	0.027715	1.028103	1.013552	1.042862	0.000138		BLCA	DFI	2.536511	2.110273	10.30774	0.007056
BLCA	PFI	0.030308	1.030772	1.036621	1.036621	8.80E-26		BLCA	PFI	2.941685	3.436284	6.330136	7.45E-13
BRCA	OS	0.044988	1.046016	1.039055	1.053023	8.06E-40		BRCA	OS	4.329891	3.138736	5.973092	0
BRCA	DSS	0.050401	1.051693	1.042761	1.060701	5.07E-31		BRCA	DSS	4.406622	2.809817	6.672306	9.27E-11
BRCA	DFI	0.031437	1.031936	1.021579	1.042398	1.01E-09		BRCA	DFI	1.981419	2.79946	6.696992	0.001495
BRCA	PFI	0.035287	1.035917	1.043189	1.043189	4.79E-23		BRCA	PFI	2.171053	3.120911	6.007206	2.39E-06
CESC	OS	0.056257	1.05787	1.046891	1.068964	4.14E-26		CESC	OS	7.344216	4.587718	11.75694	1.01E-13
CESC	DSS	0.058179	1.059905	1.047575	1.07238	1.94E-22		CESC	DSS	8.507901	4.289906	12.57312	1.42E-11
CESC	DFI	0.02304	1.023307	1.004035	1.042949	0.017544		CESC	DFI	1.635071	3.269773	16.49579	0.206331
CESC	PFI	0.039858	1.040663	1.050712	1.050712	4.34E-16		CESC	PFI	3.345709	4.599765	11.72614	7.06E-07
CHOL	OS	0.039309	1.040092	1.017835	1.062835	0.000368		CHOL	OS	4.174637	1.61458	10.79388	0.002218
CHOL	DSS	0.037717	1.038437	1.015423	1.061973	0.000972		CHOL	DSS	3.794673	1.510462	11.53792	0.005846
CHOL	DFI	0.037997	1.038728	1.012346	1.065798	0.003795		CHOL	DFI	6.427749	1.027263	16.96507	0.00157

CHOL	PFI	0.030237	1.030699	1.049733	1.049733	0.001201		CHOL	PFI	2.785503	1.665612	10.46318	0.016298
COAD	OS	0.046092	1.04717	1.039418	1.054981	5.24E-34		COAD	OS	6.291063	4.215511	9.388535	1.01E-14
COAD	DSS	0.049574	1.050823	1.041093	1.060643	1.54E-25		COAD	DSS	8.007961	3.763983	10.51479	6.60E-11
COAD	DFI	0.01583	1.015956	0.994489	1.037885	0.146276		COAD	DFI	1.185047	2.673132	14.80566	0.688679
COAD	PFI	0.03684	1.037527	1.045175	1.045175	8.24E-23		COAD	PFI	3.63727	4.359023	9.079437	1.95E-11
ESCA	OS	0.035764	1.036412	1.02677	1.046144	6.40E-14		ESCA	OS	3.3058	2.112852	5.172306	3.33E-07
ESCA	DSS	0.042259	1.043165	1.030905	1.055571	2.45E-12		ESCA	DSS	4.521529	1.907425	5.729355	3.88E-07
ESCA	DFI	-0.00528	0.994734	0.976541	1.013267	0.575096		ESCA	DFI	0.855311	1.447695	7.54877	0.713708
ESCA	PFI	0.017976	1.018139	1.026772	1.026772	3.01E-05		ESCA	PFI	1.582835	2.170643	5.034598	0.029913
GBM	OS	0.012506	1.012585	1.006509	1.018697	4.64E-05		GBM	OS	1.530462	1.080943	2.166918	0.0121
GBM	DSS	0.013569	1.013661	1.006988	1.020379	5.66E-05		GBM	DSS	1.590227	1.059152	2.211498	0.010217
GBM	DFI	NA	NA	NA	NA	NA		GBM	DFI	NA	NA	NA	NA
GBM	PFI	0.011946	1.012017	1.018043	1.018043	8.01E-05		GBM	PFI	1.456353	1.082911	2.162978	0.028091
HNSC	OS	0.043953	1.044934	1.039398	1.050499	3.70E-59		HNSC	OS	5.229774	3.984369	6.864459	0
HNSC	DSS	0.048082	1.049257	1.042027	1.056537	2.66E-42		HNSC	DSS	6.479818	3.677623	7.437014	0
HNSC	DFI	0.024216	1.024512	1.010344	1.038878	0.000654		HNSC	DFI	1.928064	2.348018	11.64835	0.074517
HNSC	PFI	0.030627	1.031101	1.036403	1.036403	1.22E-31		HNSC	PFI	3.568662	3.928627	6.961856	0
KICH	OS	0.110619	1.11697	1.06473	1.171772	6.00E-06		KICH	OS	10.69141	2.854904	40.03859	0.005035
KICH	DSS	0.108127	1.114189	1.058371	1.172951	3.73E-05		KICH	DSS	8.378225	2.380129	48.02526	0.017848
KICH	DFI	0.06385	1.065932	0.96435	1.178215	0.211463		KICH	DFI	5.659574	0.813188	140.5656	0.106982
KICH	PFI	0.054966	1.056504	1.081775	1.081775	5.18E-06		KICH	PFI	6.333523	3.222761	35.46844	0.006351
KIRC	OS	0.047322	1.04846	1.042846	1.054104	7.29E-67		KIRC	OS	7.161696	5.294575	9.687253	0
KIRC	DSS	0.050838	1.052153	1.045211	1.05914	3.28E-51		KIRC	DSS	13.03032	4.886523	10.49619	0
KIRC	DFI	0.009397	1.009441	0.976101	1.043921	0.583437		KIRC	DFI	2.455601	2.267991	22.61468	0.071734

KIRC	PFI	0.036712	1.037394	1.043198	1.043198	4.63E-38		KIRC	PFI	4.078163	5.21993	9.825781	0
KIRP	OS	0.05395	1.055432	1.044662	1.066313	6.39E-25		KIRP	OS	7.075438	3.893797	12.85681	2.80E-08
KIRP	DSS	0.05573	1.057312	1.045379	1.069381	6.36E-22		KIRP	DSS	34.23273	3.355531	14.9192	2.31E-08
KIRP	DFI	0.035373	1.036006	1.01994	1.052326	9.17E-06		KIRP	DFI	2.221221	3.354271	14.9248	0.028664
KIRP	PFI	0.033366	1.033929	1.041883	1.041883	1.43E-17		KIRP	PFI	3.002452	4.204185	11.90762	4.26E-05
LGG	OS	0.037822	1.038546	1.03353	1.043587	6.59E-53		LGG	OS	6.339803	4.450699	9.030741	0
LGG	DSS	0.037841	1.038566	1.033402	1.043755	4.44E-50		LGG	DSS	6.380016	4.368773	9.200091	0
LGG	DFI	0.031471	1.031971	1.013954	1.050308	0.000462		LGG	DFI	1.640633	2.703704	14.86594	0.228903
LGG	PFI	0.023826	1.024112	1.027663	1.027663	1.78E-41		LGG	PFI	2.836148	4.785892	8.398248	2.30E-14
LIHC	OS	0.043662	1.044629	1.037821	1.051481	3.83E-39		LIHC	OS	4.577863	3.223486	6.501293	1.11E-16
LIHC	DSS	0.046047	1.047124	1.037719	1.056614	1.47E-23		LIHC	DSS	4.547901	2.904966	7.214139	3.88E-11
LIHC	DFI	0.017212	1.017361	1.010631	1.024135	3.71E-07		LIHC	DFI	1.651653	3.273835	6.401309	0.002025
LIHC	PFI	0.017982	1.018145	1.024243	1.024243	3.58E-09		LIHC	PFI	1.706737	3.399524	6.164636	0.00024
LUAD	OS	0.043339	1.044292	1.038505	1.050111	9.35E-53		LUAD	OS	4.421304	3.286978	5.947082	0
LUAD	DSS	0.047054	1.048179	1.04047	1.055944	8.06E-36		LUAD	DSS	5.003429	3.034102	6.442739	1.89E-15
LUAD	DFI	0.029035	1.02946	1.020847	1.038146	1.26E-11		LUAD	DFI	3.043541	2.867988	6.815902	5.99E-08
LUAD	PFI	0.024599	1.024904	1.029925	1.029925	5.88E-23		LUAD	PFI	2.453805	3.351933	5.831837	5.79E-11
LUSC	OS	0.03771	1.03843	1.03276	1.044131	1.57E-41		LUSC	OS	4.020901	3.051961	5.297461	0
LUSC	DSS	0.046442	1.047538	1.038028	1.057135	1.85E-23		LUSC	DSS	5.22311	2.586336	6.251176	2.05E-14
LUSC	DFI	0.027856	1.028248	1.016682	1.039944	1.39E-06		LUSC	DFI	2.181033	2.278249	7.096523	0.001931
LUSC	PFI	0.025698	1.026031	1.032685	1.032685	6.62E-15		LUSC	PFI	2.430763	2.86922	5.634856	4.09E-08
MESO	OS	0.034582	1.035187	1.024407	1.04608	9.48E-11		MESO	OS	3.098048	1.881719	5.100602	1.24E-07
MESO	DSS	0.03874	1.0395	1.024557	1.054661	1.57E-07		MESO	DSS	3.162288	1.598315	6.005011	4.01E-05

MESO	DFI	0.01156	1.011627	0.97682	1.047674	0.517561		MESO	DFI	1.633321	0.628494	15.27126	0.490654
MESO	PFI	0.014273	1.014375	1.02466	1.02466	0.005555		MESO	PFI	1.943197	1.802227	5.325576	0.007382
OV	OS	0.052445	1.053844	1.044706	1.063063	3.80E-32		OV	OS	3.101649	2.298184	4.186011	8.88E-16
OV	DSS	0.052122	1.053505	1.043605	1.063498	2.76E-27		OV	DSS	3.182679	2.230504	4.313028	3.54E-14
OV	DFI	0.025266	1.025588	1.013392	1.037932	3.48E-05		OV	DFI	2.013978	2.048238	4.696829	0.000258
OV	PFI	0.024558	1.024862	1.031845	1.031845	1.37E-12		OV	PFI	2.083136	2.343646	4.104812	2.12E-08
PAAD	OS	0.033547	1.034116	1.026782	1.041502	2.51E-20		PAAD	OS	5.782914	3.797965	8.805266	2.22E-16
PAAD	DSS	0.036387	1.037057	1.0287	1.045481	1.20E-18		PAAD	DSS	7.557756	3.58375	9.331592	2.22E-16
PAAD	DFI	0.041357	1.042224	1.027567	1.05709	1.05E-08		PAAD	DFI	7.001339	2.004203	16.68598	3.14E-08
PAAD	PFI	0.027426	1.027805	1.034308	1.034308	1.56E-17		PAAD	PFI	3.618053	3.868614	8.644464	5.09E-12
PRAD	OS	0.14131	1.151782	1.088665	1.218558	8.91E-07		PRAD	OS	4.099757	1.18678	14.1627	0.049799
PRAD	DSS	0.165659	1.180171	1.071876	1.299407	0.000742		PRAD	DSS	4.240973	0.709732	23.68218	0.154527
PRAD	DFI	0.038602	1.039356	0.990932	1.090147	0.112795		PRAD	DFI	1.624796	2.012567	8.35153	0.174223
PRAD	PFI	0.033491	1.034058	1.052097	1.052097	0.000147		PRAD	PFI	1.431357	2.731444	6.153524	0.08115
READ	OS	0.072946	1.075672	1.053967	1.097825	2.32E-12		READ	OS	16.15626	7.151556	36.49901	2.50E-07
READ	DSS	0.068894	1.071323	1.043982	1.099381	1.76E-07		READ	DSS	8.364196	5.018188	52.01574	0.000867
READ	DFI	0.035352	1.035984	0.982362	1.092533	0.192332		READ	DFI	1.13176	2.848065	91.64985	0.881687
READ	PFI	0.043105	1.044047	1.06147	1.06147	3.31E-07		READ	PFI	2.065262	8.154721	32.00903	0.031187
SARC	OS	0.040749	1.041591	1.033593	1.04965	3.71E-25		SARC	OS	4.681251	3.131548	6.997852	2.09E-13
SARC	DSS	0.040674	1.041512	1.0327	1.0504	6.49E-21		SARC	DSS	4.407451	3.007896	7.285527	8.68E-11
SARC	DFI	0.020953	1.021174	1.011996	1.030435	5.40E-06		SARC	DFI	2.199817	2.835862	7.727493	0.000722
SARC	PFI	0.025846	1.026183	1.032668	1.032668	8.99E-16		SARC	PFI	2.360023	3.343411	6.554416	1.76E-07
SKCM	OS	0.037986	1.038717	1.032932	1.044535	1.54E-40		SKCM	OS	3.587313	2.713666	4.742225	0

SKCM	DSS	0.036833	1.03752	1.031347	1.043729	1.08E-33		SKCM	DSS	3.435138	2.664061	4.830524	0
SKCM	DFI	NA	NA	NA	NA	NA		SKCM	DFI	NA	NA	NA	NA
SKCM	PFI	0.019206	1.019392	1.024159	1.024159	7.14E-16		SKCM	PFI	1.804953	2.849723	4.515813	7.38E-08
STAD	OS	0.043787	1.04476	1.038271	1.051288	3.53E-43		STAD	OS	5.260503	3.829401	7.226429	0
STAD	DSS	0.051344	1.052685	1.04373	1.061718	5.05E-32		STAD	DSS	6.590015	3.486124	7.938012	3.33E-16
STAD	DFI	0.043858	1.044834	1.031936	1.057893	4.51E-12		STAD	DFI	3.502883	2.810443	9.846454	1.83E-05
STAD	PFI	0.031987	1.032504	1.039419	1.039419	5.87E-21		STAD	PFI	3.230016	3.717381	7.444192	2.93E-11
TGCT	OS	0.153114	1.165458	1.050616	1.292853	0.003818		TGCT	OS	3.347053	0.470095	23.83085	0.265979
TGCT	DSS	0.253656	1.288729	0.981435	1.692238	0.067981		TGCT	DSS	Inf	0.346065	32.37189	0.062173
TGCT	DFI	0.00625	1.00627	0.978561	1.034763	0.660868		TGCT	DFI	1.276691	1.654126	6.772619	0.484607
TGCT	PFI	0.030084	1.030541	1.054103	1.054103	0.0091		TGCT	PFI	1.463102	1.796385	6.236282	0.224079
THCA	OS	0.081151	1.084534	1.060659	1.108947	8.99E-13		THCA	OS	7.667555	2.874847	20.45028	0.001437
THCA	DSS	0.10388	1.109467	1.069481	1.150948	2.91E-08		THCA	DSS	Inf	1.73875	33.81246	0.005174
THCA	DFI	0.034296	1.034891	1.006279	1.064317	0.016504		THCA	DFI	2.791054	3.597175	16.34377	0.010892
THCA	PFI	0.038705	1.039464	1.0552	1.0552	4.44E-07		THCA	PFI	2.271059	4.492843	13.08557	0.003449
THYM	OS	0.090299	1.094501	1.050223	1.140647	1.82E-05		THYM	OS	Inf	Inf	Inf	0.00318
THYM	DSS	0.123009	1.130895	1.032405	1.238781	0.008146		THYM	DSS	Inf	Inf	Inf	0.056411
THYM	DFI	NA	NA	NA	NA	NA		THYM	DFI	NA	NA	NA	NA
THYM	PFI	0.034863	1.035478	1.0568	1.0568	0.000801		THYM	PFI	1.796646	Inf	Inf	0.184469
UCEC	OS	0.05724	1.05891	1.049659	1.068242	1.97E-37		UCEC	OS	8.479259	5.539962	12.97804	0
UCEC	DSS	0.059528	1.061335	1.050029	1.072763	1.23E-27		UCEC	DSS	19.72489	5.047854	14.24325	2.22E-16
UCEC	DFI	0.026763	1.027124	1.015206	1.039182	6.98E-06		UCEC	DFI	2.895532	4.914638	14.62932	6.71E-05
UCEC	PFI	0.034656	1.035264	1.042839	1.042839	1.19E-20		UCEC	PFI	4.024986	5.901243	12.18351	1.67E-13
UCS	OS	0.044324	1.045321	1.027367	1.063589	5.32E-07		UCS	OS	3.471809	1.737834	6.935909	9.29E-05
UCS	DSS	0.040985	1.041837	1.023621	1.060377	5.26E-06		UCS	DSS	3.171849	1.670641	7.214871	0.000695

UCS	DFI	0.056255	1.057868	1.010349	1.107621	0.016438		UCS	DFI	2.980268	0.698433	17.25787	0.064698
UCS	PFI	0.032438	1.03297	1.048843	1.048843	3.05E-05		UCS	PFI	2.214417	1.782611	6.761688	0.011402
UVM	OS	0.07707	1.080118	1.052587	1.108369	4.90E-09		UVM	OS	6.39964	2.800462	14.62451	9.00E-05
UVM	DSS	0.076295	1.079281	1.050904	1.108425	2.00E-08		UVM	DSS	7.72498	2.702038	15.15722	9.67E-05
UVM	DFI	NA	NA	NA	NA	NA		UVM	DFI	NA	NA	NA	NA
UVM	PFI	0.048639	1.049841	1.069291	1.069291	2.07E-07		UVM	PFI	4.571888	3.00458	13.63098	2.37E-05

Supplementary table 5. The results of the univariate cox regression and the K-M log rank test (cut point: median).

	**HR**	**Z**	**Pvalue**	**Lower**	**Upper**
TCGA_train	39.9932	34.47547	1.87E-260	32.42737	49.32426
TCGA_test1	3.61334	16.49094	4.26E-61	3.101707	4.209367
TCGA_test2	3.710495	13.93556	3.85E-44	3.085632	4.461896
TCGA_total	9.62947	43.06635	0	8.68637	10.67496
LGG_TCGA_hseq	2.55947	4.990631	6.02E-07	1.769529	3.702049
LGG_cgga693	1.760656	3.878033	0.000105	1.322849	2.343359
LGG_cgga325	1.875768	2.925316	0.003441	1.230691	2.858968
LGG_gse108474	1.392233	1.686421	0.091715	0.947741	2.045192
LGG_gse16011	2.270181	3.822947	0.000132	1.491125	3.456265
LGG_cgga	1.871617	5.168919	2.35E-07	1.475696	2.37376
GBM_cgga	1.294072	2.30106	0.021388	1.038953	1.611836
GBM_cgga693	1.242792	1.521796	0.12806	0.939334	1.644285
GBM_cgga325	1.319708	1.532866	0.125309	0.925618	1.881585
GBM_gse108474	1.425349	1.742111	0.081489	0.956648	2.123686
GBM_TCGA_array	1.125631	1.235409	0.216678	0.932947	1.35811
Glioma_cgga	2.506692	10.90983	1.03E-27	2.125211	2.956651
Glioma_tcga.hiseq	6.252328	12.20995	2.75E-34	4.658646	8.391196
Glioma_gse108474	1.478722	2.776447	0.005496	1.121914	1.94901
Glioma_cgga325	3.117612	7.840452	4.49E-15	2.346259	4.142555
Glioma_cgga693	2.092054	7.1203	1.08E-12	1.707382	2.563392
LIHC_ICGA	1.826885	1.921492	0.05467	0.988007	3.37802
LIHC_GSE76427	0.938773	-0.14993	0.88082	0.411012	2.144206
STAD_GSE15459	1.490636	1.940434	0.052327	0.99599	2.230942

Supplementary table 6. Collection of 18 regulated cell death signatures.

**Rank**	**RCD**	**Collection strategy**	**Reference**	CD: cell death
1	Apoptosis	"APOPTOSIS" in MsigDB, PMID:36341760	PMID:36341760	
2	Ferroptosis	"Ferroptosis" in MsigDB, FerrDb (zhounan.org), PMID:36341760,		
3	Pyroptosis	"Pyroptosis" in MsigDB, PMID: 36341760		

4	Necroptosis	“Necroptosis” in MsigDB, PMID:, 36341760		
5	Cuproptosis	“Copper” in MsigDB, PMID:35298263	PMID:35298263	
6	Autophagy	“Autophagy” in MsigDB, PMID: 36341760		
7	Parthanatos	PMID:34907160; 36341760	PMID:34907160	
8	Autosis	PMID:34907160; 36341760		
9	Immunogenic CD	PMID:34907160, 27057433	PMID:27057433	
10	Entotic CD	PMID: 36341760		
11	Netotic CD	PMID: 36341760		
12	Mitotic cd	PMID: 29362479	PMID: 29362479	
13	Lysosome-dependent CD	PMID: 36341760, 29362479		
14	Alkaliptosis	PMID: 34331036, 36341760	PMID: 34331036	
15	Oxeiptosis	PMID: 29255269, 36341760	PMID: 29255269	
16	MPT-driven necrosis	PMID: 36341760		
17	Anoikis	“Anoikis” in MsigDB	“Anoikis” in MsigDB	
18	Disulfidptosis	PMID: 36747082, 36918690	PMID: 36747082	36918690

Supplementary table 7. Summary of the objective respond rate.

Cancer.Type	ORR
ACC	0.062
BRCA	0.057
CESC	0.2
COAD_MSI	0.39
COAD_MSS	0
UCEC	0.13
ESCA	0.11
GBM	0.087
HNSC	0.15
LIHC	0.18
SKCM	0.37
MESO	0.13
UVM	0.036
LUAD	0.17
LUSC	0.17
OV	0.099
PAAD	0
PRAD	0.075
KIRC	0.24
SARC	0.093
BLCA	0.18

Supplementary table 8. 82 sc-RNA datasets for 29 cancer types.

DatasetName	Species	Treatment	Patients	Cells	Plateform	PriMeta	PMID	**Reference**

BCC_GSE123813_aPD1	Human	Immunotherapy	11	52884	10x.Genomics	Metastatic	31359002	31359002
BRCA_GSE143423	Human	None	2	4375	10x.Genomics	Metastatic	None	None
BRCA_GSE176078	Human	None	26	89471	10x.Genomics	Primary	34493872	34493872
BRCA_SRP114962	Human	Chemotherapy	8	2472	SNRS	Primary	29681456	29681456
CHOL_GSE125449_aPD1aPDL1aCTLA4	Human	Immunotherapy	10	5761	10x.Genomics	Primary	31588021	31588021
CRC_GSE146771_Smartseq2	Human	None	10	10468	Smart.seq2	Primary	32302573	32302573
Glioma_GSE102130	Human	None	6	3321	Smart.seq2	Primary	29674595	29674595
Glioma_GSE103224	Human	None	8	17185	Microwell	Primary	30041684	30041684
Glioma_GSE131928_10X	Human	None	9	13553	10x.Genomics	Primary	31327527	31327527
Glioma_GSE131928_Smartseq2	Human	None	28	7930	Smart.seq2	Primary	31327527	
Glioma_GSE138794	Human	None	9	18458	10x.Genomics	Primary	31554641	31554641
Glioma_GSE141982	Human	None	2	5263	10x.Genomics	Primary	32105316	32105316
Glioma_GSE148842	Human	None	7	111397	Microwell	Primary	33975634	33975634
Glioma_GSE70630	Human	None	6	4347	Smart.seq2	Primary	27806376	27806376
Glioma_GSE84465	Human	None	4	3533	Smart.seq2	Primary	29091775	29091775
Glioma_GSE89567	Human	None	10	6341	Smart.seq2	Primary	28360267	28360267
HNSC_GSE103322	Human	None	18	5902	Smart.seq2	Primary	29198524	29198524
LIHC_GSE125449_aPDL1aCTLA4	Human	Immunotherapy	9	3834	10x.Genomics	Primary	31588021	31588021
MB_GSE119926	Human	None	25	7745	Smart.seq2	Primary.Metastatic	31341285	31341285
MCC_GSE117988_aPD1aCTLA4	Human	Immunotherapy	1	10134	10x.Genomics	Metastatic	30250229	30250229
MCC_GSE118056_aPDL1	Human	Immunotherapy	1	11024	10x.Genomics	Primary	30250229	
NET_GSE140312	Human	None	1	3158	10x.Genomics	Primary.Metastatic	32054662	32054662

NSCLC_EMTAB6149	Human	None	5	40218	10x.Genomics	Primary	29988129	29988129
NSCLC_GSE117570	Human	None	4	11453	10x.Genomics	Primary	31033233	31033233
NSCLC_GSE127465	Human	None	7	31179	Smart.seq2	Primary	30979687	30979687
NSCLC_GSE143423	Human	None	3	12193	10x.Genomics	Metastatic	None	None
OV_GSE118828	Human	None	9	1909	Smart.seq2	Primary.Metastatic	30383866	30383866
PAAD_CRA001160	Human	None	35	57443	10x.Genomics	Primary	31273297	31273297
PAAD_GSE111672	Human	None	3	6122	inDrop	Primary	31932730	31932730
SKCM_GSE115978_aPD1	Human	Immunotherapy	31	7186	Smart.seq2	Primary.Metastatic	30388455	30388455
SKCM_GSE72056	Human	None	19	4645	Smart.seq2	Metastatic	27124452	27124452
STAD_GSE134520	Human	None	13	41554	10x.Genomics	Primary	31067475	31067475
UVM_GSE139829	Human	None	11	103703	10x.Genomics	Primary.Metastatic	31980621	31980621
BCC_GSE141526	Human	None	6	56116	10x.Genomics	Primary	33033234	33033234
BRCA_EMTAB8107	Human	None	14	33043	10x.Genomics	Primary	32561858	32561858
BRCA_GSE148673	Human	None	6	10359	10x.Genomics	Primary	33462507	33462507
BRCA_GSE150660	Human	None	3	10605	10x.Genomics	Metastatic	32675368	32675368
BRCA_GSE161529	Human	None	52	332168	10x.Genomics	Primary	33950524	33950524
CESC_GSE168652	Human	None	1	22998	10x.Genomics	Primary	33996252	33996252
CHOL_GSE138709	Human	None	5	33990	10x.Genomics	Primary	32505533	32505533
CHOL_GSE142784	Human	None	2	10535	10x.Genomics	Primary	32505533	
CRC_EMTAB8107	Human	None	7	23176	10x.Genomics	Primary	32561858	32561858
CRC_GSE166555	Human	None	12	66050	10x.Genomics	Primary	34409732	34409732
ESCA_GSE160269	Human	None	64	208658	10x.Genomics	Primary	34489433	34489433

Glioma_GSE141383	Human	Immunotherapy	6	10502	Microwell.seq	Primary	34011400	34011400
Glioma_GSE141460	Human	None	24	6385	Smart.seq2	Primary	32663469	32663469
NPC_GSE150430	Human	None	15	45959	10x.Genomics	Primary	32686767	32686767
NPC_GSE162025	Human	None	10	176440	10x.Genomics	Primary	33531485	33531485
OSCC_GSE172577	Human	None	6	57503	10x.Genomics	Primary	34044317	34044317
KICH_GSE159115	Human	None	1	2850	10x.Genomics	Primary	34099557	34099557
KIRC_GSE159115	Human	None	8	27669	10x.Genomics	Primary	34099557	
KIRC_GSE171306	Human	None	2	11427	10x.Genomics	Primary	34168986	34168986
LIHC_GSE146115	Human	None	4	3134	C1	Primary	33531041	33531041
LIHC_GSE146409	Human	None	6	2916	MARS.seq	Primary.Metastatic	33332768	33332768
LIHC_GSE166635	Human	None	2	22631	10x.Genomics	Primary	33619115	33619115
HB_GSE180665	Human	None	3	46486	10x.Genomics	Primary	34497364	34497364
MB_GSE155446	Human	None	28	37445	10x.Genomics	Primary	34077540	34077540
NSCLC_GSE148071	Human	None	42	82267	GEXSCOPETM	Primary	33953163	33953163
NSCLC_GSE150660	Human	None	2	5407	10x.Genomics	Metastatic	32675368	32675368
OV_EMTAB8107	Human	None	5	24781	10x.Genomics	Primary	32561858	32561858
OV_GSE130000	Human	None	8	11215	Drop.seq	Primary.Metastatic	34992217	34992217
OV_GSE147082	Human	Immunotherapy	6	9796	Drop.seq	Metastatic	34038734	34038734
OV_GSE151214	Human	None	8	59446	10x.Genomics	Metastatic	33852846	33852846
OV_GSE154600	Human	None	5	42583	10x.Genomics	Primary	32747365	32747365
OV_GSE158722	Human	Chemotherapy	21	96846	10x.Genomics	Primary	34031395	34031395
PAAD_GSE141017	Human	None	1	4934	10x.Genomics	Primary	33649045	33649045

PAAD_GSE148673	Human	None	2	6196	10x.Genomics	Primary	33462507	33462507
PAAD_GSE154778	Human	None	16	14953	10x.Genomics	Primary.Metastatic	32988401	32988401
PAAD_GSE162708	Human	None	1	22133	10x.Genomics	Primary.Metastatic	34671197	34671197
PAAD_GSE165399	Human	None	3	9569	GEXSCOPETM	Primary	34326696	34326696
PRAD_GSE176031	Human	None	11	18807	Drop.seq	Primary	35013146	35013146
PRAD_GSE137829	Human	None	6	8640	10x.Genomics	Primary	33328604	33328604
PRAD_GSE141445	Human	None	13	33441	10x.Genomics	Primary.Metastatic	33420488	33420488
PRAD_GSE143791	Human	None	16	36850	10x.Genomics	Metastatic	34719426	34719426
SS_GSE131309_10X	Human	None	3	9205	10x.Genomics	Primary	33495604	33495604
SS_GSE131309_Smartseq2	Human	None	12	7109	Smart.seq2	Primary	33495604	
GIST_GSE162115	Human	Targeted therapy	2	35308	10x.Genomics	Primary	33393143	33393143
PPB_GSE163678	Human	None	1	12239	10x.Genomics	Primary	None	None
SCLC_GSE150766	Human	None	1	1924	10x.Genomics	Primary	34016693	34016693
THCA_GSE148673	Human	None	5	19407	10x.Genomics	Primary	33462507	33462507
UVM_GSE138433	Human	None	6	12682	10x.Genomics	Primary	33462406	33462406
UVM_GSE160883	Human	None	6	12977	inDrop	Primary	34518527	34518527
total			840	2573921				

Supplementary table 9. 18 immunotherapy cohorts in bulk level.

**Dataset ID**	**Caner Type**	**PMID**	**Therapy**	**Size**	**Responder Number**	**Non-responder number**	**Time Point**
GBM-Zhao_2019	Glioblastoma	30996326	a-PD-1	34	17	17	pre
HNSC-Prat_2017	Squamous head and neck	28487385	a-PD-1	5	2	3	pre
LUSC-Prat_2017	Squamous-lung-carcinoma	28487385	a-PD-1	13	3	10	pre

Melanoma-Auslander_2018	Melanoma	30127394	a-CTLA-4, a-PD-1, and a-CTLA-4+a-PD-1	13	2	11	pre
Melanoma-Hugo_2016	Melanoma	28129544	a-PD-1	26	14	12	pre
Melanoma-Riaz_ 2017	Melanoma	29033130	a-PD-1	49	10	39	pre
Melanoma-Aleix_2017	Melanoma	28487385	a-PD-1	25	9	16	pre
Melanoma-Liu_2019	Melanoma	31792460	a-PD-1	121	47	74	pre
Melanoma-Nathanson_2017	Melanoma	27956380	a-CTLA-4	9	4	5	pre
Melanoma-Van_2015	Melanoma	26564858	a-CTLA-4	36	14	22	pre
Melanoma-Synder_ 2017	Melanoma	30753825	a-PD-1 and a-CTLA-4+a-PD-1	73	33	40	pre
NSCLC-Prat_2017	Nonsmall-cell lung cancer	28487385	a-PD-1	22	6	16	pre
NSCLC_Cho_2020	Nonsmall-cell lung cancer	32879421	a-PD-1	7	2	5	pre
NSCLC-Jung_2019	Nonsmall-cell lung cancer	31537801	a-PD-1	27	8	19	pre
RCC-Braun_2020	Renal cell carcinoma	32472114	a-PD-1	181	39	142	pre
GC-Kim_2018	Stomach adenocarcinoma	30013197	a-PD-1	45	12	33	pre
UC-Synder_2017	Urothelial carcinoma	28552987	a-PDL-1	25	6	19	pre
UC-Mariathasan_2018	Urothelial carcinoma	29443960	a-PDL-1	348	68	280	pre
Total	18 cohorts			1059	296	763	pre

Supplementary table 10. The 14 immunotherapy signatures used for comparison.

Signature Name	Title of source paper	Discription	PMID	Reference
IFNG	IFN-γ–related mRNA profile predicts clinical response to PD-1 blockade	Mean of the 6 gene expression levels	28650338	28650338
T cell–inflamed GEP	IFN-γ–related mRNA profile predicts clinical response to PD-1 blockade	Mean of the 18 gene expression levels	28650338	

PD-L1	Safety, activity, and immune correlates of anti-PD-1 antibody in cancer	PD-L1 expression by IHC	22658127	22658127
LRRC15+CAF	Single-Cell RNA Sequencing Reveals Stromal Evolution into LRRC15+ Myofibroblasts as a Determinant of Patient Response to Cancer Immunotherapy	eigenWeightedMean of the 14 gene expression levels	31699795	31699795
NLRP3 inflammasome	Pan-cancer analysis of NLRP3 inflammasome with potential implications in prognosis and immunotherapy in human cancer	ssGSEA of the 30 gene expression levels	33212483	33212483
Cytotoxic	Molecular and genetic properties of tumors associated with local immune cytolytic activity	Geometric mean of the 4 gene expression levels	25594174	25594174
ImmuneCells	A gene expression signature of TREM2hi macrophages and γδ T cells predicts immunotherapy response	Summing the log2 Z scores of 108 genes	33033253	33033253
T Cell Exclusion	A Cancer Cell Program Promotes T Cell Exclusion and Resistance to Checkpoint Blockade	Overall 203 gene expression levels	30388455	30388455
CRMA	Cancer-Germline Antigen Expression Discriminates Clinical Outcome to CTLA-4 Blockade	Geometric mean of the 8 gene expression levels	29656892	29656892
IMPRES	Robust prediction of response to immune checkpoint blockade therapy in metastatic melanoma	Comparision scores between 15 gene pairs	30127394	30127394
IPRES	Gene signatures of tumor inflammation and epithelial-to-mesenchymal transition (EMT) predict responses to immune checkpoint blockade in lung cancer with high accuracy	Mean z-scores of GSVA scores of 16 genes	31683225	31683225
TRS	Single-Cell Transcriptomic Analysis Reveals a Tumor-Reactive T Cell Signature Associated With Clinical Outcome and Immunotherapy Response In Melanoma	GSVA of the 6 gene expression levels	34804045	34804045
IMS	Ratio of the interferon-γ signature to the immunosuppression signature predicts anti-PD-1 therapy response in melanoma	Ratio of (IMS scores / IFN-γ scores)<br>IMS scores = Average gene expression of 18 immunosupression genes<br>IFN-γ scores = Average gene expression of 6 INF-γ genes	33542239	33542239

Supplementary table 11. Stemness-related signatures.

Name	Derivation	Stem Cell Type	Organism	Pubmed Id	**Reference**
Hs_EC_Skotheim	Expression Profiles	EC	Homo sapiens	15994931	15994931
Hs_ESC/EC_Sperger	Expression Profiles	ESC/EC	Homo sapiens	14595015	14595015
Hs_ESC_Assou	Expression Profiles	ESC	Homo sapiens	17204602	17204602
Hs_ESC_Bhattacharya	Expression Profiles	ESC	Homo sapiens	15070671	15070671
Hs_ESC_Sato	Expression Profiles	ESC	Homo sapiens	12921741	12921741
Hs_ESC_Skottman	Expression Profiles	ESC	Homo sapiens	16081666	16081666

Hs_ESC_Wong	Expression Profiles	ESC	Homo sapiens	18397753	18397753
Hs_HSC/MSC/NSC/ESC_Huang	Expression Profiles	HSC/MSC/NSC/ESC	Homo sapiens	18308945	18308945
Hs_HSC/MSC/NSC_Huang	Expression Profiles	HSC/MSC/NSC	Homo sapiens	18308945	
Hs_HSC_Huang	Expression Profiles	HSC	Homo sapiens	18308945	
Hs_HSC_Novershtern	Expression Profiles	HSC	Homo sapiens	21241896	21241896
Hs_HSC_Toren	Expression Profiles	HSC	Homo sapiens	16140871	16140871
Hs_MSC_Huang	Expression Profiles	MSC	Homo sapiens	18308945	18308945
Hs_MaSC_Pece	Expression Profiles	MaSC	Homo sapiens	20074520	20074520
Hs_NSC_Huang	Expression Profiles	NSC	Homo sapiens	18308945	18308945
Hs_SC_Palmer	Expression Profiles	SC	Homo sapiens	22909066	22909066
Hs_SC_Shats	Expression Profiles	SC	Homo sapiens	21169407	21169407
Hs_iPSC_Shats	Expression Profiles	IPSC	Homo sapiens	21169407	
Hs_ESC_Chia	RNAi Screens	ESC	Homo sapiens	20953172	20953172
KEGG	Literature Curation		Homo sapiens		
REACTOME	Literature Curation		Homo sapiens	24243840	24243840
HSC-Explorer	Literature Curation	HSC	Mus musculus	23936191	23936191
PluriNetWork	Literature Curation		Mus musculus	21179244	21179244
GeneCards	Computationally Derived		Homo sapiens	20689021	20689021
Plurinet	Computationally Derived		Homo sapiens	18724358	18724358

Supplementary table 12. Summary of the purified immune cell datasets and calculating the RCD score.

Sample.ID	RCD	alkaliptosis	anoikis	apoptosis	autophagy	autosis	cuproptosis	entotic_ccd	ferroptosis	immunogenic	lysosomal_cd	mitotic_cd	mpt	necroptosis	oxeiptosis	parthanatos	pyroptosis	Population	Data.set	PubMed.ID	Reference

	score																				
GSM349848	12.97155	1.074896	0.553506	0.777101	0.768525	0.826063	0.71491	1.16521	0.567249	0.682927	0.861173	0.795301	0.784329	0.962235	1.175496	0.693422	0.569205	T gamma delta	GSE13906	20132662	20132662
GSM349849	13.16254	1.190378	0.664354	0.778898	0.77671	0.798975	0.707286	1.181641	0.551836	0.671865	0.910974	0.750751	0.803705	0.941049	1.186195	0.700603	0.54732	T gamma delta	GSE13906	20132662	
GSM573357	12.68576	1.091856	0.642227	0.791032	0.768732	0.797121	0.722598	1.10321	0.534988	0.668064	0.868997	0.753386	0.795435	0.926825	0.995748	0.669477	0.556062	Immature dendritic cells	GSE23371	21708028	21708028
GSM573358	12.95513	1.045062	0.519196	0.771108	0.770678	0.80991	0.698893	1.267537	0.566476	0.678934	0.887688	0.769203	0.795416	0.950293	1.194755	0.671909	0.558075	Immature dendritic cells	GSE23371	21708028	
GSM573359	13.31618	1.263076	0.610702	0.764061	0.779524	0.812329	0.711682	1.136843	0.562851	0.681791	0.902837	0.764855	0.777193	0.957999	1.305314	0.713856	0.571272	Immature dendritic cells	GSE23371	21708028	
GSM622849	12.78719	1.020346	0.580477	0.77328	0.779173	0.807014	0.736741	1.139883	0.536109	0.663625	0.884725	0.758467	0.799995	0.938438	1.141258	0.689028	0.538633	Mast cells activated	GSE25320	24009880	24009880

GSM622850	12.97645	1.14143	0.444164	0.769799	0.74025	0.806591	0.654294	1.188398	0.543879	0.681625	0.906363	0.78289	0.772922	0.954989	1.361664	0.675076	0.552117	Mast cells activated	GSE25320	24009880	
GSM622851	13.14023	1.147881	0.69495	0.777261	0.773305	0.802671	0.75149	1.214302	0.559357	0.683513	0.858546	0.757219	0.802903	0.952502	1.123426	0.682859	0.558045	Mast cells activated	GSE25320	24009880	
GSM622852	12.87121	1.223949	0.631392	0.75469	0.781686	0.803981	0.693756	1.120472	0.568577	0.664416	0.885429	0.75631	0.77325	0.919836	1.037118	0.688044	0.568309	Mast cells activated	GSE25320	24009880	
GSM674836_BC2902B_6h	12.13176	1.216278	0.622623	0.702642	0.762595	0.753425	0.687672	1.048681	0.508395	0.638908	0.854822	0.66897	0.710064	0.904693	0.913007	0.613184	0.525801	T gamma delta	GSE27291	21968650	21968650
GSM674837_BC2902B_J7	13.0093	1.141547	0.611591	0.799135	0.747807	0.789005	0.587354	1.151771	0.562312	0.666308	0.870173	0.803931	0.771259	0.941369	1.305314	0.745671	0.514754	T gamma delta	GSE27291	21968650	
GSM674839_BC2901A_6h	12.57272	1.120109	0.640742	0.710063	0.7732	0.792032	0.777974	1.126223	0.499546	0.659406	0.88304	0.682731	0.794038	0.91282	1.028559	0.598985	0.573248	T gamma delta	GSE27291	21968650	
GSM674840_BC2901A_J7	13.04133	1.122373	0.626924	0.775493	0.75482	0.787819	0.584367	1.044892	0.599014	0.684863	0.887282	0.783398	0.788913	0.938809	1.395902	0.722313	0.544148	T gamma delta	GSE27291	21968650	
GSM674842_BC2802B_6h	12.47238	1.206923	0.545788	0.732818	0.76552	0.794835	0.728161	1.097413	0.523942	0.65859	0.847442	0.698161	0.744163	0.924474	0.993608	0.628641	0.581907	T gamma	GSE27291	21968650	

																		delta			
GSM674843_BC2802B_J7	13.38847	0.946552	0.493418	0.8219	0.749286	0.824545	0.762251	1.267402	0.582906	0.678174	0.88538	0.879335	0.800203	0.953119	1.334559	0.800873	0.608566	T gamma delta	GSE2791	21968650	
GSM674845_BC2401B_6h	12.83922	1.208054	0.586114	0.746347	0.78965	0.806915	0.766079	1.216782	0.516084	0.667328	0.912012	0.704681	0.812784	0.951144	0.975776	0.608548	0.570919	T gamma delta	GSE2791	21968650	
GSM674846_BC2401B_J7	13.27515	1.063435	0.581049	0.787769	0.762441	0.823992	0.639708	1.241819	0.589644	0.690739	0.886783	0.83971	0.789382	0.940139	1.350965	0.741311	0.546261	T gamma delta	GSE2791	21968650	
GSM687236	12.8898	1.115618	0.503532	0.753735	0.752826	0.838016	0.774554	1.263221	0.559526	0.6866	0.886358	0.795042	0.80418	0.934625	0.952951	0.662225	0.606792	NK activated	GSE7838	22419581	22419581
GSM687237	12.1915	1.012932	0.545308	0.750329	0.741887	0.800353	0.669475	0.912532	0.536616	0.668624	0.858857	0.733098	0.751746	0.91986	1.043538	0.645504	0.600836	NK activated	GSE7838	22419581	
GSM687238	12.3413	1.018092	0.524032	0.755496	0.775748	0.788474	0.661835	0.975316	0.570614	0.664432	0.881062	0.725695	0.822454	0.936034	1.034265	0.63522	0.572533	NK activated	GSE7838	22419581	
GSM687239	11.86935	1.0568	0.536542	0.745342	0.72192	0.779713	0.638146	1.003417	0.557398	0.6566	0.896676	0.73368	0.82341	0.889298	0.733972	0.5948	0.552758	NK activated	GSE7838	22419581	
GSM687240	13.60416	1.233482	0.682368	0.797734	0.782671	0.801559	0.74945	1.265987	0.561936	0.679462	0.880555	0.775296	0.749577	0.961149	1.370223	0.768468	0.544246	NK activated	GSE7838	22419581	
GSM687241	13.56135	1.155786	0.67909	0.791021	0.782963	0.805558	0.730716	1.318273	0.553373	0.691582	0.919187	0.76886	0.762572	0.964818	1.32172	0.745019	0.570809	NK activated	GSE7838	22419581	
GSM687242	12.93929	1.197193	0.569384	0.771849	0.771467	0.764624	0.72976	1.149348	0.527251	0.642307	0.845404	0.734407	0.764362	0.933887	1.310307	0.684145	0.543593	NK activated	GSE7838	22419581	

GSM687243	13.30166	1.266179	0.609835	0.767526	0.78131	0.800334	0.651277	1.295097	0.58285	0.667747	0.874548	0.772891	0.791124	0.943057	1.273216	0.681121	0.543545	NK activated	GSE27838	22419581	
GSM705297_mRNA_CD19_EXP11	13.17058	1.143894	0.577752	0.770548	0.778411	0.810129	0.699419	1.224332	0.558415	0.683542	0.888903	0.773025	0.771286	0.952409	1.281776	0.712548	0.544196	B cell activated	GSE28490	22276136	[22276136]
GSM705298_mRNA_CD19_EXP6	12.40507	1.09597	0.618145	0.740892	0.747453	0.78468	0.678289	1.093961	0.504738	0.655849	0.85872	0.736567	0.781145	0.905521	0.996461	0.643464	0.563214	B cell activated	GSE28490	22276136	
GSM705299_mRNA_CD19_EXP7	12.73358	1.191235	0.580591	0.760092	0.765733	0.791688	0.631714	1.076888	0.524944	0.665631	0.878306	0.743969	0.777553	0.959114	1.164083	0.682244	0.539796	B cell activated	GSE28490	22276136	
GSM705300_mRNA_CD19_EXP8	12.84729	1.180868	0.615096	0.757656	0.770488	0.795779	0.740086	1.184827	0.554028	0.664235	0.876172	0.753623	0.762016	0.924988	1.053524	0.646067	0.56784	B cell activated	GSE28490	22276136	
GSM705301_mRNA_CD19_EXP9	13.13217	1.042908	0.555587	0.787227	0.770093	0.821546	0.747269	1.240293	0.586363	0.688224	0.895243	0.790395	0.80935	0.94814	1.199035	0.693914	0.556306	B cell activated	GSE28490	22276136	
GSM710733_Rep1.TP.cntrl	12.40259	1.254935	0.538856	0.750075	0.758966	0.781286	0.587079	0.967239	0.518076	0.652072	0.962543	0.705046	0.802093	0.901985	1.029986	0.640806	0.551548	Eosinophils	GSE28698	22271894	[22271894]
GSM710735_Rep2.TP.cntrl	13.57899	1.07319	0.654321	0.785482	0.779793	0.839327	0.817149	1.29044	0.597204	0.699499	0.844112	0.828515	0.77846	0.975263	1.303888	0.741266	0.571086	Eosinophils	GSE28698	22271894	
GSM710977_1_CD4_1_N	13.57268	1.190734	0.644814	0.770762	0.774548	0.793001	0.793337	1.16087	0.58774	0.659602	0.942859	0.778236	0.843358	0.982866	1.360951	0.718731	0.570276	CD4 T cell resting	GSE28726	21632718	[21632718]
GSM710979_1_CD4_1_S	12.47	1.056299	0.58017	0.737	0.754	0.803	0.635426	1.1464	0.549	0.66615	0.829834	0.735	0.752	0.893881	1.086	0.680733	0.568	CD4 T cell	GSE2	21632718	

	618			054	778	531			328			435	067		335		752	activated	8726		
GSM711464_1_CD4_4_N	13.32643	1.22372	0.512094	0.754032	0.789466	0.787961	0.785249	1.089118	0.567611	0.661381	0.969004	0.781521	0.815158	0.962862	1.355244	0.721192	0.550816	CD4 T cell resting	GSE28726	21632718	
GSM711481_1_CD4_4_S	12.560015	1.034307	0.550508	0.742978	0.748318	0.785292	0.707787	1.131333	0.538529	0.675573	0.858235	0.735489	0.761494	0.910126	1.148391	0.672888	0.558901	CD4 T cell activated	GSE28726	21632718	
GSM711608_1_NKT_1_S	12.89073	1.087948	0.621413	0.760651	0.755726	0.824569	0.696591	1.235702	0.566413	0.691256	0.850226	0.755995	0.75651	0.905508	1.151958	0.680344	0.549925	NKT activated	GSE28726	21632718	
GSM711610_1_NKT_4_S	12.5262	0.980339	0.626791	0.761628	0.767714	0.796983	0.6576	1.156984	0.536265	0.674218	0.854298	0.749499	0.765732	0.933098	1.039258	0.659653	0.566614	NKT activated	GSE28726	21632718	
GSM711612_1_NKT_14_S	12.13311	1.069281	0.670606	0.783168	0.752164	0.794301	0.520397	1.037091	0.52361	0.662612	0.823303	0.736158	0.705773	0.917958	0.934405	0.668787	0.53349	NKT activated	GSE28726	21632718	
GSM711614_1_NKT_22_S	12.30825	1.261521	0.574553	0.769594	0.740533	0.795787	0.604266	1.130512	0.527558	0.654105	0.82217	0.729401	0.72061	0.925564	0.878769	0.63021	0.543094	NKT activated	GSE28726	21632718	
GSM711666_1_4_CD4_2_N	13.64328	1.174786	0.547378	0.768199	0.797619	0.806774	0.826049	1.207181	0.584163	0.666134	0.957677	0.803308	0.821317	0.973529	1.41302	0.73079	0.565356	CD4 T cell resting	GSE28726	21632718	
GSM711685_4_CD4_2_S	12.84273	1.142789	0.650268	0.77185	0.758967	0.7954	0.669318	1.159074	0.526931	0.685929	0.863623	0.744039	0.788091	0.922557	1.124853	0.665496	0.573549	CD4 T cell activated	GSE28726	21632718	

GSM711686_4_CD4_3_N	13.54254	1.184097	0.575217	0.764123	0.7727	0.80375	0.813269	1.191613	0.600562	0.663436	0.933853	0.799985	0.81471	0.957546	1.389482	0.731827	0.546372	CD4 T cell resting	GSE8726	21632718	
GSM711687_1_4_CD4_3_S	12.24596	1.117835	0.503	0.722359	0.746758	0.778978	0.639009	1.14813	0.522381	0.665703	0.823783	0.734421	0.731059	0.902467	1.029272	0.636385	0.54442	CD4 T cell activated	GSE8726	21632718	
GSM711689_4_NKT_1_S	12.518	1.18088	0.587682	0.756754	0.783544	0.799967	0.643513	1.187066	0.536803	0.654431	0.834151	0.742173	0.758364	0.926931	0.960797	0.62857	0.536373	NK T activated	GSE8726	21632718	
GSM711691_4_NKT_9_S	12.37298	1.162051	0.652046	0.770953	0.765237	0.79338	0.649866	0.981368	0.516332	0.675892	0.843368	0.732678	0.75068	0.923366	0.954377	0.641953	0.559437	NK T activated	GSE8726	21632718	
GSM926937_HD1_Balashov1_1.22.09_2	13.22745	1.175067	0.603154	0.776811	0.772055	0.821507	0.754934	1.168368	0.564395	0.694287	0.878747	0.777554	0.791592	0.960384	1.248965	0.689236	0.55039	Plasmacytoid dendritic cells	GSE37750	22688425	22688425
GSM926938_HD2_Balashov1_3.2.09	12.87014	1.097015	0.543013	0.781613	0.75782	0.8072	0.800046	1.155474	0.551836	0.671793	0.858074	0.78077	0.78313	0.976228	1.037118	0.694878	0.574133	Plasmacytoid dendritic cells	GSE37750	22688425	
GSM926939_HD3_Balashov3_21	13.16846	1.061897	0.596654	0.780157	0.790411	0.815078	0.636207	1.3028	0.558126	0.678315	0.875403	0.759616	0.798658	0.950816	1.312447	0.681163	0.570714	Plasmacytoid dendriti	GSE37750	22688425	

																		c cells			
GSM926940_HD4_Balashov3_19	12.73111	1.053987	0.480292	0.775924	0.758658	0.795347	0.635507	1.262824	0.524394	0.661957	0.906615	0.742777	0.810052	0.942765	1.150531	0.669347	0.560137	Plasmacytoid dendritic cells	GSE37750	22688425	
GSM926941_HD5_Balashov5_1_12.07.09	12.92988	1.132466	0.633396	0.747903	0.753863	0.805475	0.759796	1.092181	0.55939	0.676868	0.900181	0.77282	0.78507	0.927985	1.17193	0.662155	0.548401	Plasmacytoid dendritic cells	GSE37750	22688425	
GSM926942_HD6_Balashov5_1_12.10.09	12.9924	1.056705	0.707566	0.76962	0.774478	0.814109	0.687632	1.081265	0.553942	0.676553	0.888764	0.766346	0.7672	0.926485	1.292475	0.690454	0.538805	Plasmacytoid dendritic cells	GSE37750	22688425	
GSM926943_HD7_Balashov5_1_12.16.09	12.40308	1.144579	0.538812	0.753165	0.762326	0.790504	0.622812	1.192142	0.544432	0.657998	0.873417	0.742563	0.751632	0.915122	0.937972	0.661058	0.514549	Plasmacytoid dendritic cells	GSE37750	22688425	
GSM926944_HD8_Balashov5_8_07.06.10	12.87152	1.286423	0.65097	0.747261	0.760299	0.757611	0.715275	1.055278	0.533777	0.64457	0.873025	0.731091	0.793045	0.90495	1.160517	0.693105	0.564323	Plasmacytoid dendriti	GSE37750	22688425	

																		c cells			
GSM980737_C1	13.22974	1.087913	0.653397	0.777724	0.790971	0.809621	0.774405	1.201526	0.549997	0.662542	0.880579	0.766044	0.754635	0.985249	1.277496	0.691573	0.566072	Neutrophils	GSE39889	23451220	23451220
GSM980741_C2	12.77544	1.087822	0.576103	0.762638	0.750509	0.789506	0.668828	1.181372	0.522272	0.660795	0.886214	0.736841	0.811419	0.912461	1.192615	0.710432	0.525168	Neutrophils	GSE39889	23451220	
GSM980745_C3	13.21605	1.196213	0.554224	0.787376	0.764926	0.812148	0.805664	1.259057	0.537746	0.684404	0.904599	0.76935	0.827021	0.978951	1.087049	0.688255	0.559089	Neutrophils	GSE39889	23451220	
GSM980749_C4	12.59045	1.165119	0.623277	0.753681	0.773579	0.800952	0.577287	1.063072	0.57584	0.675119	0.865475	0.765185	0.755192	0.907326	1.083482	0.646831	0.559038	Neutrophils	GSE39889	23451220	
GSM1031685_204_PBMC_CD11c_U133_2.0	13.16722	1.127931	0.457304	0.790357	0.762965	0.819139	0.796041	1.248641	0.566862	0.688212	0.919487	0.771822	0.823435	0.962358	1.184769	0.670904	0.576991	Myeloid dendritic cells	GSE42058	--	--
GSM1031686_213_PBMC_CD11c_U133_2.0	12.4929	1.217915	0.725842	0.768699	0.755414	0.774179	0.574218	1.058386	0.532792	0.643418	0.875802	0.737963	0.736801	0.885545	1.025706	0.671621	0.508601	Myeloid dendritic cells	GSE42058	--	--
GSM1031687_214_PBMC_CD11c_U133_2.0	13.21857	1.149639	0.75421	0.772662	0.795506	0.792838	0.690376	1.189942	0.548864	0.668098	0.85024	0.748818	0.788325	0.944882	1.253244	0.702082	0.568842	Myeloid dendritic cells	GSE42058	--	--

GSM1031688_215_PBMC_CD11c_U133_2.0	12.768733	0.965215	0.486878	0.76677	0.762372	0.825948	0.734652	1.119755	0.568862	0.693481	0.880155	0.786013	0.781002	0.966631	1.157664	0.685416	0.587914	Myeloid dendritic cells	GSE42058	--	--
GSM1209554_HH1763_UI33plus2_201004	12.8458	1.173887	0.58962	0.728472	0.733306	0.776451	0.672671	1.374441	0.558743	0.678142	0.844798	0.760546	0.733514	0.860062	1.166937	0.663033	0.531176	B cell activated	GSE49910	24053356	24053356
GSM1209555_HH1778_u133plus2_211004	12.58746	1.099342	0.626623	0.731895	0.724943	0.781958	0.613714	1.270427	0.572805	0.676286	0.829091	0.768047	0.72352	0.854871	1.085622	0.693974	0.53434	B cell activated	GSE49910	24053356	
GSM1209556_HH1786_U133plus2_091104	12.90032	1.068855	0.754922	0.760623	0.745059	0.773461	0.621841	1.208622	0.562036	0.665587	0.844099	0.754439	0.732599	0.883469	1.309594	0.690292	0.524824	B cell activated	GSE49910	24053356	
GSM1209557_HH1791_u133plus2_251104	12.66272	1.095951	0.629289	0.753617	0.728585	0.783831	0.634326	1.257079	0.559781	0.672405	0.835074	0.765113	0.759725	0.881581	1.079202	0.698353	0.52881	B cell activated	GSE49910	24053356	
GSM1209558_HH1713_u133plus2_011004	13.41028	1.313075	0.73877	0.863633	0.806153	0.81549	0.64069	1.204422	0.519844	0.636135	0.908094	0.757112	0.837525	1.113782	0.854517	0.765872	0.635169	Neutrophils	GSE49910	24053356	
GSM1209559_HH1712_u133plus2_011004	13.39348	1.373867	0.78075	0.86441	0.813876	0.807353	0.640134	1.072455	0.509679	0.628983	0.916099	0.73333	0.868875	1.112137	0.893035	0.765976	0.612521	Neutrophils	GSE49910	24053356	
GSM1209560_HH1714_u133plus2_011004	13.51997	1.358363	0.854679	0.855513	0.818933	0.801566	0.653913	1.107762	0.517079	0.630582	0.867344	0.735448	0.858232	1.112155	0.964363	0.770372	0.613664	Neutrophils	GSE49910	24053356	
GSM1209564_HH1765_UI33plus2_201004	12.10229	1.091843	0.526013	0.729366	0.738215	0.764207	0.666681	0.934264	0.526866	0.656396	0.831232	0.744237	0.743778	0.898508	1.067077	0.625707	0.559765	CD8 T cell acti	GSE49910	24053356	

																		vated			
GSM1209565_HH1769_UI33plus2_201004	12.83731	1.091932	0.494612	0.754263	0.738993	0.792555	0.750362	1.125898	0.585339	0.669431	0.830643	0.777071	0.760711	0.930675	1.288909	0.664907	0.581011	CD4 T cell activated	GSE49910	24053356	
GSM1209566_HH1770_U133plus2_041104	12.31794	1.059843	0.684401	0.732608	0.77286	0.753702	0.56386	1.029869	0.525717	0.652822	0.830803	0.718948	0.735512	0.886573	1.176209	0.657343	0.536875	CD8 T cell activated	GSE49910	24053356	
GSM1209567_HH1774_u133plus2_211004	12.58018	1.111928	0.528425	0.756985	0.748505	0.793942	0.663081	0.983834	0.554034	0.667005	0.886436	0.755647	0.747531	0.946664	1.185482	0.67849	0.572189	CD4 T cell activated	GSE49910	24053356	
GSM1209568_HH1775_u133plus2_211004	12.70779	1.108147	0.581134	0.743346	0.75389	0.784298	0.72729	1.061853	0.536002	0.668468	0.842319	0.7494	0.733354	0.91764	1.266797	0.656349	0.577508	CD8 T cell activated	GSE49910	24053356	
GSM1209569_HH1780_u133plus2_211004	12.43848	1.168873	0.542754	0.718705	0.764571	0.779299	0.670324	1.056113	0.553249	0.665598	0.810117	0.759014	0.726649	0.886681	1.11986	0.63363	0.583045	CD8 T cell activated	GSE49910	24053356	
GSM1209570_HH1788_U133plus2_091104	12.17604	1.090556	0.564276	0.725456	0.749367	0.778653	0.681825	0.856152	0.544268	0.66562	0.826571	0.736102	0.71288	0.887994	1.141258	0.639572	0.575486	CD8 T cell activated	GSE49910	24053356	
GSM1209571_HH1792_u133plus2_251104	12.40772	1.069015	0.527721	0.724298	0.707004	0.804967	0.728827	0.893097	0.564524	0.66482	0.829854	0.769301	0.780253	0.888004	1.258951	0.64125	0.555833	CD4 T cell activated	GSE49910	24053356	

GSM1209572_HH1793_u133plus2_251104	12.51692	1.045217	0.461122	0.73152	0.735047	0.808178	0.696824	1.127293	0.56621	0.677626	0.821432	0.786418	0.740238	0.896862	1.187622	0.673508	0.561809	CD8 T cell activated	GSE49910	24053356	
GSM1209581_TW1676_u133plus2_061004	12.34053	0.988969	0.459865	0.72169	0.721355	0.79566	0.730229	1.194386	0.540042	0.655722	0.807995	0.777314	0.731615	0.889162	1.141258	0.646738	0.538534	CD8 T cell resting	GSE49910	24053356	
GSM1209582_TW1680_u133plus2_061004	12.30355	0.998629	0.518058	0.720433	0.735956	0.791921	0.706383	1.189517	0.540271	0.655113	0.82726	0.766869	0.750061	0.880769	1.058517	0.638304	0.525491	CD8 T cell resting	GSE49910	24053356	
GSM1209583_TW1684_u133plus2_061004	12.22316	1.023807	0.430054	0.719226	0.724849	0.792579	0.7261	1.195698	0.528074	0.657931	0.827513	0.76301	0.758528	0.88794	1.01572	0.648849	0.523284	CD8 T cell resting	GSE49910	24053356	
GSM1209584_TW1688_u133plus2_061004	12.24207	1.013325	0.482633	0.716492	0.731449	0.791157	0.711847	1.189878	0.535763	0.656738	0.850156	0.759367	0.724775	0.895161	1.029272	0.633484	0.520577	CD8 T cell resting	GSE49910	24053356	
GSM1209585_HH1762_UI33plus2_201004	13.61354	1.138687	0.605697	0.794166	0.799553	0.818229	0.720664	1.273851	0.572908	0.695044	0.985899	0.74883	0.823131	0.935379	1.380923	0.716271	0.604311	Monocytes	GSE49910	24053356	
GSM1209586_HH1767_UI33plus2_201004	13.38359	1.078013	0.586279	0.779054	0.777167	0.816412	0.75991	1.272959	0.579884	0.690927	0.972912	0.754865	0.818553	0.935325	1.278923	0.694119	0.588291	Monocytes	GSE49910	24053356	
GSM1209587_HH1772_u133plus2_211004	13.40167	1.232335	0.686505	0.797927	0.794744	0.802253	0.757977	1.148987	0.538289	0.68289	0.971925	0.724649	0.822743	0.967598	1.194755	0.691086	0.587002	Monocytes	GSE49910	24053356	
GSM1209588_HH1777_U133plus2_041104	13.2569	1.140859	0.651604	0.782354	0.808007	0.808701	0.710327	1.164211	0.5736	0.685612	0.970811	0.731027	0.810692	0.940005	1.191188	0.704781	0.583115	Monocytes	GSE49910	24053356	
GSM1209589_HH1785_U133plus2_011204	13.36	1.097762	0.617	0.77371	0.794	0.829	0.747998	1.239	0.578	0.697679	0.974433	0.754	0.79821	0.935609	1.246	0.692355	0.589	Monocytes	GSE4	24053356	

	773		957		691	217		057	643			544			111		757		9910		
GSM1209590_HH1790_U133plus2_091104	13.11421	1.06431	0.587534	0.76569	0.768365	0.807139	0.713646	1.124938	0.54686	0.687366	0.96671	0.73385	0.805232	0.921484	1.37165	0.680888	0.568554	Monocytes	GSE49910	24053356	
GSM1247614_1299_HG.U133_Plus_2_	13.38344	1.189009	0.48052	0.783735	0.768208	0.808646	0.712528	1.150727	0.594148	0.679841	0.879469	0.834098	0.800996	0.961423	1.412307	0.757662	0.570122	T helper 17	GSE51540	24492460	24492460
GSM1247616_1302_HG.U133_Plus_2_	12.48972	1.221909	0.44913	0.757742	0.778224	0.778081	0.629276	1.08856	0.536667	0.657752	0.872805	0.737061	0.781808	0.945394	1.020713	0.65219	0.582403	T helper 17	GSE51540	24492460	
GSM1247619_1305_HG.U133_Plus_2_	13.17844	1.253113	0.592599	0.786995	0.802951	0.800979	0.690293	1.145313	0.545369	0.653511	0.899925	0.764824	0.833423	0.957057	1.17193	0.703321	0.576842	T helper 17	GSE51540	24492460	
GSM1247620_1306_HG.U133_Plus_2_	13.40963	1.069429	0.587135	0.774845	0.763301	0.825531	0.710286	1.297477	0.603454	0.692701	0.870922	0.803116	0.79047	0.930527	1.376643	0.737518	0.576274	T helper 17	GSE51540	24492460	
GSM1247622_1308_HG.U133_Plus_2_	13.08997	1.120324	0.655736	0.787067	0.787597	0.797953	0.632432	1.30878	0.574996	0.67269	0.873463	0.718616	0.812959	0.923377	1.179062	0.688605	0.556314	T helper 17	GSE51540	24492460	
GSM1247624_1390_HG.U133_Plus_2_	13.10921	1.042172	0.576038	0.783273	0.741001	0.812931	0.772729	1.210335	0.570559	0.674331	0.898384	0.773686	0.82233	0.929287	1.238265	0.719705	0.544182	T helper 17	GSE51540	24492460	
GSM1247626_1392_HG.U133_Plus_2_	12.34795	1.160726	0.792761	0.767922	0.761036	0.792618	0.738525	0.993334	0.571911	0.672933	0.854858	0.699094	0.697942	0.921265	0.748951	0.616619	0.557459	T helper 17	GSE51540	24492460	
GSM1247628_1394_HG.U133_Plus_2_	12.79488	0.911361	0.528002	0.79122	0.748431	0.813068	0.717147	1.139713	0.560047	0.674779	0.87557	0.812539	0.787655	0.93722	1.243258	0.701118	0.553755	T helper 17	GSE51540	24492460	
GSM1247630_1396_HG.U133_Plus_2_	11.88604	1.114915	0.604739	0.740223	0.721942	0.758132	0.663239	1.015248	0.498814	0.642813	0.872202	0.691959	0.689919	0.903319	0.922993	0.55946	0.486122	T helper 17	GSE51540	24492460	
GSM1431110_DC_Control_0h_R1	12.80	0.958313	0.41302	0.709	0.738	0.774	0.76131	1.373	0.563	0.679351	0.864496	0.777	0.687	0.864381	1.398	0.688484	0.553	Dendritic	GSE5	25114264	2511

	507			554	978	402		708	453			318	127		041		137	cells resting	9237		4264
GSM1431111_DC_Control_0h_R2	13.06802	1.016529	0.553271	0.742091	0.787802	0.792977	0.663653	1.334113	0.553381	0.673997	0.951209	0.745218	0.792111	0.903739	1.273216	0.705392	0.57932	Dendritic cells resting	GSE59237	25114264	
GSM1431112_DC_Control_0h_R3	12.317789	1.043745	0.713374	0.762582	0.778841	0.778697	0.539124	0.916121	0.547339	0.650841	0.88275	0.743651	0.805022	0.944687	0.976489	0.659338	0.575295	Dendritic cells resting	GSE59237	25114264	
GSM1431113_DC_Control_0h_R4	12.915558	1.050921	0.559154	0.729863	0.772199	0.786568	0.659948	1.261055	0.546857	0.664267	0.909528	0.753683	0.769104	0.926348	1.306741	0.667418	0.551925	Dendritic cells resting	GSE59237	25114264	
GSM1431114_DC_Control_0h_R5	12.940554	1.078038	0.546883	0.742294	0.778251	0.790311	0.60933	1.254429	0.541546	0.66827	0.913006	0.764588	0.763417	0.933957	1.322433	0.68092	0.52871	Dendritic cells resting	GSE59237	25114264	
GSM1431115_DC_Control_0h_R6	12.89195	1.086887	0.469617	0.720937	0.742794	0.780617	0.751831	1.186921	0.586155	0.677941	0.87234	0.759637	0.748738	0.877535	1.341692	0.730541	0.557769	Dendritic cells resting	GSE59237	25114264	
GSM1431116_DC_Control_6h_R1	13.07604	1.335352	0.807055	0.837876	0.809601	0.805227	0.667284	0.967243	0.541982	0.674193	0.886797	0.720941	0.839363	1.01239	0.929412	0.677663	0.563659	Dendritic cells acti	GSE59237	25114264	

																		vated			
GSM1431117_DC_Control_6h_R2	12.90808	1.10366	0.515425	0.75092	0.732634	0.80999	0.783622	1.118387	0.558968	0.672972	0.827556	0.775029	0.803531	0.938963	1.246111	0.687243	0.583064	Dendritic cells activated	GSE59237	25114264	
GSM1431118_DC_Control_6h_R3	12.91842	1.286019	0.692663	0.802209	0.75431	0.817253	0.748437	1.147903	0.522765	0.667981	0.840592	0.754082	0.778232	0.95489	0.965077	0.642001	0.54401	Dendritic cells activated	GSE59237	25114264	
GSM1431119_DC_Control_6h_R4	12.78108	1.255159	0.712686	0.803056	0.742427	0.816389	0.742989	0.994534	0.53564	0.662917	0.850467	0.740309	0.805908	0.945898	0.943678	0.673107	0.559921	Dendritic cells activated	GSE59237	25114264	
GSM158184	12.99133	1.236137	0.634884	0.767041	0.780094	0.780912	0.637934	1.02969	0.549559	0.670116	0.907453	0.736745	0.763033	0.928971	1.335272	0.710433	0.523058	Immature dendritic cells	GSE6863	18314479	18314479
GSM158186	13.2074	1.129006	0.570529	0.776481	0.773973	0.815437	0.762848	1.276241	0.546698	0.677952	0.890208	0.776539	0.819	0.956794	1.16123	0.677672	0.596788	Immature dendritic cells	GSE6863	18314479	
GSM158187	12.58	1.04249	0.593	0.753	0.742	0.802	0.698903	1.181	0.551	0.671898	0.846459	0.751	0.766	0.931056	1.02	0.648314	0.583	Immature	GSE6	18314479	

	529		771	318	171	428		126	986			407	252				712	dendritic cells	863		
GSM198942	12.64385	1.137297	0.479814	0.773233	0.787214	0.794676	0.66451	1.387386	0.521709	0.671021	0.896596	0.713254	0.839158	0.956982	0.812433	0.614606	0.59396	NK resting	GSE8059	17623099	17623099
GSM198943	12.99826	1.185763	0.674553	0.77064	0.772129	0.811135	0.766139	1.127749	0.544312	0.668994	0.874759	0.735159	0.799947	0.96482	1.102028	0.632932	0.567197	NK activated	GSE8059	17623099	
GSM198944	12.888184	1.048691	0.548539	0.751548	0.764719	0.788269	0.7049	1.06599	0.567041	0.664868	0.890058	0.760768	0.756683	0.933101	1.342405	0.726984	0.56728	NK activated	GSE8059	17623099	
GSM198945	13.01089	1.114454	0.658477	0.765918	0.749267	0.817788	0.700432	1.017009	0.567234	0.679512	0.869501	0.837559	0.740281	0.906071	1.312447	0.765342	0.509601	NK activated	GSE8059	17623099	

Supplementary table 13. Summary of cancer cell lines information.

ID	RCD.score	cell_line_name	stripped_cell_line_name	CCLE_Name	alias	COSMIC ID	sex
ACH-001113	3.615898	LC-1/sq-SF	LC1SQSF	LC1SQSF_LUNG	LC-1F	NA	Male
ACH-001289	3.360114	COG-AR-359	COGAR359	COGAR359_SOFT_TISSUE		NA	Male
ACH-001339	4.038955	Colo 794	COLO794	COLO794_SKIN		NA	Male
ACH-001538	3.866288	KKU-213	KKU213	KKU213_BILIARY_TRACT	KKU-M213	NA	Male
ACH-000242	3.743189	RT4	RT4	RT4_URINARY_TRACT		687455	Male
ACH-000708	3.749386	SNU-283	SNU283	SNU283_LARGE_INTESTINE		1659929	Female
ACH-000327	3.360113	NCI-H1395	NCIH1395	NCIH1395_LUNG		684681	Female
ACH-000233	3.596861	DEL	DEL	DEL_HAEMATOPOIETIC_AND_LYMPHOID_TISSUE		906836	Male
ACH-000461	3.948818	SNU-1196	SNU1196	SNU1196_BILIARY_TRACT		NA	Unknown
ACH-000705	3.706891	LC-1F	LC1F	LC1F_LUNG	LC-1/sq-SF	NA	Male

ACH-001794	3.724111	93T449	93T449	93T449_SOFT_TISSUE	NA	Unknown
ACH-002023	3.929069	TGBC18TKB	TGBC18TKB	TGBC18TKB_LARGE_INTESTINE	NA	Female
ACH-000528	3.767831	ABC-1	ABC1	ABC1_LUNG	906791	Male
ACH-001655	3.629184	SKN	SKN	SKN_ENDOMETRIUM	1240215	Female
ACH-000167	3.820311	KE-97	KE97	KE97_STOMACH	NA	Male
ACH-000792	3.880538	BFTC-909	BFTC909	BFTC909_KIDNEY	910698	Male
ACH-001098	3.611166	KCI-MOH1	KCIMOH1	KCIMOH1_PANCREAS	NA	Female
ACH-000570	3.594473	YKG1	YKG1	YKG1_CENTRAL_NERVOUS_SYSTEM	687592	Female
ACH-000351	3.78321	MKN1	MKN1	MKN1_STOMACH	908138	Male
ACH-000769	3.575443	LK-2	LK2	LK2_LUNG	687787	Male
ACH-000154	3.958807	HS888T	HS888T	HS888T_FIBROBLAST	NA	Male
ACH-000828	3.645867	ZR-75-30	ZR7530	ZR7530_BREAST	909907	Female
ACH-000421	3.851242	SW837	SW837	SW837_LARGE_INTESTINE	909755	Male
ACH-000765	3.943757	WM-983B	WM983B	WM983B_SKIN	NA	Male
ACH-001375	3.519616	PACADD-119	PACADD119	PACADD119_PANCREAS	NA	Male
ACH-000008	4.097335	A101D	A101D	A101D_SKIN	910921	Male
ACH-001814	3.794478	OS252	OS252	OS252_BONE	NA	Unknown
ACH-000800	3.340802	NCI-H446	NCIH446	NCIH446_LUNG	688023	Male
ACH-000606	3.946984	PE/CA-PJ34 (clone C12)	PECAPJ34CLONEC12	PECAPJ34CLONEC12_UPPER_AERODIGESTIVE_TRACT	NA	Male
ACH-000317	3.66676	TUHR14TKB	TUHR14TKB	TUHR14TKB_KIDNEY	NA	Male
ACH-000123	3.678343	COV434	COV434	COV434_OVARY	NA	Female
ACH-000723	4.053799	YD-10B	YD10B	YD10B_UPPER_AERODIGESTIVE_TRACT	NA	Male

ACH-002066	3.682849	HS-Sch-2	HSSCH2	HSSCH2_CENTRAL_NERVOUS_SYSTEM		NA	Female
ACH-001536	3.589785	KKU-100	KKU100	KKU100_BILIARY_TRACT		NA	Female
ACH-000313	3.727852	KMRC-3	KMRC3	KMRC3_KIDNEY		NA	Male
ACH-002019	3.68387	HOKUG	HOKUG	HOKUG_CERVIX		NA	Female
ACH-000728	3.724199	KASUMI-2	KASUMI2	KASUMI2_HAEMATOPOIETIC_AND_LYMPHOID_TISSUE		NA	Male
ACH-000700	3.827296		NCIH2882	NCIH2882_LUNG	NCI-H2882	NA	Female
ACH-000823	3.626575	KYSE-140	KYSE140	KYSE140_OESOPHAGUS		753573	Male
ACH-000678	3.752579	MKN7	MKN7	MKN7_STOMACH		924250	Male
ACH-000006	3.95962	MONO-MAC-6	MONOMAC6	MONOMAC6_HAEMATOPOIETIC_AND_LYMPHOID_TISSUE		908148	Male
ACH-002029	3.831028	SAS	SAS	SAS_UPPER_AERODIGESTIVE_TRACT		NA	Female
ACH-001377	3.621007	PACADD-137	PACADD137	PACADD137_PANCREAS		NA	Female
ACH-000754	3.611304	L-428	L428	L428_HAEMATOPOIETIC_AND_LYMPHOID_TISSUE		907322	Female
ACH-000465	3.880726	SK-MEL-1	SKMEL1	SKMEL1_SKIN		909723	Male
ACH-000138	3.789795	CFPAC-1	CFPAC1	CFPAC1_PANCREAS		906821	Male
ACH-001864	3.680989	YSCCC	YSCCC	YSCCC_BILIARY_TRACT	YSCCC	NA	Female
ACH-000383	4.083607	OE33	OE33	OE33_OESOPHAGUS		910549	Female
NA	NA	NA	NA	NA	NA	NA	NA
ACH-000917	3.865604	TE-4	TE4	TE4_OESOPHAGUS		1503371	Female
ACH-000984	3.73668	HEC-6	HEC6	HEC6_ENDOMETRIUM		NA	Female
ACH-001409	3.709141	UM-UC-16	UMUC16	UMUC16_URINARY_TRACT		NA	Unknown
NA.1	NA	NA	NA	NA	NA	NA	NA
ACH-000117	3.731882	EFM-192A	EFM192A	EFM192A_BREAST		1290798	Female
ACH-000999	4.024515	SNU-1040	SNU1040	SNU1040_LARGE_INTESTINE		1659823	Male

ACH-000633	3.505759	FU97	FU97	FU97_STOMACH		1290806	Female
ACH-000520	3.845076	59M	59M	59M_OVARY		NA	Female
ACH-000176	3.625128	LOU-NH91	LOUNH91	LOUNH91_LUNG		1298226	Female
ACH-000017	3.652428	SK-BR-3	SKBR3	SKBR3_BREAST		NA	Female
ACH-000020	3.606315	MHH-CALL-2	MHHCALL2	MHHCALL2_HAEMATOPOIETIC_AND_LYMPHOID_TISSUE		908132	Female
ACH-001385	3.141498	RPMI 2650	RPMI2650	RPMI2650_UPPER_AERODIGESTIVE_TRACT		909700	Male
ACH-000587	3.95432	NCI-H1975	NCIH1975	NCIH1975_LUNG		924244	Female
ACH-000785	3.680079	NCI-H2126	NCIH2126	NCIH2126_LUNG		NA	Male
ACH-000554	3.741802	UACC-893	UACC893	UACC893_BREAST		909778	Female
ACH-001398	3.613094	SW 156	SW156	SW156_KIDNEY		1240220	Male
ACH-001991	3.192572	NZOV9	NZOV9	NZOV9_OVARY	NZOV 9, NZOV-9	NA	Female
ACH-001709	3.572287	WSU-NHL	WSUNHL	WSUNHL_HAEMATOPOIETIC_AND_LYMPHOID_TISSUE		909785	Female
ACH-001332	3.738607	BICR 78	BICR78	BICR78_UPPER_AERODIGESTIVE_TRACT		1240122	Male
ACH-000585	3.948681	EPLC-272H	EPLC272H	EPLC272H_LUNG		753556	Male
ACH-000550	3.850714	IGR-39	IGR39	IGR39_SKIN		NA	Male
ACH-000627	3.723766	LCLC-103H	LCLC103H	LCLC103H_LUNG		753586	Male
ACH-000704	3.732931	OAW42	OAW42	OAW42_OVARY		910548	Female
ACH-000276	3.831832	HCC38	HCC38	HCC38_BREAST		749717	Female
ACH-000075	3.947812	U-87 MG	U87MG	U87MG_CENTRAL_NERVOUS_SYSTEM		687590	Unknown
ACH-000818	3.565728	BT-483	BT483	BT483_BREAST		949093	Female
ACH-001616	3.24502	OCI-LY18	OCILY18	OCILY18_HAEMATOPOIETIC_AND_LYMPHOID_TISSUE		NA	Male
ACH-000625	3.735299	Hep 3B2.1-7	HEP3B217	HEP3B217_LIVER		1240147	Male

ACH-000941	3.533142	HEC-1-B	HEC1B	HEC1B_ENDOMETRIUM		NA	Female
ACH-000392	3.82944	Calu-3	CALU3	CALU3_LUNG		687777	Male
ACH-000269	3.950066	AM-38	AM38	AM38_CENTRAL_NERVOUS_SYSTEM		910933	Male
ACH-001670	3.268992	TASK1	TASK1	TASK1_CENTRAL_NERVOUS_SYSTEM		NA	Female
ACH-000856	3.59413	CAL-51	CAL51	CAL51_BREAST		910927	Female
ACH-001119	3.573096	Mac-2A	MAC2A	MAC2A_HAEMATOPOIETIC_AND_LYMPHOID_TISSUE		NA	Male
ACH-000376	3.930614	SF-295	SF295	SF295_CENTRAL_NERVOUS_SYSTEM		905985	Female
ACH-000685	3.672563	L3.3	L33	L33_PANCREAS		NA	Female
ACH-000729	3.196055	NCI-H1963	NCIH1963	NCIH1963_LUNG	H1963	688010	Male
ACH-001034	3.307794	CHLA-9	CHLA9	CHLA9_BONE		NA	Female
ACH-001283	3.296317	TC-106	TC106	TC106_BONE		NA	Male
ACH-000880	3.783743	AGS	AGS	AGS_STOMACH		906790	Female
ACH-000035	3.749579	NCI-H1650	NCIH1650	NCIH1650_LUNG		687800	Male
ACH-001200	3.474283	STM91-01	STM9101	STM9101_SOFT_TISSUE		NA	Male
ACH-000194	3.702839	Hs 934.T	HS934T	HS934T_FIBROBLAST		NA	Female
ACH-000322	3.903528	HT-144	HT144	HT144_SKIN		907067	Male
ACH-000552	3.897058	HT-29	HT29	HT29_LARGE_INTESTINE		905939	Female
ACH-002508	3.900706	WM3211	WM3211	WM3211_SKIN		NA	Female
ACH-000222	3.866906	AsPC-1	ASPC1	ASPC1_PANCREAS		910702	Female
ACH-000260	3.566856	SK-N-AS	SKNAS	SKNAS_AUTONOMIC_GANGLIA		724828	Female
ACH-001652	3.750193	SKG-II	SKGII	SKGII_ENDOMETRIUM		NA	Female
ACH-000608	3.890485	COV644	COV644	COV644_OVARY		NA	Female

ACH-000501	3.879975	LS123	LS123	LS123_LARGE_INTESTINE		907792	Female
ACH-000612	3.778061		HUT102	HUT102_HAEMATOPOIETIC_AND_LYMPHOID_TISSUE		NA	Male
ACH-001184	3.102059	SCMC-RM2	SCMCRM2	SCMCRM2_SOFT_TISSUE		NA	Female
ACH-001688	3.452044	UM-RC-7	UMRC7	UMRC7_KIDNEY		NA	Male
ACH-001838	3.920077	ICC12	ICC12	ICC12_BILIARY_TRACT		NA	Unknown
ACH-000638	3.879562	NCI-H441	NCIH441	NCIH441_LUNG		908460	Male
ACH-001846	3.745712	ICC6	ICC6	ICC6_BILIARY_TRACT		NA	Unknown
ACH-000798	3.928021	CL-40	CL40	CL40_LARGE_INTESTINE		1240124	Female
ACH-001321	3.145791	TT	TT	TT_THYROID		NA	Female
ACH-000894	3.730655	NCI-H1869	NCIH1869	NCIH1869_LUNG		1240183	Male
ACH-001559	3.692096	Mero-82	MERO82	MERO82_PLEURA		NA	Unknown
ACH-000344	3.760802	SNU-668	SNU668	SNU668_STOMACH		NA	Male
ACH-000223	3.758582	HCC1937	HCC1937	HCC1937_BREAST		749714	Female
ACH-000031	4.006224	Panc 02.13	PANC0213	PANC0213_PANCREAS		NA	Female
ACH-000034	3.921203		PLB985	PLB985_HAEMATOPOIETIC_AND_LYMPHOID_TISSUE	PLB985, PLB-985	NA	Female
ACH-001861	3.908983	TGBC1TKB	TGBC1TKB	TGBC1TKB_BILIARY_TRACT	TGBC1TKB	NA	Female
ACH-000772	3.633458	TE 441.T	TE441T	TE441T_SOFT_TISSUE		924248	Female
ACH-000946	3.875045	HEC-265	HEC265	HEC265_ENDOMETRIUM		NA	Female
ACH-000596	3.788779	LCLC-97TM1	LCLC97TM1	LCLC97TM1_LUNG		946361	Male
ACH-000632	4.126487	Hs 944.T	HS944T	HS944T_SKIN		NA	Male
ACH-000978	3.505372	EN	EN	EN_ENDOMETRIUM		1240127	Female
ACH-000332	4.078327	YAPC	YAPC	YAPC_PANCREAS		909904	Male

ACH-001841	3.833712	ICC15	ICC15	ICC15_BILIARY_TRACT		NA	Unknown
ACH-000179	3.415005	NCI-H1618	NCIH1618	NCIH1618_LUNG		NA	Female
ACH-000887	3.79032	SF-172	SF172	SF172_CENTRAL_NERVOUS_SYSTEM		NA	Female
ACH-000368	3.930471	SNU-1105	SNU1105	SNU1105_CENTRAL_NERVOUS_SYSTEM		NA	Male
ACH-000240	3.759195		HS600T	HS600T_FIBROBLAST	Hs 600.T	NA	Male
ACH-000578	3.796949	HCC-1171	HCC1171	HCC1171_LUNG		NA	Male
ACH-001611	3.889753	NP 8	NP8	NP8_CENTRAL_NERVOUS_SYSTEM		NA	Female
ACH-000135	3.734529	Hs 940.T	HS940T	HS940T_FIBROBLAST		1298145	Male
ACH-000406	3.806848	U-937	U937	U937_HAEMATOPOIETIC_AND_LYMPHOID_TISSUE		NA	Male
ACH-001541	3.468777	KMS-28PE	KMS28PE	KMS28PE_HAEMATOPOIETIC_AND_LYMPHOID_TISSUE		NA	Female
ACH-000998	3.891784	CW-2	CW2	CW2_LARGE_INTESTINE		910554	Female
ACH-000441	3.973582	SH-4	SH4	SH4_SKIN		909713	Female
ACH-000960	3.744178	Reh	REH	REH_HAEMATOPOIETIC_AND_LYMPHOID_TISSUE		909696	Female
ACH-000098	4.119903	GAMG	GAMG	GAMG_CENTRAL_NERVOUS_SYSTEM		906868	Female
ACH-001651	3.745465	SKG-I	SKGI	SKGI_ENDOMETRIUM		NA	Female
ACH-001456	3.609863	C125PM	C125PM	C125PM_LARGE_INTESTINE		NA	Unknown
ACH-001336	3.932908	Ca Ski	CASKI	CASKI_CERVIX		906824	Female
ACH-000284	3.913964		HS840T	HS840T_FIBROBLAST		NA	Male
ACH-000971	3.705383	HCT 116	HCT116	HCT116_LARGE_INTESTINE		905936	Male
ACH-001454	3.783869	C10	C10	C10_LARGE_INTESTINE		NA	Male
ACH-000926	3.688188	HT55	HT55	HT55_LARGE_INTESTINE		907287	Unknown
ACH-000033	3.497541	NCI-H1819	NCIH1819	NCIH1819_LUNG		NA	Female

ACH-000576	3.476754	KMS-27	KMS27	KMS27_HAEMATOPOIETIC_AND_LYMPHOID_TISSUE		NA	Male
ACH-000019	3.618051	MCF7	MCF7	MCF7_BREAST		905946	Female
ACH-001577	4.017472	MUTZ-8	MUTZ8	MUTZ8_HAEMATOPOIETIC_AND_LYMPHOID_TISSUE		NA	Female
ACH-000330	3.643456	EFM-19	EFM19	EFM19_BREAST		906851	Female
ACH-000848	3.611826	JHH-7	JHH7	JHH7_LIVER		1240160	Male
ACH-000481	3.622302	NCI-H2170	NCIH2170	NCIH2170_LUNG		687815	Male
ACH-001517	3.461945	HEC-1	HEC1	HEC1_ENDOMETRIUM		907051	Female
ACH-000338	3.623749	SR-786	SR786	SR786_HAEMATOPOIETIC_AND_LYMPHOID_TISSUE	SR	905965	Male
ACH-001959	3.646679	CC-LP-1	CCLP1	CCLP1_BILIARY_TRACT		NA	Female
ACH-001414	4.080306	UM-UC-6	UMUC6	UMUC6_URINARY_TRACT		NA	Male
ACH-000871	3.406116	NCI-H510	NCIH510	NCIH510_LUNG		753605	Male
ACH-001403	3.535233	TO14	TO14	TO14_OVARY		NA	Female
ACH-000805	3.879376	COLO-679	COLO679	COLO679_SKIN		906818	Female
ACH-001142	3.785064	NHAHTDD	NHAHTDD	NHAHTDD_PRIMARY		NA	Unknown
ACH-001554	3.650201	mel-202	MEL202	MEL202_UVEA		NA	Female
ACH-000847	3.378335	HGC-27	HGC27	HGC27_STOMACH		907055	Unknown
ACH-001207	3.869789	TIG-3 TD	TIG3TD	TIG3TD_FIBROBLAST		NA	Unknown
ACH-000832	4.022148	CAL 27	CAL27	CAL27_UPPER_AERODIGESTIVE_TRACT		910916	Male
ACH-000428	3.948783	UO-31	UO31	UO31_KIDNEY		905981	Unknown
ACH-001382	3.978726	PACADD-188	PACADD188	PACADD188_PANCREAS		NA	Female
ACH-001302	2.879446	COG-N-305	COGN305	COGN305_AUTONOMIC_GANGLIA		NA	Unknown
ACH-000690	3.709003	HCC-2814	HCC2814	HCC2814_LUNG		NA	Male

ACH-001134	3.362951	MYLA	MYLA	MYLA_HAEMATOPOIETIC_AND_LYMPHOID_TISSUE	NA	Unknown
ACH-000914	3.62089	HT	HT	HT_HAEMATOPOIETIC_AND_LYMPHOID_TISSUE	907063	Male
ACH-000858	3.937051	KNS-62	KNS62	KNS62_LUNG	753569	Male
ACH-001421	2.888408	WERI-Rb-1	WERIRB1	WERIRB1_AUTONOMIC_GANGLIA	NA	Female
ACH-000544	3.871361	OE21	OE21	OE21_OESOPHAGUS	1298359	Male
ACH-000725	3.379226	HCC202	HCC202	HCC202_BREAST	1290906	Female
ACH-000566	3.766483	SW-1710	SW1710	SW1710_URINARY_TRACT	909749	Female
ACH-000760	3.691888	LNZ308	LNZ308	LNZ308_CENTRAL_NERVOUS_SYSTEM	NA	Unknown
ACH-001745	3.211215	RhJT	RHJT	RHJT_SOFT_TISSUE	NA	Unknown
ACH-000787	3.818373	LXF-289	LXF289	LXF289_LUNG	753592	Male
ACH-000749	3.348552	DMS 273	DMS273	DMS273_LUNG	687985	Female
ACH-001328	3.779254	A-431	A431	A431_SKIN	910925	Female
ACH-001068	3.465449		FEPD	FEPD_HAEMATOPOIETIC_AND_LYMPHOID_TISSUE	NA	Female
ACH-000358	3.23742	NCI-H69	NCIH69	NCIH69_LUNG	688027	Male
ACH-001654	3.866325	SK-GT-4	SKGT4	SKGT4_OESOPHAGUS	1503365	Male
ACH-001521	3.697506	HKA-1	HKA1	HKA1_SKIN	NA	Female
ACH-000209	3.777769	SNU-1079	SNU1079	SNU1079_BILIARY_TRACT	NA	Male
ACH-001520	3.550309	HG-3	HG3	HG3_HAEMATOPOIETIC_AND_LYMPHOID_TISSUE	NA	Male
ACH-000213	3.855097	HUP-T4	HUPT4	HUPT4_PANCREAS	907286	Male
ACH-001610	3.535473	NP 5	NP5	NP5_CENTRAL_NERVOUS_SYSTEM	NA	Male
ACH-001408	3.884234	UM-UC-14	UMUC14	UMUC14_URINARY_TRACT	NA	Male
ACH-000991	3.927376	SNU-81	SNU81	SNU81_LARGE_INTESTINE	1660036	Male

ACH-001412	3.931722	UM-UC-10	UMUC10	UMUC10_URINARY_TRACT		NA	Unknown
ACH-000331	3.735125	IST-MES2	ISTMES2	ISTMES2_PLEURA	IST-MES-2	NA	Male
ACH-001407	3.633742	UM-UC-13	UMUC13	UMUC13_URINARY_TRACT		NA	Male
ACH-000580	4.051044	C32	C32	C32_SKIN		906830	Male
ACH-000594	3.423061	DMS 153	DMS153	DMS153_LUNG		NA	Male
ACH-000684	3.841818	KMRC-1	KMRC1	KMRC1_KIDNEY		1298168	Male
ACH-000742	3.942522		DM3	DM3_FIBROBLAST		NA	Male
ACH-000868	3.785607	HCC-1195	HCC1195	HCC1195_LUNG		NA	Male
ACH-000732	3.982324	PE/CA-PJ41 (clone D2)	PECAPJ41CLONED2	PECAPJ41CLONED2_UPPER_AERODIGESTIVE_TRACT		NA	Female
ACH-000429	3.375228	A-704	A704	A704_KIDNEY		910920	Male
ACH-000310	3.081627	IMR-32	IMR32	IMR32_AUTONOMIC_GANGLIA		NA	Male
ACH-000643	3.60834	HDQ-P1	HDQP1	HDQP1_BREAST		1290922	Female
ACH-000234	3.638077	Caki-2	CAKI2	CAKI2_KIDNEY		NA	Male
ACH-001498	3.598448	Farage	FARAGE	FARAGE_HAEMATOPOIETIC_AND_LYMPHOID_TISSUE		1297449	Female
ACH-000896	3.856816	647-V	647V	647V_URINARY_TRACT		906797	Male
ACH-000203	3.100731	NH-6	NH6	NH6_AUTONOMIC_GANGLIA		NA	Female
ACH-001607	4.030683	NOZ	NOZ	NOZ_LIVER		NA	Female
ACH-001441	3.424167	92-1	921	921_UVEA		NA	Female
ACH-000112	4.191883	SIG-M5	SIGM5	SIGM5_HAEMATOPOIETIC_AND_LYMPHOID_TISSUE		909715	Male
ACH-000186	3.902448	NCI-H2444	NCIH2444	NCIH2444_LUNG		1298356	Male
ACH-000677	3.732464	SW 1573	SW1573	SW1573_LUNG		724878	Female
ACH-000055	3.240811	D283 Med	D283MED	D283MED_CENTRAL_NERVOUS_SYSTEM	D283	906834	Male

ACH-000485	3.86791	GSU	GSU	GSU_STOMACH	NA	Male
ACH-000672	3.595989	IA-LM	IALM	IALM_LUNG	910779	Male
ACH-000287	3.745794	NU-DUL-1	NUDUL1	NUDUL1_HAEMATOPOIETIC_AND_LYMPHOID_TISSUE	1330982	Male
ACH-000784	3.704983	KYSE-70	KYSE70	KYSE70_OESOPHAGUS	753576	Male
ACH-000530	3.290837	DMS 114	DMS114	DMS114_LUNG	687983	Male
ACH-002024	3.261867	ECC4	ECC4	ECC4_LARGE_INTESTINE	NA	Male
ACH-000380	3.629398	KMS-12-BM	KMS12BM	KMS12BM_HAEMATOPOIETIC_AND_LYMPHOID_TISSUE	907281	Female
ACH-001737	3.386733	HSB2	HSB2	HSB2_HAEMATOPOIETIC_AND_LYMPHOID_TISSUE	NA	Unknown
ACH-001400	3.762212	SW 954	SW954	SW954_CERVIX	924247	Female
ACH-001054	3.034567	D458	D458	D458_CENTRAL_NERVOUS_SYSTEM	NA	Unknown
ACH-000009	3.751738	C2BBe1	C2BBE1	C2BBE1_LARGE_INTESTINE	910700	Male
ACH-000491	3.5124	NCI-H716	NCIH716	NCIH716_LARGE_INTESTINE	908458	Male
ACH-001703	3.388048	VAL	VAL	VAL_HAEMATOPOIETIC_AND_LYMPHOID_TISSUE	1331048	Female
ACH-000002	3.962045	HL-60	HL60	HL60_HAEMATOPOIETIC_AND_LYMPHOID_TISSUE	905938	Female
ACH-001568	3.526121	MM386	MM386	MM386_SKIN	NA	Male
ACH-000993	4.01837	JHUEM-7	JHUEM7	JHUEM7_ENDOMETRIUM	NA	Female
ACH-001578	3.192579	NCCIT	NCCIT	NCCIT_TESTIS	908441	Male
ACH-000877	3.601588	EB1	EB1	EB1_HAEMATOPOIETIC_AND_LYMPHOID_TISSUE	NA	Female
ACH-000711	3.803622	JIMT-1	JIMT1	JIMT1_BREAST	1298157	Female
ACH-001515	3.805269	HCS-2	HCS2	HCS2_CERVIX	NA	Female
ACH-000702	3.54218	L-1236	L1236	L1236_HAEMATOPOIETIC_AND_LYMPHOID_TISSUE	1330935	Male
ACH-001020	3.484574	BT-16	BT16	BT16_SOFT_TISSUE	NA	Male

ACH-000600	3.722774	SLR 26	SLR26	SLR26_KIDNEY		NA	Unknown
ACH-000354	3.646408	Capan-1	CAPAN1	CAPAN1_PANCREAS		753624	Male
ACH-000012	3.924282	HCC827	HCC827	HCC827_LUNG		1240146	Female
ACH-000689	3.648744	RH-18	RH18	RH18_SOFT_TISSUE		971774	Female
ACH-000459	3.910045	TUHR10TKB	TUHR10TKB	TUHR10TKB_KIDNEY		NA	Male
ACH-001413	3.744972	UM-UC-11	UMUC11	UMUC11_URINARY_TRACT		NA	Male
ACH-000686	3.973766	SNU-878	SNU878	SNU878_LIVER		NA	Female
ACH-000342	3.838059	CL-14	CL14	CL14_LARGE_INTESTINE		NA	Male
ACH-000208	3.776817	U-178	U178	U178_CENTRAL_NERVOUS_SYSTEM		NA	Male
ACH-000360	3.56743	NCI-H508	NCIH508	NCIH508_LARGE_INTESTINE		908442	Male
ACH-000249	3.848561	CL-11	CL11	CL11_LARGE_INTESTINE		1290769	Male
ACH-001368	3.515224	OAC-M5.1	OACM51	OACM51_OESOPHAGUS		1503363	Female
ACH-000231	3.850852	KALS-1	KALS1	KALS1_CENTRAL_NERVOUS_SYSTEM		907271	Female
ACH-000945	3.744262	NCI-H650	NCIH650	NCIH650_LUNG		722066	Male
ACH-000662	3.943214	COR-L23	CORL23	CORL23_LUNG	COR-L23/CPR	687780	Male
ACH-002458	3.813283	HT144 SKIN FV1	HT144SKINFV1	HT144_SKIN_FV1_RESISTANT	HT144_Dab	NA	Male
ACH-001378	3.765718	PACADD-159	PACADD159	PACADD159_PANCREAS		NA	Male
ACH-001029	3.119742	CHLA-10	CHLA10	CHLA10_BONE		NA	Female
ACH-000248	3.553233	AU565	AU565	AU565_BREAST		910704	Female
ACH-000943	3.389333	RKO	RKO	RKO_LARGE_INTESTINE		909698	Unknown
ACH-000751	3.714225	OCI-M1	OCIM1	OCIM1_HAEMATOPOIETIC_AND_LYMPHOID_TISSUE		1330985	Unknown
ACH-001016	3.8158	Becker	BECKER	BECKER_CENTRAL_NERVOUS_SYSTEM		906746	Male

ACH-000282	3.773577	NCI-H1755	NCIH1755	NCIH1755_LUNG		908475	Female
ACH-000261	3.885964	RERF-LC-AI	RERFLCAI	RERFLCAI_LUNG		NA	Unknown
ACH-000164	3.850401	PANC-1	PANC1	PANC1_PANCREAS		NA	Male
ACH-001793	3.827217	LPS27	LPS27	LPS27_SOFT_TISSUE		NA	Unknown
ACH-000262	3.754509	UOK101	UOK101	UOK101_KIDNEY		NA	Female
ACH-000158	3.774287	OCI-LY3	OCILY3	OCILY3_HAEMATOPOIETIC_AND_LYMPHOID_TISSUE		NA	Male
ACH-000773	3.495832	Ki-JK	KIJK	KIJK_HAEMATOPOIETIC_AND_LYMPHOID_TISSUE		NA	Male
ACH-000722	3.82761	SNU-C1	SNUC1	SNUC1_LARGE_INTESTINE		910905	Male
ACH-000857	4.164505	CAL-85-1	CAL851	CAL851_BREAST		910852	Female
ACH-001129	3.984023	MONO-MAC-1	MONOMAC1	MONOMAC1_HAEMATOPOIETIC_AND_LYMPHOID_TISSUE		NA	Male
ACH-001619	3.880294	OCUG-1	OCUG1	OCUG1_BILIARY_TRACT		NA	Male
ACH-000184	3.858892	Hs 343.T	HS343T	HS343T_FIBROBLAST		NA	Female
ACH-000329	3.866974	CCF-STTG1	CCFSTTG1	CCFSTTG1_CENTRAL_NERVOUS_SYSTEM		906823	Female
ACH-001569	3.57285	MM415	MM415	MM415_SKIN		NA	Male
ACH-000814	4.01739	Hs 939.T	HS939T	HS939T_SKIN		1298144	Female
NA.2	NA	NA	NA	NA	NA	NA	NA
ACH-000197	3.452627	TALL-1	TALL1	TALL1_HAEMATOPOIETIC_AND_LYMPHOID_TISSUE		909762	Male
ACH-000545	3.892178	VM-CUB1	VMCUB1	VMCUB1_URINARY_TRACT		909780	Male
ACH-000949	3.927638	TGBC11TKB	TGBC11TKB	TGBC11TKB_STOMACH		909770	Female
ACH-000124	3.543733	OCI-LY-19	OCILY19	OCILY19_HAEMATOPOIETIC_AND_LYMPHOID_TISSUE		1330984	Female
ACH-000560	3.329714	ECC10	ECC10	ECC10_STOMACH		906848	Male
ACH-000897	3.823816	FTC-238	FTC238	FTC238_THYROID		NA	Male
ACH-001626	4.052102	OSC-20	OSC20	OSC20_UPPER_AERODIGESTIVE_TRACT		1240196	Female

ACH-000619	3.875511	PE/CA-PJ15	PECAPJ15	PECAPJ15_UPPER_AERODIGESTIVE_TRACT		1240207	Male
ACH-001973	3.949046	MM485	MM485	MM485_SKIN	MM 485, MM-485	NA	Male
ACH-001340	3.628367	COV413A	COV413A	COV413A_OVARY		NA	Female
ACH-000546	3.959549	HSC-4	HSC4	HSC4_UPPER_AERODIGESTIVE_TRACT		907062	Male
ACH-002018	3.760007	Omm2.5	OMM25	OMM25_UVEA		NA	Male
ACH-000153	3.8549	NCI-H2052	NCIH2052	NCIH2052_PLEURA		688058	Male
ACH-000526	3.91894	Hs 821.T	HS821T	HS821T_FIBROBLAST		NA	Female
ACH-000116	3.602112	OAW28	OAW28	OAW28_OVARY		946360	Female
ACH-000815	3.753079	KM-H2	KMH2	KMH2_HAEMATOPOIETIC_AND_LYMPHOID_TISSUE		909976	Male
ACH-001270	3.613627	1273/99	127399	127399_SOFT_TISSUE		NA	Unknown
ACH-000324	3.998384	JHOC-5	JHOC5	JHOC5_OVARY		NA	Female
ACH-001561	3.693792	Mero-84	MERO84	MERO84_PLEURA		NA	Male
ACH-000842	3.713726	SW 480	SW480	SW480_LARGE_INTESTINE		NA	Male
ACH-000898	3.730919	SNU-719	SNU719	SNU719_STOMACH		NA	Male
ACH-000517	3.778696	SNU-410	SNU410	SNU410_PANCREAS		NA	Male
ACH-001415	3.795715	UM-UC7	UMUC7	UMUC7_URINARY_TRACT		NA	Male
ACH-000681	3.67129	A549	A549	A549_LUNG		905949	Male
ACH-001310	3.787479	HA1E	HA1E	HA1E_KIDNEY		NA	Unknown
ACH-000070	3.624667	697	697	697_HAEMATOPOIETIC_AND_LYMPHOID_TISSUE		906800	Male
ACH-000484	3.717384	VMRC-RCW	VMRCRCW	VMRCRCW_KIDNEY		1240224	Male
ACH-000108	3.705938	KP-3	KP3	KP3_PANCREAS		1298219	Male
ACH-000808	3.793528	HuH28	HUH28	HUH28_BILIARY_TRACT		NA	Female

ACH-000656	3.541561	SU-DHL-8	SUDHL8	SUDHL8_HAEMATOPOIETIC_AND_LYMPHOID_TISSUE		1331038	Male
ACH-000166	3.771099	Kasumi-6	KASUMI6	KASUMI6_HAEMATOPOIETIC_AND_LYMPHOID_TISSUE		NA	Male
ACH-000997	3.807349	HCT-15	HCT15	HCT15_LARGE_INTESTINE		905937	Male
ACH-000866	3.437751	NCI-H1048	NCIH1048	NCIH1048_LUNG		687995	Female
ACH-000819	3.93029	LN-18	LN18	LN18_CENTRAL_NERVOUS_SYSTEM		1240168	Male
ACH-000226	3.560223	SUP-M2	SUPM2	SUPM2_HAEMATOPOIETIC_AND_LYMPHOID_TISSUE		1331040	Female
ACH-000087	3.307166	SK-ES-1	SKES1	SKES1_BONE		684072	Male
ACH-000340	3.810293	Hs 870.T	HS870T	HS870T_FIBROBLAST		NA	Male
ACH-002005	3.778822	SK-MEL-19	SKMEL19	SKMEL19_SKIN	SK-MEL-19	NA	Male
ACH-000758	3.785158	MKN74	MKN74	MKN74_STOMACH		NA	Male
ACH-000196	3.590986	HCC1599	HCC1599	HCC1599_BREAST		749713	Female
ACH-000258	3.422769	Du4475	DU4475	DU4475_BREAST		906844	Female
ACH-000163	3.868799	SW579	SW579	SW579_THYROID		NA	Male
ACH-000076	3.696489	NCO2	NCO2	NCO2_HAEMATOPOIETIC_AND_LYMPHOID_TISSUE		NA	Female
ACH-000509	3.620239	HuT 78	HUT78	HUT78_HAEMATOPOIETIC_AND_LYMPHOID_TISSUE		NA	Male
ACH-001193	3.419818	SK-PN-DW	SKPNDW	SKPNDW_BONE		909731	Male
ACH-000571	3.910523	T98G	T98G	T98G_CENTRAL_NERVOUS_SYSTEM		687586	Male
ACH-000391	3.3337	MHH-ES-1	MHHES1	MHHES1_BONE		908134	Male
ACH-000293	3.693689	KLE	KLE	KLE_ENDOMETRIUM		924187	Female
ACH-000584	3.65361	JHOS-4	JHOS4	JHOS4_OVARY		1480359	Female
ACH-001690	4.027304	UPCI-SCC-026	UPCISCC026	UPCISCC026_UPPER_AERODIGESTIVE_TRACT		NA	Female
ACH-000586	3.347715	NCI-H1876	NCIH1876	NCIH1876_LUNG		1330972	Male

ACH-000944	3.593819	NAMALWA	NAMALWA	NAMALWA_HAEMATOPOIETIC_AND_LYMPHOID_TISSUE	908159	Female
ACH-000561	3.646361	T.T	TDOTT	TT_OESOPHAGUS	1299064	Male
ACH-001648	3.877175	Shmac 4	SHMAC4	SHMAC4_PROSTATE	NA	Unknown
ACH-000394	3.100084	NCI-H2081	NCIH2081	NCIH2081_LUNG	908480	Female
ACH-000954	3.782473	HEC-1-A	HEC1A	HEC1A_ENDOMETRIUM	NA	Female
ACH-000204	3.53235	LP-1	LP1	LP1_HAEMATOPOIETIC_AND_LYMPHOID_TISSUE	907791	Female
ACH-000160	3.53818	BT-12	BT12	BT12_SOFT_TISSUE	NA	Female
ACH-000989	3.726936	SNU-175	SNU175	SNU175_LARGE_INTESTINE	1659928	Female
ACH-001347	3.88833	H157	H157	H157_UPPER_AERODIGESTIVE_TRACT	NA	Male
ACH-000691	3.58391	HCC2157	HCC2157	HCC2157_BREAST	749715	Female
ACH-000268	3.712756	SNU-245	SNU245	SNU245_BILIARY_TRACT	NA	Unknown
ACH-000119	3.803676	Hs 863.T	HS863T	HS863T_FIBROBLAST	NA	Female
ACH-000379	3.719683	NCI-H1781	NCIH1781	NCIH1781_LUNG	1298350	Female
ACH-001341	3.787047	DoTc2 4510	DOTC24510	DOTC24510_CERVIX	906843	Female
ACH-000714	3.48288	KMS-11	KMS11	KMS11_HAEMATOPOIETIC_AND_LYMPHOID_TISSUE	1659817	Female
ACH-000937	3.359146	PF-382	PF382	PF382_HAEMATOPOIETIC_AND_LYMPHOID_TISSUE	909260	Female
ACH-000763	3.470231	MM1-S	MM1S	MM1S_HAEMATOPOIETIC_AND_LYMPHOID_TISSUE	1659818	Female
ACH-001673	3.61059	TFK-1	TFK1	TFK1_BILIARY_TRACT	NA	Male
ACH-000326	3.597513	JURL-MK1	JURLMK1	JURLMK1_HAEMATOPOIETIC_AND_LYMPHOID_TISSUE	1327771	Male
ACH-000300	3.834376		SLR25	SLR25_KIDNEY	NA	Unknown
ACH-000901	4.012969	HCC-1359	HCC1359	HCC1359_LUNG	NA	Female
ACH-000816	3.05914	NCI-H524	NCIH524	NCIH524_LUNG	908483	Male

ACH-000641	3.749434	CMK	CMK	CMK_HAEMATOPOIETIC_AND_LYMPHOID_TISSUE		910566	Male
ACH-000511	3.89553	Calu-1	CALU1	CALU1_LUNG		NA	Male
ACH-000583	3.575164	MC116	MC116	MC116_HAEMATOPOIETIC_AND_LYMPHOID_TISSUE		907799	Male
ACH-000836	4.081997	YD-15	YD15	YD15_SALIVARY_GLAND		NA	Male
ACH-000975	3.464716		HCC2450	HCC2450_LUNG		NA	Male
ACH-000109	3.795192	NCI-H3255	NCIH3255	NCIH3255_LUNG		NA	Female
ACH-000467	3.7978	HCC-56	HCC56	HCC56_LARGE_INTESTINE		1290907	Unknown
ACH-000712	3.700619	HCC-1833	HCC1833	HCC1833_LUNG		NA	Female
ACH-000671	3.502927	HuH-6	HUH6	HUH6_LIVER		NA	Male
ACH-000523	3.108152	NCI-H1184	NCIH1184	NCIH1184_LUNG	H1184	NA	Male
ACH-000864	3.082509	COLO 684	COLO684	COLO684_ENDOMETRIUM		910691	Female
ACH-000726	3.842505	TE-14	TE14	TE14_OESOPHAGUS		NA	Male
ACH-000136	2.940817	CHP-126	CHP126	CHP126_AUTONOMIC_GANGLIA		910567	Female
ACH-002067	3.452659	NOS-1	NOS1	NOS1_BONE		NA	Male
ACH-000440	3.319383	CA46	CA46	CA46_HAEMATOPOIETIC_AND_LYMPHOID_TISSUE		910703	Male
ACH-001461	3.698047	C99	C99	C99_LARGE_INTESTINE		NA	Male
ACH-000890	3.803727	SW 1271	SW1271	SW1271_LUNG		1299062	Male
ACH-001692	4.063046	UPCI-SCC-040	UPCISCC040	UPCISCC040_UPPER_AERODIGESTIVE_TRACT		NA	Male
ACH-000027	3.693998	GOS-3	GOS3	GOS3_CENTRAL_NERVOUS_SYSTEM		NA	Male
ACH-000562	4.117155	HCC-78	HCC78	HCC78_LUNG		1290908	Male
ACH-000748	3.660933	SJSA-1	SJSA1	SJSA1_BONE		909717	Male
NA.3	NA	NA	NA	NA	NA	NA	NA
ACH-000834	3.960746	UM-UC-1	UMUC1	UMUC1_URINARY_TRACT		NA	Male

ACH-000565	3.843238	RCM-1	RCM1	RCM1_LARGE_INTESTINE		909263	Female
ACH-000449	3.741702	MES-SA	MESSA	MESSA_SOFT_TISSUE		908127	Female
ACH-000399	3.5272	NCI-H2196	NCIH2196	NCIH2196_LUNG		908481	Male
NA.4	NA	NA	NA	NA	NA	NA	NA
ACH-000770	3.962828	P31/FUJ	P31FUJ	P31FUJ_HAEMATOPOIETIC_AND_LYMPHOID_TISSUE		909253	Male
ACH-000710	3.748814	NCI-H854	NCIH854	NCIH854_LUNG		NA	Male
ACH-000507	3.737623	KE-39	KE39	KE39_STOMACH		NA	Male
ACH-000066	3.728318	HCC4006	HCC4006	HCC4006_LUNG		NA	Male
ACH-000022	3.669704	PA-TU-8988S	PATU8988S	PATU8988S_PANCREAS		NA	Female
ACH-001668	3.23803	SUSA	SUSA	SUSA_TESTIS		NA	Male
ACH-000334	3.470608	DB	DB	DB_HAEMATOPOIETIC_AND_LYMPHOID_TISSUE		906832	Male
ACH-000374	4.025575	HCC1143	HCC1143	HCC1143_BREAST		749710	Female
ACH-000519	3.526275	PEER	PEER	PEER_HAEMATOPOIETIC_AND_LYMPHOID_TISSUE		NA	Female
ACH-000908	4.048195	SNU-520	SNU520	SNU520_STOMACH		NA	Female
ACH-002003	3.745314	A375 SKIN CJ3	A375SKINCJ3	A375_SKIN_CJ3_RESISTANT	A375 RMR SCH 0.25	NA	Unknown
ACH-000574	3.615658	FU-OV-1	FUOV1	FUOV1_OVARY		1240129	Female
ACH-000148	3.723827	Hs 578T	HS578T	HS578T_BREAST		905957	Female
ACH-002461	3.918882	RVH421 SKIN FV1	RVH421SKINFV1	RVH421_SKIN_FV1_RESISTANT	RVH421_Dab	NA	Male
ACH-000646	3.570058	OVMANA	OVMANA	OVMANA_OVARY		NA	Female
ACH-000314	4.081693	HCC-2108	HCC2108	HCC2108_LUNG		NA	Male
ACH-000040	3.863049	U-118 MG	U118MG	U118MG_CENTRAL_NERVOUS_SYSTEM		687588	Male
ACH-001057	3.539481		DERL2	DERL2_HAEMATOPOIETIC_AND_LYMPHOID_TISSUE		NA	Male
ACH-000430	3.824701	TYK-nu	TYKNU	TYKNU_OVARY		909774	Female

ACH-001041	3.505678	COLO-699	COLO699	COLO699_LUNG		NA	Female
NA.5	NA	NA	NA	NA	NA	NA	NA
ACH-000370	3.666111	SNU-626	SNU626	SNU626_CENTRAL_NERVOUS_SYSTEM		NA	Male
ACH-001630	3.550467	PEO1	PEO1	PEO1_OVARY		1480372	Female
ACH-000437	3.992151	SW 1088	SW1088	SW1088_CENTRAL_NERVOUS_SYSTEM		909745	Male
ACH-000498	3.866402	KO52	KO52	KO52_HAEMATOPOIETIC_AND_LYMPHOID_TISSUE		1330932	Male
ACH-001392	3.355222	SUM-185PE	SUM185PE	SUM185PE_BREAST		NA	Female
ACH-000503	3.860725	BICR 16	BICR16	BICR16_UPPER_AERODIGESTIVE_TRACT		NA	Male
ACH-000328	3.599682	LN-215	LN215	LN215_CENTRAL_NERVOUS_SYSTEM		NA	Female
ACH-000403	3.77842	NCI-H747	NCIH747	NCIH747_LARGE_INTESTINE		908457	Male
ACH-000400	3.764142	SK-CO-1	SKCO1	SKCO1_LARGE_INTESTINE		909718	Male
ACH-000224	3.633037		HS819T	HS819T_FIBROBLAST		NA	Male
ACH-000318	3.847287	TE-10	TE10	TE10_OESOPHAGUS		753622	Male
ACH-001430	3.197695	TC138	TC138	TC138_BONE		NA	Male
ACH-000928	3.680889	JHUEM-1	JHUEM1	JHUEM1_ENDOMETRIUM		NA	Female
ACH-000893	3.686965	NCI-H1651	NCIH1651	NCIH1651_LUNG		910900	Male
ACH-000889	3.458231	KMM-1	KMM1	KMM1_HAEMATOPOIETIC_AND_LYMPHOID_TISSUE		NA	Male
ACH-000799	3.855107	Hs 695T	HS695T	HS695T_SKIN		NA	Male
ACH-001623	3.880018	Onda 8	ONDA8	ONDA8_CENTRAL_NERVOUS_SYSTEM		NA	Female
ACH-000736	4.032142	SNU-601	SNU601	SNU601_STOMACH		NA	Male
ACH-000876	3.719835	MDA-MB-415	MDAMB415	MDAMB415_BREAST		924240	Female
ACH-001232	3.940355		UW228	UW228_CENTRAL_NERVOUS_SYSTEM		NA	Female
ACH-001618	3.455553	OCI-M2	OCIM2	OCIM2_HAEMATOPOIETIC_AND_LYMPHOID_TISSUE		NA	Unknown

ACH-000235	4.038334	Panc 04.03	PANC0403	PANC0403_PANCREAS		1298476	Male
ACH-000381	3.878761	T84	T84	T84_LARGE_INTESTINE		909761	Male
ACH-001993	3.411277	NALM-16	NALM16	NALM16_HAEMATOPOIETIC_AND_LYMPHOID_TISSUE	NALM-16, Nalm-16	NA	Female
ACH-001301	3.037196	COGN278	COGN278	COGN278_AUTONOMIC_GANGLIA		NA	Unknown
ACH-000854	3.632347	OCI-MY5	OCIMY5	OCIMY5_HAEMATOPOIETIC_AND_LYMPHOID_TISSUE		NA	Unknown
ACH-000187	3.260353	COR-L311	CORL311	CORL311_LUNG		687980	Male
ACH-000543	3.807139	SNU-489	SNU489	SNU489_CENTRAL_NERVOUS_SYSTEM		NA	Male
ACH-000569	3.688359	IST-MES1	ISTMES1	ISTMES1_PLEURA		907173	Female
ACH-000668	3.937083	HCC70	HCC70	HCC70_BREAST		907048	Female
ACH-000134	3.895198	HS274T	HS274T	HS274T_FIBROBLAST		NA	Female
ACH-000924	3.695342	NCI-H2172	NCIH2172	NCIH2172_LUNG		1298353	Female
ACH-001450	3.432043	BLUE-1	BLUE1	BLUE1_HAEMATOPOIETIC_AND_LYMPHOID_TISSUE		NA	Male
ACH-000296	3.543959	OUMS-23	OUMS23	OUMS23_LARGE_INTESTINE		NA	Male
ACH-000713	3.918992	Caov-3	CAOV3	CAOV3_OVARY		906825	Female
ACH-001360	3.648036	MS751	MS751	MS751_CERVIX		1240179	Female
ACH-000273	3.810833	SF539	SF539	SF539_CENTRAL_NERVOUS_SYSTEM		905984	Female
ACH-001715	3.572294	CAL-72	CAL72	CAL72_BONE		906827	Male
ACH-001617	3.415711	OCI-LY7	OCILY7	OCILY7_HAEMATOPOIETIC_AND_LYMPHOID_TISSUE		1659819	Male
ACH-000639	3.548271	NCI-H211	NCIH211	NCIH211_LUNG		1240189	Female
ACH-000590	3.805127	NCI-H2073	NCIH2073	NCIH2073_LUNG		NA	Female
ACH-000553	3.865107	Sq-1	SQ1	SQ1_LUNG		NA	Male
ACH-000084	4.138685		MUTZ3	MUTZ3_HAEMATOPOIETIC_AND_LYMPHOID_TISSUE	MUTZ3	NA	Male

ACH-000094	3.99307	HPAF-II	HPAFII	HPAFII_PANCREAS		724869	Male
ACH-000361	3.800501	SK-HEP-1	SKHEP1	SKHEP1_LIVER		909719	Male
ACH-000719	3.628096	RMG-I	RMGI	RMGI_OVARY		909699	Female
ACH-001396	3.493649	SUM-52PE, SUM52	SUM52PE	SUM52PE_BREAST		NA	Female
ACH-001848	3.809858	ICC8	ICC8	ICC8_BILIARY_TRACT		NA	Unknown
ACH-000339	4.081221		HCC461	HCC461_LUNG		NA	Male
NA.6	NA	NA	NA	NA	NA	NA	NA
ACH-000218	4.125458	PL-21	PL21	PL21_HAEMATOPOIETIC_AND_LYMPHOID_TISSUE		1330991	Male
ACH-000789	3.125552	NCI-H810	NCIH810	NCIH810_LUNG		925341	Male
ACH-000413	3.795423		HS739T	HS739T_FIBROBLAST		NA	Female
ACH-000125	3.892945	TO 175.T	TO175T	TO175T_FIBROBLAST		NA	Male
ACH-000180	3.75832	Hs 839.T	HS839T	HS839T_FIBROBLAST		NA	Female
ACH-000215	4.024485	LN382	LN382	LN382_CENTRAL_NERVOUS_SYSTEM	LN-382	NA	Male
ACH-001001	3.817644	143B	143B	143B_BONE		NA	Female
ACH-000407	3.792317	SNU-685	SNU685	SNU685_ENDOMETRIUM		NA	Female
ACH-002027	3.645411	HOUA-I	HOUAI	HOUAI_ENDOMETRIUM		NA	Female
ACH-001132	3.47747		MTA	MTA_HAEMATOPOIETIC_AND_LYMPHOID_TISSUE		NA	Female
ACH-001278	3.511204	BIN-67	BIN67	BIN67_OVARY		NA	Female
ACH-000032	3.582962	MHH-CALL-3	MHHCALL3	MHHCALL3_HAEMATOPOIETIC_AND_LYMPHOID_TISSUE		NA	Female
ACH-001419	3.655869	VP229	VP229	VP229_BREAST	VP229	NA	Female
ACH-000494	3.8509	OELE	OELE	OELE_OVARY	OEL(E)	NA	Unknown
ACH-000865	3.50362	KYSE-450	KYSE450	KYSE450_OESOPHAGUS		907320	Male
ACH-000439	3.91157	ME-1	ME1	ME1_HAEMATOPOIETIC_AND_LYMPHOID_TISSUE		1330942	Male

ACH-001523	3.968843	HSC-1	HSC1	HSC1_SKIN		NA	Male
ACH-000024	3.432079	OPM-2	OPM2	OPM2_HAEMATOPOIETIC_AND_LYMPHOID_TISSUE		909249	Female
ACH-000674	3.913193	NUGC-4	NUGC4	NUGC4_STOMACH		1298357	Female
ACH-000670	3.284917	SBC-5	SBC5	SBC5_LUNG		713880	Male
ACH-000599	3.948723	PA-TU-8902	PATU8902	PATU8902_PANCREAS		1298526	Female
ACH-000263	3.894251	KASUMI-1	KASUMI1	KASUMI1_HAEMATOPOIETIC_AND_LYMPHOID_TISSUE		907275	Male
ACH-000303	3.799509	SNU-5	SNU5	SNU5_STOMACH		908445	Female
ACH-001551	3.684501	MCC14/2	MCC142	MCC142_SKIN		NA	Male
ACH-000793	4.000413	KATO III	KATOIII	KATOIII_STOMACH		907276	Male
ACH-002511	3.937986	M140325	M140325	M140325_SKIN		NA	Female
ACH-000286	3.893374	SNU-1033	SNU1033	SNU1033_LARGE_INTESTINE		NA	Female
ACH-000250	3.732566	KMRC-20	KMRC20	KMRC20_KIDNEY		1298169	Unknown
ACH-000299	3.560207		HNT34	HNT34_HAEMATOPOIETIC_AND_LYMPHOID_TISSUE	HNT-34	NA	Female
ACH-000139	3.950965	Panc 03.27	PANC0327	PANC0327_PANCREAS		925346	Female
ACH-001735	3.328275	SEMK2	SEMK2	SEMK2_HAEMATOPOIETIC_AND_LYMPHOID_TISSUE		NA	Female
ACH-001389	3.609219	SUM-1315MO2	SUM1315MO2	SUM1315MO2_BREAST		NA	Female
ACH-001183	3.921585		RT11284	RT11284_URINARY_TRACT		NA	Female
ACH-000551	3.490618	K-562	K562	K562_HAEMATOPOIETIC_AND_LYMPHOID_TISSUE		905940	Female
ACH-001163	3.539728	Peds005T Susp	CCLFPEDS0001T	PEDS005TSUSP_KIDNEY		NA	Male
ACH-000037	3.930758	S-117	S117	S117_THYROID		NA	Female
ACH-000744	3.645017	NCI-H1623	NCIH1623	NCIH1623_LUNG		687798	Male
ACH-000420	3.632728	SNU-449	SNU449	SNU449_LIVER		909738	Male

ACH-000336	3.980128	OCI-AML3	OCIAML3	OCIAML3_HAEMATOPOIETIC_AND_LYMPHOID_TISSUE		1290455	Male
ACH-000486	3.763068	KU-19-19	KU1919	KU1919_URINARY_TRACT		907312	Male
ACH-000679	3.736768	OE-19	OE19	OE19_OESOPHAGUS		910079	Male
ACH-001557	3.824435	Mero-41	MERO41	MERO41_PLEURA		NA	Male
ACH-000247	3.962132	OCUM-1	OCUM1	OCUM1_STOMACH		1298358	Female
ACH-000947	3.504346	OVK18	OVK18	OVK18_OVARY		1480371	Female
ACH-000807	3.602762	SNU-738	SNU738	SNU738_CENTRAL_NERVOUS_SYSTEM		NA	Male
ACH-000476	3.689702	JHH-4	JHH4	JHH4_LIVER		1240158	Male
ACH-000525	3.169029	NCI-H2171	NCIH2171	NCIH2171_LUNG	H2171	688015	Male
ACH-001148	3.475976	OCILY-13	OCILY132	OCILY132_HAEMATOPOIETIC_AND_LYMPHOID_TISSUE		NA	Female
ACH-000853	3.658392	NCI-H661	NCIH661	NCIH661_LUNG		687829	Male
ACH-000083	3.849854	Hs 281.T	HS281T	HS281T_FIBROBLAST		NA	Female
ACH-000479	3.717819	KNS-81	KNS81	KNS81_CENTRAL_NERVOUS_SYSTEM		NA	Male
ACH-000230	3.900853	Hs 742.T	HS742T	HS742T_FIBROBLAST		NA	Female
ACH-001664	3.673609	SNU-638	SNU638	SNU638_STOMACH		NA	Male
ACH-000955	3.76897	SNU-407	SNU407	SNU407_LARGE_INTESTINE		1660034	Male
ACH-000049	3.701681	HEK TE	HEKTE	HEKTE_KIDNEY		NA	Unknown
ACH-000675	3.871172	NCI-H1734	NCIH1734	NCIH1734_LUNG		722058	Female
ACH-000199	3.827669	HS255T	HS255T	HS255T_FIBROBLAST		NA	Female
ACH-000193	3.741661	KARPAS-620	KARPAS620	KARPAS620_HAEMATOPOIETIC_AND_LYMPHOID_TISSUE		1327775	Female
ACH-000538	3.47378	HUTU80	HUTU80	HUTU80_SMALL_INTESTINE		907073	Male
ACH-001647	3.931629	SHI-1	SHI1	SHI1_HAEMATOPOIETIC_AND_LYMPHOID_TISSUE		NA	Male

ACH-001649	3.84659	Shmac 5	SHMAC5	SHMAC5_PROSTATE	NA	Unknown
ACH-000155	3.766655	SW 1990	SW1990	SW1990_PANCREAS	910907	Male
ACH-000270	3.671328	HPAC	HPAC	HPAC_PANCREAS	1298136	Female
ACH-000683	3.689565	SNU-503	SNU503	SNU503_LARGE_INTESTINE	NA	Male
ACH-002017	3.284207	Omm1	OMM1	OMM1_UVEA	NA	Male
ACH-000655	3.843084	SF268	SF268	SF268_CENTRAL_NERVOUS_SYSTEM	905986	Female
ACH-000145	3.882225	SK-LMS-1	SKLMS1	SKLMS1_SOFT_TISSUE	909720	Female
ACH-001495	3.738699	EMTOKA	EMTOKA	EMTOKA_ENDOMETRIUM	NA	Female
ACH-000188	3.975823	SCC-25	SCC25	SCC25_UPPER_AERODIGESTIVE_TRACT	910701	Male
ACH-000080	3.814857	BDCM	BDCM	BDCM_HAEMATOPOIETIC_AND_LYMPHOID_TISSUE	NA	Male
ACH-000146	3.852673	THP-1	THP1	THP1_HAEMATOPOIETIC_AND_LYMPHOID_TISSUE	909771	Male
ACH-000415	3.901895	BICR 6	BICR6	BICR6_UPPER_AERODIGESTIVE_TRACT	NA	Male
ACH-001518	3.593026	HEC-116	HEC116	HEC116_ENDOMETRIUM	NA	Female
ACH-000891	3.892587	HCC-1438	HCC1438	HCC1438_LUNG	NA	Male
ACH-001625	3.924508	OSC-19	OSC19	OSC19_UPPER_AERODIGESTIVE_TRACT	1298362	Male
ACH-000895	3.896074	CL-34	CL34	CL34_LARGE_INTESTINE	1290771	Female
ACH-000851	3.658372	MOR/CPR	MORCPR	MORCPR_LUNG	NA	Unknown
ACH-000824	3.813209	KYSE-510	KYSE510	KYSE510_OESOPHAGUS	907321	Female
ACH-000506	3.317824	NCI-H146	NCIH146	NCIH146_LUNG	910899	Male
ACH-000540	3.897598	Hs 606.T	HS606T	HS606T_FIBROBLAST	NA	Female
ACH-000061	3.616474	HH	HH	HH_HAEMATOPOIETIC_AND_LYMPHOID_TISSUE	907056	Male
ACH-000185	3.837018		HS737T	HS737T_FIBROBLAST	NA	Female

ACH-000568	3.547906	UACC-812	UACC812	UACC812_BREAST		910910	Female
ACH-000707	3.613188	P3HR-1	P3HR1	P3HR1_HAEMATOPOIETIC_AND_LYMPHOID_TISSUE		NA	Male
ACH-000603	3.617096	BEN	BEN	BEN_LUNG		753534	Male
ACH-000983	3.574323	KCL-22	KCL22	KCL22_HAEMATOPOIETIC_AND_LYMPHOID_TISSUE		1330931	Female
ACH-000541	3.665976	KMS-34	KMS34	KMS34_HAEMATOPOIETIC_AND_LYMPHOID_TISSUE		NA	Female
ACH-000680	3.663811	SW948	SW948	SW948_LARGE_INTESTINE		909757	Female
ACH-002460	4.000113	HT144 SKIN FV2	HT144SKINFV2	HT144_SKIN_FV2_RESISTANT	HTT144_Dab_Rox	NA	Male
ACH-000111	3.801236	HCC1187	HCC1187	HCC1187_BREAST		749711	Female
ACH-002463	3.686095	RPE1-ss77	RPE1SS77	RPE1SS77_ENGINEERED		NA	Female
ACH-000369	3.695764	MOLM-16	MOLM16	MOLM16_HAEMATOPOIETIC_AND_LYMPHOID_TISSUE		1330948	Female
ACH-000618	3.984439	SNU-1041	SNU1041	SNU1041_UPPER_AERODIGESTIVE_TRACT		NA	Male
ACH-000470	3.922452	SW1463	SW1463	SW1463_LARGE_INTESTINE		909748	Female
ACH-002039	3.947848	PK-8	PK8	PK8_PANCREAS		NA	Unknown
ACH-000118	3.722787	HUP-T3	HUPT3	HUPT3_PANCREAS		907285	Male
ACH-000614	4.001424	RVH-421	RVH421	RVH421_SKIN		909706	Male
ACH-001338	2.905978	CHP-134	CHP134	CHP134_AUTONOMIC_GANGLIA		910941	Male
ACH-000432	3.733622	BV-173	BV173	BV173_HAEMATOPOIETIC_AND_LYMPHOID_TISSUE		910710	Male
ACH-000556	3.630432	SIHA	SIHA	SIHA_CERVIX		930297	Female
ACH-001804	3.858296	LPS510	LPS510	LPS510_SOFT_TISSUE		NA	Unknown
ACH-001386	3.234424	SCLC-22H	SCLC22H	SCLC22H_LUNG		NA	Male
ACH-001060	3.612563		DL40	DL40_HAEMATOPOIETIC_AND_LYMPHOID_TISSUE	DL-40	NA	Female
ACH-001525	3.636466	HT-3	HT3	HT3_CERVIX		907068	Female

ACH-001539	3.427127	KML-1	KML1	KML1_HAEMATOPOIETIC_AND_LYMPHOID_TISSUE		NA	Female
ACH-000014	4.0834	Hs 294T	HS294T	HS294T_SKIN	A101D, Hs 294.T	NA	Male
ACH-000637	3.840558	KYSE-520	KYSE520	KYSE520_OESOPHAGUS		753575	Female
ACH-000455	3.696776	LN-428	LN428	LN428_CENTRAL_NERVOUS_SYSTEM		NA	Male
ACH-000389	3.778703	H4	H4	H4_CENTRAL_NERVOUS_SYSTEM		907042	Male
ACH-000129	3.611855	NCI-H1341	NCIH1341	NCIH1341_LUNG		1330964	Female
ACH-000490	3.767843	SF767	SF767	SF767_CENTRAL_NERVOUS_SYSTEM		NA	Female
ACH-000434	3.58885	NCI-H1915	NCIH1915	NCIH1915_LUNG		1240184	Female
ACH-001086	3.651428		HELA	HELA_CERVIX	HeLa	1298134	Female
ACH-002042	4.092267	T3M-5	T3M5	T3M5_THYROID		NA	Female
ACH-000597	3.438015	TTC-709	TTC709	TTC709_SOFT_TISSUE		NA	Unknown
ACH-000756	3.706084	GI-1	GI1	GI1_CENTRAL_NERVOUS_SYSTEM		906871	Male
ACH-000396	3.76404	J82	J82	J82_URINARY_TRACT		753566	Male
ACH-000089	3.686058	NCI-H684	NCIH684	NCIH684_LARGE_INTESTINE		NA	Male
ACH-000371	3.692825	RL	RL	RL_HAEMATOPOIETIC_AND_LYMPHOID_TISSUE		910861	Male
ACH-000265	3.791612	KP4	KP4	KP4_PANCREAS		753572	Male
ACH-000132	3.670688	JHOS-2	JHOS2	JHOS2_OVARY		1479995	Female
ACH-000216	3.975366	JH-EsoAd1	JHESOAD1	JHESOAD1_OESOPHAGUS		NA	Male
ACH-000613	3.526318	HOS	HOS	HOS_BONE		907060	Female
ACH-000855	3.835705	KYSE-150	KYSE150	KYSE150_OESOPHAGUS		907317	Female
ACH-000906	3.908365	ES-2	ES2	ES2_OVARY		1240128	Female
ACH-000348	3.938652	RPMI-7951	RPMI7951	RPMI7951_SKIN		910903	Female

ACH-001100	3.16271		KHYG	KHYG_HAEMATOPOIETIC_AND_LYMPHOID_TISSUE	KHYG1, KHYG-1	NA	Unknown
ACH-000238	3.93725	SCC-4	SCC4	SCC4_UPPER_AERODIGESTIVE_TRACT		910904	Male
ACH-000237	3.971973	JHOM-1	JHOM1	JHOM1_OVARY		NA	Female
ACH-000393	3.856194	HLF	HLF	HLF_LIVER		NA	Male
ACH-000623	3.83	SNU-201	SNU201	SNU201_CENTRAL_NERVOUS_SYSTEM		NA	Male
ACH-000359	3.689771	MG-63	MG63	MG63_BONE		908131	Male
ACH-001028	3.238091	CHLA-06	CHLA06ATRT	CHLA06ATRT_SOFT_TISSUE		NA	Female
ACH-000621	3.400126	MDA-MB-157	MDAMB157	MDAMB157_BREAST		925338	Female
ACH-001641	3.944425	SAT	SAT	SAT_UPPER_AERODIGESTIVE_TRACT		1299050	Unknown
ACH-000056	3.740096	DOHH-2	DOHH2	DOHH2_HAEMATOPOIETIC_AND_LYMPHOID_TISSUE		906842	Male
ACH-000483	3.615794	SNU-182	SNU182	SNU182_LIVER		1240216	Male
ACH-000514	2.995876	NCI-H1092	NCIH1092	NCIH1092_LUNG		687997	Male
ACH-000051	3.562763	TE 617.T	TE617T	TE617T_SOFT_TISSUE		NA	Female
ACH-000043	3.801519	Hs 895.T	HS895T	HS895T_FIBROBLAST		NA	Female
ACH-000936	3.742532	EFO-27	EFO27	EFO27_OVARY		906852	Female
ACH-000716	3.967037	TT2609-C02	TT2609C02	TT2609C02_THYROID		1240223	Male
ACH-000988	3.693779	MFE-319	MFE319	MFE319_ENDOMETRIUM		1240174	Female
ACH-000952	3.494664	MDA PCa 2b	MDAPCA2B	MDAPCA2B_PROSTATE		NA	Male
ACH-000065	3.817225	OCI-AML5	OCIAML5	OCIAML5_HAEMATOPOIETIC_AND_LYMPHOID_TISSUE		1330983	Male
ACH-000609	3.862763	SF126	SF126	SF126_CENTRAL_NERVOUS_SYSTEM		909712	Female
ACH-001411	3.896817	UM-UC-5	UMUC5	UMUC5_URINARY_TRACT		NA	Female
ACH-000504	4.078923	SNB75	SNB75	SNB75_CENTRAL_NERVOUS_SYSTEM		905982	Female

ACH-000956	3.3846	22Rv1	22RV1	22RV1_PROSTATE	22-RV1	924100	Male
ACH-001712	3.788704	Hs 860.T	HS860T	HS860T_BONE	HS-860, HS860	NA	Male
ACH-000652	4.011096	SUIT-2	SUIT2	SUIT2_PANCREAS		1240219	Male
ACH-000414	3.781126	NCI-H1944	NCIH1944	NCIH1944_LUNG		1240185	Female
ACH-000472	3.99564	HSC-2	HSC2	HSC2_UPPER_AERODIGESTIVE_TRACT		753562	Male
ACH-000007	3.699876	LS513	LS513	LS513_LARGE_INTESTINE		907795	Male
ACH-000446	3.445975	KP-N-SI9s	KPNSI9S	KPNSI9S_AUTONOMIC_GANGLIA		NA	Male
ACH-000386	3.74911	KG-1	KG1	KG1_HAEMATOPOIETIC_AND_LYMPHOID_TISSUE		907278	Male
ACH-001850	3.750079	G415	G415	G415_BILIARY_TRACT		NA	Male
ACH-000755	3.49848	HCC2218	HCC2218	HCC2218_BREAST		749716	Female
ACH-000028	3.584907	KPL-1	KPL1	KPL1_BREAST		NA	Female
ACH-000665	3.710354	SK-MES-1	SKMES1	SKMES1_LUNG		909728	Male
ACH-000078	2.884775	MHH-NB-11	MHHNB11	MHHNB11_AUTONOMIC_GANGLIA		908135	Male
ACH-002046	3.842153	HTMMT	HTMMT	HTMMT_ENDOMETRIUM		NA	Female
ACH-000397	3.728282	TEN	TEN	TEN_ENDOMETRIUM		NA	Female
ACH-000229	3.776077		HS822T	HS822T_FIBROBLAST	Hs 822.T	NA	Female
ACH-000518	3.929898	CAL-33	CAL33	CAL33_UPPER_AERODIGESTIVE_TRACT		753541	Male
ACH-001566	3.656748	MM370	MM370	MM370_SKIN		NA	Male
ACH-000252	3.782705	LS1034	LS1034	LS1034_LARGE_INTESTINE		917486	Male
ACH-000572	3.774556	G-361	G361	G361_SKIN		906865	Male
ACH-001605	3.69409	no.10	NO10	NO10_CENTRAL_NERVOUS_SYSTEM	ONDA10	908452	Male
ACH-000277	3.521784	HCC1419	HCC1419	HCC1419_BREAST		907045	Female

ACH-001239	3.965853	WM-266-4	WM2664	WM2664_SKIN		NA	Female
ACH-000786	3.59358	Daudi	DAUDI	DAUDI_HAEMATOPOIETIC_AND_LYMPHOID_TISSUE		906831	Male
ACH-000803	3.375738	COLO 668	COLO668	COLO668_LUNG		910692	Female
ACH-001970	3.552208	MM253	MM253	MM253_SKIN	MM 253, MM-253	NA	Male
ACH-002038	3.832477	HSKTC	HSKTC	HSKTC_OVARY		NA	Female
ACH-000537	3.846293	SNU-761	SNU761	SNU761_LIVER		NA	Male
ACH-000419	3.5606	KMS-28BM	KMS28BM	KMS28BM_HAEMATOPOIETIC_AND_LYMPHOID_TISSUE		NA	Female
ACH-000724	3.739235	HT-1376	HT1376	HT1376_URINARY_TRACT		907066	Female
ACH-000522	3.852047	UM-UC-3	UMUC3	UMUC3_URINARY_TRACT		724838	Male
ACH-001549	3.027299	Lu-135	LU135	LU135_LUNG		713899	Male
ACH-000706	3.671154	EKVX	EKVX	EKVX_LUNG		905970	Male
ACH-000737	3.283878	NCI-H1385	NCIH1385	NCIH1385_LUNG		NA	Female
ACH-000548	3.914105	BHY	BHY	BHY_UPPER_AERODIGESTIVE_TRACT		753535	Male
ACH-001839	3.662761	ICC13-7	ICC137	ICC137_BILIARY_TRACT	ICC13	NA	Unknown
ACH-001799	3.541299	LPS141	LPS141	LPS141_SOFT_TISSUE		NA	Unknown
ACH-001573	3.437413	MOLM-1	MOLM1	MOLM1_HAEMATOPOIETIC_AND_LYMPHOID_TISSUE		NA	Male
ACH-000425	3.778309	UACC-62	UACC62	UACC62_SKIN		905976	Unknown
ACH-000375	3.620187	G-402	G402	G402_SOFT_TISSUE		907298	Female
ACH-000753	3.837903	JMSU-1	JMSU1	JMSU1_URINARY_TRACT		NA	Male
ACH-000275	3.653372		HS834T	HS834T_FIBROBLAST		NA	Female
ACH-000735	4.045039	PE/CA-PJ49	PECAPJ49	PECAPJ49_UPPER_AERODIGESTIVE_TRACT		NA	Male
ACH-000654	3.697268	Raji	RAJI	RAJI_HAEMATOPOIETIC_AND_LYMPHOID_TISSUE		909262	Male

ACH-001067	3.785373	F5	F5	F5_CENTRAL_NERVOUS_SYSTEM		NA	Male
ACH-000170	3.883031	PrEC LH	PRECLH	PRECLH_PROSTATE		NA	Unknown
ACH-001394	3.838171	SUM-229PE	SUM229PE	SUM229PE_BREAST		NA	Female
ACH-000404	3.88963	K029AX	K029AX	K029AX_SKIN		NA	Unknown
ACH-000882	3.735701	IGR-1	IGR1	IGR1_SKIN		907169	Male
ACH-001509	3.672171	H357	H357	H357_UPPER_AERODIGESTIVE_TRACT		NA	Male
ACH-000210	3.375431	CADO-ES1	CADOES1	CADOES1_BONE	CADO-ES-1	753539	Female
ACH-000254	4.050646	SCC-15	SCC15	SCC15_UPPER_AERODIGESTIVE_TRACT		910911	Male
ACH-000604	3.564246	KYO-1	KYO1	KYO1_HAEMATOPOIETIC_AND_LYMPHOID_TISSUE		NA	Male
ACH-000090	3.663646	PC-3	PC3	PC3_PROSTATE		905934	Male
ACH-000195	3.85179	Set-2	SET2	SET2_HAEMATOPOIETIC_AND_LYMPHOID_TISSUE		NA	Female
ACH-001791	3.416803	LPS6	LPS6	LPS6_SOFT_TISSUE		NA	Unknown
ACH-001650	3.750579	SISO	SISO	SISO_CERVIX		1240212	Female
ACH-000605	3.608992	TE-6	TE6	TE6_OESOPHAGUS		946355	Male
ACH-000458	4.089527	CJM	CJM	CJM_SKIN		NA	Unknown
NA.7	NA	NA	NA	NA	NA	NA	NA
ACH-000004	3.633794	HEL	HEL	HEL_HAEMATOPOIETIC_AND_LYMPHOID_TISSUE		907053	Male
ACH-000251	3.794898		NCIH2887	NCIH2887_LUNG	NCI-H2887	NA	Male
ACH-000241	3.792554	JK-1	JK1	JK1_HAEMATOPOIETIC_AND_LYMPHOID_TISSUE		NA	Male
ACH-001194	3.864941	SK-RC 31	SKRC31	SKRC31_KIDNEY		NA	Unknown
NA.8	NA	NA	NA	NA	NA	NA	NA
ACH-001443	3.891563	ASH-3	ASH3	ASH3_THYROID		1290722	Female
ACH-000688	3.920374	OV7	OV7	OV7_OVARY		1480360	Female

ACH-000405	3.707769	MEC-1	MEC1	MEC1_HAEMATOPOIETIC_AND_LYMPHOID_TISSUE		NA	Male
ACH-000821	3.573372	EJM	EJM	EJM_HAEMATOPOIETIC_AND_LYMPHOID_TISSUE		1297447	Female
ACH-001677	3.392476	U-2904	U2904	U2904_HAEMATOPOIETIC_AND_LYMPHOID_TISSUE		NA	Male
ACH-001627	3.947653	P4E6	P4E6	P4E6_PROSTATE		NA	Male
ACH-000951	3.732909	NCI-H2342	NCIH2342	NCIH2342_LUNG		687819	Male
ACH-001036	3.586082	CMK-11-5	CMK115	CMK115_HAEMATOPOIETIC_AND_LYMPHOID_TISSUE		NA	Male
ACH-000547	3.90351	HT-1197	HT1197	HT1197_URINARY_TRACT		907065	Male
ACH-000826	3.996842	CAL-12T	CAL12T	CAL12T_LUNG		753540	Male
ACH-001147	3.464529		OCILY12	OCILY12_HAEMATOPOIETIC_AND_LYMPHOID_TISSUE		NA	Male
ACH-000162	3.639777	GA-10	GA10	GA10_HAEMATOPOIETIC_AND_LYMPHOID_TISSUE		1303896	Male
ACH-001751	3.145359	Rh36	RH36	RH36_SOFT_TISSUE		NA	Male
ACH-000018	3.814953	T24	T24	T24_URINARY_TRACT		724812	Female
ACH-000721	3.753217	HMC-1-8	HMC18	HMC18_BREAST		NA	Female
ACH-000003	3.748607	CACO2	CACO2	CACO2_LARGE_INTESTINE	CACO2, CaCo-2	NA	Male
ACH-000092	3.904569	NCI-H2452	NCIH2452	NCIH2452_PLEURA		908462	Male
ACH-000669	3.754256	SW 900	SW900	SW900_LUNG		724879	Male
ACH-001099	3.603446	KD	KD	KD_SOFT_TISSUE		NA	Unknown
ACH-000648	3.637866	NCI-H28	NCIH28	NCIH28_PLEURA		908470	Male
ACH-000950	3.733688	LoVo	LOVO	LOVO_LARGE_INTESTINE		907790	Male
ACH-000050	3.381345	NCI-H929	NCIH929	NCIH929_HAEMATOPOIETIC_AND_LYMPHOID_TISSUE		724825	Female
ACH-001106	3.804778	KOPN-8	KOPN8	KOPN8_HAEMATOPOIETIC_AND_LYMPHOID_TISSUE		1330933	Female
ACH-000367	3.8154	NCI-H226	NCIH226	NCIH226_LUNG		905941	Male

ACH-000589	3.582762	NCI-H1437	NCIH1437	NCIH1437_LUNG		687794	Male
ACH-000564	3.327689	KHM-1B	KHM1B	KHM1B_HAEMATOPOIETIC_AND_LYMPHOID_TISSUE		NA	Male
ACH-001522	3.758154	HMY-1	HMY1	HMY1_SKIN		NA	Male
ACH-000831	3.807523	HEC-50B	HEC50B	HEC50B_ENDOMETRIUM		NA	Female
ACH-000424	3.384437	TC-71	TC71	TC71_BONE		1240221	Male
ACH-000409	3.566891	OVSAHO	OVSAHO	OVSAHO_OVARY		NA	Female
ACH-000616	3.982242	Hs 746T	HS746T	HS746T_STOMACH		1240151	Male
ACH-000628	3.884033	NCI-H596	NCIH596	NCIH596_LUNG		908459	Male
ACH-000512	3.480428		INA6	INA6_HAEMATOPOIETIC_AND_LYMPHOID_TISSUE	INA-6	NA	Male
ACH-000232	3.759695	U-251 MG	U251MG	U251MG_CENTRAL_NERVOUS_SYSTEM	U251-MG	905983	Male
ACH-000820	3.855609	SW403	SW403	SW403_LARGE_INTESTINE		NA	Female
ACH-001295	2.894458	Y-79	Y79	Y79_AUTONOMIC_GANGLIA		NA	Female
ACH-001164	3.635188	CCLF_PEDS_0003_T	CCLFPEDS0003T	CCLFPEDS0003T_SOFT_TISSUE		NA	Unknown
ACH-000806	3.541828	L-540	L540	L540_HAEMATOPOIETIC_AND_LYMPHOID_TISSUE		907323	Female
ACH-000645	3.794607	JL-1	JL1	JL1_PLEURA		NA	Male
ACH-000099	3.017065	SIMA	SIMA	SIMA_AUTONOMIC_GANGLIA		753620	Male
ACH-000902	3.59854	CAL-148	CAL148	CAL148_BREAST		924106	Female
ACH-000649	3.795855	786-O	786O	786O_KIDNEY		905947	Male
ACH-000086	3.620493	ACC-MESO-1	ACCMESO1	ACCMESO1_PLEURA		NA	Female
ACH-000149	3.602296	SK-N-SH	SKNSH	SKNSH_AUTONOMIC_GANGLIA		717431	Female
ACH-000142	3.936873	CAL-29	CAL29	CAL29_URINARY_TRACT		1290730	Female
ACH-000227	2.990527	KP-N-YN	KPNYN	KPNYN_AUTONOMIC_GANGLIA		907314	Male

ACH-000697	3.440848	A3/KAW	A3KAW	A3KAW_HAEMATOPOIETIC_AND_LYMPHOID_TISSUE		910935	Female
ACH-000942	3.254576	HPB-ALL	HPBALL	HPBALL_HAEMATOPOIETIC_AND_LYMPHOID_TISSUE		NA	Male
ACH-000264	3.574258	Calu-6	CALU6	CALU6_LUNG		724859	Female
ACH-000809	3.765432	KYSE-410	KYSE410	KYSE410_OESOPHAGUS		753574	Male
ACH-000100	3.390969	RH-41	RH41	RH41_SOFT_TISSUE		1240210	Female
ACH-000060	3.771836	Panc 10.05	PANC1005	PANC1005_PANCREAS		925348	Male
ACH-000001	3.611756	NIH:OVCAR-3	NIHOVCAR3	NIHOVCAR3_OVARY	OVCAR3	905933	Female
ACH-000957	3.874486	LS 180	LS180	LS180_LARGE_INTESTINE		998189	Female
ACH-000650	3.717502	IGR-37	IGR37	IGR37_SKIN		1240153	Male
ACH-001096	3.309854	JR	JR	JR_SOFT_TISSUE	JR-1	NA	Unknown
ACH-000239	3.853721	HuG1-N	HUG1N	HUG1N_STOMACH		NA	Male
ACH-000762	3.956052	YD-38	YD38	YD38_UPPER_AERODIGESTIVE_TRACT		NA	Male
ACH-000922	3.743213	RCH-ACV	RCHACV	RCHACV_HAEMATOPOIETIC_AND_LYMPHOID_TISSUE		1330994	Female
ACH-000859	3.915575	HCC1954	HCC1954	HCC1954_BREAST		749709	Female
ACH-000990	3.466627	HEC-108	HEC108	HEC108_ENDOMETRIUM		NA	Female
ACH-000410	3.75201	Saos-2	SAOS2	SAOS2_BONE		909707	Female
ACH-000927	3.627985	BT-474	BT474	BT474_BREAST		946359	Female
ACH-001853	3.472618	KMCH-1	KMCH1	KMCH1_BILIARY_TRACT		NA	Male
ACH-000694	3.870785	TE-9	TE9	TE9_OESOPHAGUS		946353	Male
ACH-000935	4.001062	MDST8	MDST8	MDST8_LARGE_INTESTINE		1240173	Unknown
ACH-000918	3.450478	MOLT-16	MOLT16	MOLT16_HAEMATOPOIETIC_AND_LYMPHOID_TISSUE		908147	Female
ACH-000761	4.000098	NUGC-2	NUGC2	NUGC2_STOMACH		NA	Female

ACH-001563	3.781641	MM127	MM127	MM127_SKIN		NA	Male
ACH-000974	3.62029	SNG-M	SNGM	SNGM_ENDOMETRIUM		909735	Female
ACH-000297	3.187038	NCI-H889	NCIH889	NCIH889_LUNG		NA	Female
ACH-001719	3.755537	OCI-C4P	OCIC4P	OCIC4P_OVARY		NA	Female
ACH-000243	3.953688	DAN-G	DANG	DANG_PANCREAS		1290797	Female
ACH-001418	3.64894	UWB1.289	UWB1289	UWB1289_OVARY		1480374	Female
ACH-000362	4.002161	MOLM-13	MOLM13	MOLM13_HAEMATOPOIETIC_AND_LYMPHOID_TISSUE		1330947	Male
ACH-000843	3.684449	HARA	HARA	HARA_LUNG		1240142	Male
ACH-000840	4.114938	HCC-366	HCC366	HCC366_LUNG		1240144	Female
ACH-001852	3.854851	HKGZ-CC	HKGZCC	HKGZCC_BILIARY_TRACT		NA	Male
ACH-001528	3.774036	IHH-4	IHH4	IHH4_THYROID		1240154	Male
ACH-001656	3.834643	SKNO-1	SKNO1	SKNO1_HAEMATOPOIETIC_AND_LYMPHOID_TISSUE		NA	Male
ACH-000016	3.706731	SLR 21	SLR21	SLR21_KIDNEY		NA	Unknown
ACH-000165	3.906198		HS751T	HS751T_FIBROBLAST		NA	Male
ACH-001460	3.818332	C84	C84	C84_LARGE_INTESTINE		NA	Male
ACH-000592	3.71471	TM-31	TM31	TM31_CENTRAL_NERVOUS_SYSTEM		NA	Female
ACH-001192	3.269167	SK-NEP-1	SKNEP1	SKNEP1_BONE	HTB-48	NA	Female
ACH-000045	3.873608	MV4;11	MV411	MV411_HAEMATOPOIETIC_AND_LYMPHOID_TISSUE	MV-4-11	908156	Male
ACH-000741	3.849039	U-BLC1	UBLC1	UBLC1_URINARY_TRACT		NA	Female
ACH-000912	3.660734	NCI-H2286	NCIH2286	NCIH2286_LUNG		NA	Female
ACH-000529	3.782549	T1-73	T173	T173_FIBROBLAST		NA	Unknown
ACH-000508	3.375489	COR-L88	CORL88	CORL88_LUNG		906808	Male

ACH-000577	3.943886	JHH-2	JHH2	JHH2_LIVER	1240157	Male
ACH-000768	3.934912	MDA-MB-231	MDAMB231	MDAMB231_BREAST	905960	Female
ACH-000636	3.308202	RPMI-8402	RPMI8402	RPMI8402_HAEMATOPOIETIC_AND_LYMPHOID_TISSUE	909702	Female
ACH-000667	3.88775	HCC-44	HCC44	HCC44_LUNG	1240145	Female
ACH-000905	4.066516	5637	5637	5637_URINARY_TRACT	687452	Male
ACH-001494	3.590996	EGI-1	EGI1	EGI1_BILIARY_TRACT	906853	Male
ACH-000387	3.76326	TF-1	TF1	TF1_HAEMATOPOIETIC_AND_LYMPHOID_TISSUE	NA	Male
ACH-002510	3.709307	M040416	M040416	M040416_SKIN	NA	Male
ACH-001303	2.870622	NB1643	NB1643	NB1643_AUTONOMIC_GANGLIA	NA	Male
ACH-000026	3.748284	253J-BV	253JBV	253JBV_URINARY_TRACT	NA	Male
ACH-000838	3.547217	AMO-1	AMO1	AMO1_HAEMATOPOIETIC_AND_LYMPHOID_TISSUE	1295741	Female
ACH-001172	3.709544		R256	R256_CENTRAL_NERVOUS_SYSTEM	NA	Unknown
ACH-000822	4.072044	SK-MEL-24	SKMEL24	SKMEL24_SKIN	909725	Male
ACH-000531	3.888791		RS5	RS5_FIBROBLAST	NA	Male
ACH-000349	3.456863	HCC1500	HCC1500	HCC1500_BREAST	1303900	Female
ACH-000527	3.667181	OVISE	OVISE	OVISE_OVARY	1240198	Female
ACH-000279	3.326902	EWS502	EWS502	EWS502_BONE	NA	Unknown
ACH-000516	3.741544	CAL-78	CAL78	CAL78_BONE	1290765	Male
ACH-000812	4.096718	COLO-783	COLO783	COLO783_SKIN	1240125	Female
ACH-001300	3.066149	CHLA15	CHLA15	CHLA15_AUTONOMIC_GANGLIA	NA	Female
ACH-000524	3.554158	KURAMOCHI	KURAMOCHI	KURAMOCHI_OVARY	909975	Female
ACH-000611	3.678595	SU-DHL-6	SUDHL6	SUDHL6_HAEMATOPOIETIC_AND_LYMPHOID_TISSUE	1331037	Male

ACH-000411	3.875311	769-P	769P	769P_KIDNEY		910922	Female
ACH-000718	3.78165	NCI-H2291	NCIH2291	NCIH2291_LUNG		724874	Male
ACH-000903	3.789537	FTC-133	FTC133	FTC133_THYROID		906864	Male
NA.9	NA	NA	NA	NA	NA	NA	NA
ACH-000433	3.742025	Caki-1	CAKI1	CAKI1_KIDNEY		905963	Male
ACH-000499	3.300005	EW8	EW8	EW8_BONE		NA	Male
ACH-000278	3.647794	COV362	COV362	COV362_OVARY		NA	Female
ACH-000202	3.367947	COLO-320	COLO320	COLO320_LARGE_INTESTINE		NA	Female
ACH-001736	3.562313	HB1119	HB1119	HB1119_HAEMATOPOIETIC_AND_LYMPHOID_TISSUE		NA	Unknown
ACH-001379	3.791102	PACADD-161	PACADD161	PACADD161_PANCREAS		NA	Female
ACH-000128	3.452604	LN-319	LN319	LN319_CENTRAL_NERVOUS_SYSTEM		NA	Male
ACH-000696	3.750998	OVCAR-8	OVCAR8	OVCAR8_OVARY		905991	Female
ACH-000041	3.326378	RD-ES	RDES	RDES_BONE		NA	Male
ACH-000214	3.852381		HS675T	HS675T_FIBROBLAST		NA	Male
ACH-000747	3.818628	NCI-H1703	NCIH1703	NCIH1703_LUNG		908474	Male
ACH-001307	3.94608	8505C	8505C	8505C_THYROID		924102	Female
ACH-000341	3.153535	SK-N-FI	SKNFI	SKNFI_AUTONOMIC_GANGLIA		688087	Male
ACH-001740	3.273599	RH28	RH28	RH28_SOFT_TISSUE		NA	Male
ACH-000417	4.018832	Panc 08.13	PANC0813	PANC0813_PANCREAS		925347	Male
ACH-000120	3.463784	CHP-212	CHP212	CHP212_AUTONOMIC_GANGLIA		906820	Male
ACH-001694	3.877869	UPCI-SCC-074	UPCISCC074	UPCISCC074_UPPER_AERODIGESTIVE_TRACT		NA	Female
ACH-001542	3.8392	KON	KON	KON_UPPER_AERODIGESTIVE_TRACT		1298215	Male
ACH-002509	3.756186	WM4235	WM4235	WM4235_SKIN		NA	Unknown

ACH-000438	3.822214		LU65	LU65_LUNG		724863	Male
ACH-001366	2.923965	NGP	NGP	NGP_AUTONOMIC_GANGLIA		NA	Male
ACH-000532	3.930416	SNU-61	SNU61	SNU61_LARGE_INTESTINE		1660035	Male
ACH-000653	3.596465	JJN-3	JJN3	JJN3_HAEMATOPOIETIC_AND_LYMPHOID_TISSUE		1327766	Female
ACH-000839	4.211326	SCaBER	SCABER	SCABER_URINARY_TRACT		NA	Male
ACH-000673	3.877066	LN-443	LN443	LN443_CENTRAL_NERVOUS_SYSTEM		NA	Unknown
ACH-001524	3.7083	HSC-5	HSC5	HSC5_SKIN		NA	Male
ACH-000069	4.029569	Hs 611.T	HS611T	HS611T_HAEMATOPOIETIC_AND_LYMPHOID_TISSUE		NA	Female
ACH-000323	3.662721	42-MG-BA	42MGBA	42MGBA_CENTRAL_NERVOUS_SYSTEM		687561	Male
ACH-000021	3.557531	NCI-H1693	NCIH1693	NCIH1693_LUNG		687802	Female
ACH-001863	3.739838	TKKK	TKKK	TKKK_BILIARY_TRACT	TKKK	NA	Male
ACH-000980	3.419026	NCI-H1155	NCIH1155	NCIH1155_LUNG		908467	Male
ACH-000106	3.920921	JVM-2	JVM2	JVM2_HAEMATOPOIETIC_AND_LYMPHOID_TISSUE		907269	Female
ACH-000734	3.694319	JHH-5	JHH5	JHH5_LIVER		NA	Male
ACH-000845	3.830011	NCI-H1373	NCIH1373	NCIH1373_LUNG		NA	Male
NA.10	NA	NA	NA	NA	NA	NA	NA
ACH-000730	3.912016	SK-MEL-5	SKMEL5	SKMEL5_SKIN		905956	Female
ACH-001334	3.901408	C-4 I	C4I	C4I_CERVIX		687506	Female
ACH-000698	3.478979	DMS 53	DMS53	DMS53_LUNG		907295	Male
ACH-000802	3.992234	BFTC-905	BFTC905	BFTC905_URINARY_TRACT		910926	Female
ACH-001836	3.754725	ICC10-8	ICC108	ICC108_BILIARY_TRACT		NA	Unknown
ACH-000573	3.988106	MDA-MB-436	MDAMB436	MDAMB436_BREAST		1240172	Female
ACH-001699	3.930764	UPCI-SCC-131	UPCISCC131	UPCISCC131_UPPER_AERODIGESTIVE_TRACT		NA	Male

ACH-000963	3.790827	CCK-81	CCK81	CCK81_LARGE_INTESTINE		1240123	Female
ACH-001567	3.775671	MM383	MM383	MM383_SKIN		NA	Male
ACH-001653	3.81583	SK-GT-2	SKGT2	SKGT2_STOMACH		1503364	Male
ACH-000852	3.909684	NCI-H1435	NCIH1435	NCIH1435_LUNG		1298347	Female
ACH-001862	3.904105	TGBC52TKB	TGBC52TKB	TGBC52TKB_BILIARY_TRACT	TGBC52TKB	NA	Male
ACH-000661	3.995356	WM1799	WM1799	WM1799_SKIN		NA	Male
ACH-001500	3.863403	FLO-1	FLO1	FLO1_OESOPHAGUS		1503361	Male
ACH-001190	3.795812	SK-MEL-2	SKMEL2	SKMEL2_SKIN		905955	Male
ACH-002467	3.71026	RPE1-ss51	RPE1SS51	RPE1SS51_ENGINEERED		NA	Female
ACH-000364	3.511236	U-2 OS	U2OS	U2OS_BONE	U2-OS, U-2 OS	909776	Female
ACH-000448	3.758868	NCI-H1666	NCIH1666	NCIH1666_LUNG		908473	Female
ACH-000932	3.444945	SNU-1	SNU1	SNU1_STOMACH		908444	Male
ACH-000783	3.460316	CAMA-1	CAMA1	CAMA1_BREAST		946382	Female
ACH-002044	3.299204	HSQ-89	HSQ89	HSQ89_UPPER_AERODIGESTIVE_TRACT		NA	Male
ACH-000301	3.774393	LAMA-84	LAMA84	LAMA84_HAEMATOPOIETIC_AND_LYMPHOID_TISSUE		907783	Female
ACH-002459	3.96376	HT144 SKIN FV3	HT144SKINFV3	HT144_SKIN_FV3_RESISTANT	HT144_Dab_Tram	NA	Male
ACH-000976	3.975712	HuCCT1	HUCCT1	HUCCT1_BILIARY_TRACT		907069	Male
ACH-001516	3.340654	HCSC-1	HCSC1	HCSC1_CERVIX		NA	Female
ACH-001354	2.957412	LAN-2	LAN2	LAN2_AUTONOMIC_GANGLIA		NA	Female
ACH-001645	3.730095	SEKI	SEKI	SEKI_SKIN		NA	Female
ACH-000867	3.6755	ChaGo-K-1	CHAGOK1	CHAGOK1_LUNG		687596	Male
ACH-000304	3.92528	WM-115	WM115	WM115_SKIN		909784	Female

ACH-002069	3.406057	HS-Os-1	HSOS1	HSOS1_BONE		NA	Female
ACH-000931	3.543064	HMCB	HMCB	HMCB_SKIN	CHL-1	NA	Female
ACH-001374	3.320658	PA-1 [PA1]	PA1	PA1_OVARY		909255	Female
ACH-000302	3.669968	SNU-1077	SNU1077	SNU1077_ENDOMETRIUM		NA	Female
ACH-000305	3.821407	EC-GI-10	ECGI10	ECGI10_OESOPHAGUS		753555	Male
ACH-000072	3.772927	MEG-01	MEG01	MEG01_HAEMATOPOIETIC_AND_LYMPHOID_TISSUE		908126	Male
ACH-000766	4.100783	NCI-H1648	NCIH1648	NCIH1648_LUNG		687799	Male
ACH-000533	3.634161	NCI-H2004 RT	NCIH2004RT	NCIH2004RT_SOFT_TISSUE		NA	Unknown
ACH-000343	3.321349	NCI-H522	NCIH522	NCIH522_LUNG		905944	Male
ACH-000171	3.747461	VMRC-RCZ	VMRCRCZ	VMRCRCZ_KIDNEY		909781	Unknown
ACH-000173	4.020049	JHUEM-3	JHUEM3	JHUEM3_ENDOMETRIUM		NA	Female
ACH-000535	4.128811	BxPC-3	BXPC3	BXPC3_PANCREAS		906693	Female
ACH-000774	4.042058	RERF-LC-Ad2	RERFLCAD2	RERFLCAD2_LUNG		NA	Male
ACH-000178	3.952651	Hs 766T	HS766T	HS766T_PANCREAS		1298141	Male
ACH-001306	3.943842	8305C	8305C	8305C_THYROID		906795	Female
ACH-000994	3.797481	HEC-59	HEC59	HEC59_ENDOMETRIUM		NA	Female
ACH-001188	2.860219	SH-SY5Y	SHSY5Y	SHSY5Y_AUTONOMIC_GANGLIA		NA	Female
ACH-000372	3.268217	P12-ICHIKAWA	P12ICHIKAWA	P12ICHIKAWA_HAEMATOPOIETIC_AND_LYMPHOID_TISSUE		909251	Male
ACH-000804	3.251515	NB-1	NB1	NB1_AUTONOMIC_GANGLIA		949179	Male
ACH-000011	3.638472	253J	253J	253J_URINARY_TRACT		NA	Male
ACH-000733	3.839392	NCI-H1838	NCIH1838	NCIH1838_LUNG		687807	Female
ACH-001603	3.046267	NH-12	NH12	NH12_AUTONOMIC_GANGLIA		908447	Male

ACH-000829	3.671203	HuNS1	HUNS1	HUNS1_HAEMATOPOIETIC_AND_LYMPHOID_TISSUE		NA	Male
ACH-000141	3.893302	SNU-308	SNU308	SNU308_BILIARY_TRACT		NA	Unknown
ACH-000810	3.974847	SK-MEL-30	SKMEL30	SKMEL30_SKIN		909726	Male
ACH-001145	3.517945	OC 316	OC316	OC316_OVARY		NA	Female
ACH-000717	3.844064	COLO-680N	COLO680N	COLO680N_OESOPHAGUS		906817	Female
ACH-000738	3.768004	GB-1	GB1	GB1_CENTRAL_NERVOUS_SYSTEM		687568	Male
ACH-001843	3.707733	ICC3	ICC3	ICC3_BILIARY_TRACT		NA	Unknown
ACH-000780	3.24027	NCI-H1105	NCIH1105	NCIH1105_LUNG		908468	Male
ACH-001548	3.04134	LS	LS	LS_AUTONOMIC_GANGLIA		NA	Female
ACH-000759	3.710182	MDA-MB-175-VII	MDAMB175VII	MDAMB175VII_BREAST		908120	Female
ACH-001555	3.718861	Mero-14	MERO14	MERO14_PLEURA		NA	Male
ACH-001369	3.715358	OCI-C5x	OCIC5X	OCIC5X_OVARY		NA	Female
ACH-000969	3.628521	KM12	KM12	KM12_LARGE_INTESTINE		905989	Unknown
ACH-000873	3.795562	KYSE-270	KYSE270	KYSE270_OESOPHAGUS		907319	Male
ACH-001275	3.370211	SYO1	SYO1	SYO1_SOFT_TISSUE		NA	Female
ACH-000921	3.902181	NCI-H1339	NCIH1339	NCIH1339_LUNG		NA	Female
ACH-001373	3.459275	OV17R	OV17R	OV17R_OVARY		1480361	Female
ACH-001022	3.14686	CBAGPN	CBAGPN	CBAGPN_BONE	CB-AGPN	NA	Unknown
ACH-000082	3.769701	G-292, clone A141B1	G292CLONEA141B1	G292CLONEA141B1_BONE	G292CLONEA141B1, G-292 clone A141B1	1290807	Female
ACH-000488	4.055122	TE-11	TE11	TE11_OESOPHAGUS		946354	Male
ACH-000790	3.351528	SHP-77	SHP77	SHP77_LUNG		724872	Male
ACH-000172	3.568573	TM-87	TM87	TM87_SOFT_TISSUE	TM87	NA	Unknown

ACH-000647	3.875777	TE-1	TE1	TE1_OESOPHAGUS		753621	Male
ACH-000602	3.788225	M-07e	M07E	M07E_HAEMATOPOIETIC_AND_LYMPHOID_TISSUE		NA	Female
ACH-000156	3.685677	MHH-CALL-4	MHHCALL4	MHHCALL4_HAEMATOPOIETIC_AND_LYMPHOID_TISSUE		908133	Male
ACH-002001	3.752164	A375 SKIN CJ1	A375SKINCJ1	A375_SKIN_CJ1_RESISTANT	A375 RMR	NA	Unknown
ACH-000315	3.588992	KARPAS-422	KARPAS422	KARPAS422_HAEMATOPOIETIC_AND_LYMPHOID_TISSUE		907274	Female
ACH-002446	3.763957	CCLF_UPGI_0005_T	CCLFUPGI0005T	CCLFUPGI0005T_STOMACH		NA	Unknown
ACH-000201	3.890551	A-204	A204	A204_SOFT_TISSUE		910784	Female
ACH-000435	3.823668	EFE-184	EFE184	EFE184_ENDOMETRIUM		NA	Female
ACH-000965	3.85632	RL95-2	RL952	RL952_ENDOMETRIUM		930082	Female
ACH-001550	3.800786	MCC13	MCC13	MCC13_SKIN		NA	Female
ACH-002026	3.805154	HHUA	HHUA	HHUA_ENDOMETRIUM		NA	Female
ACH-000883	4.041466	SW 1783	SW1783	SW1783_CENTRAL_NERVOUS_SYSTEM		909750	Male
ACH-000482	3.653835	RERF-LC-KJ	RERFLCKJ	RERFLCKJ_LUNG		1298537	Male
ACH-000450	3.799137	MEL-HO	MELHO	MELHO_SKIN		908124	Female
ACH-000835	3.939344	GCT	GCT	GCT_SOFT_TISSUE		906999	Male
ACH-001802	3.551048	LPS853	LPS853	LPS853_SOFT_TISSUE		NA	Unknown
ACH-000629	3.772314		IOMMLEE	IOMMLEE_CENTRAL_NERVOUS_SYSTEM	IOMM-LEE	NA	Male
ACH-000715	3.880879	SNU-1214	SNU1214	SNU1214_UPPER_AERODIGESTIVE_TRACT		NA	Male
ACH-000870	3.169145	NCI-H1930	NCIH1930	NCIH1930_LUNG		NA	Male
ACH-000874	3.372361	RS4;11	RS411	RS411_HAEMATOPOIETIC_AND_LYMPHOID_TISSUE		909703	Female
ACH-000910	3.519067	MDA-MB-453	MDAMB453	MDAMB453_BREAST		908122	Female
ACH-000779	3.878301		PC9	PC9_LUNG		NA	Male

ACH-000140	3.503469	Pfeiffer	PFEIFFER	PFEIFFER_HAEMATOPOIETIC_AND_LYMPHOID_TISSUE		NA	Male
ACH-001196	3.349384	SMS-CTR	SMSCTR	SMSCTR_SOFT_TISSUE		NA	Male
ACH-001484	3.913872	CI	CI	CI_HAEMATOPOIETIC_AND_LYMPHOID_TISSUE		NA	Female
ACH-000693	3.848982	KYSE-180	KYSE180	KYSE180_OESOPHAGUS		907318	Male
ACH-000811	3.744624	SK-OV-3	SKOV3	SKOV3_OVARY		905959	Female
ACH-000101	3.394699	KE-37	KE37	KE37_HAEMATOPOIETIC_AND_LYMPHOID_TISSUE		907277	Male
ACH-000423	3.770067	SK-MEL-3	SKMEL3	SKMEL3_SKIN		909724	Female
ACH-000290	3.142046	NCI-H209	NCIH209	NCIH209_LUNG	H209	688013	Male
ACH-000492	3.70809	MUTZ-5	MUTZ5	MUTZ5_HAEMATOPOIETIC_AND_LYMPHOID_TISSUE		NA	Male
ACH-001519	3.427335	H-EMC-SS	HEMCSS	HEMCSS_BONE		907290	Female
ACH-000110	3.505344	NCC-StC-K140	NCCSTCK140	NCCSTCK140_STOMACH		NA	Female
ACH-001497	4.018243	ESO51	ESO51	ESO51_OESOPHAGUS		1503367	Male
ACH-000102	3.923554	GMS-10	GMS10	GMS10_CENTRAL_NERVOUS_SYSTEM		906873	Male
ACH-000817	3.659887	RPMI 8226	RPMI8226	RPMI8226_HAEMATOPOIETIC_AND_LYMPHOID_TISSUE		905964	Male
ACH-001556	3.717401	Mero-25	MERO25	MERO25_PLEURA		NA	Male
ACH-000964	3.14861	MOLT-3	MOLT3	MOLT3_HAEMATOPOIETIC_AND_LYMPHOID_TISSUE		NA	Male
ACH-000378	3.898265	NCI-H647	NCIH647	NCIH647_LUNG		1240191	Male
ACH-000913	4.076375	ESS-1	ESS1	ESS1_ENDOMETRIUM		907000	Female
ACH-000575	3.861861		HCC364	HCC364_LUNG		NA	Male
ACH-000634	3.740512	LN-340	LN340	LN340_CENTRAL_NERVOUS_SYSTEM		NA	Male
ACH-001322	3.501951	CME-1	CME1	CME1_SOFT_TISSUE		NA	Unknown
ACH-000767	3.334698	NCI-H526	NCIH526	NCIH526_LUNG		688025	Male

ACH-001642	3.14139	SCC-3	SCC3	SCC3_HAEMATOPOIETIC_AND_LYMPHOID_TISSUE		910930	Female
ACH-001552	3.642128	MCC26	MCC26	MCC26_SKIN		NA	Female
ACH-000451	3.836229	NCI-H2085	NCIH2085	NCIH2085_LUNG		687812	Male
ACH-000879	3.654202	MFE-296	MFE296	MFE296_ENDOMETRIUM		908130	Female
ACH-001842	3.645001	ICC2	ICC2	ICC2_BILIARY_TRACT		NA	Unknown
ACH-001032	3.072243	CHLA-32	CHLA32	CHLA32_BONE		NA	Female
ACH-000982	3.767409	GP2d	GP2D	GP2D_LARGE_INTESTINE		NA	Female
ACH-001636	3.252	Ramos	RAMOS	RAMOS_HAEMATOPOIETIC_AND_LYMPHOID_TISSUE	RA 1	NA	Male
ACH-000189	3.795696	RCC10RGB	RCC10RGB	RCC10RGB_KIDNEY		909974	Male
ACH-000454	3.71846	HCC-95	HCC95	HCC95_LUNG		NA	Male
ACH-000131	3.691366	Hs 229.T	HS229T	HS229T_FIBROBLAST		NA	Male
ACH-000660	3.727979	SU-DHL-5	SUDHL5	SUDHL5_HAEMATOPOIETIC_AND_LYMPHOID_TISSUE		1331036	Female
ACH-001197	3.558158		SMZ1	SMZ1_HAEMATOPOIETIC_AND_LYMPHOID_TISSUE	SMZ-1	NA	Male
ACH-000915	4.03141	IPC-298	IPC298	IPC298_SKIN		907171	Female
ACH-000480	3.694177	HuH-7	HUH7	HUH7_LIVER		907071	Male
ACH-000745	3.276814	MOLP-8	MOLP8	MOLP8_HAEMATOPOIETIC_AND_LYMPHOID_TISSUE		1330950	Male
ACH-000161	3.744099	COR-L105	CORL105	CORL105_LUNG		906805	Male
ACH-000395	3.391756	NCI-H520	NCIH520	NCIH520_LUNG		908443	Male
ACH-001459	3.588148	C80	C80	C80_LARGE_INTESTINE		NA	Male
ACH-000916	3.696548	NCI-H1573	NCIH1573	NCIH1573_LUNG		908472	Female
ACH-001609	3.833503	NP 3	NP3	NP3_CENTRAL_NERVOUS_SYSTEM		NA	Male
ACH-001960	4.026303	CC-SW-1	CCSW1	CCSW1_BILIARY_TRACT		NA	Female

ACH-001856	3.66574	RBE	RBE	RBE_BILIARY_TRACT		NA	Female
ACH-000295	3.936876	EM-2	EM2	EM2_HAEMATOPOIETIC_AND_LYMPHOID_TISSUE		906855	Female
ACH-001485	3.661416	CII	CII	CII_HAEMATOPOIETIC_AND_LYMPHOID_TISSUE		NA	Female
ACH-000292	3.309541	NCI-H841	NCIH841	NCIH841_LUNG		1240192	Male
ACH-000137	3.6625	8-MG-BA	8MGBA	8MGBA_CENTRAL_NERVOUS_SYSTEM		687562	Female
ACH-000308	3.864903	EFO-21	EFO21	EFO21_OVARY		911905	Female
ACH-000445	3.715018	KNS-60	KNS60	KNS60_CENTRAL_NERVOUS_SYSTEM		NA	Male
ACH-001229	3.734587	UPCI-SCC-154	UPCISCC154	UPCISCC154_UPPER_AERODIGESTIVE_TRACT	UPCISCC154, UPCI:SCC154	NA	Male
ACH-000144	3.495424	RERF-GC-1B	RERFGC1B	RERFGC1B_STOMACH		1240209	Male
ACH-000837	3.821018	NCI-H322	NCIH322	NCIH322_LUNG		NA	Male
ACH-000048	3.428026	TOV-112D	TOV112D	TOV112D_OVARY		1299070	Female
ACH-000463	3.699821	NCI-H460	NCIH460	NCIH460_LUNG		905943	Male
ACH-000881	3.922761	MEL-JUSO	MELJUSO	MELJUSO_SKIN		908125	Female
ACH-000388	3.674901	NU-DHL-1	NUDHL1	NUDHL1_HAEMATOPOIETIC_AND_LYMPHOID_TISSUE		NA	Male
NA.11	NA	NA	NA	NA	NA	NA	NA
ACH-000147	3.39813	T-47D	T47D	T47D_BREAST		905945	Female
ACH-000930	3.944707	HCC1569	HCC1569	HCC1569_BREAST		907046	Female
ACH-000899	3.894743	WM-88	WM88	WM88_SKIN		NA	Male
ACH-000366	3.157091	SK-N-DZ	SKNDZ	SKNDZ_AUTONOMIC_GANGLIA		688086	Female
ACH-001526	3.379331	HuO9	HUO9	HUO9_BONE		907072	Female
ACH-000064	3.788474	SALE	SALE	SALE_LUNG		NA	Unknown
ACH-001329	3.580541	ANGM-CSS	ANGMCSS	ANGMCSS_CENTRAL_NERVOUS_SYSTEM		NA	Male
ACH-000418	3.930969	SW 1353	SW1353	SW1353_BONE		NA	Female

ACH-001280	3.42293	SCS-214	SCS214	SCS214_SOFT_TISSUE	NA	Unknown
ACH-000861	3.771067	HOP-62	HOP62	HOP62_LUNG	905972	Female
ACH-000692	3.928269	SNU-899	SNU899	SNU899_UPPER_AERODIGESTIVE_TRACT	NA	Male
ACH-000107	3.89903	Capan-2	CAPAN2	CAPAN2_PANCREAS	910915	Male
ACH-000133	3.802852	Hs 729	HS729	HS729_SOFT_TISSUE	NA	Male
ACH-000306	3.78261	Hs 688(A).T	HS688AT	HS688AT_FIBROBLAST	NA	Male
ACH-001530	3.411207	JEG-3	JEG3	JEG3_PLACENTA	907176	Male
ACH-000651	3.555519	SW 620	SW620	SW620_LARGE_INTESTINE	905962	Male
ACH-000563	3.842653	EBC-1	EBC1	EBC1_LUNG	753554	Male
ACH-000058	3.735725	ML-1	ML1	ML1_THYROID	1240178	Unknown
ACH-000345	2.976775	KP-N-RT-BM-1	KPNRTBM1	KPNRTBM1_AUTONOMIC_GANGLIA	924189	Female
ACH-000316	3.875326	SNU-886	SNU886	SNU886_LIVER	NA	Male
ACH-000884	3.88023	MDA-MB-435S	MDAMB435S	MDAMB435S_SKIN	NA	Unknown
ACH-000289	3.789471	SNU-466	SNU466	SNU466_CENTRAL_NERVOUS_SYSTEM	NA	Male
ACH-001451	3.760744	BOKU	BOKU	BOKU_CERVIX	NA	Female
ACH-000355	3.055121	NCI-H82	NCIH82	NCIH82_LUNG	688031	Male
ACH-000962	3.426364	OC 314	OC314	OC314_OVARY	909257	Female
ACH-000473	3.878357	RT-112	RT112	RT112_URINARY_TRACT	909704	Female
ACH-000192	3.36657	MFE-280	MFE280	MFE280_ENDOMETRIUM	908129	Female
ACH-001624	3.423857	Onda 9	ONDA9	ONDA9_CENTRAL_NERVOUS_SYSTEM	NA	Male
ACH-001807	3.690684	LPS067	LPS067	LPS067_SOFT_TISSUE	NA	Unknown
ACH-002466	3.784801	RPE1-ss111	RPE1SS111	RPE1SS111_ENGINEERED	NA	Female

ACH-001344	3.624976	GI-ME-N	GIMEN	GIMEN_AUTONOMIC_GANGLIA	906872	Male
ACH-000757	3.579392	A427	A427	A427_LUNG	910851	Male
ACH-000054	3.777578	HT-1080	HT1080	HT1080_SOFT_TISSUE	907064	Male
ACH-001318	3.755978	PLC/PRF/5	PLCPRF5	PLCPRF5_LIVER	NA	Male
ACH-000601	3.71922	MIA PaCa-2	MIAPACA2	MIAPACA2_PANCREAS	724870	Male
ACH-000635	3.544238	SNU-119	SNU119	SNU119_OVARY	NA	Female
ACH-001857	3.716405	SG231	SG231	SG231_BILIARY_TRACT	NA	Male
ACH-000966	3.676301	IGROV1	IGROV1	IGROV1_OVARY	905968	Female
ACH-000630	3.856012	YD-8	YD8	YD8_UPPER_AERODIGESTIVE_TRACT	NA	Female
ACH-000225	3.207893	ECC12	ECC12	ECC12_STOMACH	906849	Male
ACH-000657	3.440477	A2780	A2780	A2780_OVARY	906804	Female
ACH-000624	3.734237	HCC1806	HCC1806	HCC1806_BREAST	907047	Female
ACH-000699	3.900283	HCC1395	HCC1395	HCC1395_BREAST	749712	Female
ACH-000923	3.469148	BCP-1	BCP1	BCP1_HAEMATOPOIETIC_AND_LYMPHOID_TISSUE	NA	Male
ACH-000687	3.88859	CI-1	CI1	CI1_HAEMATOPOIETIC_AND_LYMPHOID_TISSUE	NA	Female
ACH-000044	3.064641	MDA-MB-134-VI	MDAMB134VI	MDAMB134VI_BREAST	NA	Female
ACH-000740	3.95722	A-253	A253	A253_SALIVARY_GLAND	906794	Male
ACH-000447	4.090094	NCI-H2228	NCIH2228	NCIH2228_LUNG	687816	Female
ACH-001416	3.722446	UM-UC9	UMUC9	UMUC9_URINARY_TRACT	NA	Male
ACH-000319	3.718692	MPP 89	MPP89	MPP89_PLEURA	908150	Male
ACH-000198	3.838363	EOL-1	EOL1	EOL1_HAEMATOPOIETIC_AND_LYMPHOID_TISSUE	906856	Male
ACH-000280	3.7327	SNU-840	SNU840	SNU840_OVARY	NA	Female

ACH-000104	3.590472	Loucy	LOUCY	LOUCY_HAEMATOPOIETIC_AND_LYMPHOID_TISSUE		907789	Female
ACH-001835	3.602054	ICC10-6	ICC106	ICC106_BILIARY_TRACT		NA	Unknown
ACH-000892	3.78885	NCI-H1563	NCIH1563	NCIH1563_LUNG		753600	Male
ACH-000493	4.013155	SNU-423	SNU423	SNU423_LIVER		909737	Male
ACH-000042	3.737243	Panc 02.03	PANC0203	PANC0203_PANCREAS		1298475	Female
ACH-002045	3.867264	HO-1-u-1	HO1U1	HO1U1_UPPER_AERODIGESTIVE_TRACT		NA	Male
ACH-000221	3.478374	SNU-398	SNU398	SNU398_LIVER		1240217	Male
ACH-000093	3.95641	Panc 05.04	PANC0504	PANC0504_PANCREAS		NA	Female
ACH-000477	3.905939	Malme-3M	MALME3M	MALME3M_SKIN		NA	Male
ACH-000746	3.39661	GSS	GSS	GSS_STOMACH		NA	Male
ACH-000356	3.705087	MKN-45	MKN45	MKN45_STOMACH		925340	Female
ACH-000457	3.683134	CAL-54	CAL54	CAL54_KIDNEY		910952	Male
ACH-000904	3.20558	NCI-H2106	NCIH2106	NCIH2106_LUNG		NA	Male
ACH-000244	3.614762	DK-MG	DKMG	DKMG_CENTRAL_NERVOUS_SYSTEM		906839	Female
ACH-000610	3.130024	NCI-H2227	NCIH2227	NCIH2227_LUNG		688018	Male
ACH-000081	3.770053	GDM-1	GDM1	GDM1_HAEMATOPOIETIC_AND_LYMPHOID_TISSUE		906870	Female
ACH-000255	3.781939	LMSU	LMSU	LMSU_STOMACH		NA	Female
ACH-001038	3.130617		COGE352	COGE352_BONE	COG-E-352	NA	Male
ACH-000352	3.657147	HCC1428	HCC1428	HCC1428_BREAST		1290905	Female
ACH-002016	3.697094	Mel290	MEL290	MEL290_UVEA		NA	Female
ACH-000416	3.511831	NCI-H838	NCIH838	NCIH838_LUNG		910399	Male
ACH-000752	3.597459	NCI-H196	NCIH196	NCIH196_LUNG		1240186	Male

ACH-000862	3.664023	KMBC-2	KMBC2	KMBC2_URINARY_TRACT	NA	Unknown
ACH-000236	3.773744	SW1417	SW1417	SW1417_LARGE_INTESTINE	909747	Female
ACH-000401	4.024643	COLO-800	COLO800	COLO800_SKIN	906813	Male
ACH-000731	3.776286	HCC-2279	HCC2279	HCC2279_LUNG	NA	Female
ACH-000875	3.857733	NCI-H2347	NCIH2347	NCIH2347_LUNG	687820	Female
ACH-000626	3.632997	U266B1	U266B1	U266B1_HAEMATOPOIETIC_AND_LYMPHOID_TISSUE	753615	Male
ACH-000593	4.022194	BC-3C	BC3C	BC3C_URINARY_TRACT	NA	Female
ACH-000182	3.88115	SNU-869	SNU869	SNU869_BILIARY_TRACT	NA	Unknown
ACH-000442	3.828631	RERF-LC-Sq1	RERFLCSQ1	RERFLCSQ1_LUNG	1298538	Female
ACH-000159	3.840597	OS-RC-2	OSRC2	OSRC2_KIDNEY	909250	Male
ACH-000907	3.772483	SNU-349	SNU349	SNU349_KIDNEY	NA	Male
ACH-000191	3.771592	BHT-101	BHT101	BHT101_THYROID	906696	Female
ACH-000205	4.028062	PK-59	PK59	PK59_PANCREAS	NA	Female
ACH-000103	3.529534	Caov-4	CAOV4	CAOV4_OVARY	949090	Female
ACH-000739	3.541845	Hep G2	HEPG2	HEPG2_LIVER	NA	Male
ACH-001558	3.755246	Mero-48a	MERO48A	MERO48A_PLEURA	NA	Male
ACH-000206	3.785605		C8166	C8166_HAEMATOPOIETIC_AND_LYMPHOID_TISSUE	NA	Unknown
ACH-000841	3.695838	NCI-H2087	NCIH2087	NCIH2087_LUNG	724834	Male
ACH-000615	3.891264	SK-MEL-28	SKMEL28	SKMEL28_SKIN	905954	Male
ACH-001613	3.420629	OCI-AML4	OCIAML4	OCIAML4_HAEMATOPOIETIC_AND_LYMPHOID_TISSUE	NA	Female
ACH-001608	3.747281	NP 2	NP2	NP2_CENTRAL_NERVOUS_SYSTEM	NA	Male
ACH-000801	3.777446	Hs 936.T	HS936T	HS936T_SKIN	NA	Male

ACH-000539	3.771563	Hs 618.T	HS618T	HS618T_FIBROBLAST		NA	Female
ACH-000464	3.797172	CAS-1	CAS1	CAS1_CENTRAL_NERVOUS_SYSTEM		910943	Male
ACH-000622	3.649326	KNS-42	KNS42	KNS42_CENTRAL_NERVOUS_SYSTEM		907282	Male
ACH-000452	3.837543	TE-8	TE8	TE8_OESOPHAGUS		753623	Male
ACH-001390	3.788448	SUM-149PT	SUM149PT	SUM149PT_BREAST		NA	Female
ACH-001401	3.444292	SW-13	SW13	SW13_ADRENAL_CORTEX		909744	Female
ACH-000365	3.757681	SU-DHL-4	SUDHL4	SUDHL4_HAEMATOPOIETIC_AND_LYMPHOID_TISSUE		1331035	Male
ACH-001431	3.162503	TC205	TC205	TC205_BONE		NA	Unknown
ACH-000475	3.706706	huH-1	HUH1	HUH1_LIVER		1298146	Male
ACH-000860	3.572774	NCI-H358	NCIH358	NCIH358_LUNG		908465	Male
ACH-000074	3.771519	KU812	KU812	KU812_HAEMATOPOIETIC_AND_LYMPHOID_TISSUE		907311	Male
ACH-000377	4.089332	SNU-478	SNU478	SNU478_BILIARY_TRACT		NA	Unknown
ACH-001562	3.622396	Mero-95	MERO95	MERO95_PLEURA		NA	Male
ACH-002475	3.21221	HAP1	HAP1	HAP1_ENGINEERED		NA	Male
ACH-000259	3.06315	KELLY	KELLY	KELLY_AUTONOMIC_GANGLIA		753618	Female
ACH-001388	3.592767	SUM-102PT	SUM102PT	SUM102PT_BREAST		NA	Female
ACH-001410	3.728756	UM-UC-4	UMUC4	UMUC4_URINARY_TRACT		NA	Female
ACH-002025	3.922354	TT1TKB	TT1TKB	TT1TKB_LARGE_INTESTINE		NA	Male
ACH-000720	3.905083	TCCSUP	TCCSUP	TCCSUP_URINARY_TRACT		687459	Female
ACH-000095	3.270518	D341	D341	D341MED_CENTRAL_NERVOUS_SYSTEM	D341MED	NA	Male
ACH-001345	3.675653	GP5d	GP5D	GP5D_LARGE_INTESTINE		907291	Female
ACH-000558	3.793931	A-172	A172	A172_CENTRAL_NERVOUS_SYSTEM		687563	Male

ACH-000664	3.425884	SU-DHL-1	SUDHL1	SUDHL1_HAEMATOPOIETIC_AND_LYMPHOID_TISSUE		909742	Male
ACH-000177	3.562937	NCI-H660	NCIH660	NCIH660_PROSTATE		1330975	Male
ACH-000335	3.858154	MSTO-211H	MSTO211H	MSTO211H_PLEURA		908152	Male
ACH-000940	3.659556	AN3 CA	AN3CA	AN3CA_ENDOMETRIUM		910781	Female
ACH-000631	3.879428	KS-1	KS1	KS1_CENTRAL_NERVOUS_SYSTEM		907313	Female
ACH-000207	4.094257	Detroit 562	DETROIT562	DETROIT562_UPPER_AERODIGESTIVE_TRACT		906837	Female
ACH-000764	3.820978	SH-10-TC	SH10TC	SH10TC_STOMACH		NA	Unknown
ACH-000995	3.363624	JURKAT	JURKAT	JURKAT_HAEMATOPOIETIC_AND_LYMPHOID_TISSUE		998184	Male
ACH-000373	4.007794	SKM-1	SKM1	SKM1_HAEMATOPOIETIC_AND_LYMPHOID_TISSUE		909722	Male
ACH-000953	3.4188	SUP-T1	SUPT1	SUPT1_HAEMATOPOIETIC_AND_LYMPHOID_TISSUE	Sup-T1	909743	Male
ACH-000709	3.756872	KMRC-2	KMRC2	KMRC2_KIDNEY		NA	Male
ACH-000846	3.952788	FaDu	FADU	FADU_UPPER_AERODIGESTIVE_TRACT		906863	Male
ACH-001702	3.480423	VA-ES-BJ	VAESBJ	VAESBJ_SOFT_TISSUE		688121	Male
ACH-000333	3.758865	JHOM-2B	JHOM2B	JHOM2B_OVARY		NA	Female
ACH-002059	3.415076	P30/OHK	P30OHK	P30OHK_HAEMATOPOIETIC_AND_LYMPHOID_TISSUE		NA	Female
ACH-000929	3.946539	NCI-H2110	NCIH2110	NCIH2110_LUNG		1298351	Unknown
ACH-001395	3.354923	SUM-44PE	SUM44PE	SUM44PE_BREAST		NA	Female
ACH-000948	3.777736	23132/87	2313287	2313287_STOMACH		910924	Male
ACH-002015	3.715521	Mel285	MEL285	MEL285_UVEA		NA	Female
ACH-000981	3.744478	DND-41	DND41	DND41_HAEMATOPOIETIC_AND_LYMPHOID_TISSUE		1297446	Male
ACH-000617	3.707226	OVCAR-4	OVCAR4	OVCAR4_OVARY		905990	Female
ACH-000311	3.865649	NCI-H2122	NCIH2122	NCIH2122_LUNG		722046	Female

ACH-001210	3.583297	TTC-1240	TTC1240	TTC1240_SOFT_TISSUE		NA	Female
ACH-001834	3.783502	ICC10	ICC10	ICC10_BILIARY_TRACT		NA	Unknown
ACH-000390	3.803967	LUDLU-1	LUDLU1	LUDLU1_LUNG		NA	Male
ACH-000468	3.744741	PK-45H	PK45H	PK45H_PANCREAS		NA	Unknown
ACH-001061	3.761082	DLD-1	DLD1	DLD1_LARGE_INTESTINE		NA	Male
ACH-000077	3.770433	MJ	MJ	MJ_HAEMATOPOIETIC_AND_LYMPHOID_TISSUE		NA	Male
ACH-000456	3.820969	B-CPAP	BCPAP	BCPAP_THYROID		924104	Female
ACH-000496	3.774521	NCI-H1792	NCIH1792	NCIH1792_LUNG		724868	Male
ACH-002512	3.945112	MM1610113	MM1610113	MM1610113_SKIN	M1610113	NA	Male
ACH-000062	3.969249	RERF-LC-MS	RERFLCMS	RERFLCMS_LUNG		910931	Male
ACH-002464	3.794951	RPE1-ss6	RPE1SS6	RPE1SS6_ENGINEERED		NA	Female
ACH-000088	3.727657		HS172T	HS172T_FIBROBLAST		NA	Male
ACH-000536	3.831604	BT-20	BT20	BT20_BREAST		906801	Female
ACH-000071	3.68345		HS706T	HS706T_BONE		NA	Female
ACH-002004	3.547318	UACC62 SKIN CJ1	UACC62SKINCJ1	UACC62_SKIN_CJ1_RESISTANT	UACC62 RMR	NA	Unknown
ACH-000878	3.804693	HCC-15	HCC15	HCC15_LUNG		1240143	Male
ACH-001380	3.681601	PACADD-165	PACADD165	PACADD165_PANCREAS		NA	Male
NA.12	NA	NA	NA	NA	NA	NA	NA
ACH-000385	3.79405	SK-RC-20	SKRC20	SKRC20_KIDNEY		NA	Unknown
ACH-000412	4.126402	SNU-1197	SNU1197	SNU1197_LARGE_INTESTINE		NA	Male
ACH-000427	3.83552	NCI-N87	NCIN87	NCIN87_STOMACH		908461	Male
ACH-000938	3.624414	NALM-6	NALM6	NALM6_HAEMATOPOIETIC_AND_LYMPHOID_TISSUE		908158	Male
ACH-000462	3.7874	NALM-1	NALM1	NALM1_HAEMATOPOIETIC_AND_LYMPHOID_TISSUE		NA	Female

ACH-000939	3.744439	SK-UT-1	SKUT1	SKUT1_SOFT_TISSUE		909732	Female
ACH-000325	3.991631	SNU-620	SNU620	SNU620_STOMACH		NA	Female
ACH-000515	3.095717	HCC-33	HCC33	HCC33_LUNG		1303901	Male
ACH-001033	3.510638	CHLA-57	CHLA57	CHLA57_BONE		NA	Unknown
ACH-000919	3.95525	IM95	IM95	IM95_STOMACH		1240155	Male
ACH-000863	3.951993	DBTRG-05MG	DBTRG05MG	DBTRG05MG_CENTRAL_NERVOUS_SYSTEM		906835	Female
ACH-000642	4.082399	HMEL	HMEL	HMEL_BREAST		NA	Unknown
ACH-000581	3.822224	SNU-16	SNU16	SNU16_STOMACH		908446	Female
ACH-001050	3.659783	CW9019	CW9019	CW9019_SOFT_TISSUE		NA	Unknown
ACH-001818	3.628757	C396	C396	C396_BONE		NA	Unknown
ACH-001384	3.842458	RO82-W-1	RO82W1	RO82W1_THYROID		930083	Female
ACH-000797	3.880854	HLF-a	HLFA	HLFA_FIBROBLAST		NA	Female
ACH-000220	3.753998	Mino	MINO	MINO_HAEMATOPOIETIC_AND_LYMPHOID_TISSUE		NA	Male
NA.13	NA	NA	NA	NA	NA	NA	NA
ACH-000827	4.1301	WM-793	WM793	WM793_SKIN		1299081	Male
ACH-000961	3.512532	Ishikawa (Heraklio) 02 ER-	ISHIKAWAHERAKLIO02ER	ISHIKAWAHERAKLIO02ER_ENDOMETRIUM		NA	Female
ACH-000778	4.03404	HSC-3	HSC3	HSC3_UPPER_AERODIGESTIVE_TRACT		907061	Male
ACH-000825	3.690945	HOP-92	HOP92	HOP92_LUNG		905973	Male
ACH-000743	3.523824	COR-L95	CORL95	CORL95_LUNG		1297439	Male
ACH-000190	3.687939	HD-MY-Z	HDMYZ	HDMYZ_HAEMATOPOIETIC_AND_LYMPHOID_TISSUE		907050	Male
ACH-001819	3.585909	MFM-223	MFM223	MFM223_BREAST		NA	Female
ACH-000157	3.50957	A4/Fuk	A4FUK	A4FUK_HAEMATOPOIETIC_AND_LYMPHOID_TISSUE		910934	Female
ACH-001786	3.754217	SNU-1544	SNU1544	SNU1544_LARGE_INTESTINE		NA	Female

ACH-000253	3.741487	COLO 201	COLO201	COLO201_LARGE_INTESTINE	NA	Male
ACH-001376	3.572567	PACADD-135	PACADD135	PACADD135_PANCREAS	NA	Female
ACH-000958	3.802233	SW48	SW48	SW48_LARGE_INTESTINE	909751	Female
ACH-000228	3.85045	BICR 31	BICR31	BICR31_UPPER_AERODIGESTIVE_TRACT	1290725	Male
ACH-000266	3.878852	SNU-213	SNU213	SNU213_PANCREAS	NA	Male
ACH-001750	3.612555	TTC442	TTC442	TTC442_SOFT_TISSUE	NA	Male
ACH-000174	3.500291	CAL-62	CAL62	CAL62_THYROID	906828	Female
ACH-000245	3.689158	BL-41	BL41	BL41_HAEMATOPOIETIC_AND_LYMPHOID_TISSUE	910706	Male
ACH-001279	3.554902	SCCOHT-1	SCCOHT1	SCCOHT1_OVARY	NA	Unknown
ACH-000620	3.657847	JHH-1	JHH1	JHH1_LIVER	1298151	Male
ACH-000813	3.976593	T3M-10	T3M10	T3M10_LUNG	NA	Male
ACH-001513	3.747875	HCA-1	HCA1	HCA1_CERVIX	NA	Female
ACH-000168	4.099179	NOMO-1	NOMO1	NOMO1_HAEMATOPOIETIC_AND_LYMPHOID_TISSUE	908451	Female
ACH-000257	3.356498	COR-L279	CORL279	CORL279_LUNG	910937	Male
ACH-000211	3.566661	Daoy	DAOY	DAOY_CENTRAL_NERVOUS_SYSTEM	906833	Male
ACH-000283	3.912468	A1207	A1207	A1207_CENTRAL_NERVOUS_SYSTEM	NA	Female
ACH-002041	3.836369	HOTHC	HOTHC	HOTHC_THYROID	NA	Female
ACH-000987	3.891821	MeWo	MEWO	MEWO_SKIN	908128	Male
ACH-000682	3.994259	SNU-1066	SNU1066	SNU1066_UPPER_AERODIGESTIVE_TRACT	NA	Male
ACH-000073	3.764253	GRANTA-519	GRANTA519	GRANTA519_HAEMATOPOIETIC_AND_LYMPHOID_TISSUE	1303897	Female
ACH-000212	3.70803	CAL-120	CAL120	CAL120_BREAST	906826	Female
ACH-000096	3.400095	G-401	G401	G401_SOFT_TISSUE	907299	Male

ACH-001433	3.666725	CCLF_PEDS_0008_T	CCLFPEDS0008T	CCLFPEDS0008T_SOFT_TISSUE		NA	Unknown
ACH-000063	3.829617		HS616T	HS616T_FIBROBLAST		NA	Male
ACH-001999	3.928601	950-5-BIK	9505BIK	9505BIK_PANCREAS		NA	Unknown
ACH-000776	3.740022	ONS-76	ONS76	ONS76_CENTRAL_NERVOUS_SYSTEM		909248	Female
ACH-000521	3.785975	NCI-H2030	NCIH2030	NCIH2030_LUNG		722045	Male
ACH-000025	3.805562	CH-157MN	CH157MN	CH157MN_CENTRAL_NERVOUS_SYSTEM		NA	Female
ACH-000591	3.905503		LN235	LN235_CENTRAL_NERVOUS_SYSTEM	LN-235	NA	Male
ACH-001716	3.0911	GOTO	GOTO	GOTO_AUTONOMIC_GANGLIA		906875	Male
ACH-000833	3.648195	RH-30	RH30	RH30_SOFT_TISSUE		NA	Male
ACH-001628	3.65572	PEA1	PEA1	PEA1_OVARY		NA	Female
ACH-000398	3.84094	RI-1	RI1	RI1_HAEMATOPOIETIC_AND_LYMPHOID_TISSUE		NA	Female
ACH-000274	3.923463	Hs 852.T	HS852T	HS852T_SKIN		NA	Male
ACH-001370	3.835192	OCI-P5x	OCIP5X	OCIP5X_OVARY		NA	Female
ACH-000029	3.801877	HCC-827-GR5	HCC827GR5	HCC827GR5_LUNG	HCC827 GR5	NA	Female
ACH-001849	3.670593	ICC9	ICC9	ICC9_BILIARY_TRACT		NA	Unknown
ACH-000644	3.976241	COLO 829	COLO829	COLO829_SKIN		687448	Male
ACH-001422	3.757875	WPE1-NA22	WPE1NA22	WPE1NA22_PROSTATE		NA	Male
ACH-000588	3.6223	KMS-26	KMS26	KMS26_HAEMATOPOIETIC_AND_LYMPHOID_TISSUE		NA	Male
ACH-000849	3.924706	MDA-MB-468	MDAMB468	MDAMB468_BREAST		908123	Female
ACH-000219	3.845819	A-375	A375	A375_SKIN		906793	Female
ACH-000150	3.85825	HCC2935	HCC2935	HCC2935_LUNG		NA	Male
ACH-001845	3.575055	ICC5	ICC5	ICC5_BILIARY_TRACT		NA	Unknown

ACH-000384	3.791238	SW 780	SW780	SW780_URINARY_TRACT		687457	Female
ACH-001053	2.903131	D425	D425	D425_CENTRAL_NERVOUS_SYSTEM		NA	Unknown
ACH-001274	3.944727	SW982	SW982	SW982_SOFT_TISSUE		909759	Female
ACH-000091	3.871947	OV56	OV56	OV56_OVARY		1480362	Female
NA.14	NA	NA	NA	NA	NA	NA	NA
ACH-001634	3.501732	PGA-1	PGA1	PGA1_HAEMATOPOIETIC_AND_LYMPHOID_TISSUE		NA	Male
ACH-000495	3.961646	TUHR4TKB	TUHR4TKB	TUHR4TKB_KIDNEY		NA	Male
ACH-000911	4.089167	NUGC-3	NUGC3	NUGC3_STOMACH		908455	Male
ACH-000013	3.571086	ONCO-DG-1	ONCODG1	ONCODG1_OVARY		NA	Female
ACH-000777	3.702893	KYSE-30	KYSE30	KYSE30_OESOPHAGUS		NA	Male
ACH-001765	3.260093	Rh4	RH4	RH4_SOFT_TISSUE		NA	Female
ACH-000312	3.070988	SK-N-BE(2)	SKNBE2	SKNBE2_AUTONOMIC_GANGLIA		NA	Male
ACH-000085	3.860393	T3M-4	T3M4	T3M4_PANCREAS		NA	Male
ACH-000436	3.288113	OCI-My7	OCIMY7	OCIMY7_HAEMATOPOIETIC_AND_LYMPHOID_TISSUE	OCI-MY7	NA	Unknown
ACH-000869	3.746177	NCI-H1568	NCIH1568	NCIH1568_LUNG		1298348	Female
ACH-000267	3.710545	HDLM-2	HDLM2	HDLM2_HAEMATOPOIETIC_AND_LYMPHOID_TISSUE		924110	Male
ACH-000489	3.92844	SW1116	SW1116	SW1116_LARGE_INTESTINE		909746	Male
ACH-000431	3.062868	NCI-H1694	NCIH1694	NCIH1694_LUNG		688006	Male
ACH-000152	3.713724	M059K	M059K	M059K_CENTRAL_NERVOUS_SYSTEM		NA	Male
ACH-000909	3.722167	JHUEM-2	JHUEM2	JHUEM2_ENDOMETRIUM		NA	Female
ACH-000534	3.803353	WSU-DLCL2	WSUDLCL2	WSUDLCL2_HAEMATOPOIETIC_AND_LYMPHOID_TISSUE		1331050	Male
ACH-000582	3.744271	COLO 741	COLO741	COLO741_SKIN		NA	Female
ACH-000460	3.842225	SNU-8	SNU8	SNU8_OVARY		NA	Female

ACH-000443	3.31096	OVKATE	OVKATE	OVKATE_OVARY	1240199	Female
ACH-001570	3.787193	MM426	MM426	MM426_SKIN	NA	Male
ACH-000986	3.758812	HT115	HT115	HT115_LARGE_INTESTINE	907289	Unknown
ACH-001335	3.697937	C-4 II	C4II	C4II_CERVIX	NA	Female
ACH-000023	3.652237	PA-TU-8988T	PATU8988T	PATU8988T_PANCREAS	1240201	Female
ACH-002062	3.824471	SLVL	SLVL	SLVL_HAEMATOPOIETIC_AND_LYMPHOID_TISSUE	NA	Female
ACH-002462	3.720775	RPE1-ss48	RPE1SS48	RPE1SS48_ENGINEERED	NA	Female
ACH-000151	3.566211	JM1	JM1	JM1_HAEMATOPOIETIC_AND_LYMPHOID_TISSUE	1327768	Male
ACH-001277	3.297418	Yamato	YAMATO	YAMATO_SOFT_TISSUE	NA	Unknown
ACH-000607	3.412773	KYM-1	KYM1	KYM1_SOFT_TISSUE	1240166	Male
ACH-000703	3.290107	DMS 79	DMS79	DMS79_LUNG	753551	Male
ACH-001211	3.628291	TTC-549	TTC549	TTC549_SOFT_TISSUE	NA	Female
ACH-001511	3.714435	H413	H413	H413_UPPER_AERODIGESTIVE_TRACT	NA	Female
ACH-000113	3.907787	OCI-AML2	OCIAML2	OCIAML2_HAEMATOPOIETIC_AND_LYMPHOID_TISSUE	910947	Male
ACH-000337	3.928657	NCI-H3122	NCIH3122	NCIH3122_LUNG	1240190	Unknown
ACH-000598	3.564375	KMS-21BM	KMS21BM	KMS21BM_HAEMATOPOIETIC_AND_LYMPHOID_TISSUE	NA	Male
ACH-000057	3.620027	OPM-1	OPM1	OPM1_HAEMATOPOIETIC_AND_LYMPHOID_TISSUE	NA	Female
ACH-000281	3.657488	KP-2	KP2	KP2_PANCREAS	1298218	Female
ACH-000321	3.837511	MOLM-6	MOLM6	MOLM6_HAEMATOPOIETIC_AND_LYMPHOID_TISSUE	NA	Male
ACH-000985	3.751233	LS411N	LS411N	LS411N_LARGE_INTESTINE	907794	Male
ACH-000444	3.780637	LU99	LU99	LU99_LUNG	NA	Male
ACH-000347	3.525491	QGP-1	QGP1	QGP1_PANCREAS	1298534	Male

ACH-001399	3.780387	SW 626	SW626	SW626_LARGE_INTESTINE		909753	Female
ACH-001097	3.178386		KARPAS384	KARPAS384_HAEMATOPOIETIC_AND_LYMPHOID_TISSUE		NA	Male
ACH-000127	3.617997	SLR 20	SLR20	SLR20_KIDNEY		NA	Unknown
ACH-001961	3.794684	GB2	GB2	GB2_BILIARY_TRACT	EHGB2	NA	Unknown
ACH-001796	3.590539	95T1000	95T1000	95T1000_SOFT_TISSUE		NA	Unknown
ACH-000307	3.784561	PK-1	PK1	PK1_PANCREAS		NA	Male
ACH-001030	3.528348		CHLA218	CHLA218_BONE		NA	Female
ACH-000595	3.887319	LN-229	LN229	LN229_CENTRAL_NERVOUS_SYSTEM		1240169	Female
ACH-001540	3.61863	KMLS-1	KMLS1	KMLS1_SOFT_TISSUE		NA	Unknown
ACH-000298	3.383649	NCI-H2029	NCIH2029	NCIH2029_LUNG		688011	Female
ACH-000771	3.841848	BICR 56	BICR56	BICR56_UPPER_AERODIGESTIVE_TRACT		NA	Female
ACH-000500	3.900881	SNU-46	SNU46	SNU46_UPPER_AERODIGESTIVE_TRACT		NA	Male
ACH-000353	3.938889	TE-15	TE15	TE15_OESOPHAGUS		753614	Female
ACH-000363	3.500138	SK-MM-2	SKMM2	SKMM2_HAEMATOPOIETIC_AND_LYMPHOID_TISSUE		753612	Male
ACH-000350	3.676739	COLO-678	COLO678	COLO678_LARGE_INTESTINE		910689	Male
ACH-000053	3.607572	KARPAS-299	KARPAS299	KARPAS299_HAEMATOPOIETIC_AND_LYMPHOID_TISSUE		907273	Male
ACH-001543	3.956249	KOSC-2 cl3-43	KOSC2CL343	KOSC2CL343_UPPER_AERODIGESTIVE_TRACT	KOSC-2	NA	Male
ACH-000246	3.862752	SLR 23	SLR23	SLR23_KIDNEY		NA	Unknown
ACH-000663	3.720404	OVTOKO	OVTOKO	OVTOKO_OVARY		1298365	Female
ACH-000346	3.857779	JVM-3	JVM3	JVM3_HAEMATOPOIETIC_AND_LYMPHOID_TISSUE		907270	Male
ACH-000970	3.991047	SNU-C5	SNUC5	SNUC5_LARGE_INTESTINE		1674021	Female
ACH-001333	3.314514	C-33 A	C33A	C33A_CERVIX		687505	Female

ACH-000794	3.949058	BICR 22	BICR22	BICR22_UPPER_AERODIGESTIVE_TRACT		1240121	Male
ACH-000039	3.368568	SK-N-MC	SKNMC	SKNMC_BONE		NA	Female
ACH-000471	3.835278	Li-7	LI7	LI7_LIVER		NA	Male
ACH-000850	3.926715	Hs 698.T	HS698T	HS698T_FIBROBLAST		NA	Male
ACH-000291	3.772191	OV-90	OV90	OV90_OVARY		1240197	Female
ACH-001453	3.895519	BPH-1	BPH1	BPH1_PROSTATE		924105	Male
ACH-001458	3.556929	C75	C75	C75_LARGE_INTESTINE		NA	Male
ACH-000886	3.782604	NCI-H2009	NCIH2009	NCIH2009_LUNG		724873	Female
ACH-000888	3.867418	NCI-H1793	NCIH1793	NCIH1793_LUNG		908463	Female
ACH-000659	3.150246	SCLC-21H	SCLC21H	SCLC21H_LUNG		NA	Male
ACH-000030	3.876269	PC-14	PC14	PC14_LUNG		753608	Male
ACH-001201	2.936103	SU-MB-002	SUMB002	SUMB002_CENTRAL_NERVOUS_SYSTEM	SUMB002	NA	Unknown
ACH-000271	3.698724	SU-DHL-10	SUDHL10	SUDHL10_HAEMATOPOIETIC_AND_LYMPHOID_TISSUE		1331033	Male
ACH-000549	3.919862	SNU-1076	SNU1076	SNU1076_UPPER_AERODIGESTIVE_TRACT		NA	Male
ACH-000555	3.751542	A-498	A498	A498_KIDNEY		905948	Male
ACH-000478	4.009349	SNU-387	SNU387	SNU387_LIVER		909736	Female
ACH-000775	3.818423	NCI-H727	NCIH727	NCIH727_LUNG		724855	Female
ACH-000038	3.887452	EHEB	EHEB	EHEB_HAEMATOPOIETIC_AND_LYMPHOID_TISSUE		906854	Female
ACH-001109	3.595961	KP-MRT-RY	KPMRTRY	KPMRTRY_SOFT_TISSUE		NA	Male
ACH-001442	3.969604	A388	A388	A388_SKIN		910697	Male
ACH-000559	3.457322	NCI-H1836	NCIH1836	NCIH1836_LUNG		1240182	Male
ACH-000510	3.611848	NCI-H1299	NCIH1299	NCIH1299_LUNG		724831	Male

ACH-002065	3.986114	HS-PSS	HSPSS	HSPSS_CENTRAL_NERVOUS_SYSTEM		NA	Male
ACH-000676	3.898076	LN-464	LN464	LN464_CENTRAL_NERVOUS_SYSTEM		NA	Male
ACH-000844	3.378875	DMS 454	DMS454	DMS454_LUNG		NA	Male
ACH-000925	3.769427	DV-90	DV90	DV90_LUNG		NA	Male
NA.15	NA	NA	NA	NA	NA	NA	NA
ACH-000272	3.81404	SLR 24	SLR24	SLR24_KIDNEY		NA	Unknown
ACH-001035	3.284664	CHLA-99	CHLA99	CHLA99_BONE		NA	Male
ACH-000513	3.845504	SNU-1272	SNU1272	SNU1272_KIDNEY		NA	Female
ACH-001059	3.348421		DL	DL_SOFT_TISSUE		NA	Unknown
ACH-000959	3.874365	SNU-C4	SNUC4	SNUC4_LARGE_INTESTINE		NA	Male
ACH-000169	3.443193	RD	RD	RD_SOFT_TISSUE		909264	Female
ACH-001402	3.8741	SW756	SW756	SW756_CERVIX		724839	Female
ACH-000796	4.060662	MCAS	MCAS	MCAS_OVARY		NA	Female
ACH-000143	3.29503		HCC2429	HCC2429_LUNG		NA	Female
ACH-000067	3.942587	Hs 683	HS683	HS683_CENTRAL_NERVOUS_SYSTEM		1240150	Male
ACH-000640	4.007016	SK-MEL-31	SKMEL31	SKMEL31_SKIN		909727	Female
ACH-001674	3.051668	TGW	TGW	TGW_AUTONOMIC_GANGLIA		910780	Male
ACH-001698	3.686967	UPCI-SCC-116	UPCISCC116	UPCISCC116_UPPER_AERODIGESTIVE_TRACT		NA	Male
ACH-002002	3.875065	A375 SKIN CJ2	A375SKINCJ2	A375_SKIN_CJ2_RESISTANT	A375 ER-2	NA	Unknown
ACH-002011	3.524958	MP46	MP46	MP46_UVEA		NA	Male
ACH-000967	3.72922	SNU-C2A	SNUC2A	SNUC2A_LARGE_INTESTINE		NA	Female
ACH-000469	3.823306	YH-13	YH13	YH13_CENTRAL_NERVOUS_SYSTEM		909905	Male
ACH-001353	3.889068	JOPACA-1	JOPACA1	JOPACA1_PANCREAS		NA	Male

ACH-001574	3.748951	MOLM-14	MOLM14	MOLM14_HAEMATOPOIETIC_AND_LYMPHOID_TISSUE		NA	Male
ACH-000097	3.556153	ZR-75-1	ZR751	ZR751_BREAST		NA	Female
ACH-000256	3.779045	COV318	COV318	COV318_OVARY		NA	Female
ACH-000977	3.417816	LNCaP clone FGC	LNCAPCLONEFGC	LNCAPCLONEFGC_PROSTATE	LNCAPCLONEFGC, LN-Cap-cloneFGC, LNCaP clone FGC	907788	Male
ACH-000972	3.63872	HEC-151	HEC151	HEC151_ENDOMETRIUM		NA	Female
ACH-001367	2.821598	NMB	NMB	NMB_AUTONOMIC_GANGLIA		NA	Unknown
ACH-000382	3.234439	COR-L24	CORL24	CORL24_LUNG		NA	Male
ACH-000579	3.745638	UACC-257	UACC257	UACC257_SKIN		905977	Unknown
ACH-001532	3.393405	JMU-RTK-2	JMURTK2	JMURTK2_SOFT_TISSUE		NA	Female
ACH-000695	3.196709	COR-L47	CORL47	CORL47_LUNG		NA	Male
ACH-001795	3.87121	94T778	94T778	94T778_SOFT_TISSUE		NA	Female
ACH-001510	3.923369	H376	H376	H376_UPPER_AERODIGESTIVE_TRACT		NA	Female
ACH-000502	3.875824	TCC-PAN2	TCCPAN2	TCCPAN2_PANCREAS		NA	Female
ACH-000183	3.57175	L-363	L363	L363_HAEMATOPOIETIC_AND_LYMPHOID_TISSUE		924239	Female
ACH-000408	3.678874	TE-5	TE5	TE5_OESOPHAGUS		735784	Female
ACH-001496	3.760348	ESO26	ESO26	ESO26_OESOPHAGUS		1503366	Male
ACH-000788	4.036504	A2058	A2058	A2058_SKIN		906792	Male
ACH-001622	3.895778	Onda 7	ONDA7	ONDA7_CENTRAL_NERVOUS_SYSTEM		NA	Male
ACH-000466	3.875612	SNU-216	SNU216	SNU216_STOMACH		NA	Female
ACH-001560	3.625632	Mero-83	MERO83	MERO83_PLEURA		NA	Male
ACH-000005	3.615885	HEL 92.1.7	HEL9217	HEL9217_HAEMATOPOIETIC_AND_LYMPHOID_TISSUE		NA	Male

ACH-001687	3.539489	UM-RC-3	UMRC3	UMRC3_KIDNEY		NA	Unknown
ACH-001151	3.676872	OVCAR-5	OVCAR5	OVCAR5_OVARY		905969	Female
ACH-000701	3.777389	RMUG-S	RMUGS	RMUGS_OVARY		NA	Female
ACH-000542	3.814792	Hey-A8	HEYA8	HEYA8_OVARY		NA	Female
ACH-000126	3.869969	KG-1-C	KG1C	KG1C_CENTRAL_NERVOUS_SYSTEM		NA	Male
ACH-001685	3.635105	U-HO1	UHO1	UHO1_HAEMATOPOIETIC_AND_LYMPHOID_TISSUE		NA	Male
ACH-000933	3.954071	SNU-324	SNU324	SNU324_PANCREAS		NA	Male
ACH-000068	3.878087	REC-1	REC1	REC1_HAEMATOPOIETIC_AND_LYMPHOID_TISSUE		NA	Male
ACH-000015	3.354254	NCI-H1581	NCIH1581	NCIH1581_LUNG	NCI-H2077	908471	Male
ACH-001111	3.468339	L82	L82	L82_HAEMATOPOIETIC_AND_LYMPHOID_TISSUE		NA	Female
ACH-000487	3.795466	F-36P	F36P	F36P_HAEMATOPOIETIC_AND_LYMPHOID_TISSUE		NA	Male
ACH-000122	3.446184	SUP-T11	SUPT11	SUPT11_HAEMATOPOIETIC_AND_LYMPHOID_TISSUE		NA	Male
ACH-000288	3.998844	BT-549	BT549	BT549_BREAST		905951	Female
ACH-001820	3.792054	COLO-824	COLO824	COLO824_BREAST		NA	Female
ACH-000782	3.608195	SEM	SEM	SEM_HAEMATOPOIETIC_AND_LYMPHOID_TISSUE		NA	Female
ACH-001632	3.411378	PEO4	PEO4	PEO4_OVARY		NA	Female
ACH-000357	3.432354	JeKo-1	JEKO1	JEKO1_HAEMATOPOIETIC_AND_LYMPHOID_TISSUE		1327765	Female
ACH-000079	3.865726	TE 125.T	TE125T	TE125T_FIBROBLAST		NA	Female
ACH-001031	3.263601	CHLA-266	CHLA266	CHLA266_SOFT_TISSUE		NA	Female
ACH-001048	3.935542	COV504	COV504	COV504_OVARY		NA	Female
ACH-000453	3.58329	MOLP-2	MOLP2	MOLP2_HAEMATOPOIETIC_AND_LYMPHOID_TISSUE		NA	Male
ACH-000791	3.794631	RERF-LC-Ad1	RERFLCAD1	RERFLCAD1_LUNG		NA	Male

ACH-000567	3.773749	ST486	ST486	ST486_HAEMATOPOIETIC_AND_LYMPHOID_TISSUE		910906	Female
ACH-001391	3.820156	SUM-159PT	SUM159PT	SUM159PT_BREAST		NA	Female
ACH-001075	3.758094	NCI-H292	NCIH292	NCIH292_LUNG	H292	753604	Female
ACH-000105	3.43624	ALL-SIL	ALLSIL	ALLSIL_HAEMATOPOIETIC_AND_LYMPHOID_TISSUE		1295740	Male
ACH-000557	4.040134	AML-193	AML193	AML193_HAEMATOPOIETIC_AND_LYMPHOID_TISSUE		NA	Female
ACH-000175	3.832424		TE159T	TE159T_FIBROBLAST		NA	Male
ACH-001356	3.664728	MB-1	MB1	MB1_THYROID		NA	Male
ACH-001844	3.659069	ICC4	ICC4	ICC4_BILIARY_TRACT		NA	Unknown
ACH-001346	3.855495	H103	H103	H103_UPPER_AERODIGESTIVE_TRACT		NA	Male
ACH-000885	3.671987	TOV-21G	TOV21G	TOV21G_OVARY		1240222	Female
ACH-000900	3.849246	NCI-H23	NCIH23	NCIH23_LUNG		905942	Male
ACH-000666	3.757822	NCI-H1355	NCIH1355	NCIH1355_LUNG		724866	Male
ACH-000217	3.951375	JHH-6	JHH6	JHH6_LIVER		1240159	Female
ACH-000872	3.803336	HCC515	HCC515	HCC515_LUNG		NA	Female
ACH-001638	3.667704	RC-K8	RCK8	RCK8_HAEMATOPOIETIC_AND_LYMPHOID_TISSUE		1330995	Male
ACH-001228	3.629309		UPCISCC152	UPCISCC152_UPPER_AERODIGESTIVE_TRACT	UPCISCC152, UPCI:SCC152	NA	Male
ACH-000181	3.82683	SCC-9	SCC9	SCC9_UPPER_AERODIGESTIVE_TRACT		909709	Male
ACH-000115	3.445933	VCaP	VCAP	VCAP_PROSTATE		1299075	Male
ACH-000130	3.61071	NALM-19	NALM19	NALM19_HAEMATOPOIETIC_AND_LYMPHOID_TISSUE		NA	Male
ACH-000121	4.005904	NCI-H2405	NCIH2405	NCIH2405_LUNG		687821	Male
ACH-001711	3.479371	PFSK-1	PFSK1	PFSK1_CENTRAL_NERVOUS_SYSTEM		683667	Male
ACH-000934	3.880571	MDA-MB-361	MDAMB361	MDAMB361_BREAST		908121	Female

ACH-000996	3.644292	HEC-251	HEC251	HEC251_ENDOMETRIUM		NA	Female
ACH-001529	3.541464	JAR	JAR	JAR_PLACENTA		907175	Male
ACH-001743	3.368242	RC2	RC2	RC2_SOFT_TISSUE		NA	Unknown
ACH-000320	3.925598	PSN1	PSN1	PSN1_PANCREAS	GR-M	910546	Male
ACH-000505	3.910352	RKN	RKN	RKN_SOFT_TISSUE		1298539	Female
ACH-000781	3.749928	NCI-H2023	NCIH2023	NCIH2023_LUNG		1240187	Male
ACH-000059	3.81008	SUP-B15	SUPB15	SUPB15_HAEMATOPOIETIC_AND_LYMPHOID_TISSUE		1247871	Male
ACH-000920	3.392498	CML-T1	CMLT1	CMLT1_HAEMATOPOIETIC_AND_LYMPHOID_TISSUE		910951	Female
ACH-000294	3.951325	NB-4	NB4	NB4_HAEMATOPOIETIC_AND_LYMPHOID_TISSUE		1323913	Female
ACH-000422	3.729063	SNU-475	SNU475	SNU475_LIVER		909739	Male
ACH-000200	3.827301	NMC-G1	NMCG1	NMCG1_CENTRAL_NERVOUS_SYSTEM		908449	Female
ACH-000046	3.825129	ACHN	ACHN	ACHN_KIDNEY		905950	Male
ACH-000968	3.999407	COLO 792	COLO792	COLO792_SKIN		906814	Male
ACH-000830	3.304473	NCI-H1436	NCIH1436	NCIH1436_LUNG		908469	Male
ACH-000114	3.851951	SU.86.86	SU8686	SU8686_PANCREAS		1240218	Female
ACH-000402	3.637287	BL-70	BL70	BL70_HAEMATOPOIETIC_AND_LYMPHOID_TISSUE		NA	Male
ACH-000036	3.918352	U343	U343	U343_CENTRAL_NERVOUS_SYSTEM		NA	Unknown
ACH-000973	3.688828	639-V	639V	639V_URINARY_TRACT		906798	Male
ACH-001128	3.576506	MON	MON	MON_SOFT_TISSUE		NA	Male
ACH-000750	3.763651	LOX IMVI	LOXIMVI	LOXIMVI_SKIN		905974	Male
ACH-000285	3.687728	Toledo	TOLEDO	TOLEDO_HAEMATOPOIETIC_AND_LYMPHOID_TISSUE		NA	Female
ACH-001858	3.913694	SSP-25	SSP25	SSP25_BILIARY_TRACT		NA	Female

ACH-001997	3.905788	ECC2	ECC2	ECC2_BILIARY_TRACT	NA	Unknown
ACH-000052	3.614723	A-673	A673	A673_BONE	684052	Female

Supplementary table 14. Summary of survival information of 18 immunotherapy cohorts in bulk level.

batch	ID	OS	status	PFS	status2	response	BOR
Bruan_RCC_pre_aPD1_combo_tpm	G138701_RCCBMS-00020-T_v1_RNA_OnPrem	25.34795	1	12.16438	1	0	0
Bruan_RCC_pre_aPD1_combo_tpm	G138701_RCCBMS-00097-T_v1_RNA_OnPrem	36.52603	1	5.490411	1	1	0
Bruan_RCC_pre_aPD1_combo_tpm	G138701_RCCBMS-00141-T_v1_RNA_OnPrem	68.54795	0	61.90685	0	1	0
Bruan_RCC_pre_aPD1_combo_tpm	G138701_RCCBMS-00099-T_v1_RNA_OnPrem	1.479452	1	1.249315	1	0	1
Bruan_RCC_pre_aPD1_combo_tpm	G138701_RCCBMS-00163-T_v1_RNA_OnPrem	63.97808	0	5.654795	1	0	0
Bruan_RCC_pre_aPD1_combo_tpm	G138701_RCCBMS-00053-T_v1_RNA_OnPrem	72.13151	0	15.41918	1	0	0
Bruan_RCC_pre_aPD1_combo_tpm	G138701_RCCBMS-00116-T_v1_RNA_OnPrem	5.950685	1	1.216438	1	0	1
Bruan_RCC_pre_aPD1_combo_tpm	G138701_RCCBMS-00136-T_v1_RNA_OnPrem	35.5726	1	2.860274	1	0	0
Bruan_RCC_pre_aPD1_combo_tpm	G138701_RCCBMS-00057-T_v1_RNA_OnPrem	39.09041	1	7.00274	1	0	0
Bruan_RCC_pre_aPD1_combo_tpm	G109543_RCCBMS-00007-T_v1_RNA_OnPrem	73.15068	0	33.33699	1	1	0
Bruan_RCC_pre_aPD1_combo_tpm	G138701_RCCBMS-00008-T_v1_RNA_OnPrem	1.183562	1	0.953425	1	0	1
Bruan_RCC_pre_aPD1_combo_tpm	G138701_RCCBMS-00065-T_v1_RNA_OnPrem	13.05205	1	1.380822	1	0	1
Bruan_RCC_pre_aPD1_combo_tpm	G138701_RCCBMS-00110-T_v1_RNA_OnPrem	9.435616	1	2.10411	1	0	0
Bruan_RCC_pre_aPD1_combo_tpm	G138701_RCCBMS-00111-T_v1_RNA_OnPrem	4.043836	1	1.413699	1	0	1
Bruan_RCC_pre_aPD1_combo_tpm	G109543_RCCBMS-00147-T_v1_RNA_OnPrem	1.972603	1	1.972603	1	0	1
Bruan_RCC_pre_aPD1_combo_tpm	G138701_RCCBMS-00044-T_v1_RNA_OnPrem	4.536986	1	1.643836	1	0	1
Bruan_RCC_pre_aPD1_combo_tpm	G138701_RCCBMS-00084-T_v1_RNA_OnPrem	56.71233	1	1.380822	1	0	1
Bruan_RCC_pre_aPD1_combo_tpm	G138701_RCCBMS-00093-T_v1_RNA_OnPrem	5.128767	1	1.315068	1	0	1

Bruan_RCC_pre_aPD1_combo_tpm	G138701_RCCBMS-00154-T_v1_RNA_OnPrem	46.25753	1	27.87945	1	1	0
Bruan_RCC_pre_aPD1_combo_tpm	G138701_RCCBMS-00194-T_v1_RNA_OnPrem	7.989041	1	1.315068	1	0	1
Bruan_RCC_pre_aPD1_combo_tpm	G138701_RCCBMS-00028-T_v1_RNA_OnPrem	18.57534	1	10.84932	1	1	0
Bruan_RCC_pre_aPD1_combo_tpm	G138701_RCCBMS-00047-T_v1_RNA_OnPrem	64.96438	1	4.175342	1	0	0
Bruan_RCC_pre_aPD1_combo_tpm	G138701_RCCBMS-00178-T_v1_RNA_OnPrem	8.219178	1	5.523288	1	1	0
Bruan_RCC_pre_aPD1_combo_tpm	G138701_RCCBMS-00068-T_v1_RNA_OnPrem	4.175342	1	2.20274	1	0	1
Bruan_RCC_pre_aPD1_combo_tpm	G138701_RCCBMS-00072-T_v1_RNA_OnPrem	60.72329	0	39.15616	0	1	0
Bruan_RCC_pre_aPD1_combo_tpm	G138701_RCCBMS-00035-T_v1_RNA_OnPrem	2.958904	1	1.183562	1	0	1
Bruan_RCC_pre_aPD1_combo_tpm	G109543_RCCBMS-00114-T_v1_RNA_OnPrem	15.87945	1	7.10137	1	0	0
Bruan_RCC_pre_aPD1_combo_tpm	G138701_RCCBMS-00125-T_v1_RNA_OnPrem	69.99452	0	2.630137	1	0	1
Bruan_RCC_pre_aPD1_combo_tpm	G138701_RCCBMS-00165-T_v1_RNA_OnPrem	50.36712	1	1.413699	1	0	1
Bruan_RCC_pre_aPD1_combo_tpm	G138701_RCCBMS-00092-T_v1_RNA_OnPrem	25.31507	1	1.413699	1	0	1
Bruan_RCC_pre_aPD1_combo_tpm	G138701_RCCBMS-00036-T_v1_RNA_OnPrem	13.38082	1	0.526027	1	0	1
Bruan_RCC_pre_aPD1_combo_tpm	G138701_RCCBMS-00011-T_v1_RNA_OnPrem	68.90959	0	8.547945	1	0	0
Bruan_RCC_pre_aPD1_combo_tpm	G109543_RCCBMS-00145-T_v1_RNA_OnPrem	25.24932	1	1.413699	1	0	1
Bruan_RCC_pre_aPD1_combo_tpm	G109543_RCCBMS-00196-T_v1_RNA_OnPrem	50.66301	1	1.347945	1	0	1
Bruan_RCC_pre_aPD1_combo_tpm	G138701_RCCBMS-00090-T_v1_RNA_OnPrem	22.71781	1	2.991781	1	0	0
Bruan_RCC_pre_aPD1_combo_tpm	G138701_RCCBMS-00113-T_v1_RNA_OnPrem	68.94247	0	11.0137	1	0	0
Bruan_RCC_pre_aPD1_combo_tpm	G109543_RCCBMS-00076-T2_v1_RNA_OnPrem	28.20822	1	6.049315	1	1	0
Bruan_RCC_pre_aPD1_combo_tpm	G138701_RCCBMS-00098-T_v1_RNA_OnPrem	39.15616	1	39.15616	1	1	0
Bruan_RCC_pre_aPD1_combo_tpm	G138701_RCCBMS-00103-T_v1_RNA_OnPrem	13.21644	0	8.054795	1	0	0
Bruan_RCC_pre_aPD1_combo_tpm	G138701_RCCBMS-00137-T_v1_RNA_OnPrem	22.84932	1	9.928767	1	1	0

Bruan_RCC_pre_aPD1_combo_tpm	G109543_RCCBMS-00150-T_v1_RNA_OnPrem	7.956164	1	1.446575	1	0	1
Bruan_RCC_pre_aPD1_combo_tpm	G138701_RCCBMS-00191-T_v1_RNA_OnPrem	6.936986	1	1.347945	1	0	1
Bruan_RCC_pre_aPD1_combo_tpm	G138701_RCCBMS-00169-T_v1_RNA_OnPrem	61.8411	0	25.11781	1	1	0
Bruan_RCC_pre_aPD1_combo_tpm	G138701_RCCBMS-00049-T_v1_RNA_OnPrem	5.227397	1	4.109589	1	0	0
Bruan_RCC_pre_aPD1_combo_tpm	G138701_RCCBMS-00025-T_v1_RNA_OnPrem	31.26575	1	0.032877	1	0	0
Bruan_RCC_pre_aPD1_combo_tpm	EA639146	5.650924	1	0.722793	1	0	0
Bruan_RCC_pre_aPD1_combo_tpm	P66425-02F-Run1_S7_L001	10.87474	1	0.985626	1	0	1
Bruan_RCC_pre_aPD1_combo_tpm	P66291-09A-Run1_S10_L001	29.86448	1	3.843943	1	0	0
Bruan_RCC_pre_aPD1_combo_tpm	P66425-08F-Run1_S7_L001	50.03696	1	15.6386	1	1	0
Bruan_RCC_pre_aPD1_combo_tpm	P66511-05E-Run1_S11_L001	2.135524	1	1.182752	1	0	1
Bruan_RCC_pre_aPD1_combo_tpm	EA632171	63.40862	0	3.055441	1	0	0
Bruan_RCC_pre_aPD1_combo_tpm	P66425-10A-Run1_S13_L002	11.72895	1	5.355236	1	0	0
Bruan_RCC_pre_aPD1_combo_tpm	P66287-07B-Run1_S2_L001	59.92608	0	57.56057	0	1	0
Bruan_RCC_pre_aPD1_combo_tpm	P66287-09H-Run1_S14_L001	17.577	1	1.839836	1	0	1
Bruan_RCC_pre_aPD1_combo_tpm	P66507-10D-Run1_S4_L001	38.57084	1	9.264887	1	0	0
Bruan_RCC_pre_aPD1_combo_tpm	P66291-12D-Run1_S29_L002	57.00205	0	3.581109	1	0	0
Bruan_RCC_pre_aPD1_combo_tpm	P66507-09C-Run1_S26_L002	6.603696	1	4.960986	1	0	0
Bruan_RCC_pre_aPD1_combo_tpm	P66451-09H-Run1_S2_L001	19.77823	1	5.322382	1	0	0
Bruan_RCC_pre_aPD1_combo_tpm	EA647814	57.00205	1	11.13758	1	0	0
Bruan_RCC_pre_aPD1_combo_tpm	EA595647	31.83573	1	29.01027	1	1	0
Bruan_RCC_pre_aPD1_combo_tpm	P66432-08G-Run1_S26_L002	47.2115	0	1.806982	1	0	1
Bruan_RCC_pre_aPD1_combo_tpm	P66451-06C-Run1_S12_L001	28.25462	1	7.293634	1	0	0
Bruan_RCC_pre_aPD1_combo_tpm	P66287-11C-Run1_S22_L002	16.72279	1	1.905544	1	0	0
Bruan_RCC_pre_aPD1_combo_tpm	P66282-01G-Run1_S3_L001	57.7577	0	54.99795	0	1	0
Bruan_RCC_pre_aPD1_combo_tpm	P66425-06E-Run1_S28_L002	9.067762	1	5.716632	1	0	0
Bruan_RCC_pre_aPD1_combo_tpm	P66511-10D-Run1_S3_L001	56.31211	0	1.544148	1	0	1
Bruan_RCC_pre_aPD1_combo_tpm	P66511-06G-Run1_S16_L001	20.36961	1	9.232033	1	0	0
Bruan_RCC_pre_aPD1_combo_tpm	EA632688	59.43326	0	6.505133	1	1	0
Bruan_RCC_pre_aPD1_combo_tpm	P66425-07C-Run1_S2_L001	22.43943	1	7.195072	1	0	0
Bruan_RCC_pre_aPD1_combo_tpm	P66425-06G-Run1_S30_L002	22.86653	1	10.94045	1	1	0
Bruan_RCC_pre_aPD1_combo_tpm	EA647820	36.20534	1	5.355236	1	0	0
Bruan_RCC_pre_aPD1_combo_tpm	EA595632	0.295688	1	0.065708	1	0	0
Bruan_RCC_pre_aPD1_combo_tpm	P66511-09B-Run1_S26_L002	55.88501	0	5.519507	1	0	0
Bruan_RCC_pre_aPD1_combo_tpm	P66282-10E-Run1_S12_L002	35.12115	1	11.40041	1	0	0
Bruan_RCC_pre_aPD1_combo_tpm	P66432-07E-Run1_S20_L002	5.092402	1	0.032854	1	0	0
Bruan_RCC_pre_aPD1_combo_tpm	EA639153	51.18686	0	4.731006	1	0	0
Bruan_RCC_pre_aPD1_combo_tpm	EA632174	62.62012	0	38.6694	1	1	0
Bruan_RCC_pre_aPD1_combo_tpm	P66287-06H-Run1_S30_L002	12.94456	1	5.552361	1	1	0
Bruan_RCC_pre_aPD1_combo_tpm	P66451-08H-Run1_S26_L002	0.952772	0	0.032854	0	0	0

Bruan_RCC_pre_aPD1_combo_tpm	EA632758	0.821355	1	0.459959	1	0	0
Bruan_RCC_pre_aPD1_combo_tpm	EA632149	63.2115	0	14.88296	1	0	0
Bruan_RCC_pre_aPD1_combo_tpm	P66507-04A-Run1_S1_L001	65.80698	0	7.392197	1	0	0
Bruan_RCC_pre_aPD1_combo_tpm	P66282-05D-Run1_S21_L002	21.91376	1	1.774127	1	0	1
Bruan_RCC_pre_aPD1_combo_tpm	P66451-05D-Run1_S8_L001	30.0616	1	1.839836	1	0	1
Bruan_RCC_pre_aPD1_combo_tpm	P66287-04D-Run1_S18_L002	40.01643	1	1.741273	1	0	1
Bruan_RCC_pre_aPD1_combo_tpm	P66291-05G-Run1_S22_L002	31.17864	1	1.806982	1	0	1
Bruan_RCC_pre_aPD1_combo_tpm	P66291-01B-Run1_S2_L001	63.27721	0	61.66735	0	1	0
Bruan_RCC_pre_aPD1_combo_tpm	P66287-03A-Run1_S9_L001	5.256674	1	1.806982	1	0	1
Bruan_RCC_pre_aPD1_combo_tpm	EA632123	12.74743	1	1.839836	1	0	1
Bruan_RCC_pre_aPD1_combo_tpm	P66507-02D-Run1_S25_L002	47.70431	1	37.71663	1	0	0
Bruan_RCC_pre_aPD1_combo_tpm	EA595720	36.96099	1	16.16427	1	0	0
Bruan_RCC_pre_aPD1_combo_tpm	P66507-08D-Run1_S20_L002	9.724846	1	1.905544	1	0	1
Bruan_RCC_pre_aPD1_combo_tpm	P66507-12B-Run1_S13_L001	50.85832	0	1.938398	1	0	1
Bruan_RCC_pre_aPD1_combo_tpm	P66507-01E-Run1_S19_L002	40.31211	0	37.74949	0	1	0
Bruan_RCC_pre_aPD1_combo_tpm	EA595719	53.22382	0	7.162218	1	0	0
Bruan_RCC_pre_aPD1_combo_tpm	P66432-04D-Run1_S2_L001	43.3347	1	1.938398	1	0	1
Bruan_RCC_pre_aPD1_combo_tpm	P66507-07F-Run1_S16_L002	1.774127	1	1.478439	1	0	1
Bruan_RCC_pre_aPD1_combo_tpm	EA632813	13.96304	1	5.848049	1	0	0
Bruan_RCC_pre_aPD1_combo_tpm	EA632133	13.17454	1	13.17454	1	1	0
Bruan_RCC_pre_aPD1_combo_tpm	P66432-11C-Run1_S7_L001	12.4846	0	1.741273	1	0	1
Bruan_RCC_pre_aPD1_combo_tpm	P66432-02C-Run1_S22_L002	12.25462	1	3.843943	1	0	0
Bruan_RCC_pre_aPD1_combo_tpm	EA639099	22.11088	1	7.162218	1	1	0
Bruan_RCC_pre_aPD1_combo_tpm	P66287-04G-Run1_S20_L002	27.6961	1	9.232033	1	0	0
Bruan_RCC_pre_aPD1_combo_tpm	P66432-06D-Run1_S11_L001	12.09035	1	0.985626	1	0	1
Bruan_RCC_pre_aPD1_combo_tpm	P66425-02C-Run1_S5_L001	34.52977	1	7.359343	1	0	0
Bruan_RCC_pre_aPD1_combo_tpm	P66451-01E-Run1_S17_L002	59.92608	0	1.774127	1	0	1
Bruan_RCC_pre_aPD1_combo_tpm	P66291-06C-Run1_S26_L002	10.21766	1	0.032854	1	0	0
Bruan_RCC_pre_aPD1_combo_tpm	P66432-10G-Run1_S4_L001	58.9076	0	29.2731	1	0	1
Bruan_RCC_pre_aPD1_combo_tpm	P66425-02E-Run1_S6_L001	10.64476	1	2.004107	1	0	1
Bruan_RCC_pre_aPD1_combo_tpm	P66507-03H-Run1_S30_L002	31.34292	1	1.708419	1	0	1
Bruan_RCC_pre_aPD1_combo_tpm	EA639120	14.39014	1	7.425051	1	0	0
Bruan_RCC_pre_aPD1_combo_tpm	P66511-10G-Run1_S4_L001	8.706366	1	1.511294	1	0	1
Bruan_RCC_pre_aPD1_combo_tpm	EA639131	53.12526	0	3.778234	1	0	0
Bruan_RCC_pre_aPD1_combo_tpm	P66451-04F-Run1_S6_L001	22.20945	1	1.741273	1	0	1
Bruan_RCC_pre_aPD1_combo_tpm	P66451-08G-Run1_S25_L002	14.423	1	7.392197	1	0	0
Bruan_RCC_pre_aPD1_combo_tpm	P66432-07D-Run1_S19_L002	38.80082	1	1.938398	1	0	1
Bruan_RCC_pre_aPD1_combo_tpm	EA595529	20.69815	1	0.032854	1	0	0
Bruan_RCC_pre_aPD1_combo_tpm	EA595597	57.3963	0	53.71663	0	1	0
Bruan_RCC_pre_aPD1_combo_tpm	P66291-10G-Run1_S20_L002	62.48871	0	15.47433	1	0	0
Bruan_RCC_pre_aPD1_combo_tpm	P66507-02B-Run1_S23_L002	1.577002	1	1.379877	1	0	1
Bruan_RCC_pre_aPD1_combo_tpm	EA647824	7.49076	1	3.712526	1	0	0
Bruan_RCC_pre_aPD1_combo_tpm	P66511-01E-Run1_S17_L002	23.35934	1	5.848049	1	0	0
Bruan_RCC_pre_aPD1_combo_tpm	EA632768	10.15195	1	1.215606	1	0	0

Bruan_RCC_pre_aPD1_combo_tpm	EA639107	22.01232	0	9.166324	1	0	0
Bruan_RCC_pre_aPD1_combo_tpm	EA639109	53.51951	0	23.98357	1	1	0
Bruan_RCC_pre_aPD1_combo_tpm	EA595500	3.548255	1	2.135524	1	0	1
Bruan_RCC_pre_aPD1_combo_tpm	P66425-10B-Run1_S14_L001	10.48049	1	0.032854	1	0	0
Bruan_RCC_pre_aPD1_combo_tpm	P66425-03D-Run1_S11_L001	56.54209	1	2.069815	1	0	1
Bruan_RCC_pre_aPD1_combo_tpm	EA632802	41.52772	1	9.954825	1	0	1
Bruan_RCC_pre_aPD1_combo_tpm	P66451-05G-Run1_S9_L001	13.33881	1	2.036961	1	0	1
Bruan_RCC_pre_aPD1_combo_tpm	P66291-07E-Run1_S3_L001	4.731006	1	1.839836	1	0	1
Bruan_RCC_pre_aPD1_combo_tpm	P66291-07H-Run1_S5_L001	55.68789	0	9.560575	1	1	0
Bruan_RCC_pre_aPD1_combo_tpm	P66291-11F-Run1_S24_L002	13.43737	1	1.87269	1	0	1
Bruan_RCC_pre_aPD1_combo_tpm	P66425-06F-Run1_S29_L002	60.02464	0	11.30185	1	0	0
Bruan_RCC_pre_aPD1_combo_tpm	P66432-02B-Run1_S21_L002	59.82752	0	20.3039	1	0	0
Bruan_RCC_pre_aPD1_combo_tpm	P66282-05G-Run1_S23_L002	18.13552	1	5.092402	1	0	0
Bruan_RCC_pre_aPD1_combo_tpm	P66282-01B-Run1_S1_L001	42.38193	1	42.38193	1	1	0
Bruan_RCC_pre_aPD1_combo_tpm	EA595522	19.64682	1	3.186858	1	0	0
Bruan_RCC_pre_aPD1_combo_tpm	P66291-08G-Run1_S8_L001	11.23614	1	3.712526	1	0	0
Bruan_RCC_pre_aPD1_combo_tpm	EA639018	17.05133	1	5.420945	1	0	0
Bruan_RCC_pre_aPD1_combo_tpm	P66287-05E-Run1_S24_L002	60.64887	0	21.8809	1	0	0
Bruan_RCC_pre_aPD1_combo_tpm	P66291-07B-Run1_S17_L002	15.11294	1	1.675565	1	0	1
Bruan_RCC_pre_aPD1_combo_tpm	P66287-09C-Run1_S12_L001	44.58316	1	7.655031	1	1	0
Bruan_RCC_pre_aPD1_combo_tpm	EA595635	3.679671	1	1.01848	1	0	1
Bruan_RCC_pre_aPD1_combo_tpm	EA595654	58.57906	0	11.26899	1	1	0
Bruan_RCC_pre_aPD1_combo_tpm	P66282-04A-Run1_S14_L002	45.17454	1	11.03901	1	1	0
Bruan_RCC_pre_aPD1_combo_tpm	EA595624	17.83984	0	11.82752	1	1	0
Bruan_RCC_pre_aPD1_combo_tpm	EA595454	37.8152	1	11.10472	1	0	0
Bruan_RCC_pre_aPD1_combo_tpm	P66291-09D-Run1_S11_L001	19.58111	1	5.585216	1	0	0
Bruan_RCC_pre_aPD1_combo_tpm	EA632234	16.91992	1	8.344969	1	0	0
Bruan_RCC_pre_aPD1_combo_tpm	P66511-07H-Run1_S19_L002	8.279261	1	3.778234	1	0	0
Bruan_RCC_pre_aPD1_combo_tpm	EA647826	25.98768	1	5.782341	1	1	0
Bruan_RCC_pre_aPD1_combo_tpm	P66451-07A-Run1_S15_L002	16.7885	1	1.905544	1	0	1
Bruan_RCC_pre_aPD1_combo_tpm	P66511-06F-Run1_S15_L001	3.022587	1	1.511294	1	0	1
Bruan_RCC_pre_aPD1_combo_tpm	P66287-02A-Run1_S5_L001	50.75975	0	46.09446	0	1	0
Bruan_RCC_pre_aPD1_combo_tpm	P66282-03D-Run1_S11_L001	5.519507	0	1.806982	1	0	1
Bruan_RCC_pre_aPD1_combo_tpm	P66287-11B-Run1_S21_L002	9.330595	1	9.330595	1	1	0
Bruan_RCC_pre_aPD1_combo_tpm	P66287-08B-Run1_S7_L001	13.33881	1	1.741273	1	0	1
Bruan_RCC_pre_aPD1_combo_tpm	EA639040	34.03696	1	1.675565	1	0	1
Bruan_RCC_pre_aPD1_combo_tpm	P66507-04D-Run1_S3_L001	55.35934	0	10.15195	0	1	0
Bruan_RCC_pre_aPD1_combo_tpm	P66507-07G-Run1_S17_L002	55.12936	0	5.585216	1	1	0
Bruan_RCC_pre_aPD1_combo_tpm	P66425-05B-Run1_S21_L002	63.50719	0	1.64271	1	0	1
Bruan_RCC_pre_aPD1_combo_tpm	P66425-03B-Run1_S10_L001	9.593429	1	3.581109	1	0	0
Bruan_RCC_pre_aPD1_combo_tpm	EA639069	54.80082	0	1.87269	1	0	1
Bruan_RCC_pre_aPD1_combo_tpm	P66282-11G-Run1_S20_L002	2.004107	1	1.971253	1	0	1
Bruan_RCC_pre_aPD1_combo_tpm	RCC_102_T_SCR	5.424658	0	3.550685	1	0	0
Bruan_RCC_pre_aPD1_combo_tpm	RCC_105_T_SCR	3.879452	1	1.479452	1	0	1

Bruan_RCC_pre_aPD1_combo_tpm	RCC_106_T_SCR	28.60274	0	26.99178	0	1	0
Bruan_RCC_pre_aPD1_combo_tpm	RCC_115_T_SCR	12.03288	0	2.860274	1	0	0
Bruan_RCC_pre_aPD1_combo_tpm	RCC_117_T_SCR	2.10411	0	1.249315	0	0	0
Bruan_RCC_pre_aPD1_combo_tpm	RCC_14_T_SCR	33.69863	0	17.7863	1	1	0
Bruan_RCC_pre_aPD1_combo_tpm	RCC_18_T_SCR	3.649315	0	1.413699	1	0	1
Bruan_RCC_pre_aPD1_combo_tpm	RCC_21_T_SCR	11.47397	0	1.413699	0	0	0
Bruan_RCC_pre_aPD1_combo_tpm	RCC_27_T_SCR	31.82466	0	1.150685	1	0	1
Bruan_RCC_pre_aPD1_combo_tpm	RCC_41_T_SCR	6.410959	0	1.380822	1	0	1
Bruan_RCC_pre_aPD1_combo_tpm	RCC_50_T_SCR	32.28493	0	2.827397	1	0	0
Bruan_RCC_pre_aPD1_combo_tpm	RCC_73_T_SCR	5.852055	1	1.906849	1	0	1
Bruan_RCC_pre_aPD1_combo_tpm	RCC_90_T_SCR	16.40548	1	1.183562	1	0	1
Bruan_RCC_pre_aPD1_combo_tpm	RCC_93_T_SCR	22.48767	1	16.43836	1	1	0
Bruan_RCC_pre_aPD1_combo_tpm	RCC_96_T_SCR	19.13425	0	1.216438	1	0	1
Bruan_RCC_pre_aPD1_combo_tpm	RCC_99_T_SCR	1.347945	1	1.347945	1	0	1
Mariathasan_UC_pre_aPDL1_combo_tpm	SAMf2ce197162ce	1.577002	1	NA	NA	0	0
Mariathasan_UC_pre_aPDL1_combo_tpm	SAM698d8d76b934	0.427105	1	NA	NA	0	0
Mariathasan_UC_pre_aPDL1_combo_tpm	SAMc1b27bc16435	24.1807	0	NA	NA	1	0
Mariathasan_UC_pre_aPDL1_combo_tpm	SAM85e41e7f33f9	0.62423	1	NA	NA	0	0
Mariathasan_UC_pre_aPDL1_combo_tpm	SAMf275eb859a39	24.47639	0	NA	NA	1	0
Mariathasan_UC_pre_aPDL1_combo_tpm	SAM7f0d9cc7f001	4.632444	1	NA	NA	0	1
Mariathasan_UC_pre_aPDL1_combo_tpm	SAM4305ab968b90	1.412731	1	NA	NA	0	1
Mariathasan_UC_pre_aPDL1_combo_tpm	SAMcf018fee2acd	16.22998	1	NA	NA	0	1
Mariathasan_UC_pre_aPDL1_combo_tpm	SAMb2f1d0e54ece	11.40041	1	NA	NA	1	0
Mariathasan_UC_pre_aPDL1_combo_tpm	SAMcc4675f394a1	3.12115	1	NA	NA	0	1
Mariathasan_UC_pre_aPDL1_combo_tpm	SAM49f9b2e57aa5	7.457906	1	NA	NA	0	1
Mariathasan_UC_pre_aPDL1_combo_tpm	SAM2e7aa8fa0ab3	7.720739	1	NA	NA	0	1
Mariathasan_UC_pre_aPDL1_combo_tpm	SAMd0e47be700b0	0.887064	1	NA	NA	0	0
Mariathasan_UC_pre_aPDL1_combo_tpm	SAMdf3e42c8672a	7.326489	1	NA	NA	0	1
Mariathasan_UC_pre_aPDL1_combo_tpm	SAMd027124354ce	23.52361	0	NA	NA	1	0
Mariathasan_UC_pre_aPDL1_combo_tpm	SAM36d87392593b	23.68789	0	NA	NA	0	0
Mariathasan_UC_pre_aPDL1_combo_tpm	SAM4edbe45817b3	1.051335	1	NA	NA	0	0
Mariathasan_UC_pre_aPDL1_combo_tpm	SAM36a9225b0222	23.8193	0	NA	NA	0	0
Mariathasan_UC_pre_aPDL1_combo_tpm	SAMe7bf6c015192	3.12115	1	NA	NA	0	1
Mariathasan_UC_pre_aPDL1_combo_tpm	SAM6dd7ad1d797d	19.28542	1	NA	NA	0	1
Mariathasan_UC_pre_aPDL1_combo_tpm	SAMc0d625a50eb8	13.2731	1	NA	NA	0	0
Mariathasan_UC_pre_aPDL1_combo_tpm	SAM18039827e1b9	1.478439	1	NA	NA	0	1
Mariathasan_UC_pre_aPDL1_combo_tpm	SAM681e4bf7cf85	23.3922	0	NA	NA	1	0
Mariathasan_UC_pre_aPDL1_combo_tpm	SAMc692536a795a	5.650924	1	NA	NA	0	1
Mariathasan_UC_pre_aPDL1_combo_tpm	SAM9a2cf3c06fb3	5.650924	1	NA	NA	0	1
Mariathasan_UC_pre_aPDL1_combo_tpm	SAM557dde1b9f3e	6.702259	1	NA	NA	0	1
Mariathasan_UC_pre_aPDL1_combo_tpm	SAMb8f13a0525a6	2.694045	1	NA	NA	0	0
Mariathasan_UC_pre_aPDL1_combo_tpm	SAM23aa15d4a0b0	5.716632	1	NA	NA	0	1
Mariathasan_UC_pre_aPDL1_combo_tpm	SAM468a9e1dc821	3.876797	1	NA	NA	0	1
Mariathasan_UC_pre_aPDL1_combo_tpm	SAM81b71522417a	2.135524	1	NA	NA	0	1

Mariathasan_UC_pre_aPDL1_combo_tpm	SAM6cb230f208a8	23.22793	0	NA	NA	1	0
Mariathasan_UC_pre_aPDL1_combo_tpm	SAM0684af734db1	23.26078	0	NA	NA	1	0
Mariathasan_UC_pre_aPDL1_combo_tpm	SAMb963dda93cfd	2.759754	1	NA	NA	0	1
Mariathasan_UC_pre_aPDL1_combo_tpm	SAMa9ca8536d2b1	21.84805	0	NA	NA	0	0
Mariathasan_UC_pre_aPDL1_combo_tpm	SAM9fb814c22bdb	19.12115	1	NA	NA	0	0
Mariathasan_UC_pre_aPDL1_combo_tpm	SAMbcbc7957c264	4.172485	1	NA	NA	0	1
Mariathasan_UC_pre_aPDL1_combo_tpm	SAM7fb6987514a4	22.83368	0	NA	NA	0	1
Mariathasan_UC_pre_aPDL1_combo_tpm	SAM63405b04ab2d	6.800821	1	NA	NA	0	1
Mariathasan_UC_pre_aPDL1_combo_tpm	SAM18bc1078bc15	6.899384	1	NA	NA	0	1
Mariathasan_UC_pre_aPDL1_combo_tpm	SAM7d2dfba6cd84	12.71458	1	NA	NA	0	0
Mariathasan_UC_pre_aPDL1_combo_tpm	SAMd1bd63734394	1.938398	1	NA	NA	0	1
Mariathasan_UC_pre_aPDL1_combo_tpm	SAMe9ae8beb82fa	5.486653	1	NA	NA	0	1
Mariathasan_UC_pre_aPDL1_combo_tpm	SAMb0a83e5fbde9	23.32649	0	NA	NA	0	0
Mariathasan_UC_pre_aPDL1_combo_tpm	SAMba7176afe070	3.449692	0	NA	NA	0	1
Mariathasan_UC_pre_aPDL1_combo_tpm	SAMae1690469964	21.15811	1	NA	NA	0	0
Mariathasan_UC_pre_aPDL1_combo_tpm	SAMbe83eae4026e	2.726899	1	NA	NA	0	1
Mariathasan_UC_pre_aPDL1_combo_tpm	SAMe5bc41772bc9	6.110883	1	NA	NA	0	1
Mariathasan_UC_pre_aPDL1_combo_tpm	SAM23095936e611	2.234086	1	NA	NA	0	1
Mariathasan_UC_pre_aPDL1_combo_tpm	SAM7114d99032ec	10.84189	1	NA	NA	0	1
Mariathasan_UC_pre_aPDL1_combo_tpm	SAMd215b503f99a	1.708419	0	NA	NA	0	0
Mariathasan_UC_pre_aPDL1_combo_tpm	SAMdb3f50c9129c	15.80287	1	NA	NA	0	1
Mariathasan_UC_pre_aPDL1_combo_tpm	SAMbf1a3ae828e6	20.73101	0	NA	NA	1	0
Mariathasan_UC_pre_aPDL1_combo_tpm	SAM52e3fa3ad574	7.950719	1	NA	NA	0	1
Mariathasan_UC_pre_aPDL1_combo_tpm	SAMd4c0837b0997	5.38809	1	NA	NA	0	1
Mariathasan_UC_pre_aPDL1_combo_tpm	SAM9cafb905b36a	7.622177	1	NA	NA	0	1
Mariathasan_UC_pre_aPDL1_combo_tpm	SAM3f2033c90438	0.821355	1	NA	NA	0	0
Mariathasan_UC_pre_aPDL1_combo_tpm	SAM032c642382a7	2.49692	1	NA	NA	0	1
Mariathasan_UC_pre_aPDL1_combo_tpm	SAM5ffd7e4cd794	1.215606	1	NA	NA	0	0
Mariathasan_UC_pre_aPDL1_combo_tpm	SAMb419a8fcbfcd	22.73511	0	NA	NA	1	0
Mariathasan_UC_pre_aPDL1_combo_tpm	SAM8884fe446d20	23.16222	0	NA	NA	1	0
Mariathasan_UC_pre_aPDL1_combo_tpm	SAM17c45bf16bb6	2.694045	1	NA	NA	0	0
Mariathasan_UC_pre_aPDL1_combo_tpm	SAM2070b416069c	8.016427	1	NA	NA	0	0
Mariathasan_UC_pre_aPDL1_combo_tpm	SAM0ce9c983b20f	6.603696	1	NA	NA	0	1
Mariathasan_UC_pre_aPDL1_combo_tpm	SAMa1e62d323e1d	10.57906	1	NA	NA	0	0
Mariathasan_UC_pre_aPDL1_combo_tpm	SAM61baf919bb01	15.60575	1	NA	NA	0	0
Mariathasan_UC_pre_aPDL1_combo_tpm	SAM97a00e0929fb	12.71458	0	NA	NA	1	0
Mariathasan_UC_pre_aPDL1_combo_tpm	SAM2f228939632f	7.88501	1	NA	NA	0	1
Mariathasan_UC_pre_aPDL1_combo_tpm	SAM9539a4f19ebc	7.950719	1	NA	NA	0	0
Mariathasan_UC_pre_aPDL1_combo_tpm	SAM36851bc8b9ae	3.548255	1	NA	NA	0	0
Mariathasan_UC_pre_aPDL1_combo_tpm	SAM297c0301e861	8.082136	1	NA	NA	0	1
Mariathasan_UC_pre_aPDL1_combo_tpm	SAM1fa6bcb7fc48	22.73511	0	NA	NA	0	1
Mariathasan_UC_pre_aPDL1_combo_tpm	SAM075e037d95bc	21.32238	0	NA	NA	1	0
Mariathasan_UC_pre_aPDL1_combo_tpm	SAM6d2ae0c39b96	22.11088	0	NA	NA	0	1
Mariathasan_UC_pre_aPDL1_combo_tpm	SAMcabb6d58ff55	20.27105	0	NA	NA	0	0

Mariathasan_UC_pre_aPDL1_combo_tpm	SAM716f54e468f4	21.19097	0	NA	NA	0	0
Mariathasan_UC_pre_aPDL1_combo_tpm	SAMeff2ce356ccb	8.082136	1	NA	NA	0	1
Mariathasan_UC_pre_aPDL1_combo_tpm	SAM110501d0eedb	4.271047	1	NA	NA	0	1
Mariathasan_UC_pre_aPDL1_combo_tpm	SAM2e9ac0b1b250	22.14374	0	NA	NA	1	0
Mariathasan_UC_pre_aPDL1_combo_tpm	SAMc0ef41aa6c8b	9.560575	1	NA	NA	0	1
Mariathasan_UC_pre_aPDL1_combo_tpm	SAMd636e3461955	10.48049	1	NA	NA	0	1
Mariathasan_UC_pre_aPDL1_combo_tpm	SAMe712352fb82a	22.11088	0	NA	NA	1	0
Mariathasan_UC_pre_aPDL1_combo_tpm	SAM30b5c6c54cf7	9.264887	1	NA	NA	1	0
Mariathasan_UC_pre_aPDL1_combo_tpm	SAMd98bac0a070f	2.069815	1	NA	NA	0	1
Mariathasan_UC_pre_aPDL1_combo_tpm	SAM8e8ef2368dfa	4.501027	1	NA	NA	0	1
Mariathasan_UC_pre_aPDL1_combo_tpm	SAM08cce2fa88f2	22.11088	0	NA	NA	0	0
Mariathasan_UC_pre_aPDL1_combo_tpm	SAMb4c7a001537d	3.186858	1	NA	NA	0	1
Mariathasan_UC_pre_aPDL1_combo_tpm	SAMfd947610629d	9.034908	1	NA	NA	0	1
Mariathasan_UC_pre_aPDL1_combo_tpm	SAM943df5cf15df	2.135524	1	NA	NA	0	1
Mariathasan_UC_pre_aPDL1_combo_tpm	SAM31f41dd0d6ca	8.279261	1	NA	NA	0	0
Mariathasan_UC_pre_aPDL1_combo_tpm	SAMb3c02294aba7	22.40657	0	NA	NA	1	0
Mariathasan_UC_pre_aPDL1_combo_tpm	SAM39eb94fa504d	17.90554	1	NA	NA	0	1
Mariathasan_UC_pre_aPDL1_combo_tpm	SAM62fb1388c871	22.50513	0	NA	NA	0	1
Mariathasan_UC_pre_aPDL1_combo_tpm	SAMc0da5d48686d	9.494867	1	NA	NA	0	1
Mariathasan_UC_pre_aPDL1_combo_tpm	SAMe7bcab05402e	7.917864	1	NA	NA	0	0
Mariathasan_UC_pre_aPDL1_combo_tpm	SAM2570ff4aae6e	22.37372	0	NA	NA	1	0
Mariathasan_UC_pre_aPDL1_combo_tpm	SAM2dc3f04e45e9	10.1848	1	NA	NA	0	1
Mariathasan_UC_pre_aPDL1_combo_tpm	SAM9448d858692c	2.036961	1	NA	NA	0	0
Mariathasan_UC_pre_aPDL1_combo_tpm	SAM4501e41e4751	1.117043	1	NA	NA	0	1
Mariathasan_UC_pre_aPDL1_combo_tpm	SAM714285adf612	0.62423	1	NA	NA	0	0
Mariathasan_UC_pre_aPDL1_combo_tpm	SAM5b57e47fdcb3	3.843943	1	NA	NA	0	1
Mariathasan_UC_pre_aPDL1_combo_tpm	SAMae4da274eded	4.99384	1	NA	NA	0	1
Mariathasan_UC_pre_aPDL1_combo_tpm	SAMd35318127278	21.58522	0	NA	NA	1	0
Mariathasan_UC_pre_aPDL1_combo_tpm	SAMa913c6139ec8	5.125257	1	NA	NA	0	0
Mariathasan_UC_pre_aPDL1_combo_tpm	SAM75142fcab9df	11.92608	1	NA	NA	0	1
Mariathasan_UC_pre_aPDL1_combo_tpm	SAMd697ba701077	21.15811	1	NA	NA	0	0
Mariathasan_UC_pre_aPDL1_combo_tpm	SAM166a419a4e5a	5.38809	1	NA	NA	0	1
Mariathasan_UC_pre_aPDL1_combo_tpm	SAM025b45c27e05	8.772074	1	NA	NA	0	1
Mariathasan_UC_pre_aPDL1_combo_tpm	SAMe07c4560772d	2.102669	0	NA	NA	0	1
Mariathasan_UC_pre_aPDL1_combo_tpm	SAM2dc578e0165f	1.971253	1	NA	NA	0	0
Mariathasan_UC_pre_aPDL1_combo_tpm	SAM3785587846ce	7.063655	1	NA	NA	0	0
Mariathasan_UC_pre_aPDL1_combo_tpm	SAM560f23d6a3ad	21.38809	0	NA	NA	1	0
Mariathasan_UC_pre_aPDL1_combo_tpm	SAM52500cabdd36	20.46817	0	NA	NA	0	0
Mariathasan_UC_pre_aPDL1_combo_tpm	SAM14df63a65411	21.42094	0	NA	NA	1	0
Mariathasan_UC_pre_aPDL1_combo_tpm	SAMf3a9bce50099	2.102669	0	NA	NA	0	1
Mariathasan_UC_pre_aPDL1_combo_tpm	SAMffa5c7cad0e5	15.54004	1	NA	NA	1	0
Mariathasan_UC_pre_aPDL1_combo_tpm	SAMdab9ca8fb5de	0.197125	0	NA	NA	0	1
Mariathasan_UC_pre_aPDL1_combo_tpm	SAM34430ef08e5b	18.16838	0	NA	NA	0	1
Mariathasan_UC_pre_aPDL1_combo_tpm	SAM4b0175e8db6e	2.069815	1	NA	NA	0	1

Mariathasan_UC_pre_aPDL1_combo_tpm	SAMf28c01545593	6.406571	1	NA	NA	0	0
Mariathasan_UC_pre_aPDL1_combo_tpm	SAMaec7380f9ab0	20.86242	0	NA	NA	1	0
Mariathasan_UC_pre_aPDL1_combo_tpm	SAMaf7578d55754	0.229979	1	NA	NA	0	0
Mariathasan_UC_pre_aPDL1_combo_tpm	SAMbd8ee73983b8	0.854209	1	NA	NA	0	0
Mariathasan_UC_pre_aPDL1_combo_tpm	SAMe94c30c30616	15.37577	1	NA	NA	0	0
Mariathasan_UC_pre_aPDL1_combo_tpm	SAM54e58f1b0230	4.36961	1	NA	NA	0	1
Mariathasan_UC_pre_aPDL1_combo_tpm	SAM5e3bae090b8c	6.735113	1	NA	NA	0	1
Mariathasan_UC_pre_aPDL1_combo_tpm	SAM3cb94b0d5297	5.059548	1	NA	NA	0	1
Mariathasan_UC_pre_aPDL1_combo_tpm	SAM61b9d4d84c64	21.38809	0	NA	NA	0	0
Mariathasan_UC_pre_aPDL1_combo_tpm	SAM8a42c0d59187	2.825462	1	NA	NA	0	0
Mariathasan_UC_pre_aPDL1_combo_tpm	SAMcb132b0cdd2c	2.529774	1	NA	NA	0	1
Mariathasan_UC_pre_aPDL1_combo_tpm	SAMe97af0feefdf	11.10472	1	NA	NA	0	1
Mariathasan_UC_pre_aPDL1_combo_tpm	SAMee3844cc0b9f	3.219713	1	NA	NA	0	1
Mariathasan_UC_pre_aPDL1_combo_tpm	SAM73b653ae20d1	1.806982	1	NA	NA	0	0
Mariathasan_UC_pre_aPDL1_combo_tpm	SAMdcae54fcd7fa	6.2423	1	NA	NA	0	0
Mariathasan_UC_pre_aPDL1_combo_tpm	SAMdad5c29dc105	2.431211	1	NA	NA	0	1
Mariathasan_UC_pre_aPDL1_combo_tpm	SAMa424c75831b4	3.548255	1	NA	NA	0	1
Mariathasan_UC_pre_aPDL1_combo_tpm	SAM28687037e4ff	21.58522	0	NA	NA	0	0
Mariathasan_UC_pre_aPDL1_combo_tpm	SAMe50d15fde368	18.89117	0	NA	NA	1	0
Mariathasan_UC_pre_aPDL1_combo_tpm	SAM45c8e6412c66	3.482546	1	NA	NA	0	1
Mariathasan_UC_pre_aPDL1_combo_tpm	SAM8533e5e261d6	12.81314	1	NA	NA	1	0
Mariathasan_UC_pre_aPDL1_combo_tpm	SAMaaf505c36f93	20.56674	0	NA	NA	1	0
Mariathasan_UC_pre_aPDL1_combo_tpm	SAM73663ee4a96e	21.25667	0	NA	NA	1	0
Mariathasan_UC_pre_aPDL1_combo_tpm	SAM0bdb3428bd13	2.234086	1	NA	NA	0	1
Mariathasan_UC_pre_aPDL1_combo_tpm	SAM3330c03fdf00	19.18686	0	NA	NA	0	1
Mariathasan_UC_pre_aPDL1_combo_tpm	SAM0a7c2091dd56	2.628337	1	NA	NA	0	1
Mariathasan_UC_pre_aPDL1_combo_tpm	SAM7bff231634e9	10.25051	1	NA	NA	0	0
Mariathasan_UC_pre_aPDL1_combo_tpm	SAM5d1dfd5207f5	10.9076	1	NA	NA	0	1
Mariathasan_UC_pre_aPDL1_combo_tpm	SAMeb587a68006b	20.69815	0	NA	NA	0	0
Mariathasan_UC_pre_aPDL1_combo_tpm	SAM99a46b9eec27	3.613963	1	NA	NA	0	1
Mariathasan_UC_pre_aPDL1_combo_tpm	SAM1ac4e3dee297	5.519507	1	NA	NA	0	1
Mariathasan_UC_pre_aPDL1_combo_tpm	SAM553c3c35b847	6.702259	1	NA	NA	0	1
Mariathasan_UC_pre_aPDL1_combo_tpm	SAM3e04eb914f3d	17.28131	0	NA	NA	1	0
Mariathasan_UC_pre_aPDL1_combo_tpm	SAMc2a1820d4e6b	20.73101	0	NA	NA	1	0
Mariathasan_UC_pre_aPDL1_combo_tpm	SAM822b226466a1	17.28131	0	NA	NA	0	0
Mariathasan_UC_pre_aPDL1_combo_tpm	SAMabc151b01ea3	0.558522	1	NA	NA	0	1
Mariathasan_UC_pre_aPDL1_combo_tpm	SAMe1eb5d988760	21.05955	0	NA	NA	1	0
Mariathasan_UC_pre_aPDL1_combo_tpm	SAMe3210d3632b4	2.168378	1	NA	NA	0	1
Mariathasan_UC_pre_aPDL1_combo_tpm	SAMb15ad09d6e24	3.876797	1	NA	NA	0	1
Mariathasan_UC_pre_aPDL1_combo_tpm	SAM7893196e0e89	2.661191	1	NA	NA	0	1
Mariathasan_UC_pre_aPDL1_combo_tpm	SAM3f446449bf81	0.755647	1	NA	NA	0	0
Mariathasan_UC_pre_aPDL1_combo_tpm	SAM961d04c42bd9	5.880903	1	NA	NA	0	1
Mariathasan_UC_pre_aPDL1_combo_tpm	SAMb0d11db9aa79	10.5462	1	NA	NA	0	1
Mariathasan_UC_pre_aPDL1_combo_tpm	SAM19fec8f3b3bd	9.889117	1	NA	NA	0	1

Mariathasan_UC_pre_aPDL1_combo_tpm	SAM6083aac8db99	20.63244	0	NA	NA	1	0
Mariathasan_UC_pre_aPDL1_combo_tpm	SAM7ee2b6e4d6b3	1.708419	1	NA	NA	0	0
Mariathasan_UC_pre_aPDL1_combo_tpm	SAMa90d73f8d891	1.01848	1	NA	NA	0	1
Mariathasan_UC_pre_aPDL1_combo_tpm	SAMaff272833538	2.135524	1	NA	NA	0	0
Mariathasan_UC_pre_aPDL1_combo_tpm	SAM415f36ad349e	17.97125	1	NA	NA	0	1
Mariathasan_UC_pre_aPDL1_combo_tpm	SAM7c67b05aa109	8.246407	1	NA	NA	0	0
Mariathasan_UC_pre_aPDL1_combo_tpm	SAMad83c9c53537	14.98152	0	NA	NA	0	1
Mariathasan_UC_pre_aPDL1_combo_tpm	SAM3e8baff50d7a	6.965092	1	NA	NA	0	0
Mariathasan_UC_pre_aPDL1_combo_tpm	SAM3ee5dcd894f0	8.706366	1	NA	NA	0	1
Mariathasan_UC_pre_aPDL1_combo_tpm	SAM8a1b0e02ee42	20.56674	0	NA	NA	1	0
Mariathasan_UC_pre_aPDL1_combo_tpm	SAMd7d57ee3a863	20.27105	0	NA	NA	1	0
Mariathasan_UC_pre_aPDL1_combo_tpm	SAMeb29625f76a5	3.12115	1	NA	NA	0	1
Mariathasan_UC_pre_aPDL1_combo_tpm	SAM563d6233dfa2	2.135524	1	NA	NA	0	1
Mariathasan_UC_pre_aPDL1_combo_tpm	SAM4581bac493af	20.04107	0	NA	NA	0	0
Mariathasan_UC_pre_aPDL1_combo_tpm	SAMa1871f491b02	1.741273	1	NA	NA	0	1
Mariathasan_UC_pre_aPDL1_combo_tpm	SAM30cf07d4874f	11.66324	1	NA	NA	0	0
Mariathasan_UC_pre_aPDL1_combo_tpm	SAM31291c256373	1.149897	1	NA	NA	0	0
Mariathasan_UC_pre_aPDL1_combo_tpm	SAMd135d5867fe3	3.613963	1	NA	NA	0	1
Mariathasan_UC_pre_aPDL1_combo_tpm	SAM9e11ec6bea80	15.40862	1	NA	NA	0	0
Mariathasan_UC_pre_aPDL1_combo_tpm	SAM065890737112	20.07392	0	NA	NA	1	0
Mariathasan_UC_pre_aPDL1_combo_tpm	SAMb470eb8f04be	20.07392	0	NA	NA	1	0
Mariathasan_UC_pre_aPDL1_combo_tpm	SAM675a12a09c15	2.89117	1	NA	NA	0	1
Mariathasan_UC_pre_aPDL1_combo_tpm	SAM1e9c4d1d39ae	12.41889	1	NA	NA	0	1
Mariathasan_UC_pre_aPDL1_combo_tpm	SAM5a2347c0498a	4.895277	1	NA	NA	0	0
Mariathasan_UC_pre_aPDL1_combo_tpm	SAM0571f17f4045	4.533881	1	NA	NA	0	0
Mariathasan_UC_pre_aPDL1_combo_tpm	SAM4b7ea015fd9e	13.2731	1	NA	NA	0	1
Mariathasan_UC_pre_aPDL1_combo_tpm	SAM9306c5c92444	15.44148	0	NA	NA	0	1
Mariathasan_UC_pre_aPDL1_combo_tpm	SAMb15ac6e4c4ef	20.00821	0	NA	NA	1	0
Mariathasan_UC_pre_aPDL1_combo_tpm	SAM28e6031ac18b	9.232033	1	NA	NA	0	0
Mariathasan_UC_pre_aPDL1_combo_tpm	SAMd86389d0d768	2.201232	1	NA	NA	0	1
Mariathasan_UC_pre_aPDL1_combo_tpm	SAM63b2189c36d7	3.975359	1	NA	NA	0	1
Mariathasan_UC_pre_aPDL1_combo_tpm	SAM18be5b395318	9.002053	1	NA	NA	0	1
Mariathasan_UC_pre_aPDL1_combo_tpm	SAM0d855cff64e6	2.858316	1	NA	NA	0	1
Mariathasan_UC_pre_aPDL1_combo_tpm	SAM6cbc10abddb0	5.946612	1	NA	NA	0	1
Mariathasan_UC_pre_aPDL1_combo_tpm	SAMdee1011782cd	3.909651	1	NA	NA	0	1
Mariathasan_UC_pre_aPDL1_combo_tpm	SAMe9475f77504b	18.66119	0	NA	NA	0	0
Mariathasan_UC_pre_aPDL1_combo_tpm	SAMa535fcdf18a0	17.28131	0	NA	NA	1	0
Mariathasan_UC_pre_aPDL1_combo_tpm	SAM7746b76437e6	2.825462	1	NA	NA	0	0
Mariathasan_UC_pre_aPDL1_combo_tpm	SAM548551ef782c	17.28131	0	NA	NA	1	0
Mariathasan_UC_pre_aPDL1_combo_tpm	SAM203dcf14f927	19.35113	0	NA	NA	1	0
Mariathasan_UC_pre_aPDL1_combo_tpm	SAMe41b1e773582	0.854209	1	NA	NA	0	1
Mariathasan_UC_pre_aPDL1_combo_tpm	SAM978a587b207e	2.102669	1	NA	NA	0	1
Mariathasan_UC_pre_aPDL1_combo_tpm	SAM5234688806a7	5.880903	1	NA	NA	0	1
Mariathasan_UC_pre_aPDL1_combo_tpm	SAM2c9586161ce6	11.26899	1	NA	NA	0	1

Mariathasan_UC_pre_aPDL1_combo_tpm	SAM76a431ba6ce1	14.12731	1	NA	NA	0	1
Mariathasan_UC_pre_aPDL1_combo_tpm	SAM7a9093b9c7e9	1.806982	1	NA	NA	0	0
Mariathasan_UC_pre_aPDL1_combo_tpm	SAM8f2275c36e8c	20.73101	0	NA	NA	0	0
Mariathasan_UC_pre_aPDL1_combo_tpm	SAMab8052a03398	13.30595	1	NA	NA	0	0
Mariathasan_UC_pre_aPDL1_combo_tpm	SAMd3bd67996035	20.82957	0	NA	NA	1	0
Mariathasan_UC_pre_aPDL1_combo_tpm	SAMfddc359e862b	1.938398	1	NA	NA	0	0
Mariathasan_UC_pre_aPDL1_combo_tpm	SAM753d4bb52dbe	11.36756	1	NA	NA	0	0
Mariathasan_UC_pre_aPDL1_combo_tpm	SAMd3601288319e	23.12936	0	NA	NA	1	0
Mariathasan_UC_pre_aPDL1_combo_tpm	SAM59f392864f5d	17.77413	1	NA	NA	0	0
Mariathasan_UC_pre_aPDL1_combo_tpm	SAM26104d5adc89	5.38809	1	NA	NA	0	0
Mariathasan_UC_pre_aPDL1_combo_tpm	SAMba1a34b5a060	12.846	1	NA	NA	0	1
Mariathasan_UC_pre_aPDL1_combo_tpm	SAM18a4dabbc557	16.45996	1	NA	NA	0	1
Mariathasan_UC_pre_aPDL1_combo_tpm	SAMfb7aec7cb0e2	22.53799	0	NA	NA	1	0
Mariathasan_UC_pre_aPDL1_combo_tpm	SAMfed609955db9	20.56674	0	NA	NA	0	1
Mariathasan_UC_pre_aPDL1_combo_tpm	SAMb8070b7937e7	21.02669	1	NA	NA	0	0
Mariathasan_UC_pre_aPDL1_combo_tpm	SAMf2aae1443f67	10.34908	1	NA	NA	0	1
Mariathasan_UC_pre_aPDL1_combo_tpm	SAM2bba8cb35e48	10.48049	1	NA	NA	0	1
Mariathasan_UC_pre_aPDL1_combo_tpm	SAMcee0fa8c05b4	1.675565	1	NA	NA	0	0
Mariathasan_UC_pre_aPDL1_combo_tpm	SAM5c139c5c1c4f	7.227926	1	NA	NA	0	1
Mariathasan_UC_pre_aPDL1_combo_tpm	SAMc1251c7bfee2	21.61807	0	NA	NA	1	0
Mariathasan_UC_pre_aPDL1_combo_tpm	SAM6780ed436b55	6.2423	1	NA	NA	0	1
Mariathasan_UC_pre_aPDL1_combo_tpm	SAM85f0a3ac1c45	5.848049	1	NA	NA	0	1
Mariathasan_UC_pre_aPDL1_combo_tpm	SAM9d2494119c05	1.445585	1	NA	NA	0	1
Mariathasan_UC_pre_aPDL1_combo_tpm	SAM9410b866974a	6.735113	1	NA	NA	0	0
Mariathasan_UC_pre_aPDL1_combo_tpm	SAM5d989c86255e	3.449692	1	NA	NA	0	1
Mariathasan_UC_pre_aPDL1_combo_tpm	SAM27299aed7681	18.85832	0	NA	NA	1	0
Mariathasan_UC_pre_aPDL1_combo_tpm	SAM49d48750e294	9.856263	1	NA	NA	0	1
Mariathasan_UC_pre_aPDL1_combo_tpm	SAMd43f8933066b	20.00821	0	NA	NA	1	0
Mariathasan_UC_pre_aPDL1_combo_tpm	SAM7aa01fc49a80	2.135524	1	NA	NA	0	1
Mariathasan_UC_pre_aPDL1_combo_tpm	SAM0257bbbbd388	15.6386	1	NA	NA	0	0
Mariathasan_UC_pre_aPDL1_combo_tpm	SAMc919aebc7fdd	20.73101	0	NA	NA	1	0
Mariathasan_UC_pre_aPDL1_combo_tpm	SAMa321770ac31c	7.063655	0	NA	NA	0	1
Mariathasan_UC_pre_aPDL1_combo_tpm	SAM3894ac3956a5	2.102669	0	NA	NA	0	1
Mariathasan_UC_pre_aPDL1_combo_tpm	SAM3779e979db6b	1.314168	1	NA	NA	0	0
Mariathasan_UC_pre_aPDL1_combo_tpm	SAM1c0ecfb3eb63	5.848049	1	NA	NA	0	1
Mariathasan_UC_pre_aPDL1_combo_tpm	SAMbf91f27e7f9b	0.591376	1	NA	NA	0	0
Mariathasan_UC_pre_aPDL1_combo_tpm	SAMbcb07ba81cee	2.529774	1	NA	NA	0	1
Mariathasan_UC_pre_aPDL1_combo_tpm	SAM9aa6a095a9d6	8.837782	1	NA	NA	0	1
Mariathasan_UC_pre_aPDL1_combo_tpm	SAM957378bd907f	2.234086	1	NA	NA	0	1
Mariathasan_UC_pre_aPDL1_combo_tpm	SAMf82bbdc267c8	3.12115	1	NA	NA	0	1
Mariathasan_UC_pre_aPDL1_combo_tpm	SAM2624229effe8	2.562628	1	NA	NA	0	1
Mariathasan_UC_pre_aPDL1_combo_tpm	SAMd2492b2a31bb	7.852156	1	NA	NA	0	1
Mariathasan_UC_pre_aPDL1_combo_tpm	SAM670649e105b5	22.07803	0	NA	NA	1	0
Mariathasan_UC_pre_aPDL1_combo_tpm	SAM91c47b054ffb	17.01848	1	NA	NA	1	0

Mariathasan_UC_pre_aPDL1_combo_tpm	SAM491e341d5a82	1.87269	1	NA	NA	0	0
Mariathasan_UC_pre_aPDL1_combo_tpm	SAM5fe7a81a39dd	17.08419	1	NA	NA	0	1
Mariathasan_UC_pre_aPDL1_combo_tpm	SAM29da928587ad	2.595483	1	NA	NA	0	1
Mariathasan_UC_pre_aPDL1_combo_tpm	SAM0a0f2bac4b20	1.182752	1	NA	NA	0	1
Mariathasan_UC_pre_aPDL1_combo_tpm	SAM8e469834acc1	3.712526	1	NA	NA	0	1
Mariathasan_UC_pre_aPDL1_combo_tpm	SAM8e43e9caf307	0.492813	1	NA	NA	0	0
Mariathasan_UC_pre_aPDL1_combo_tpm	SAM99b1f6a9534e	0.492813	1	NA	NA	0	0
Mariathasan_UC_pre_aPDL1_combo_tpm	SAM12502d970c10	7.392197	1	NA	NA	0	0
Mariathasan_UC_pre_aPDL1_combo_tpm	SAM9681450bbc90	4.960986	1	NA	NA	0	0
Mariathasan_UC_pre_aPDL1_combo_tpm	SAM7d7c54623618	21.22382	0	NA	NA	1	0
Mariathasan_UC_pre_aPDL1_combo_tpm	SAM181b638b8248	10.87474	1	NA	NA	0	1
Mariathasan_UC_pre_aPDL1_combo_tpm	SAMbfdffb97c446	6.702259	1	NA	NA	0	0
Mariathasan_UC_pre_aPDL1_combo_tpm	SAM3b15b4c6311d	21.4538	0	NA	NA	1	0
Mariathasan_UC_pre_aPDL1_combo_tpm	SAM00b9e5c52da9	1.905544	1	NA	NA	0	0
Mariathasan_UC_pre_aPDL1_combo_tpm	SAM59b825252c0d	0.525667	1	NA	NA	0	0
Mariathasan_UC_pre_aPDL1_combo_tpm	SAM1c8b086175ca	20.76386	1	NA	NA	0	1
Mariathasan_UC_pre_aPDL1_combo_tpm	SAMeaa477a5384b	21.58522	0	NA	NA	1	0
Mariathasan_UC_pre_aPDL1_combo_tpm	SAM1dda30f1c5be	21.25667	0	NA	NA	0	1
Mariathasan_UC_pre_aPDL1_combo_tpm	SAM9eebdef2858a	0.821355	1	NA	NA	0	0
Mariathasan_UC_pre_aPDL1_combo_tpm	SAM9daccafc18db	16.45996	0	NA	NA	0	1
Mariathasan_UC_pre_aPDL1_combo_tpm	SAM2eb07dedf07f	20.50103	0	NA	NA	1	0
Mariathasan_UC_pre_aPDL1_combo_tpm	SAM25510f300d79	20.69815	0	NA	NA	1	0
Mariathasan_UC_pre_aPDL1_combo_tpm	SAMbda79f955628	20.63244	0	NA	NA	0	0
Mariathasan_UC_pre_aPDL1_combo_tpm	SAM9b9d48b0b02c	2.89117	1	NA	NA	0	0
Mariathasan_UC_pre_aPDL1_combo_tpm	SAMff41c4e8c08f	2.464066	1	NA	NA	0	0
Mariathasan_UC_pre_aPDL1_combo_tpm	SAMc97f35a29d16	0.821355	0	NA	NA	0	0
Mariathasan_UC_pre_aPDL1_combo_tpm	SAM187e056d6a2a	2.299795	1	NA	NA	0	1
Mariathasan_UC_pre_aPDL1_combo_tpm	SAMbc8dc3a7b54e	14.75154	1	NA	NA	0	1
Mariathasan_UC_pre_aPDL1_combo_tpm	SAM7b40007f4aa4	21.65092	0	NA	NA	1	0
Mariathasan_UC_pre_aPDL1_combo_tpm	SAM568ce160abd9	12.846	1	NA	NA	0	0
Mariathasan_UC_pre_aPDL1_combo_tpm	SAM14938611a2d3	21.42094	0	NA	NA	1	0
Mariathasan_UC_pre_aPDL1_combo_tpm	SAM1a87df750b9d	5.38809	1	NA	NA	0	0
Mariathasan_UC_pre_aPDL1_combo_tpm	SAMae02629a97f7	3.252567	1	NA	NA	0	1
Mariathasan_UC_pre_aPDL1_combo_tpm	SAM47fc46c3d6be	18.7269	0	NA	NA	0	0
Mariathasan_UC_pre_aPDL1_combo_tpm	SAM7829a341b9f3	3.876797	1	NA	NA	0	1
Mariathasan_UC_pre_aPDL1_combo_tpm	SAM0f956e757453	10.41478	1	NA	NA	0	1
Mariathasan_UC_pre_aPDL1_combo_tpm	SAMaf42c1541269	5.519507	0	NA	NA	0	0
Mariathasan_UC_pre_aPDL1_combo_tpm	SAM09c84ec0cf34	6.505133	1	NA	NA	0	1
Mariathasan_UC_pre_aPDL1_combo_tpm	SAM7538ad9ff524	19.12115	0	NA	NA	0	0
Mariathasan_UC_pre_aPDL1_combo_tpm	SAM95c70496ffb5	0.361396	1	NA	NA	0	0
Mariathasan_UC_pre_aPDL1_combo_tpm	SAM7edacb3deb65	18.00411	0	NA	NA	0	0
Mariathasan_UC_pre_aPDL1_combo_tpm	SAMb2e4a082541a	4.36961	1	NA	NA	0	1
Mariathasan_UC_pre_aPDL1_combo_tpm	SAM771445e92421	19.41684	0	NA	NA	1	0
Mariathasan_UC_pre_aPDL1_combo_tpm	SAM59fda9035d1d	3.74538	1	NA	NA	0	1

Mariathasan_UC_pre_aPDL1_combo_tpm	SAM727c0e92a2a7	18.7269	0	NA	NA	1	0
Mariathasan_UC_pre_aPDL1_combo_tpm	SAM6f2a102a99df	19.28542	0	NA	NA	1	0
Mariathasan_UC_pre_aPDL1_combo_tpm	SAMe56c96c51190	9.7577	1	NA	NA	0	1
Mariathasan_UC_pre_aPDL1_combo_tpm	SAM2b672f4336c7	15.31006	1	NA	NA	0	0
Mariathasan_UC_pre_aPDL1_combo_tpm	SAM1ab1b28d9f2b	2.102669	1	NA	NA	0	1
Mariathasan_UC_pre_aPDL1_combo_tpm	SAM1bcc62d8290c	18.62834	0	NA	NA	0	0
Mariathasan_UC_pre_aPDL1_combo_tpm	SAM6662f5181f87	19.28542	0	NA	NA	0	1
Mariathasan_UC_pre_aPDL1_combo_tpm	SAMe0c49ea0df5d	4.13963	1	NA	NA	0	0
Mariathasan_UC_pre_aPDL1_combo_tpm	SAM59289ca42c99	1.708419	1	NA	NA	0	0
Mariathasan_UC_pre_aPDL1_combo_tpm	SAM18b9351e265a	15.86858	1	NA	NA	0	1
Mariathasan_UC_pre_aPDL1_combo_tpm	SAM7fb7a13c096b	1.675565	1	NA	NA	0	1
Mariathasan_UC_pre_aPDL1_combo_tpm	SAM87a8e18eb45b	2.759754	1	NA	NA	0	1
Mariathasan_UC_pre_aPDL1_combo_tpm	SAM1f3c93814cb9	1.445585	1	NA	NA	0	0
Mariathasan_UC_pre_aPDL1_combo_tpm	SAM5fc9ae0aed1f	0.62423	1	NA	NA	0	0
Mariathasan_UC_pre_aPDL1_combo_tpm	SAM9725303dce0c	8.903491	0	NA	NA	0	1
Mariathasan_UC_pre_aPDL1_combo_tpm	SAM6ff654a20f98	18.56263	0	NA	NA	1	0
Mariathasan_UC_pre_aPDL1_combo_tpm	SAM65afda25b920	8.082136	1	NA	NA	0	0
Mariathasan_UC_pre_aPDL1_combo_tpm	SAMef0e3d2415fd	10.1191	1	NA	NA	0	0
Mariathasan_UC_pre_aPDL1_combo_tpm	SAM07a93a28f801	12.846	0	NA	NA	1	0
Mariathasan_UC_pre_aPDL1_combo_tpm	SAM94859b440b1d	13.33881	1	NA	NA	0	1
Mariathasan_UC_pre_aPDL1_combo_tpm	SAM58e7832f4e7d	16.45996	0	NA	NA	1	0
Mariathasan_UC_pre_aPDL1_combo_tpm	SAMc57eadb2d82b	8.016427	1	NA	NA	0	1
Mariathasan_UC_pre_aPDL1_combo_tpm	SAMd5ab7fbfab4e	18.10267	0	NA	NA	0	0
Mariathasan_UC_pre_aPDL1_combo_tpm	SAM5767dd75d142	18.16838	0	NA	NA	1	0
Mariathasan_UC_pre_aPDL1_combo_tpm	SAMaabf4afe4213	2.628337	1	NA	NA	0	1
Mariathasan_UC_pre_aPDL1_combo_tpm	SAMcc7a42d87e9c	17.21561	0	NA	NA	1	0
Mariathasan_UC_pre_aPDL1_combo_tpm	SAMce39dd79b441	6.275154	1	NA	NA	0	1
Mariathasan_UC_pre_aPDL1_combo_tpm	SAM75f12d1a55fc	14.75154	0	NA	NA	0	0
Mariathasan_UC_pre_aPDL1_combo_tpm	SAM6964a6d7b967	16.26283	1	NA	NA	0	1
Mariathasan_UC_pre_aPDL1_combo_tpm	SAM3b1066e5801b	15.6386	0	NA	NA	0	0
Mariathasan_UC_pre_aPDL1_combo_tpm	SAMf20b827dca51	4.36961	1	NA	NA	0	1
Mariathasan_UC_pre_aPDL1_combo_tpm	SAM6792d6e98068	6.01232	1	NA	NA	0	1
Mariathasan_UC_pre_aPDL1_combo_tpm	SAM31d9176e11fb	17.11704	0	NA	NA	1	0
Mariathasan_UC_pre_aPDL1_combo_tpm	SAM04c589eb3fb3	0.689938	0	NA	NA	0	0
Mariathasan_UC_pre_aPDL1_combo_tpm	SAM5cc2d9036053	5.683778	1	NA	NA	0	1
Mariathasan_UC_pre_aPDL1_combo_tpm	SAMb8101c538753	13.40452	1	NA	NA	0	0
Mariathasan_UC_pre_aPDL1_combo_tpm	SAM80c6183220e6	14.75154	0	NA	NA	0	1
Mariathasan_UC_pre_aPDL1_combo_tpm	SAM572f19794c96	16.82136	0	NA	NA	1	0
Mariathasan_UC_pre_aPDL1_combo_tpm	SAM1abf01dd4544	16.85421	0	NA	NA	1	0
Mariathasan_UC_pre_aPDL1_combo_tpm	SAM1f83ebd6be9b	3.5154	1	NA	NA	0	1
Mariathasan_UC_pre_aPDL1_combo_tpm	SAM1f66db567eb5	16.59138	0	NA	NA	1	0
Mariathasan_UC_pre_aPDL1_combo_tpm	SAM4918c524b83a	0.62423	0	NA	NA	0	0
Mariathasan_UC_pre_aPDL1_combo_tpm	SAMa0ca029b7afd	0.361396	1	NA	NA	0	0
Mariathasan_UC_pre_aPDL1_combo_tpm	SAM6157c8f38b72	16.59138	0	NA	NA	1	0

Mariathasan_UC_pre_aPDL1_combo_tpm	SAMe7e4f7c076a7	1.971253	1	NA	NA	0	1
Mariathasan_UC_pre_aPDL1_combo_tpm	SAMbe25e2c88f3e	2.924025	1	NA	NA	0	0
Mariathasan_UC_pre_aPDL1_combo_tpm	SAM4caabd64e7fd	15.70431	0	NA	NA	1	0
Mariathasan_UC_pre_aPDL1_combo_tpm	SAMc6eff056c89a	1.806982	1	NA	NA	0	1
Mariathasan_UC_pre_aPDL1_combo_tpm	SAM5cfa1699bdb7	15.83573	0	NA	NA	0	1
Mariathasan_UC_pre_aPDL1_combo_tpm	SAMda4d892fddc8	14.12731	0	NA	NA	0	1
Mariathasan_UC_pre_aPDL1_combo_tpm	SAM3a1c9632ff7b	16.03285	0	NA	NA	0	0
Mariathasan_UC_pre_aPDL1_combo_tpm	SAM8b4b8b0f9e73	15.67146	0	NA	NA	0	0
Mariathasan_UC_pre_aPDL1_combo_tpm	SAMe3d4266775a9	13.99589	0	NA	NA	0	1
Mariathasan_UC_pre_aPDL1_combo_tpm	SAM2dc7cffb5f72	1.839836	1	NA	NA	0	0
Hugo_SKCM_pre_aPD1	SRR3184279	20.23333	1	NA	NA	0	1
Hugo_SKCM_pre_aPD1	SRR3184280	30.9	0	NA	NA	1	0
Hugo_SKCM_pre_aPD1	SRR3184281	31.6	0	NA	NA	1	0
Hugo_SKCM_pre_aPD1	SRR3184282	14.63333	0	NA	NA	1	0
Hugo_SKCM_pre_aPD1	SRR3184283	29.4	0	NA	NA	1	0
Hugo_SKCM_pre_aPD1	SRR3184284	22.06667	1	NA	NA	0	1
Hugo_SKCM_pre_aPD1	SRR3184285	0	0	NA	NA	1	0
Hugo_SKCM_pre_aPD1	SRR3184286	35.13333	0	NA	NA	1	0
Hugo_SKCM_pre_aPD1	SRR3184287	12.9	0	NA	NA	0	1
Hugo_SKCM_pre_aPD1	SRR3184288	10.9	1	NA	NA	0	1
Hugo_SKCM_pre_aPD1	SRR3184289	30.56667	0	NA	NA	1	0
Hugo_SKCM_pre_aPD1	SRR3184290	1.8	0	NA	NA	0	1
Hugo_SKCM_pre_aPD1	SRR3184291	32.66667	1	NA	NA	1	0
Hugo_SKCM_pre_aPD1	SRR3184293	35.33333	0	NA	NA	1	0
Hugo_SKCM_pre_aPD1	SRR3184294	11.23333	1	NA	NA	0	1
Hugo_SKCM_pre_aPD1	SRR3184295	6.066667	1	NA	NA	0	1
Hugo_SKCM_pre_aPD1	SRR3184296	3.433333	1	NA	NA	0	1
Hugo_SKCM_pre_aPD1	SRR3184297	8.733333	1	NA	NA	0	1
Hugo_SKCM_pre_aPD1	SRR3184298	18.26667	0	NA	NA	1	0
Hugo_SKCM_pre_aPD1	SRR3184300	14.63333	1	NA	NA	1	0
Hugo_SKCM_pre_aPD1	SRR3184301	8.966667	1	NA	NA	0	1
Hugo_SKCM_pre_aPD1	SRR3184302	23.46667	0	NA	NA	0	1
Hugo_SKCM_pre_aPD1	SRR3184303	5.7	1	NA	NA	0	1
Hugo_SKCM_pre_aPD1	SRR3184304	14.23333	0	NA	NA	1	0
Hugo_SKCM_pre_aPD1	SRR3184305	12.13333	0	NA	NA	1	0
Hugo_SKCM_pre_aPD1	SRR3184306	14.93333	0	NA	NA	1	0
Liu_SKCM_pre_aPD1_combo	Patient1	9.433333	1	3.1	1	0	1
Liu_SKCM_pre_aPD1_combo	Patient10	37.96667	1	5.633333	1	1	0
Liu_SKCM_pre_aPD1_combo	Patient100	31.26667	1	30.73333	1	1	0
Liu_SKCM_pre_aPD1_combo	Patient102	26.16667	0	5.866667	1	1	0
Liu_SKCM_pre_aPD1_combo	Patient105	17.36667	1	12.6	1	1	0
Liu_SKCM_pre_aPD1_combo	Patient106	26	0	26	0	1	0
Liu_SKCM_pre_aPD1_combo	Patient107	1.566667	1	1.566667	1	1	0
Liu_SKCM_pre_aPD1_combo	Patient108	5.5	1	2.566667	1	0	1

Liu_SKCM_pre_aPD1_combo	Patient11	5.333333	1	2.833333	1	0	1
Liu_SKCM_pre_aPD1_combo	Patient112	17.36667	1	3.033333	1	0	1
Liu_SKCM_pre_aPD1_combo	Patient116	3.933333	1	2.133333	1	0	1
Liu_SKCM_pre_aPD1_combo	Patient117	9.833333	1	3.333333	1	0	1
Liu_SKCM_pre_aPD1_combo	Patient121	43.53333	0	40.93333	1	1	0
Liu_SKCM_pre_aPD1_combo	Patient125	32.6	0	32.6	0	1	0
Liu_SKCM_pre_aPD1_combo	Patient126	33.06667	0	24.73333	1	1	0
Liu_SKCM_pre_aPD1_combo	Patient127	28.86667	0	28.86667	0	1	0
Liu_SKCM_pre_aPD1_combo	Patient13	1.933333	1	1.466667	1	0	1
Liu_SKCM_pre_aPD1_combo	Patient130	6.4	1	4.133333	1	0	1
Liu_SKCM_pre_aPD1_combo	Patient131	27.76667	0	27.76667	0	1	0
Liu_SKCM_pre_aPD1_combo	Patient132	24.63333	0	12.96667	1	1	0
Liu_SKCM_pre_aPD1_combo	Patient133	12.33333	1	2.1	1	0	1
Liu_SKCM_pre_aPD1_combo	Patient134	19.66667	1	2.1	1	0	1
Liu_SKCM_pre_aPD1_combo	Patient135	20.93333	0	20.93333	0	1	0
Liu_SKCM_pre_aPD1_combo	Patient137	25.73333	0	25.73333	0	1	0
Liu_SKCM_pre_aPD1_combo	Patient14	6.2	1	2.533333	1	0	1
Liu_SKCM_pre_aPD1_combo	Patient140	11.7	1	2.233333	1	0	1
Liu_SKCM_pre_aPD1_combo	Patient141	29.16667	0	29.16667	0	1	0
Liu_SKCM_pre_aPD1_combo	Patient142	33.2	0	33.2	0	0	0
Liu_SKCM_pre_aPD1_combo	Patient143	21	0	5.1	1	0	1
Liu_SKCM_pre_aPD1_combo	Patient144	22.06667	0	22.06667	0	1	0
Liu_SKCM_pre_aPD1_combo	Patient145	27.76667	0	27.76667	0	1	0
Liu_SKCM_pre_aPD1_combo	Patient146	20.4	0	20.4	0	1	0
Liu_SKCM_pre_aPD1_combo	Patient147	32	0	32	0	1	0
Liu_SKCM_pre_aPD1_combo	Patient148	26.4	0	26.4	0	1	0
Liu_SKCM_pre_aPD1_combo	Patient149	16.56667	1	7	1	0	0
Liu_SKCM_pre_aPD1_combo	Patient15	4.966667	1	3.5	1	1	0
Liu_SKCM_pre_aPD1_combo	Patient150	22.83333	0	22.83333	0	1	0
Liu_SKCM_pre_aPD1_combo	Patient154	21.16667	0	8.366667	1	0	0
Liu_SKCM_pre_aPD1_combo	Patient155	15.83333	0	3.766667	1	0	1
Liu_SKCM_pre_aPD1_combo	Patient156	2.9	1	2.666667	1	0	1
Liu_SKCM_pre_aPD1_combo	Patient158	6.466667	0	5.4	1	1	0
Liu_SKCM_pre_aPD1_combo	Patient159	9.6	0	7.2	1	0	0
Liu_SKCM_pre_aPD1_combo	Patient162	6.7	1	1.766667	1	0	1
Liu_SKCM_pre_aPD1_combo	Patient163	5.766667	1	1.6	1	0	1
Liu_SKCM_pre_aPD1_combo	Patient165	2.5	0	0.433333	1	0	1
Liu_SKCM_pre_aPD1_combo	Patient166	6.833333	1	6.833333	1	1	0
Liu_SKCM_pre_aPD1_combo	Patient167	9.4	1	1.9	1	0	1
Liu_SKCM_pre_aPD1_combo	Patient168	20.76667	0	19.33333	0	1	0
Liu_SKCM_pre_aPD1_combo	Patient169	18.1	0	18.1	0	1	0
Liu_SKCM_pre_aPD1_combo	Patient17	22.86667	1	6.733333	1	0	0
Liu_SKCM_pre_aPD1_combo	Patient170	11.56667	0	11.56667	0	0	0
Liu_SKCM_pre_aPD1_combo	Patient172	9.066667	0	9.066667	1	0	0

Liu_SKCM_pre_aPD1_combo	Patient173	1.4	1	0.533333	1	0	1
Liu_SKCM_pre_aPD1_combo	Patient179	21.76667	0	11.2	1	1	0
Liu_SKCM_pre_aPD1_combo	Patient18	4.333333	1	2.866667	1	0	1
Liu_SKCM_pre_aPD1_combo	Patient181	15.86667	0	15.86667	0	1	0
Liu_SKCM_pre_aPD1_combo	Patient183	19.06667	1	2.8	1	0	1
Liu_SKCM_pre_aPD1_combo	Patient184	17.1	0	12.4	0	1	0
Liu_SKCM_pre_aPD1_combo	Patient185	6.766667	0	6.766667	0	0	0
Liu_SKCM_pre_aPD1_combo	Patient187	20.56667	0	20.56667	0	1	0
Liu_SKCM_pre_aPD1_combo	Patient188	2.466667	1	0.766667	1	0	1
Liu_SKCM_pre_aPD1_combo	Patient189	23.93333	0	13.53333	1	1	0
Liu_SKCM_pre_aPD1_combo	Patient191	21.93333	0	21.93333	0	1	0
Liu_SKCM_pre_aPD1_combo	Patient193	21.96667	0	15.86667	1	0	0
Liu_SKCM_pre_aPD1_combo	Patient195	29.83333	0	29.83333	0	1	0
Liu_SKCM_pre_aPD1_combo	Patient196	2.7	1	2.7	1	0	1
Liu_SKCM_pre_aPD1_combo	Patient197	32	0	2.966667	1	1	0
Liu_SKCM_pre_aPD1_combo	Patient20	8.066667	1	2.8	1	0	1
Liu_SKCM_pre_aPD1_combo	Patient200	17.06667	1	3.6	1	0	1
Liu_SKCM_pre_aPD1_combo	Patient201	5.566667	1	2.3	1	0	1
Liu_SKCM_pre_aPD1_combo	Patient203	20.86667	1	3.3	1	0	1
Liu_SKCM_pre_aPD1_combo	Patient204	27.3	0	21	0	1	0
Liu_SKCM_pre_aPD1_combo	Patient205	13.06667	1	6.333333	1	0	1
Liu_SKCM_pre_aPD1_combo	Patient206	3	1	1.4	1	0	1
Liu_SKCM_pre_aPD1_combo	Patient21	30.2	0	30.2	0	1	0
Liu_SKCM_pre_aPD1_combo	Patient22	8.433333	1	3.033333	1	0	1
Liu_SKCM_pre_aPD1_combo	Patient23	32.5	0	2.566667	1	0	1
Liu_SKCM_pre_aPD1_combo	Patient24	4.566667	1	1.033333	1	0	1
Liu_SKCM_pre_aPD1_combo	Patient25	36.53333	0	25.06667	1	1	0
Liu_SKCM_pre_aPD1_combo	Patient27	14.36667	1	3.133333	1	0	1
Liu_SKCM_pre_aPD1_combo	Patient30	7.1	1	4.433333	1	0	0
Liu_SKCM_pre_aPD1_combo	Patient31	14.2	1	2.833333	1	0	1
Liu_SKCM_pre_aPD1_combo	Patient32	1.766667	1	1.633333	1	0	1
Liu_SKCM_pre_aPD1_combo	Patient33	55.96667	0	55.96667	0	1	0
Liu_SKCM_pre_aPD1_combo	Patient34	56.36667	0	8	1	1	0
Liu_SKCM_pre_aPD1_combo	Patient35	32.26667	1	6.033333	1	0	0
Liu_SKCM_pre_aPD1_combo	Patient36	15.03333	1	2.1	1	0	1
Liu_SKCM_pre_aPD1_combo	Patient37	9.166667	1	2.566667	1	0	1
Liu_SKCM_pre_aPD1_combo	Patient38	11.43333	1	3.266667	1	0	1
Liu_SKCM_pre_aPD1_combo	Patient4	20	1	19.13333	1	1	0
Liu_SKCM_pre_aPD1_combo	Patient40	15.6	1	2.1	1	0	1
Liu_SKCM_pre_aPD1_combo	Patient42	34.23333	0	34.23333	0	1	0
Liu_SKCM_pre_aPD1_combo	Patient44	30.73333	0	30.73333	0	1	0
Liu_SKCM_pre_aPD1_combo	Patient45	55.53333	0	55.53333	0	1	0
Liu_SKCM_pre_aPD1_combo	Patient47	31.23333	0	2.466667	1	0	1
Liu_SKCM_pre_aPD1_combo	Patient48	1.766667	1	1.666667	1	0	1

Liu_SKCM_pre_aPD1_combo	Patient51	51.56667	0	15.7	1	1	0
Liu_SKCM_pre_aPD1_combo	Patient56	4.066667	1	2.366667	1	0	1
Liu_SKCM_pre_aPD1_combo	Patient58	10.1	1	2.566667	1	0	1
Liu_SKCM_pre_aPD1_combo	Patient6	22.9	1	4.133333	1	0	0
Liu_SKCM_pre_aPD1_combo	Patient61	32.9	0	32.9	0	0	0
Liu_SKCM_pre_aPD1_combo	Patient62	33.36667	0	33.36667	0	1	0
Liu_SKCM_pre_aPD1_combo	Patient63	5.933333	1	2.966667	1	0	1
Liu_SKCM_pre_aPD1_combo	Patient67	4.533333	1	3.133333	1	0	1
Liu_SKCM_pre_aPD1_combo	Patient7	8.966667	1	7.233333	1	0	0
Liu_SKCM_pre_aPD1_combo	Patient72	13.16667	0	2.233333	1	0	1
Liu_SKCM_pre_aPD1_combo	Patient73	28.2	1	2.833333	1	0	1
Liu_SKCM_pre_aPD1_combo	Patient75	29.7	0	29.7	1	1	0
Liu_SKCM_pre_aPD1_combo	Patient77	14.8	1	3.166667	1	0	1
Liu_SKCM_pre_aPD1_combo	Patient78	11.86667	1	2.866667	1	0	1
Liu_SKCM_pre_aPD1_combo	Patient79	28.3	0	2.8	1	0	1
Liu_SKCM_pre_aPD1_combo	Patient8	33.5	1	31.83333	1	0	0
Liu_SKCM_pre_aPD1_combo	Patient82	4.366667	1	2.966667	1	0	1
Liu_SKCM_pre_aPD1_combo	Patient83	2.366667	1	2.366667	1	0	1
Liu_SKCM_pre_aPD1_combo	Patient86	44.23333	0	44.23333	1	0	0
Liu_SKCM_pre_aPD1_combo	Patient87	42.1	0	38.06667	1	1	0
Liu_SKCM_pre_aPD1_combo	Patient9	32.1	0	0.7	1	0	1
Liu_SKCM_pre_aPD1_combo	Patient94	1.333333	1	1.333333	1	0	1
Liu_SKCM_pre_aPD1_combo	Patient96	30.43333	0	30.43333	0	1	0
Liu_SKCM_pre_aPD1_combo	Patient98	22.76667	1	2.433333	1	0	1
Liu_SKCM_pre_aPD1_combo	Patient99	30.7	0	10.63333	1	1	0
Gide_SKCM_pre_combo	ERR2208941	2.166667	1	0.433333	1	0	1
Gide_SKCM_pre_combo	ERR2208949	1.966667	1	0.766667	1	0	1
Gide_SKCM_pre_combo	ERR3262564	0.733333	1	0.766667	1	0	1
Gide_SKCM_pre_combo	ERR2208968	5.166667	1	1.3	1	0	1
Gide_SKCM_pre_combo	ERR2208973	3.133333	1	1.4	1	0	1
Gide_SKCM_pre_combo	ERR2208974	2.666667	1	1.566667	1	0	1
Gide_SKCM_pre_combo	ERR3262565	1.933333	1	1.933333	1	0	1
Gide_SKCM_pre_combo	ERR2208977	3.3	1	2.366667	1	0	1
Gide_SKCM_pre_combo	ERR2208929	18.36667	0	2.533333	1	0	1
Gide_SKCM_pre_combo	ERR2208930	5.533333	1	2.6	1	0	1
Gide_SKCM_pre_combo	ERR2208931	6.633333	1	2.666667	1	0	0
Gide_SKCM_pre_combo	ERR2208933	3.2	1	2.733333	1	0	1
Gide_SKCM_pre_combo	ERR2208934	33.1	1	2.766667	1	0	1
Gide_SKCM_pre_combo	ERR2208935	10	1	2.8	1	0	1
Gide_SKCM_pre_combo	ERR2208936	5.633333	1	2.8	1	0	1
Gide_SKCM_pre_combo	ERR2208938	8.6	1	3.2	1	0	1
Gide_SKCM_pre_combo	ERR2208939	7.6	1	3.333333	1	0	1
Gide_SKCM_pre_combo	ERR2208940	12.83333	1	4.166667	1	0	0
Gide_SKCM_pre_combo	ERR2208942	18.36667	1	5.533333	1	0	0

Gide_SKCM_pre_combo	ERR2208943	6.366667	1	6.366667	1	0	0
Gide_SKCM_pre_combo	ERR2208945	29.9	1	9.033333	1	1	0
Gide_SKCM_pre_combo	ERR2208946	35.56667	1	10.46667	1	1	0
Gide_SKCM_pre_combo	ERR2208948	27.5	1	11.16667	1	0	0
Gide_SKCM_pre_combo	ERR3262563	19.36667	0	11.93333	1	0	0
Gide_SKCM_pre_combo	ERR2208951	29.26667	1	18.13333	1	1	0
Gide_SKCM_pre_combo	ERR2208952	36.36667	0	19.53333	1	1	0
Gide_SKCM_pre_combo	ERR2208953	20.23333	1	19.66667	1	1	0
Gide_SKCM_pre_combo	ERR2208954	51.56667	0	25.16667	1	1	0
Gide_SKCM_pre_combo	ERR2208956	28.16667	0	28.16667	0	1	0
Gide_SKCM_pre_combo	ERR2208958	29.53333	0	29	1	1	0
Gide_SKCM_pre_combo	ERR2208959	29.7	0	29.7	0	1	0
Gide_SKCM_pre_combo	ERR2208960	35.83333	0	35.83333	0	1	0
Gide_SKCM_pre_combo	ERR2208961	36.16667	0	36.16667	0	1	0
Gide_SKCM_pre_combo	ERR2208962	36.6	0	36.6	0	1	0
Gide_SKCM_pre_combo	ERR2208964	39.13333	0	39.13333	0	1	0
Gide_SKCM_pre_combo	ERR2208966	39.63333	0	39.63333	0	1	0
Gide_SKCM_pre_combo	ERR2208967	44.26667	0	44.26667	0	1	0
Gide_SKCM_pre_combo	ERR2208969	44.73333	0	44.73333	0	1	0
Gide_SKCM_pre_combo	ERR2208970	51.76667	0	51.76667	0	1	0
Gide_SKCM_pre_combo	ERR2208971	52.96667	0	52.96667	0	1	0
Gide_SKCM_pre_combo	ERR2208972	53.43333	0	53.43333	0	1	0
Gide_SKCM_pre_combo	ERR2208888	22.96667	0	0.5	1	0	1
Gide_SKCM_pre_combo	ERR2208898	17.66667	0	1.133333	1	0	1
Gide_SKCM_pre_combo	ERR2208924	3.6	1	1.366667	1	0	1
Gide_SKCM_pre_combo	ERR3262562	5.633333	0	2.7	1	1	0
Gide_SKCM_pre_combo	ERR2208926	21.23333	0	2.7	1	0	1
Gide_SKCM_pre_combo	ERR2208889	5.866667	1	2.7	1	0	1
Gide_SKCM_pre_combo	ERR2208890	9.766667	0	2.733333	1	0	1
Gide_SKCM_pre_combo	ERR2208892	11.9	1	3.6	1	1	0
Gide_SKCM_pre_combo	ERR2208893	23.03333	0	4.1	1	0	0
Gide_SKCM_pre_combo	ERR2208894	22	1	5.066667	1	0	0
Gide_SKCM_pre_combo	ERR2208895	6.533333	0	6.533333	0	1	0
Gide_SKCM_pre_combo	ERR2208897	21.16667	0	10.03333	1	0	0
Gide_SKCM_pre_combo	ERR2208899	17.93333	0	12.86667	1	0	0
Gide_SKCM_pre_combo	ERR2208900	13.66667	0	13.66667	0	1	0
Gide_SKCM_pre_combo	ERR2208901	16.86667	0	16.86667	0	1	0
Gide_SKCM_pre_combo	ERR2208902	40.1	0	16.96667	1	1	0
Gide_SKCM_pre_combo	ERR2208903	17.26667	1	17.26667	1	0	0
Gide_SKCM_pre_combo	ERR2208904	18.13333	0	18.13333	0	1	0
Gide_SKCM_pre_combo	ERR2208906	18.2	0	18.2	0	1	0
Gide_SKCM_pre_combo	ERR2208907	19.9	0	19.9	0	1	0
Gide_SKCM_pre_combo	ERR2208909	20.3	0	20.3	0	1	0
Gide_SKCM_pre_combo	ERR2208910	20.76667	0	20.76667	0	1	0

Gide_SKCM_pre_combo	ERR2208912	20.9	0	20.9	0	1	0
Gide_SKCM_pre_combo	ERR2208914	21.23333	0	21.23333	0	1	0
Gide_SKCM_pre_combo	ERR2208915	21.23333	0	21.23333	0	1	0
Gide_SKCM_pre_combo	ERR2208916	21.66667	0	21.66667	0	1	0
Gide_SKCM_pre_combo	ERR2208918	23.16667	0	23.16667	0	1	0
Gide_SKCM_pre_combo	ERR2208919	24.03333	0	24.03333	0	1	0
Gide_SKCM_pre_combo	ERR2208920	24.03333	0	24.03333	0	1	0
Gide_SKCM_pre_combo	ERR2208921	24.4	0	24.4	0	1	0
Gide_SKCM_pre_combo	ERR2208922	25.83333	0	25.83333	0	1	0
Gide_SKCM_pre_combo	ERR2208923	30.13333	0	30.13333	0	1	0
Riaz_SKCM_pre_aPD1_combo	Pt1_Pre_AD101148-6	5.333333	1	NA	NA	0	1
Riaz_SKCM_pre_aPD1_combo	Pt10_Pre_E9047565-6	8.533333	1	NA	NA	0	0
Riaz_SKCM_pre_aPD1_combo	Pt101_Pre_AD486328-5	27.8	0	NA	NA	1	0
Riaz_SKCM_pre_aPD1_combo	Pt103_Pre_AE134058-2	16.13333	0	NA	NA	0	1
Riaz_SKCM_pre_aPD1_combo	Pt106_Pre_AD502250-5	3.033333	1	NA	NA	0	1
Riaz_SKCM_pre_aPD1_combo	Pt11_Pre_AD153352-6	27.9	1	NA	NA	0	1
Riaz_SKCM_pre_aPD1_combo	Pt17_Pre_E9047563-6	1.9	1	NA	NA	0	1
Riaz_SKCM_pre_aPD1_combo	Pt18_Pre_E9024732-6	35.76667	0	NA	NA	1	0
Riaz_SKCM_pre_aPD1_combo	Pt2_Pre_AD101150-6	9.166667	1	NA	NA	0	0
Riaz_SKCM_pre_aPD1_combo	Pt24_Pre_AD436687-5	4.966667	0	NA	NA	0	1
Riaz_SKCM_pre_aPD1_combo	Pt26_Pre_AD467789-6	31.73333	0	NA	NA	0	0
Riaz_SKCM_pre_aPD1_combo	Pt27_Pre_AD453873-5	15.83333	1	NA	NA	0	1
Riaz_SKCM_pre_aPD1_combo	Pt28_Pre_AD297619-6	24.66667	1	NA	NA	0	1
Riaz_SKCM_pre_aPD1_combo	Pt29_Pre_AD497504-5	9.1	1	NA	NA	0	1
Riaz_SKCM_pre_aPD1_combo	Pt3_Pre_E9024733-3	38.13333	0	NA	NA	1	0
Riaz_SKCM_pre_aPD1_combo	Pt30_Pre_AD497503-5	35.1	0	NA	NA	1	0
Riaz_SKCM_pre_aPD1_combo	Pt31_Pre_AD453872-5	32.03333	0	NA	NA	0	1
Riaz_SKCM_pre_aPD1_combo	Pt34_Pre_AD466985-6	27.8	0	NA	NA	1	0
Riaz_SKCM_pre_aPD1_combo	Pt36_Pre_AD467095-6	36.03333	0	NA	NA	0	0
Riaz_SKCM_pre_aPD1_combo	Pt37_Pre_AD502452-5	21.53333	1	NA	NA	0	0
Riaz_SKCM_pre_aPD1_combo	Pt38_Pre_E9200719-6	5.566667	1	NA	NA	0	0
Riaz_SKCM_pre_aPD1_combo	Pt39_Pre_AD485899-5	5.533333	1	NA	NA	0	1
Riaz_SKCM_pre_aPD1_combo	Pt4_Pre_E9021023-6	21.1	1	NA	NA	0	0
Riaz_SKCM_pre_aPD1_combo	Pt44_Pre_AD467790-6	36.43333	0	NA	NA	1	0
Riaz_SKCM_pre_aPD1_combo	Pt46_Pre_AD467096-6	7.566667	1	NA	NA	0	1
Riaz_SKCM_pre_aPD1_combo	Pt47_Pre_AD506073-6	23.93333	1	NA	NA	0	1
Riaz_SKCM_pre_aPD1_combo	Pt48_Pre_E9047561-7	34.86667	0	NA	NA	1	0
Riaz_SKCM_pre_aPD1_combo	Pt49_Pre_AD667851-6	27.56667	0	NA	NA	1	0
Riaz_SKCM_pre_aPD1_combo	Pt5_Pre_E9021022-6	7.4	1	NA	NA	0	1
Riaz_SKCM_pre_aPD1_combo	Pt52_Pre_AD506075-6	15.86667	1	NA	NA	0	1
Riaz_SKCM_pre_aPD1_combo	Pt59_Pre_AD823915-5	23.6	1	NA	NA	0	0
Riaz_SKCM_pre_aPD1_combo	Pt62_Pre_AD608303-5	15.73333	1	NA	NA	0	1
Riaz_SKCM_pre_aPD1_combo	Pt65_Pre_AD793919-6	23.06667	0	NA	NA	0	0
Riaz_SKCM_pre_aPD1_combo	Pt66_Pre_AD667850-6	18.06667	1	NA	NA	0	1

Riaz_SKCM_pre_aPD1_combo	Pt67_Pre_AD506074-6	34.4	0	NA	NA	0	0
Riaz_SKCM_pre_aPD1_combo	Pt72_Pre_AD793922-5	25.66667	1	NA	NA	1	0
Riaz_SKCM_pre_aPD1_combo	Pt77_Pre_AD733591-7	15.7	1	NA	NA	0	0
Riaz_SKCM_pre_aPD1_combo	Pt78_Pre_AD467018-5	2.6	1	NA	NA	0	1
Riaz_SKCM_pre_aPD1_combo	Pt79_Pre_AD733587-5	7.266667	1	NA	NA	0	0
Riaz_SKCM_pre_aPD1_combo	Pt8_Pre_AD153354-6	8.633333	1	NA	NA	0	1
Riaz_SKCM_pre_aPD1_combo	Pt82_Pre_AD823914-8	14.3	1	NA	NA	0	0
Riaz_SKCM_pre_aPD1_combo	Pt84_Pre_AD486532-5	5.033333	1	NA	NA	0	1
Riaz_SKCM_pre_aPD1_combo	Pt85_Pre_AD486329-5	30.13333	0	NA	NA	0	1
Riaz_SKCM_pre_aPD1_combo	Pt89_Pre_AE070951-5	28.13333	1	NA	NA	0	0
Riaz_SKCM_pre_aPD1_combo	Pt9_Pre_E9021024-6	3.066667	1	NA	NA	0	1
Riaz_SKCM_pre_aPD1_combo	Pt90_Pre_AD467873-6	5.8	1	NA	NA	0	1
Riaz_SKCM_pre_aPD1_combo	Pt92_Pre_AE134060-5	11.1	1	NA	NA	0	0
Riaz_SKCM_pre_aPD1_combo	Pt94_Pre_AD732850-6	32.7	0	NA	NA	1	0
Riaz_SKCM_pre_aPD1_combo	Pt98_Pre_AD733586-8	24.9	1	NA	NA	0	0
Van_SKCM_pre_aPD1	Pat02	54.4	0	17.93333	1	1	0
Van_SKCM_pre_aPD1	Pat04	32.9	0	21.53333	0	1	0
Van_SKCM_pre_aPD1	Pat123	28.43333	1	18.13333	1	1	0
Van_SKCM_pre_aPD1	Pat126	21.36667	0	6.233333	1	1	0
Van_SKCM_pre_aPD1	Pat29	44.2	0	11.36667	1	1	NA
Van_SKCM_pre_aPD1	Pat38	51.3	0	10.06667	1	1	0
Van_SKCM_pre_aPD1	Pat39	49.56667	0	49.56667	0	1	0
Van_SKCM_pre_aPD1	Pat47	36.86667	0	36.86667	0	1	0
Van_SKCM_pre_aPD1	Pat49	34.46667	0	5.433333	1	1	0
Van_SKCM_pre_aPD1	Pat79	26.7	1	13.5	1	1	0
Van_SKCM_pre_aPD1	Pat80	24.13333	1	6.066667	1	1	0
Van_SKCM_pre_aPD1	Pat88	32.96667	0	22.16667	0	1	0
Van_SKCM_pre_aPD1	Pat90	33.5	0	22.3	0	1	0
Van_SKCM_pre_aPD1	Pat91	22.66667	1	22.66667	0	1	0
Van_SKCM_pre_aPD1	Pat03	3.333333	1	2.533333	1	0	1
Van_SKCM_pre_aPD1	Pat06	5.366667	1	2.566667	1	0	1
Van_SKCM_pre_aPD1	Pat08	4.666667	1	2.433333	1	0	1
Van_SKCM_pre_aPD1	Pat118	10.43333	1	4	1	0	1
Van_SKCM_pre_aPD1	Pat14	5.433333	1	2.8	1	0	1
Van_SKCM_pre_aPD1	Pat15	1.666667	1	1.033333	1	0	1
Van_SKCM_pre_aPD1	Pat19	5.833333	1	0.7	1	0	1
Van_SKCM_pre_aPD1	Pat20	3.866667	1	2.366667	1	0	1
Van_SKCM_pre_aPD1	Pat25	10.83333	1	2.233333	1	0	1
Van_SKCM_pre_aPD1	Pat33	7.033333	1	7.033333	1	0	1
Van_SKCM_pre_aPD1	Pat36	1.8	1	1.366667	1	0	1
Van_SKCM_pre_aPD1	Pat37	2.333333	1	0.766667	1	0	1
Van_SKCM_pre_aPD1	Pat40	1.133333	1	1.133333	1	0	1
Van_SKCM_pre_aPD1	Pat43	1.233333	1	1.233333	1	0	1
Van_SKCM_pre_aPD1	Pat44	9.033333	1	2.066667	1	0	1

Van_SKCM_pre_aPD1	Pat45	2.966667	1	2.033333	1	0	1
Van_SKCM_pre_aPD1	Pat46	5.333333	1	1.2	1	0	1
Van_SKCM_pre_aPD1	Pat50	2.166667	1	2.166667	1	0	1
Van_SKCM_pre_aPD1	Pat81	20.93333	1	2.533333	1	0	1
Van_SKCM_pre_aPD1	Pat85	15.26667	1	2.766667	1	0	1
Van_SKCM_pre_aPD1	Pat86	9.766667	1	5.1	1	0	0
Van_SKCM_pre_aPD1	Pat98	4.666667	1	2.566667	1	0	1
Kim_GC_pre_aPD1	PB.16.002	NA	NA	NA	NA	0	1
Kim_GC_pre_aPD1	PB.16.003	NA	NA	NA	NA	0	0
Kim_GC_pre_aPD1	PB.16.004	NA	NA	NA	NA	0	1
Kim_GC_pre_aPD1	PB.16.005	NA	NA	NA	NA	1	0
Kim_GC_pre_aPD1	PB.16.018	NA	NA	NA	NA	0	1
Kim_GC_pre_aPD1	PB.16.019	NA	NA	NA	NA	1	0
Kim_GC_pre_aPD1	PB.16.020	NA	NA	NA	NA	1	0
Kim_GC_pre_aPD1	PB.16.021	NA	NA	NA	NA	1	0
Kim_GC_pre_aPD1	PB.16.022	NA	NA	NA	NA	0	0
Kim_GC_pre_aPD1	PB.16.024	NA	NA	NA	NA	0	1
Kim_GC_pre_aPD1	PB.16.025	NA	NA	NA	NA	0	1
Kim_GC_pre_aPD1	PB.16.029	NA	NA	NA	NA	0	1
Kim_GC_pre_aPD1	PB.16.030	NA	NA	NA	NA	0	1
Kim_GC_pre_aPD1	PB.16.023	NA	NA	NA	NA	0	1
Kim_GC_pre_aPD1	PB.16.032	NA	NA	NA	NA	0	1
Kim_GC_pre_aPD1	PB.16.034	NA	NA	NA	NA	0	0
Kim_GC_pre_aPD1	PB.16.035	NA	NA	NA	NA	0	1
Kim_GC_pre_aPD1	PB.16.037	NA	NA	NA	NA	0	0
Kim_GC_pre_aPD1	PB.16.038	NA	NA	NA	NA	1	0
Kim_GC_pre_aPD1	PB.16.039	NA	NA	NA	NA	0	1
Kim_GC_pre_aPD1	PB.16.040	NA	NA	NA	NA	0	0
Kim_GC_pre_aPD1	PB.16.041	NA	NA	NA	NA	0	1
Kim_GC_pre_aPD1	PB.16.042	NA	NA	NA	NA	0	0
Kim_GC_pre_aPD1	PB.16.043	NA	NA	NA	NA	0	1
Kim_GC_pre_aPD1	PB.16.044	NA	NA	NA	NA	1	0
Kim_GC_pre_aPD1	PB.16.045	NA	NA	NA	NA	1	0
Kim_GC_pre_aPD1	PB.16.047	NA	NA	NA	NA	0	0
Kim_GC_pre_aPD1	PB.16.048	NA	NA	NA	NA	1	0
Kim_GC_pre_aPD1	PB.16.049	NA	NA	NA	NA	0	0
Kim_GC_pre_aPD1	PB.16.051	NA	NA	NA	NA	0	1
Kim_GC_pre_aPD1	PB.16.055	NA	NA	NA	NA	0	1
Kim_GC_pre_aPD1	PB.16.052	NA	NA	NA	NA	0	0
Kim_GC_pre_aPD1	PB.16.056	NA	NA	NA	NA	0	0
Kim_GC_pre_aPD1	PB.16.057	NA	NA	NA	NA	0	1
Kim_GC_pre_aPD1	PB.16.054	NA	NA	NA	NA	0	0
Kim_GC_pre_aPD1	PB.16.060	NA	NA	NA	NA	0	1
Kim_GC_pre_aPD1	PB.16.059	NA	NA	NA	NA	1	0

Kim_GC_pre_aPD1	PB.16.064	NA	NA	NA	NA	0	0
Kim_GC_pre_aPD1	PB.16.062	NA	NA	NA	NA	0	0
Kim_GC_pre_aPD1	PB.16.063	NA	NA	NA	NA	1	0
Kim_GC_pre_aPD1	PB.16.066	NA	NA	NA	NA	0	0
Kim_GC_pre_aPD1	PB.16.067	NA	NA	NA	NA	0	1
Kim_GC_pre_aPD1	PB.16.068	NA	NA	NA	NA	1	0
Kim_GC_pre_aPD1	PB.16.069	NA	NA	NA	NA	0	0
Kim_GC_pre_aPD1	PB.16.031	NA	NA	NA	NA	1	0
Zhao_GBM_pre_aPD1	SRR8281239	14.6	0	NA	NA	1	NA
Zhao_GBM_pre_aPD1	SRR8281240	47.13333	0	NA	NA	1	NA
Zhao_GBM_pre_aPD1	SRR8281218	34.43333	1	NA	NA	0	NA
Zhao_GBM_pre_aPD1	SRR8281248	23.46667	0	NA	NA	1	NA
Zhao_GBM_pre_aPD1	SRR8281230	19.83333	1	NA	NA	0	NA
Zhao_GBM_pre_aPD1	SRR8281226	31.7	1	NA	NA	0	NA
Zhao_GBM_pre_aPD1	SRR8281224	56.4	1	NA	NA	0	NA
Zhao_GBM_pre_aPD1	SRR8281242	46.2	1	NA	NA	1	NA
Zhao_GBM_pre_aPD1	SRR8281232	15.5	1	NA	NA	0	NA
Zhao_GBM_pre_aPD1	SRR8281237	46	0	NA	NA	0	NA
Zhao_GBM_pre_aPD1	SRR8281246	16.16667	1	NA	NA	1	NA
Zhao_GBM_pre_aPD1	SRR8281238	24.3	0	NA	NA	1	NA
Zhao_GBM_pre_aPD1	SRR8281231	25.8	0	NA	NA	1	NA
Zhao_GBM_pre_aPD1	SRR8281223	10.4	1	NA	NA	0	NA
Zhao_GBM_pre_aPD1	SRR8281229	37.06667	0	NA	NA	1	NA
Zhao_GBM_pre_aPD1	SRR8281249	54.2	1	NA	NA	1	NA
Zhao_GBM_pre_aPD1	SRR8281245	47.5	0	NA	NA	1	NA
Snyder_UC_pre_aPDL1	40	0.8	1	0.666667	1	0	NA
Snyder_UC_pre_aPDL1	471	10.96667	1	2.033333	1	0	NA
Snyder_UC_pre_aPDL1	522	2.533333	1	1.933333	1	0	NA
Snyder_UC_pre_aPDL1	1233	24.13333	0	13.26667	1	0	NA
Snyder_UC_pre_aPDL1	1249	1.8	1	1.366667	1	0	NA
Snyder_UC_pre_aPDL1	1849	15.6	1	8.833333	1	0	NA
Snyder_UC_pre_aPDL1	1994	13.43333	1	4.033333	1	0	NA
Snyder_UC_pre_aPDL1	2131	21	0	18.66667	0	1	NA
Snyder_UC_pre_aPDL1	2278	23.4	0	21.83333	0	1	NA
Snyder_UC_pre_aPDL1	2389	21.7	0	21.53333	0	1	NA
Snyder_UC_pre_aPDL1	2849	19.53333	1	2.5	1	0	NA
Snyder_UC_pre_aPDL1	2937	1.466667	1	1.233333	1	0	NA
Snyder_UC_pre_aPDL1	3529	6.066667	1	2.133333	1	0	NA
Snyder_UC_pre_aPDL1	5037	20.33333	0	18.7	0	1	NA
Snyder_UC_pre_aPDL1	5122	23.33333	0	18.83333	0	1	NA
Snyder_UC_pre_aPDL1	5338	8.866667	1	2.233333	1	0	NA
Snyder_UC_pre_aPDL1	6229	21.73333	0	8.4	1	0	NA
Snyder_UC_pre_aPDL1	6428	5.433333	1	2.033333	1	0	NA
Snyder_UC_pre_aPDL1	6800	21.7	0	21.66667	0	1	NA

Snyder_UC_pre_aPDL1	7577	3.9	1	2	1	0	NA
Snyder_UC_pre_aPDL1	7729	11.8	1	2	1	0	NA
Snyder_UC_pre_aPDL1	8728	18.2	1	2.033333	1	0	NA
Snyder_UC_pre_aPDL1	9517	11.03333	1	4.033333	1	0	NA
Snyder_UC_pre_aPDL1	9723	0.733333	1	0.633333	1	0	NA
Snyder_UC_pre_aPDL1	9881	10.6	1	2.8	1	0	NA
Aleix_SKCM_pre_aPD1	GSM2445716	1.803279	1	NA	NA	0	NA
Aleix_SKCM_pre_aPD1	GSM2445717	1.114754	1	NA	NA	0	NA
Aleix_SKCM_pre_aPD1	GSM2445718	2.491803	1	NA	NA	0	NA
Aleix_SKCM_pre_aPD1	GSM2445719	3.901639	1	NA	NA	0	NA
Aleix_SKCM_pre_aPD1	GSM2445720	2.52459	1	NA	NA	0	NA
Aleix_SKCM_pre_aPD1	GSM2445721	3.114754	1	NA	NA	0	NA
Aleix_SKCM_pre_aPD1	GSM2445722	2.52459	1	NA	NA	0	NA
Aleix_SKCM_pre_aPD1	GSM2445723	2.065574	1	NA	NA	0	NA
Aleix_SKCM_pre_aPD1	GSM2445724	2.586885	1	NA	NA	0	NA
Aleix_SKCM_pre_aPD1	GSM2445725	1.442623	1	NA	NA	0	NA
Aleix_SKCM_pre_aPD1	GSM2445726	6.196721	0	NA	NA	0	NA
Aleix_SKCM_pre_aPD1	GSM2445727	20.62295	0	NA	NA	1	NA
Aleix_SKCM_pre_aPD1	GSM2445728	7.180328	1	NA	NA	1	NA
Aleix_SKCM_pre_aPD1	GSM2445729	16.13115	0	NA	NA	1	NA
Aleix_SKCM_pre_aPD1	GSM2445730	6.393443	1	NA	NA	1	NA
Aleix_SKCM_pre_aPD1	GSM2445731	8.229508	0	NA	NA	1	NA
Aleix_SKCM_pre_aPD1	GSM2445732	2.52459	1	NA	NA	1	NA
Aleix_SKCM_pre_aPD1	GSM2445733	31.67213	0	NA	NA	1	NA
Aleix_SKCM_pre_aPD1	GSM2445734	2.557377	1	NA	NA	0	NA
Aleix_SKCM_pre_aPD1	GSM2445735	2.360656	1	NA	NA	0	NA
Aleix_SKCM_pre_aPD1	GSM2445736	1.672131	1	NA	NA	0	NA
Aleix_SKCM_pre_aPD1	GSM2445737	22.06557	0	NA	NA	1	NA
Aleix_SKCM_pre_aPD1	GSM2445738	4.360656	1	NA	NA	0	NA
Aleix_SKCM_pre_aPD1	GSM2445739	3.114754	1	NA	NA	0	NA
Aleix_SKCM_pre_aPD1	GSM2445740	22.03279	0	NA	NA	1	NA
Nathanson_SKCM_pre_aCTLA4	6126	22.7377	0	NA	NA	1	NA
Nathanson_SKCM_pre_aCTLA4	167	32.31148	0	NA	NA	1	NA
Nathanson_SKCM_pre_aCTLA4	1494	23.93443	0	NA	NA	1	NA
Nathanson_SKCM_pre_aCTLA4	2056	46.67213	0	NA	NA	1	NA
Nathanson_SKCM_pre_aCTLA4	2051	9.57377	1	NA	NA	0	NA
Nathanson_SKCM_pre_aCTLA4	5784	5.983607	1	NA	NA	0	NA
Nathanson_SKCM_pre_aCTLA4	8727	4.786885	1	NA	NA	0	NA
Nathanson_SKCM_pre_aCTLA4	1867	17.95082	1	NA	NA	0	NA
Nathanson_SKCM_pre_aCTLA4	3549	5.983607	1	NA	NA	0	NA
Auslander_SKCM_pre_aPD1_aCTLA4_Com	SRR7344544	NA		NA	NA	0	NA
Auslander_SKCM_pre_aPD1_aCTLA4_Com	SRR7344546	NA		NA	NA	1	NA
Auslander_SKCM_pre_aPD1_aCTLA4_Com	SRR7344550	NA		NA	NA	0	NA
Auslander_SKCM_pre_aPD1_aCTLA4_Com	SRR7344552	NA		NA	NA	0	NA

Auslander_SKCM_pre_aPD1_aCTLA4_Com	SRR7344554	NA		NA	NA	0	NA
Auslander_SKCM_pre_aPD1_aCTLA4_Com	SRR7344556	NA		NA	NA	0	NA
Auslander_SKCM_pre_aPD1_aCTLA4_Com	SRR7344559	NA		NA	NA	1	NA
Auslander_SKCM_pre_aPD1_aCTLA4_Com	SRR7344561	NA		NA	NA	0	NA
Auslander_SKCM_pre_aPD1_aCTLA4_Com	SRR7344564	NA		NA	NA	0	NA
Auslander_SKCM_pre_aPD1_aCTLA4_Com	SRR7344565	NA		NA	NA	0	NA
Auslander_SKCM_pre_aPD1_aCTLA4_Com	SRR7344567	NA		NA	NA	0	NA
Auslander_SKCM_pre_aPD1_aCTLA4_Com	SRR7344574	NA		NA	NA	0	NA
Auslander_SKCM_pre_aPD1_aCTLA4_Com	SRR7344575	NA		NA	NA	0	NA
Prat_HNSC_pre_aPD1	GSM2445676	2.688525	1	NA	NA	0	NA
Prat_HNSC_pre_aPD1	GSM2445677	1.967213	1	NA	NA	0	NA
Prat_HNSC_pre_aPD1	GSM2445678	1.770492	0	NA	NA	0	NA
Prat_HNSC_pre_aPD1	GSM2445679	13.34426	0	NA	NA	1	NA
Prat_HNSC_pre_aPD1	GSM2445680	11.96721	0	NA	NA	1	NA
Prat_LUSC_pre_aPD1	GSM2445703	1.344262	1	NA	NA	0	NA
Prat_LUSC_pre_aPD1	GSM2445704	0	1	NA	NA	0	NA
Prat_LUSC_pre_aPD1	GSM2445705	5.47541	1	NA	NA	0	NA
Prat_LUSC_pre_aPD1	GSM2445706	2.557377	1	NA	NA	0	NA
Prat_LUSC_pre_aPD1	GSM2445707	7.540984	1	NA	NA	1	NA
Prat_LUSC_pre_aPD1	GSM2445708	2.754098	1	NA	NA	0	NA
Prat_LUSC_pre_aPD1	GSM2445709	25.67213	0	NA	NA	1	NA
Prat_LUSC_pre_aPD1	GSM2445710	12.13115	1	NA	NA	0	NA
Prat_LUSC_pre_aPD1	GSM2445711	23.04918	0	NA	NA	1	NA
Prat_LUSC_pre_aPD1	GSM2445712	2.262295	1	NA	NA	0	NA
Prat_LUSC_pre_aPD1	GSM2445713	6.032787	1	NA	NA	0	NA
Prat_LUSC_pre_aPD1	GSM2445714	4.557377	1	NA	NA	0	NA
Prat_LUSC_pre_aPD1	GSM2445715	2.032787	0	NA	NA	0	NA
Prat_NSCLC_pre_aPD1	GSM2445681	25.31148	0	NA	NA	1	NA
Prat_NSCLC_pre_aPD1	GSM2445682	0.983607	1	NA	NA	0	NA
Prat_NSCLC_pre_aPD1	GSM2445683	0	1	NA	NA	0	NA
Prat_NSCLC_pre_aPD1	GSM2445684	1.901639	0	NA	NA	0	NA
Prat_NSCLC_pre_aPD1	GSM2445685	2.754098	1	NA	NA	0	NA
Prat_NSCLC_pre_aPD1	GSM2445686	2.819672	0	NA	NA	0	NA
Prat_NSCLC_pre_aPD1	GSM2445687	3.704918	1	NA	NA	0	NA
Prat_NSCLC_pre_aPD1	GSM2445688	4.819672	0	NA	NA	1	NA
Prat_NSCLC_pre_aPD1	GSM2445689	3.147541	1	NA	NA	0	NA
Prat_NSCLC_pre_aPD1	GSM2445690	1.836066	0	NA	NA	0	NA
Prat_NSCLC_pre_aPD1	GSM2445691	0.95082	1	NA	NA	0	NA
Prat_NSCLC_pre_aPD1	GSM2445692	11.90164	0	NA	NA	0	NA
Prat_NSCLC_pre_aPD1	GSM2445693	1.377049	1	NA	NA	0	NA
Prat_NSCLC_pre_aPD1	GSM2445694	6.196721	1	NA	NA	1	NA
Prat_NSCLC_pre_aPD1	GSM2445695	20.36066	1	NA	NA	0	NA
Prat_NSCLC_pre_aPD1	GSM2445696	18.39344	1	NA	NA	1	NA
Prat_NSCLC_pre_aPD1	GSM2445697	0.688525	1	NA	NA	0	NA

Prat_NSCLC_pre_aPD1	GSM2445698	0.688525	1	NA	NA	0	NA
Prat_NSCLC_pre_aPD1	GSM2445699	6.754098	1	NA	NA	1	NA
Prat_NSCLC_pre_aPD1	GSM2445700	3.409836	1	NA	NA	0	NA
Prat_NSCLC_pre_aPD1	GSM2445701	4.819672	0	NA	NA	1	NA
Prat_NSCLC_pre_aPD1	GSM2445702	2.754098	1	NA	NA	0	NA
Cho_NSCLC_pre_aPD1	GSM3589675	NA	NA	NA	NA	0	NA
Cho_NSCLC_pre_aPD1	GSM3589677	NA	NA	NA	NA	1	NA
Cho_NSCLC_pre_aPD1	GSM3589678	NA	NA	NA	NA	1	NA
Cho_NSCLC_pre_aPD1	GSM3589679	NA	NA	NA	NA	0	NA
Cho_NSCLC_pre_aPD1	GSM3589683	NA	NA	NA	NA	0	NA
Cho_NSCLC_pre_aPD1	GSM3589686	NA	NA	NA	NA	0	NA
Cho_NSCLC_pre_aPD1	GSM3589688	NA	NA	NA	NA	0	NA
Jung_NSCLC_pre_aPD1	GSM3995402	NA	NA	NA	NA	0	NA
Jung_NSCLC_pre_aPD1	GSM3995403	NA	NA	NA	NA	1	NA
Jung_NSCLC_pre_aPD1	GSM3995404	NA	NA	NA	NA	0	NA
Jung_NSCLC_pre_aPD1	GSM3995405	NA	NA	NA	NA	0	NA
Jung_NSCLC_pre_aPD1	GSM3995406	NA	NA	NA	NA	1	NA
Jung_NSCLC_pre_aPD1	GSM3995407	NA	NA	NA	NA	0	NA
Jung_NSCLC_pre_aPD1	GSM3995408	NA	NA	NA	NA	0	NA
Jung_NSCLC_pre_aPD1	GSM3995409	NA	NA	NA	NA	1	NA
Jung_NSCLC_pre_aPD1	GSM3995410	NA	NA	NA	NA	0	NA
Jung_NSCLC_pre_aPD1	GSM3995411	NA	NA	NA	NA	0	NA
Jung_NSCLC_pre_aPD1	GSM3995412	NA	NA	NA	NA	1	NA
Jung_NSCLC_pre_aPD1	GSM3995413	NA	NA	NA	NA	1	NA
Jung_NSCLC_pre_aPD1	GSM3995414	NA	NA	NA	NA	0	NA
Jung_NSCLC_pre_aPD1	GSM3995415	NA	NA	NA	NA	0	NA
Jung_NSCLC_pre_aPD1	GSM3995416	NA	NA	NA	NA	0	NA
Jung_NSCLC_pre_aPD1	GSM3995417	NA	NA	NA	NA	0	NA
Jung_NSCLC_pre_aPD1	GSM3995418	NA	NA	NA	NA	1	NA
Jung_NSCLC_pre_aPD1	GSM3995419	NA	NA	NA	NA	1	NA
Jung_NSCLC_pre_aPD1	GSM3995420	NA	NA	NA	NA	1	NA
Jung_NSCLC_pre_aPD1	GSM3995421	NA	NA	NA	NA	0	NA
Jung_NSCLC_pre_aPD1	GSM3995422	NA	NA	NA	NA	0	NA
Jung_NSCLC_pre_aPD1	GSM3995423	NA	NA	NA	NA	0	NA
Jung_NSCLC_pre_aPD1	GSM3995424	NA	NA	NA	NA	0	NA
Jung_NSCLC_pre_aPD1	GSM3995425	NA	NA	NA	NA	0	NA
Jung_NSCLC_pre_aPD1	GSM3995426	NA	NA	NA	NA	0	NA
Jung_NSCLC_pre_aPD1	GSM3995427	NA	NA	NA	NA	0	NA
Jung_NSCLC_pre_aPD1	GSM3995428	NA	NA	NA	NA	0	NA

Supplementary table 15. Summary of 17 immunotherapy cohorts with CRISPR.

ID	PMID	Reference
Freeman 2019--NK--Melanoma--B16	31509742	31509742

Freeman 2019--OT1--Melanoma--B16	31509742	
Kearney 2018--NK_10--Colon--MC38	29776993	29776993
Kearney 2018--T_IgG--Colon--MC38	29776993	
Kearney 2018--T_PD1--Colon--MC38	29776993	
Lawson 2020--Mid_CTL--Breast--EMT6HA	32968282	32968282
Lawson 2020--Mid_CTL--Breast--X4T1HA	32968282	
Lawson 2020--Mid_CTL--Colon--CT26HA	32968282	
Lawson 2020--Mid_CTL--Colon--MC38OVA	32968282	
Lawson 2020--Mid_CTL--Melanoma--B16OVA	32968282	
Lawson 2020--Mid_CTL--Renal--RencaHA	32968282	
Manguso 2017--GVAX--Melanoma--B16	28723893	28723893
Manguso 2017--GVAX+PD1--Melanoma--B16	28723893	
Pan 2018--OT1--Melanoma--B16	29301958	29301958
Pan 2018--Pmel1--Melanoma--B16	29301958	
Patel 2017--1--Melanoma--Mel624	28783722	28783722
Vredevoogd 2019--MART1--Melanoma--D10 IFNGR1-	31303383	31303383

Supplementary table 16. Summary of 1793 immune related genes, associated with 17 immune related pathways.

AZGP1	MMP12	NLRX1	CCL7	IGHV7-81	ANGPT1	TGFBR2	IL9	TRAJ2
B2M	BPIFB6	PGC	CCL3	IGK	ANGPT4	TGFBR3	INHA	TRAJ3
CALR	LEAP2	VEGFA	CCL11	IGKC	ANGPTL1	THRA	INHBA	TRAJ4
CANX	SFTPD	IKBKE	CCR5	IGKDEL	ANGPTL2	THRB	INHBB	TRAJ5
CD1A	LCN9	ISG15	CCL23	IGKJ	ANGPTL3	TIE1	INHBC	TRAJ6
CD1B	BPIFB2	DHX58	CCL25	IGKJ1	ANGPTL4	TNFRSF10C	INHBE	TRAJ7
CD1C	PTGDS	TNFAIP3	CCL3L3	IGKJ2	ANGPTL6	TNFRSF10D	INS	TRAJ8
CD1D	TMSB4X	TFR2	CCL4L2	IGKJ3	APLNR	TNFRSF11A	INS-IGF2	TRAJ9
CD1E	PGLYRP1	FCN2	CCL3L1	IGKJ4	AR	TNFRSF12A	INSL3	TRAJ10

CD4	ZC3HAV1	MUC4	CCR1	IGKJ5	AVPR1A	TNFRSF13B	INSL4	TRAJ11
CD8A	TMSB15A	F2R	CCL24	IGKV@	AVPR1B	TNFRSF13C	INSL5	TRAJ12
CD8B	S100B	ELN	XCL2	IGKV1-12	AVPR2	TNFRSF14	INSL6	TRAJ13
CD74	S100A13	IL27	CXCR4	IGKV1-13	BMPR1A	TNFRSF17	JAG1	TRAJ14
CREB1	S100A6	MAPT	CXCR6	IGKV1-16	BMPR1B	TNFRSF18	JAG2	TRAJ15
CTSB	DEFB119	LYZ	CCR4	IGKV1-17	BMPR2	TNFRSF19	FGF7P6	TRAJ16
CTSE	DEFB107A	CCL5	TAFA5	IGKV1-27	BRD8	TNFRSF1A	FGF7P3	TRAJ17
CTSL	DEFB105A	LEP	TAFA3	IGKV1-33	C3AR1	TNFRSF1B	KITLG	TRAJ18
CTSS	SERPIND1	CYLD	TAFA4	IGKV1-37	CALCR	TNFRSF21	KL	TRAJ19
FCER1G	DEFB129	KLKB1	TAFA1	IGKV1-39	CALCRL	TNFRSF25	LACRT	TRAJ20
FCGRT	DEFB127	CST4	TAFA2	IGKV1-5	CNTFR	TNFRSF4	LEFTY1	TRAJ21
PDIA3	S100P	CSRP1	CCL15-CCL14	IGKV1-6	CRHR1	TNFRSF6B	LEFTY2	TRAJ22
HFE	S100A7	MAPK14	PTK2B	IGKV1-8	CRHR2	TNFRSF8	LHB	TRAJ23
HLA-A	DEFB104A	JUN	IL4	IGKV1-9	CRIM1	TNFRSF9	LIF	TRAJ24
HLA-B	DEFB126	ITGAV	CDH1	IGKV1D-12	CRLF1	TRHR	LRSAM1	TRAJ25
HLA-C	DEFB106B	IRF5	LTBP1	IGKV1D-13	CRLF2	TSHR	LTB	TRAJ26
HLA-DMA	DEFB104B	CCR6	IL13	IGKV1D-16	CRLF3	TUBB3	LTBP2	TRAJ27
HLA-DMB	DEFB107B	IL12B	IL10	IGKV1D-17	CSF1R	VIPR1	LTBP3	TRAJ28
HLA-DOA	PGLYRP3	TLR8	IL2	IGKV1D-33	CSF2RA	VIPR2	LTBP4	TRAJ29
HLA-DOB	PGLYRP2	GNLY	PPARG	IGKV1D-37	CSF2RB	ADM	MDK	TRAJ30
HLA-DPA1	S100A10	CD81	FGR	IGKV1D-39	CSF3R	ADM2	MIA	TRAJ31
HLA-DPB1	S100A2	EIF2AK2	MIF	IGKV1D-42	EGFR	AGRP	MLN	TRAJ32
HLA-DQA1	DEFB125	APOM	CRP	IGKV1D-43	ENG	AGT	MSTN	TRAJ33
HLA-DQA2	DEFB123	CACYBP	JAK2	IGKV1D-8	EPOR	AMBN	NAMPT	TRAJ34
HLA-DQB1	DEFB105B	NOD1	PTK2	IGKV2-24	ESR1	AMELX	NDP	TRAJ35
HLA-DRA	DEFB132	MAPK8	PTGDR	IGKV2-28	ESR2	AMH	NENF	TRAJ36
HLA-DRB1	BPIFB3	MAPK3	CD86	IGKV2-30	ESRRA	ANGPTL5	NGF	TRAJ37
HLA-DRB3	LCN12	BST2	HCK	IGKV2-40	ESRRB	ANGPTL7	NMB	TRAJ38
HLA-DRB4	PGLYRP4	BPHL	VDR	IGKV2D-24	ESRRG	APLN	NODAL	TRAJ39
HLA-DRB5	S100A11	PLA2G2A	OLR1	IGKV2D-28	FGFR1	AREG	CCN3	TRAJ40
HLA-E	S100A5	GRN	GRK2	IGKV2D-29	FGFR2	MANF	NPFF	TRAJ41
HLA-F	S100A3	NEWENTRY	TXK	IGKV2D-30	FGFR3	CDNF	NPPA	TRAJ42
HLA-G	S100A1	PDGFRA	RNASE2	IGKV2D-40	FGFR4	ARTN	NPPB	TRAJ43
HLA-H	DEFB128	GNAI1	CD79A	IGKV3-11	FGFRL1	AVP	NPPC	TRAJ44
MR1	DEFB108B	WNT5A	CD79B	IGKV3-15	FLT1	BDNF	NPY	TRAJ45
HSPA1A	HTN1	FURIN	LYN	IGKV3-20	FLT3	BMP1	NRG1	TRAJ46
HSPA1B	LMBR1L	ADAR	SYK	IGKV3-7	FLT4	BMP10	NRG2	TRAJ47
HSPA1L	S100A7A	TYK2	BTK	IGKV3D-11	FSHR	BMP15	NRG3	TRAJ48
HSPA2	DEFB118	NOS2	BLNK	IGKV3D-15	GALR2	BMP2	NRG4	TRAJ49
HSPA4	COLEC12	TRAF3	VAV3	IGKV3D-20	GALR3	BMP3	NRTN	TRAJ50
HSPA5	TMSB4Y	TPT1	VAV1	IGKV3D-7	GCGR	BMP4	NTF3	TRAJ52
HSPA6	DEFB131A	TPM2	VAV2	IGKV4-1	GHR	BMP5	NTF4	TRAJ53
HSPA8	DEFB134	NEO1	RAC1	IGKV5-2	GHRHR	BMP6	NTS	TRAJ54
HSP90AA1	DEFB130A	AHNAK	RAC2	IGKV6-21	GHSR	BMP7	NUDT6	TRAJ56

HSP90AB1	DEFB124	TLR1	RAC3	IGKV6D-21	GIPR	BMP8A	OGN	TRAJ57
ICAM1	DEFB121	TK2	PPP3CA	IGKV6D-41	GLP1R	BMP8B	OSGIN1	TRAJ58
IFNA1	DEFB116	PRDX2	PPP3CB	IGL	GLP2R	BTC	OSM	TRAJ59
IFNA2	DEFB115	MX2	PPP3CC	IGLC1	GNRHR	MYDGF	OSTN	TRAJ61
IFNA4	DEFB114	FGF2	CHP1	IGLC2	GPER1	CALCA	OXT	TRAV1-1
IFNA5	DEFB113	FGA	PPP3R1	IGLC3	HNF4A	CALCB	ENDOU	TRAV1-2
IFNA6	DEFB112	TCF7L2	PPP3R2	IGLC6	HNF4G	CAT	PDGFA	TRAV2
IFNA7	DEFB110	F2RL1	CHP2	IGLC7	HTR3A	CCK	PDGFB	TRAV3
IFNA8	TMSB15B	TKFC	NFAT5	IGLJ	HTR3B	CD320	PDGFC	TRAV4
IFNA10	DEFB133	MSR1	NFATC1	IGLJ1	HTR3C	CD70	PDGFD	TRAV5
IFNA13	S100Z	NFKBIZ	NFATC2	IGLJ2	HTR3D	ADA2	PDGFRL	TRAV7
IFNA14	MAVS	LMBR1	NFATC3	IGLJ3	HTR3E	CER1	PGF	TRAV8-1
IFNA16	TMSB4XP8	EPPIN	NFATC4	IGLJ4	IFNGR2	CGA	PMCH	TRAV8-2
IFNA17	S100A14	SRC	HRAS	IGLJ5	IGF1R	CGB3	PNOC	TRAV8-3
IFNA21	LCN10	MPO	KRAS	IGLJ6	IGF2R	CGB1	POMC	TRAV8-4
IFNG	S100A16	ELAVL1	NRAS	IGLJ7	IL10RA	CGB2	PPBPP2	TRAV8-6
KIR2DL1	DEFB136	ROBO3	FOS	IGLV@	IL10RB	CGB5	PPY	TRAV8-7
KIR2DL2	DEFB135	SP1	CARD11	IGLV1-36	IL11RA	CGB7	PRL	TRAV9-1
KIR2DL3	DEFB117	SOD1	BCL10	IGLV1-40	IL12RB1	CGB8	PRLH	TRAV9-2
KIR2DL4	ZC3HAV1L	PDF	MALT1	IGLV1-44	IL12RB2	CHGA	PROK1	TRAV10
KIR2DS1	S100A7L2	DLL4	CHUK	IGLV1-47	IL13RA1	CHGB	PSPN	TRAV12-1
KIR2DS3	MBL3P	ECD	IKBKB	IGLV1-50	IL13RA2	CLCF1	PTH	TRAV12-2
KIR2DS4	DEFB4B	SLC11A1	IKBKG	IGLV1-51	IL15RA	CLEC11A	PTH2	TRAV12-3
KIR2DS5	BPIFB4	DMBT1	NFKBIA	IGLV10-54	IL2RB	CMTM1	PTHLH	TRAV13-1
KIR3DL1	IFNAR1	STING1	NFKBIB	IGLV11-55	IL17RA	CMTM2	PTN	TRAV13-2
KIR3DL2	AZU1	SKIV2L	NFKBIE	IGLV2-11	IL17RB	CMTM3	PYY	TRAV14DV4
KLRC1	DEFB131B	SEMG2	CD19	IGLV2-14	IL17RC	CMTM4	QRFP	TRAV16
KLRC2	DEFA1A3	DES	CR2	IGLV2-18	IL17RD	CMTM5	RABEP1	TRAV17
KLRC3	LCN1P1	DCK	PIK3R5	IGLV2-23	IL17RE	CMTM6	RABEP2	TRAV18
KLRD1	S100G	DAXX	PIK3R1	IGLV2-33	IL18R1	CMTM7	REG1A	TRAV19
LTA	DEFA7P	TNFRSF10A	PIK3R2	IGLV2-8	IL18RAP	CMTM8	RETN	TRAV20
CIITA	DEFB130B	TNFRSF10B	PIK3R3	IGLV3-1	IL1R1	CNTF	RETNLB	TRAV21
MICA	DEFB108F	EED	PIK3CA	IGLV3-10	IL1R2	CORT	RLN1	TRAV22
MICB	DEFB131C	CCL4	PIK3CB	IGLV3-12	IL1RAP	CRH	RLN2	TRAV23DV6
NFYA	TCHHL1	LIMS1	PIK3CD	IGLV3-16	IL1RL1	CSF1	RLN3	TRAV24
NFYB	TINAGL1	LALBA	AKT3	IGLV3-19	IL1RL2	CSF2	SCG2	TRAV25
NFYC	IFNGR1	APOBEC3H	AKT1	IGLV3-21	IL20RA	CSF3	SCGB3A1	TRAV26-1
LGMN	SLC22A17	TMPRSS6	AKT2	IGLV3-22	IL20RB	CSH1	SCT	TRAV26-2
PSMB8	WFIKKN1	SPINK5	GSK3B	IGLV3-25	IL21R	CSH2	AIMP1	TRAV27
PSMC1	WFDC2	MARCO	INPP5D	IGLV3-27	IL22RA1	CSHL1	SECTM1	TRAV29DV5
PSMC2	IL6	BECN1	CD22	IGLV3-32	IL22RA2	CSPG5	SLURP1	TRAV30
PSMC3	UMODL1	TNFSF11	CD72	IGLV3-9	IL23R	CTF1	SPP1	TRAV34
PSMC4	TGFB1	KNG1	PTPN6	IGLV4-3	IL27RA	CCN2	SST	TRAV35
PSMC5	PF4V1	CSK	LILRB3	IGLV4-60	IL2RA	DKK1	STC1	TRAV36DV7

PSMC6	MMP9	KLRK1	FCGR2B	IGLV4-69	IL2RG	EBI3	STC2	TRAV38-1
PSMD1	ANOS1	KCNH2	RASGRP3	IGLV5-37	IL31RA	EGF	TAC1	TRAV38-2DV8
PSMD2	TLR4	JUND	PLCG2	IGLV5-39	IL3RA	EPGN	TDGF1	TRAV39
PSMD3	SPAG11B	JAK1	PRKCB	IGLV5-45	IL4R	EPO	TDGF1P3	TRAV40
PSMD4	A2M	CLDN4	IFITM1	IGLV5-48	IL5RA	EREG	TG	TRAV41
PSMD5	NFKB1	CCL28	IGH	IGLV5-52	IL6R	ESM1	TGFA	TRBC1
PSMD7	APOBEC3G	RNASE3	IGHA1	IGLV6-57	IL9R	FAM3B	TGFB2	TRBC2
PSMD8	FABP6	RN7SL1	IGHA2	IGLV7-43	INSR	FAM3C	TGFB3	TRBD1
PSMD10	NOD2	IRF7	IGHD	IGLV7-46	KDR	FAM3D	THPO	TRBD2
PSMD11	MBL2	IREB2	IGHD1-1	IGLV8-61	LEPR	FGF1	TNFRSF11B	TRBJ1-1
PSMD13	SFTPA1	ILK	IGHD1-14	IGLV9-49	LGR4	FGF11	TNFSF12	TRBJ1-2
PSME1	RBP1	IL18	IGHD1-20	C5AR1	LGR5	FGF12	TNFSF13	TRBJ1-3
PSME2	TLR2	IL17A	IGHD1-26	CCR9	LGR6	FGF13	TNFSF13B	TRBJ1-4
RELB	SLC40A1	LTB4R	IGHD1-7	CCRL2	LHCGR	FGF14	TNFSF14	TRBJ1-5
RFX5	PLAU	APOBEC3A	IGHD2-15	CMKLR1	LIFR	FGF16	TNFSF15	TRBJ1-6
RFXAP	IL1B	MASP2	IGHD2-2	CX3CR1	LTBR	FGF17	TNFSF18	TRBJ2-1
SLC10A2	PAEP	TRIM27	IGHD2-21	CXCR3	MC1R	FGF18	TNFSF8	TRBJ2-2
TAP1	HJV	RELA	IGHD2-8	CXCR5	MC2R	FGF19	TNFSF9	TRBJ2-3
TAP2	MUC5AC	IL7R	IGHD3-10	ACKR3	MC3R	FGF20	TOR2A	TRBJ2-4
TAPBP	OBP2A	IL1A	IGHD3-16	CYSLTR1	MC4R	FGF21	TRH	TRBJ2-5
THBS1	PLTP	PTX3	IGHD3-22	CYSLTR2	MCHR1	FGF22	TSHB	TRBJ2-6
SEM1	MX1	IFNAR2	IGHD3-3	ACKR1	MCHR2	FGF23	TSLP	TRBJ2-7
KLRC4	DDX58	IFN1@	IGHD3-9	EDNRA	MET	FGF3	TXLNA	TRBV2
AP3B1	IFNL1	SYTL1	IGHD4-11	EDNRB	MLNR	FGF4	UCN	TRBV3-1
RFXANK	IRF3	APOBEC3C	IGHD4-17	FPR1	MPL	FGF5	UCN2	TRBV4-1
PSMD6	SFTPA2	DDX17	IGHD4-23	FPR2	MTNR1A	FGF6	UCN3	TRBV4-2
PSME3	LPA	PTGS2	IGHD4-4	GPR17	MTNR1B	FGF7	UTS2	TRBV4-3
PSMD14	LBP	HTR1A	IGHD5-12	GPR32	NGFR	FGF8	UTS2B	TRBV5-1
CLEC4M	RBP4	SEPTIN7	IGHD5-18	GPR33	NMBR	FGF9	VEGFB	TRBV5-4
IFI30	NOX4	CD40LG	IGHD5-24	PTGDR2	NPR1	VEGFD	VEGFC	TRBV5-5
PROCR	LTF	CD14	IGHD5-5	C5AR2	NPR3	FIGNL2	VGF	TRBV5-6
ADRM1	IFNB1	MASP1	IGHD6-13	CXCR2	NR0B1	FLT3LG	VIP	TRBV5-7
ECPAS	RBP5	PROC	IGHD6-19	LTB4R2	NR0B2	FSHB	PTPN11	TRBV5-8
TRPC4AP	FABP7	MAP2K2	IGHD6-25	PLAUR	NR1D1	GAL	ICAM2	TRBV6-1
CD209	FABP5	MAP2K1	IGHD6-6	PLXNA1	NR1D2	GALP	ITGAL	TRBV6-2
UBXN1	FABP3	HRG	IGHD7-27	PLXNA2	NR1H2	GAST	ITGB2	TRBV6-3
ERAP1	FABP2	NDRG1	IGHE	PLXNA3	NR1H3	GCG	PAK1	TRBV6-4
TAPBPL	FABP4	IRF9	IGHG1	PLXNA4	NR1H4	GDF1	NCR2	TRBV6-5
KIR2DL5A	R3HDML	TRIM22	IGHG2	PLXNB1	NR1I2	GDF10	TYROBP	TRBV6-6
ERAP2	BPIFA3	LANCL1	IGHG3	PLXNB2	NR1I3	GDF11	LCK	TRBV6-7
ULBP3	BPIFB1	PPP4C	IGHG4	PLXNB3	NR2C1	GDF2	FCGR3A	TRBV6-8
ULBP2	OASL	HMOX1	IGHJ1	PLXNC1	NR2C2	GDF3	FCGR3B	TRBV6-9
ULBP1	CRABP2	HMGB1	IGHJ2	PLXND1	NR2E1	GDF5	NCR1	TRBV7-2
KIR3DL3	CRABP1	RNASE7	IGHJ3	PTAFR	NR2E3	GDF6	NCR3	TRBV7-3

RAET1E	RBP7	ABCC4	IGHJ4	ROBO1	NR2F1	GDF7	CD247	TRBV7-4
RAET1L	DUOX1	HGF	IGHJ5	ROBO2	NR2F2	GDF9	ZAP70	TRBV7-6
UBR1	OBP2B	HDAC1	IGHJ6	RXFP3	NR2F6	GDNF	LCP2	TRBV7-7
RAET1G	RBP2	IFNLR1	IGHM	XCR1	NR3C1	GH1	LAT	TRBV7-8
PDIA2	LCN15	PLSCR1	IGHV1-18	C3	NR3C2	GH2	PLCG1	TRBV7-9
HAMP	CETP	BACH2	IGHV1-2	C5	NR4A1	GHRH	SH3BP2	TRBV9
PI3	FABP12	TANK	IGHV1-24	CCL3P1	NR4A2	GHRL	FYN	TRBV10-1
CAMP	FABP9	PIK3CG	IGHV1-3	CKLF	NR4A3	GIP	SHC2	TRBV10-2
DEFB4A	BPIFA1	ARRB1	IGHV1-45	CMA1	NR5A1	GKN1	SHC4	TRBV10-3
PPBP	LCNL1	RSAD2	IGHV1-46	CX3CL1	NR5A2	GMFB	SHC3	TRBV11-1
REG3G	C8G	STAB2	IGHV1-58	CXCL17	NR6A1	GMFG	SHC1	TRBV11-2
CXCL14	SPAG11A	TBK1	IGHV1-69	CCN1	NRP1	GNRH1	GRB2	TRBV11-3
CXCL16	PI15	PDYN	IGHV1-8	EDN1	NRP2	GNRH2	SOS1	TRBV12-3
SLPI	NOX1	PDGFRB	IGHV1-38-4	EDN2	OGFR	GPHA2	SOS2	TRBV12-4
CXCL8	PMP2	PDCD1	IGHV1-69-2	EDN3	OPRD1	GPHB5	ARAF	TRBV12-5
CXCL10	APOD	PCSK2	IGHV2-26	FGF10	OPRK1	GPI	BRAF	TRBV13
CXCL9	ORM2	PCSK1	IGHV2-5	LECT2	OPRL1	GREM1	RAF1	TRBV14
CXCL5	ORM1	ARG2	IGHV2-70	PPBPP1	OPRM1	GREM2	HCST	TRBV15
CXCL11	TNF	AQP9	IGHV3-11	PROK2	OSMR	GRP	CD48	TRBV16
CXCL6	CTSG	FASLG	IGHV3-13	SAA1	OXTR	GUCA2A	CD244	TRBV17
CXCL1	PRTN3	APOH	IGHV3-15	SAA2	PGR	HBEGF	PRKCA	TRBV18
CXCL12	MAPK1	BIRC5	IGHV3-16	SBDS	PGRMC2	HDGF	PRKCG	TRBV19
CXCL13	PML	ANXA6	IGHV3-20	SEMA3A	PPARA	HDGFL3	SH2D1B	TRBV20-1
CXCL2	AEN	IL22	IGHV3-21	SEMA3B	PPARD	IAPP	SH2D1A	TRBV24-1
PF4	CYBB	VTN	IGHV3-23	SEMA3C	PRLHR	IFNE	FAS	TRBV25-1
XCL1	BPIFA2	VIM	IGHV3-30	SEMA3D	PRLR	IFNK	GZMB	TRBV27
CXCL3	ISG20	VCAM1	IGHV3-30-3	SEMA3E	PTGER1	IFNW1	PRF1	TRBV28
DEFB103B	BCL3	PRDX1	IGHV3-30-5	SEMA3F	PTGER2	IGF1	CASP3	TRBV29-1
CCL13	ISG20L2	GFAP	IGHV3-33	SEMA3G	PTGER3	IGF2	BID	TRBV30
CCL1	NOX5	GBP2	IGHV3-35	SEMA4A	PTGER4	IL11	CD3D	TRDC
DEFB1	NOX3	ALB	IGHV3-38	SEMA4B	PTGFR	IL12A	CD3E	TRDD1
CCL8	DUOX2	SLC29A3	IGHV3-43	SEMA4C	PTH1R	IL16	CD3G	TRDD2
ELANE	TLR3	OAS1	IGHV3-48	SEMA4D	PTH2R	IL17B	PTPRC	TRDD3
DEFB103A	TFRC	AGER	IGHV3-49	SEMA4F	RARA	IL17C	ITK	TRDJ1
DEFA3	IFIH1	UNC93B1	IGHV3-53	SEMA4G	RARB	IL17D	TEC	TRDJ2
DEFA1	LRP1	TNFSF4	IGHV3-64	SEMA5A	RARG	IL17F	NCK1	TRDJ3
TMSB10	TRIM5	NOS1	IGHV3-66	SEMA5B	RORA	IL19	NCK2	TRDJ4
DEFA6	IDO1	ACTG1	IGHV3-7	SEMA6A	RORB	IL1F10	GRAP2	TRDV1
DEFA5	GDF15	ACTA1	IGHV3-72	SEMA6B	RORC	IL36RN	PAK2	TRDV2
DEFA4	NEDD4	ACO1	IGHV3-73	SEMA6C	RXFP1	IL36A	PAK3	TRDV3
LCN2	ADIPOQ	SERPINA3	IGHV3-74	SEMA6D	RXFP2	IL37	PAK4	TRGV9
LCN1	STAT3	CXCR1	IGHV3-9	SEMA7A	RXRA	IL36B	PAK6	TRGV8
COLEC10	STAT1	CCL15	IGHV3-38-3	SLIT1	RXRB	IL36G	PAK5	TRGV5
BPI	IFNL2	CCL14	IGHV3-69-1	SLIT2	RXRG	IL1RN	RHOA	TRGV4

S100A9	SOCS3	CCL16	IGHV4-28	TNC	S1PR1	IL20	CDC42	TRGV3
S100A8	SEMG1	CCL19	IGHV4-30-1	TYMP	S1PR2	IL21	CD28	TRGV2
DCD	TNFSF10	CCL18	IGHV4-30-2	ACVR1B	SCTR	IL23A	ICOS	TRGJP2
LCN6	CCL20	CCL17	IGHV4-30-4	ACVR1C	SDC1	IL24	MAP3K8	TRGJP1
S100A12	SOCS1	CCL26	IGHV4-31	ACVR2A	SDC2	IL25	MAP3K14	TRGJP
HTN3	RNASEL	CCL22	IGHV4-34	ACVR2B	SDC3	IL26	CTLA4	TRGJ2
LCN8	IRF1	CCR3	IGHV4-39	ACVRL1	SDC4	IFNL3	CBLC	TRGJ1
DEFA1B	IL15	CCL4L1	IGHV4-4	ADCYAP1R1	SORT1	IL3	CBL	TRGC2
CCR10	APOBEC3F	ACKR2	IGHV4-59	ADIPOR1	SSTR1	IL31	CBLB	TRGC1
CELA1	PLAAT4	CCR7	IGHV4-61	ADIPOR2	SSTR2	IL32	CDK4	TRAV6
DEFB106A	CHIT1	CCL27	IGHV4-38-2	ADRB1	SSTR5	IL33	RASGRP1	
PENK	CD40	CCR8	IGHV5-51	ADRB2	ST2	IL34	PDK1	
BPIFC	TLR7	ACKR4	IGHV5-10-1	AGTR1	TACR1	IL5	PRKCQ	
	PPIA	CCL2	IGHV6-1	AGTR2	TEK	IL6ST	TRAC	
	ZYX	CCL21	IGHV7-4-1	AMHR2	TGFBR1	IL7	TRAJ1	

Supplementary table 17. Summary of 75 immune checkpoint genes.

Genes	Category	ICI
CD80	Co-stimulator	Stimulatory
CD28	Co-stimulator	Stimulatory
ICOSLG	Co-stimulator	Stimulatory
PDCD1LG2	Co-inhibitor	N/A
CD274	Co-inhibitor	Inhibitory
VTCN1	Co-inhibitor	Inhibitory
SLAMF7	Co-inhibitor	Inhibitory
BTN3A2	Co-inhibitor	Stimulatory
BTN3A1	Co-inhibitor	Stimulatory
C10orf54	Co-inhibitor	Inhibitory
CD276	Co-inhibitor	Inhibitory
TNFSF9	Ligand	Stimulatory
TNF	Ligand	Stimulatory
TNFSF4	Ligand	Stimulatory
IL1B	Ligand	Stimulatory
CXCL9	Ligand	Stimulatory
CXCL10	Ligand	Stimulatory
CCL5	Ligand	Stimulatory
VEGFB	Ligand	Inhibitory
CX3CL1	Ligand	Stimulatory
TGFB1	Ligand	Inhibitory
VEGFA	Ligand	Inhibitory
CD70	Ligand	Stimulatory
CD40LG	Ligand	Stimulatory
IL10	Ligand	Inhibitory
IFNG	Ligand	Stimulatory

IL1A	Ligand	Stimulatory
IL12A	Ligand	Stimulatory
IFNA2	Ligand	Stimulatory
IFNA1	Ligand	Stimulatory
IL4	Ligand	Inhibitory
IL2	Ligand	Stimulatory
IL13	Ligand	Inhibitory
TNFRSF18	Receptor	Stimulatory
TIGIT	Receptor	Inhibitory
PDCD1	Receptor	Inhibitory
CTLA4	Receptor	Inhibitory
IL2RA	Receptor	Stimulatory
TNFRSF4	Receptor	Stimulatory
CD27	Receptor	Stimulatory
LAG3	Receptor	Inhibitory
TNFRSF9	Receptor	Stimulatory
ICOS	Receptor	Stimulatory
BTLA	Receptor	Inhibitory
KIR2DL3	Receptor	Inhibitory
KIR2DL1	Receptor	Inhibitory
TNFRSF14	Receptor	Stimulatory
EDNRB	Receptor	Inhibitory
CD40	Receptor	Stimulatory
ADORA2A	Receptor	Inhibitory
TLR4	Receptor	Stimulatory
HAVCR2	Receptor	Inhibitory
ITGB2	Cell adhesion	Stimulatory
ICAM1	Cell adhesion	Stimulatory
SELP	Cell adhesion	Stimulatory
HLA-DRB5	Antigen presentation	N/A
HLA-DQA1	Antigen presentation	N/A
HLA-DQB1	Antigen presentation	N/A
MICA	Antigen presentation	N/A
MICB	Antigen presentation	N/A
HLA-DQA2	Antigen presentation	N/A
HLA-DQB2	Antigen presentation	N/A
HLA-B	Antigen presentation	N/A
HLA-A	Antigen presentation	N/A
HLA-C	Antigen presentation	N/A
HLA-DRA	Antigen presentation	N/A
HLA-DRB1	Antigen presentation	N/A
HLA-DPB1	Antigen presentation	N/A
HLA-DPA1	Antigen presentation	N/A
IDO1	Other	Inhibitory

GZMA	Other	Stimulatory
PRF1	Other	Stimulatory
ARG1	Other	Inhibitory
HMGB1	Other	Stimulatory
ENTPD1	Other	Stimulatory

Supplementary table 18. 100 genes of the signatures.

**2021.Cancer.cell_Pancancer_TME_29Fges**			**immune.contexture.related.92.signature.29628290**			**cancerSEA**			**anti-tumor immune cycle**					**Immunotherapy Response Signature**	
name	symbol		name	symbol		name	symbol		GeneSymbol	Steps	Direction	ImmuneCellType		name	symbol
MHCI	HLA.A		Activated.B.cell	ADAM28		Angiogenesis	ACVRL1		IL10	1	positive	Multiple		CAF	A1BG
MHCI	HLA.B		Activated.B.cell	CD180		Angiogenesis	JAG1		TGFB1	1	positive	Multiple		CAF	A1BG.AS1
MHCI	HLA.C		Activated.B.cell	CD79B		Angiogenesis	ANGPT1		HMGB1	1	positive	Multiple		CAF	A1CF
MHCI	B2M		Activated.B.cell	BLK		Angiogenesis	ANGPT2		ANXA1	1	positive	Multiple		CAF	A2M
MHCI	TAP1		Activated.B.cell	CD19		Angiogenesis	CD34		CALR	1	positive	Multiple		CAF	A2M.AS1
MHCI	TAP2		Activated.B.cell	MS4A1		Angiogenesis	CDC42		CXCL10	1	positive	Multiple		CAF	A2ML1
MHCI	TAPBP		Activated.B.cell	TNFRSF17		Angiogenesis	MAPK14		PDIA3	1	positive	Multiple		CAF	A2MP1

MHCII	HLA.DRA		Activated.B.cell	IGHM		Angiogenesis	TYMP		HSPA1A	1	positive	Multiple		CAF	A4GALT
MHCII	HLA.DRB1		Activated.B.cell	GNG7		Angiogenesis	EDN1		HSPA1B	1	positive	Multiple		CAF	A4GNT
MHCII	HLA.DMA		Activated.B.cell	MICAL3		Angiogenesis	EFNB2		HSPA2	1	positive	Multiple		CAF	AA06
MHCII	HLA.DPA1		Activated.B.cell	SPIB		Angiogenesis	EGR3		HSPA8	1	positive	Multiple		CAF	AAAS
MHCII	HLA.DPB1		Activated.B.cell	HLA.DOB		Angiogenesis	EPHB4		HSPA4	1	positive	Multiple		CAF	AACS
MHCII	HLA.DMB		Activated.B.cell	IGKC		Angiogenesis	PTK2B		HSPA14	1	positive	Multiple		CAF	AACSP1
MHCII	HLA.DQB1		Activated.B.cell	PNOC		Angiogenesis	FGF2		HSPA5	1	positive	Multiple		CAF	AADAC
MHCII	HLA.DQA1		Activated.B.cell	FCRL2		Angiogenesis	FGFR1		HSPA6	1	positive	Multiple		CAF	AADACL2
MHCII	CIITA		Activated.B.cell	BACH2		Angiogenesis	VEGFD		HSPA9	1	positive	Multiple		CAF	AADACP1
Co.activation.molecules	CD28		Activated.B.cell	CR2		Angiogenesis	FOXC2		HSPA13	1	positive	Multiple		CAF	AADAT
Co.activation.molecules	CD40		Activated.B.cell	TCL1A		Angiogenesis	FLT1		HSPA7	1	positive	Multiple		CAF	AAGAB

Co.activation.molecules	TNFRSF4		Activated.B.cell	AKNA		Angiogenesis	FLT4		HSPA8	1	positive	Multiple		CAF	AAK1
Co.activation.molecules	ICOS		Activated.B.cell	ARHGAP25		Angiogenesis	FN1		HSPA12A	1	positive	Multiple		CAF	AAMDC
Co.activation.molecules	TNFRSF9		Activated.B.cell	CCL21		Angiogenesis	GPLD1		HSPA12B	1	positive	Multiple		CAF	AAMP
Co.activation.molecules	CD27		Activated.B.cell	CD27		Angiogenesis	NR4A1		HSP90AA1	1	positive	Multiple		CAF	AANAT
Co.activation.molecules	CD80		Activated.B.cell	CD38		Angiogenesis	ID1		HSP90AB1	1	positive	Multiple		CAF	AARS
Co.activation.molecules	CD86		Activated.B.cell	CLEC17A		Angiogenesis	ITGA5		HSP90B1	1	positive	Multiple		CAF	AARS2
Co.activation.molecules	CD40LG		Activated.B.cell	CLEC9A		Angiogenesis	ITGAV		IFNA2	1	positive	Multiple		CAF	AASDH
Co.activation.molecules	CD83		Activated.B.cell	CLECL1		Angiogenesis	ITGB1		IFNA1	1	positive	Multiple		CAF	AASDHPPT
Co.activation.molecules	TNFSF4		Activated.CD4.T.cell	AIM2		Angiogenesis	KDR		IFNA13	1	positive	Multiple		CAF	AASS
Co.activation.molecules	ICOSLG		Activated.CD4.T.cell	BIRC3		Angiogenesis	LOXL2		IFNA6	1	positive	Multiple		CAF	AATBC
Co.activation.molecules	TNFSF9		Activated.CD4.T.cell	BRIP1		Angiogenesis	MMP14		IFNA21	1	positive	Multiple		CAF	AATF

Co.activation.molecules	CD70		Activated.CD4.T.cell	CCL20		Angiogenesis	NOTCH1		IFNA4	1	positive	Multiple		CAF	AATK
Effector.cells	IFNG		Activated.CD4.T.cell	CCL4		Angiogenesis	PDGFA		IFNA8	1	positive	Multiple		CAF	ABAT
Effector.cells	GZMA		Activated.CD4.T.cell	CCL5		Angiogenesis	PDGFRB		IFNA5	1	positive	Multiple		CAF	ABCA1
Effector.cells	GZMB		Activated.CD4.T.cell	CCNB1		Angiogenesis	PGF		IFNA7	1	positive	Multiple		CAF	ABCA11P
Effector.cells	PRF1		Activated.CD4.T.cell	CCR7		Angiogenesis	PIK3CA		IFNA14	1	positive	Multiple		CAF	ABCA12
Effector.cells	GZMK		Activated.CD4.T.cell	DUSP2		Angiogenesis	PTGS2		IFNA16	1	positive	Multiple		CAF	ABCA13
Effector.cells	ZAP70		Activated.CD4.T.cell	ESCO2		Angiogenesis	PTK2		IFNA10	1	positive	Multiple		CAF	ABCA17P
Effector.cells	GNLY		Activated.CD4.T.cell	ETS1		Angiogenesis	ROBO1		IFNA17	1	positive	Multiple		CAF	ABCA2
Effector.cells	FASLG		Activated.CD4.T.cell	EXO1		Angiogenesis	SHC1		IFNB1	1	positive	Multiple		CAF	ABCA3
Effector.cells	TBX21		Activated.CD4.T.cell	EXOC6		Angiogenesis	SRF		IFNE	1	positive	Multiple		CAF	ABCA4
Effector.cells	EOMES		Activated.CD4.T.cell	IARS		Angiogenesis	TAL1		IFNW1	1	positive	Multiple		CAF	ABCA5

Effector.cells	CD8A		Activated.CD4.T.cell	ITK		Angiogenesis	TDGF1		TNF	2	positive	Multiple		CAF	ABCA6
Effector.cells	CD8B		Activated.CD4.T.cell	KIF11		Angiogenesis	TEK		IL1A	2	positive	Multiple		CAF	ABCA7
Effector.cell.traffic	CXCL9		Activated.CD4.T.cell	KNTC1		Angiogenesis	VAV2		IL1B	2	positive	Multiple		CAF	ABCA8
Effector.cell.traffic	CXCL10		Activated.CD4.T.cell	NUF2		Angiogenesis	VEGFA		IFNA2	2	positive	Multiple		CAF	ABCA9
Effector.cell.traffic	CXCL11		Activated.CD4.T.cell	PRC1		Angiogenesis	VEGFC		IFNA1	2	positive	Multiple		CAF	ABCB1
Effector.cell.traffic	CX3CL1		Activated.CD4.T.cell	PSAT1		Angiogenesis	FGF18		IFNA13	2	positive	Multiple		CAF	ABCB10
Effector.cell.traffic	CCL3		Activated.CD4.T.cell	RGS1		Angiogenesis	NRP1		IFNA6	2	positive	Multiple		CAF	ABCB11
Effector.cell.traffic	CCL4		Activated.CD4.T.cell	RTKN2		Angiogenesis	SEMA5A		IFNA21	2	positive	Multiple		CAF	ABCB4
Effector.cell.traffic	CX3CR1		Activated.CD4.T.cell	SAMSN1		Angiogenesis	RAMP2		IFNA4	2	positive	Multiple		CAF	ABCB5
Effector.cell.traffic	CCL5		Activated.CD4.T.cell	SELL		Angiogenesis	CIB1		IFNA8	2	positive	Multiple		CAF	ABCB6
Effector.cell.traffic	CXCR3		Activated.CD4.T.cell	TRAT1		Angiogenesis	ESM1		IFNA5	2	positive	Multiple		CAF	ABCB7

NK.cells	NKG7		Activated.CD8.T.cell	ADRM1		Angiogenesis	JMJD6		IFNA7	2	positive	Multiple		CAF	ABCB8
NK.cells	CD160		Activated.CD8.T.cell	AHSA1		Angiogenesis	HEY1		IFNA14	2	positive	Multiple		CAF	ABCB9
NK.cells	CD244		Activated.CD8.T.cell	C1GALT1C1		Angiogenesis	ADGRA2		IFNA16	2	positive	Multiple		CAF	ABCC1
NK.cells	NCR1		Activated.CD8.T.cell	CCT6B		Angiogenesis	GREM1		IFNA10	2	positive	Multiple		CAF	ABCC10
NK.cells	KLRC2		Activated.CD8.T.cell	CD37		Angiogenesis	SRPX2		IFNA17	2	positive	Multiple		CAF	ABCC11
NK.cells	KLRK1		Activated.CD8.T.cell	CD3D		Angiogenesis	SOX18		CD40LG	2	positive	Multiple		CAF	ABCC12
NK.cells	CD226		Activated.CD8.T.cell	CD3E		Angiogenesis	PARVA		CD40	2	positive	Multiple		CAF	ABCC13
NK.cells	GZMH		Activated.CD8.T.cell	CD3G		Angiogenesis	RNF213		NT5C	2	positive	Multiple		CAF	ABCC2
NK.cells	GNLY		Activated.CD8.T.cell	CD69		Angiogenesis	E2F8		HMGB1	2	positive	Multiple		CAF	ABCC3
NK.cells	IFNG		Activated.CD8.T.cell	CD8A		Angiogenesis	RSPO3		TLR1	2	positive	Multiple		CAF	ABCC4
NK.cells	KIR2DL4		Activated.CD8.T.cell	CETN3		Angiogenesis	OTU		TLR2	2	positive	Multiple		CAF	ABCC5

							LIN								
NK.cells	EOMES		Activated.CD8.T.cell	CSE1L		Angiogenesis	E2F7		TLR3	2	positive	Multiple		CAF	ABCC6
NK.cells	GZMB		Activated.CD8.T.cell	GEMIN6		Angiogenesis	CCBE1		TLR4	2	positive	Multiple		CAF	ABCC6P1
NK.cells	FGFBP2		Activated.CD8.T.cell	GNLY		Angiogenesis	BMPER		TLR5	2	positive	Multiple		CAF	ABCC8
NK.cells	KLRF1		Activated.CD8.T.cell	GPT2		Angiogenesis	NRARP		TLR6	2	positive	Multiple		CAF	ABCC9
NK.cells	SH2D1B		Activated.CD8.T.cell	GZMA		Angiogenesis	TNFAIP6		TLR7	2	positive	Multiple		CAF	ABCD1
NK.cells	NCR3		Activated.CD8.T.cell	GZMH		Angiogenesis	VCAN		TLR8	2	positive	Multiple		CAF	ABCD2
T.cells	TBX21		Activated.CD8.T.cell	GZMK		Angiogenesis	SPP1		TLR9	2	positive	Multiple		CAF	ABCD3
T.cells	ITK		Activated.CD8.T.cell	IL2RB		Angiogenesis	CCND2		TLR10	2	positive	Multiple		CAF	ABCD4
T.cells	CD3D		Activated.CD8.T.cell	LCK		Angiogenesis	PIK3R1		HLAA	2	positive	Multiple		CAF	ABCE1
T.cells	CD3E		Activated.CD8.T.cell	MPZL1		Angiogenesis	STC1		B2M	2	positive	Multiple		CAF	ABCF1
T.cells	CD3G		Activated.CD8.T.cell	NKG7		Angiogenesis	JAG2		TAP1	2	posit	Mult		CAF	ABCF2

											ive	iple			
T.cells	TRAC		Activated.CD8.T.cell	PIK3IP1		Apoptosis	APAF1		IL10	2	negative	Multiple		CAF	ABCF3
T.cells	TRBC1		Activated.CD8.T.cell	PTRH2		Apoptosis	ATF4		IL4	2	negative	Multiple		CAF	ABCG1
T.cells	TRBC2		Activated.CD8.T.cell	TIMM13		Apoptosis	ATM		IL13	2	negative	Multiple		CAF	ABCG2
T.cells	CD28		Activated.CD8.T.cell	ZAP70		Apoptosis	BAK1		CD3D	3	positive	Multiple		CAF	ABCG4
T.cells	CD5		Activated.dendritic.cell	ABCD1		Apoptosis	BID		CD3E	3	positive	Multiple		CAF	ABCG5
T.cells	TRAT1		Activated.dendritic.cell	C1QC		Apoptosis	BOK		CD3G	3	positive	Multiple		CAF	ABCG8
B.cells	CD19		Activated.dendritic.cell	CAPG		Apoptosis	BRCA2		CD247	3	positive	Multiple		CAF	ABHD1
B.cells	MS4A1		Activated.dendritic.cell	CCL3L3		Apoptosis	CASP2		CD28	3	positive	Multiple		CAF	ABHD10
B.cells	TNFRSF13C		Activated.dendritic.cell	CD207		Apoptosis	CASP4		TNFRSF9	3	positive	Multiple		CAF	ABHD11
B.cells	CR2		Activated.dendritic.cell	CD302		Apoptosis	CASP9		TNFSF9	3	positive	Multiple		CAF	ABHD12
B.cells	TNFRSF17		Activated.dendritic.cell	ATP5B		Apoptosis	DAPK3		TNFRSF4	3	posit	Mult		CAF	ABHD13

											ive	iple			
B.cells	TNFRSF13B		Activated.dendritic.cell	ATP5L		Apoptosis	DDIT3		TNFSF4	3	positive	Multiple		CAF	ABHD14A
B.cells	CD22		Activated.dendritic.cell	ATP6V1A		Apoptosis	E2F2		CD27	3	positive	Multiple		CAF	ABHD14B
B.cells	CD79A		Activated.dendritic.cell	BCL2L1		Apoptosis	EP300		CD70	3	positive	Multiple		CAF	ABHD15
B.cells	CD79B		Activated.dendritic.cell	C1QB		Apoptosis	GSN		TNFRSF14	3	positive	Multiple		CAF	ABHD17A
B.cells	BLK		Activated.dendritic.cell	SNURF		Apoptosis	IFI16		TNFSF14	3	positive	Multiple		CAF	ABHD18
B.cells	FCRL5		Activated.dendritic.cell	SPCS3		Apoptosis	INHBA		CD40	3	positive	Multiple		CAF	ABHD2
B.cells	PAX5		Activated.dendritic.cell	CCNA1		Apoptosis	KRT8		CD40LG	3	positive	Multiple		CAF	ABHD3
B.cells	STAP1		Activated.dendritic.cell	CEACAM8		Apoptosis	LGALS9		TNFRSF18	3	positive	Multiple		CAF	ABHD4
M1.signature	NOS2		Activated.dendritic.cell	NOS2		Apoptosis	NFATC4		TNFSF18	3	positive	Multiple		CAF	ABHD5
M1.signature	TNF		Activated.dendritic.cell	SRA1		Apoptosis	P2RX7		TNFRSF25	3	positive	Multiple		CAF	ABHD6

M1.signature	IL1B		Activated.dendritic.cell	TNFRSF6B		Apoptosis	PMAIP1		TNFSF15	3	positive	Multiple		CAF	ABHD8
M1.signature	SOCS3		Activated.dendritic.cell	TREM1		Apoptosis	POLB		TNFRSF8	3	positive	Multiple		CAF	ABI1
M1.signature	CMKLR1		Activated.dendritic.cell	TREML1		Apoptosis	PPP2R1B		TNFSF8	3	positive	Multiple		CAF	ABI2
M1.signature	IRF5		Activated.dendritic.cell	RHOA		Apoptosis	PPP2R5C		HAVCR1	3	positive	Multiple		CAF	ABI3
M1.signature	IL12A		Activated.dendritic.cell	SLC25A37		Apoptosis	SORT1		TIMD4	3	positive	Multiple		CAF	ABI3BP
M1.signature	IL12B		Activated.dendritic.cell	TNFSF14		Apoptosis	SKIL		SLAMF7	3	positive	Multiple		CAF	ABITRAM

Supplementary table 19. mITH of the 30 cancers in TCGA.

CancerType	mITH	mSig
GBM	0.07	-0.11178
LGG	0.04	-0.28625
CESC	0.1	0.22795
COAD	0.09	0.151713
BRCA	0.09	-0.02165
LUAD	0.23	-0.00728
ESCA	0.205	-0.0909
SARC	0.12	-0.04763
KIRP	0.01	-0.09082
STAD	0.14	-0.13192
SKCM	0.1	0.064219
UCEC	0.04	0.15933
PRAD	0.03	-0.03716
HNSC	0.13	0.209145
KIRC	0.02	-0.26483
LUSC	0.22	0.165222
LIHC	0.07	0.088947
MESO	0.04	0.025181

READ	0.12	0.151448
PAAD	0.08	-0.0733
OV	0.18	0.025356
TGCT	0.11	-0.08947
PCPG	0.015	-0.25376
THCA	0	-0.15727
UVM	0.02	0.174682
UCS	0.19	0.104181
BLCA	0.16	0.200841
ACC	0.06	-0.01993
KICH	0	-0.1495
CHOL	0.06	0.08436

Supplementary table 20. The summary of the other data utilized in this study.

**Rank**	**Data**	**Source**
1	PAN-cancer TCGA	UCSC Xena (http://xena.ucsc.edu/public/)
2	GTEx	UCSC Xena (http://xena.ucsc.edu/public/)
3	Immune cell lines	GSE13906, GSE23371, GSE25320, GSE27291, GSE27838, GSE28490, GSE28698, GSE28726, GSE37750, GSE39889, GSE42058, GSE49910, GSE51540, GSE59237, GSE6863, GSE8059
4	Immune related genes	ImmPort (https://www.immport.org/), 17pathways,1793 genes
5	92 Immunity contexture signatures	PMID: 29628290
6	75 immune checkpoint genes	PMID: 32814346
7	Cancer cell lines	CCLE
8	18 immunotherapy bulk-level cohorts	PMID:30996326,28487385,28487385,30127394,28129544,29033130,28487385,31792460,27956380,26564858,30753825,28487385,32879421,31537801,32472114,30013197,28552987,29443960
9	2 sc-RNA seq datasets with immunotherapy	PMID:30388455,31359002
10	signatures of cancerSEA	PMID: 30329142
11	Signatures of HALLMARK, C6 genesets	MSigDB(https://www.gsea-msigdb.org/gsea/msigdb)
12	data of the ITH	PMID:29628290
13	CancerCell 29 TME signature	PMID:34019806
14	Microenrivonment signature	PMID:31942077
15	anti-cancer immunity cycle signatures	PMID: 30154154

16	ORR data of the 21 cancers in TCGA	PMID: 31436822
17	T cell-inflamed GEP signature	PMID:28650338
18	CAF signature	PMID:30127393
19	TAM M2 signature	PMID:30127393
20	IFNG signature	PMID:30127393
21	CD8 signature	PMID:30127393
22	CD274 signature	PMID:30127393
23	TLS signature	PMID:32238929
24	TLS-melanoma signature	PMID:32238929
25	T cell Dysfunction signature	PMID:30127393
26	T cell exclusion signature	PMID:30127393
27	MDSC signature	PMID:30127393
28	82 sc-RNA datasets for 29 cancer types	Details were summarized in the corresponding tables
29	26 stemness related gene signature	Details were summarized in the corresponding tables
30	13 immunotherapy signature	Details were summarized in the corresponding tables
31	989 cell lines with CERES scores	Depmap (https://depmap.org/portal/download/all/)
32	17 Immune Screening Datasets	Details were summarized in the corresponding tables
33	External validation datasets for prognostic signature	CGGA, GSE16011,GSE108474, LIHC-ICGC,GSE76427,GSE15459
